# Supplementary material for: CryoET shows cofilactin filaments inside the microtubule lumen
Source: EMBO Rep. 2023 Sep 13;24(11):e57264. doi: 10.15252/embr.202357264 (PMC10626427; doi:10.15252/embr.202357264)
Supplement: Supplementary file 1 — Appendix [file EMBR-24-e57264-s002.pdf]

Appendix

Table of contents

Appendix Figure S1. Validation of the microtubule orientation assignment. 2

Appendix Figure S2. Validation of the *Drosophila* α-tubulin acetylase (dTAT) CRISPR knock-out. 3

Appendix Table S1. EMDB entries of actin together with actin-binding proteins solved by helical reconstruction. 4

Appendix Table S2. Acquisition and processing parameters for datasets 1 – 4 (EMPIAR-11450). 5

Appendix Table S3. Acquisition and processing parameters for datasets 5 – 7 (EMPIAR-11451) and dataset 8 (EMPIAR-11452). 6

Appendix Table S4. Acquisition and processing parameters for datasets 9 – 12 (EMPIAR-11453). 7

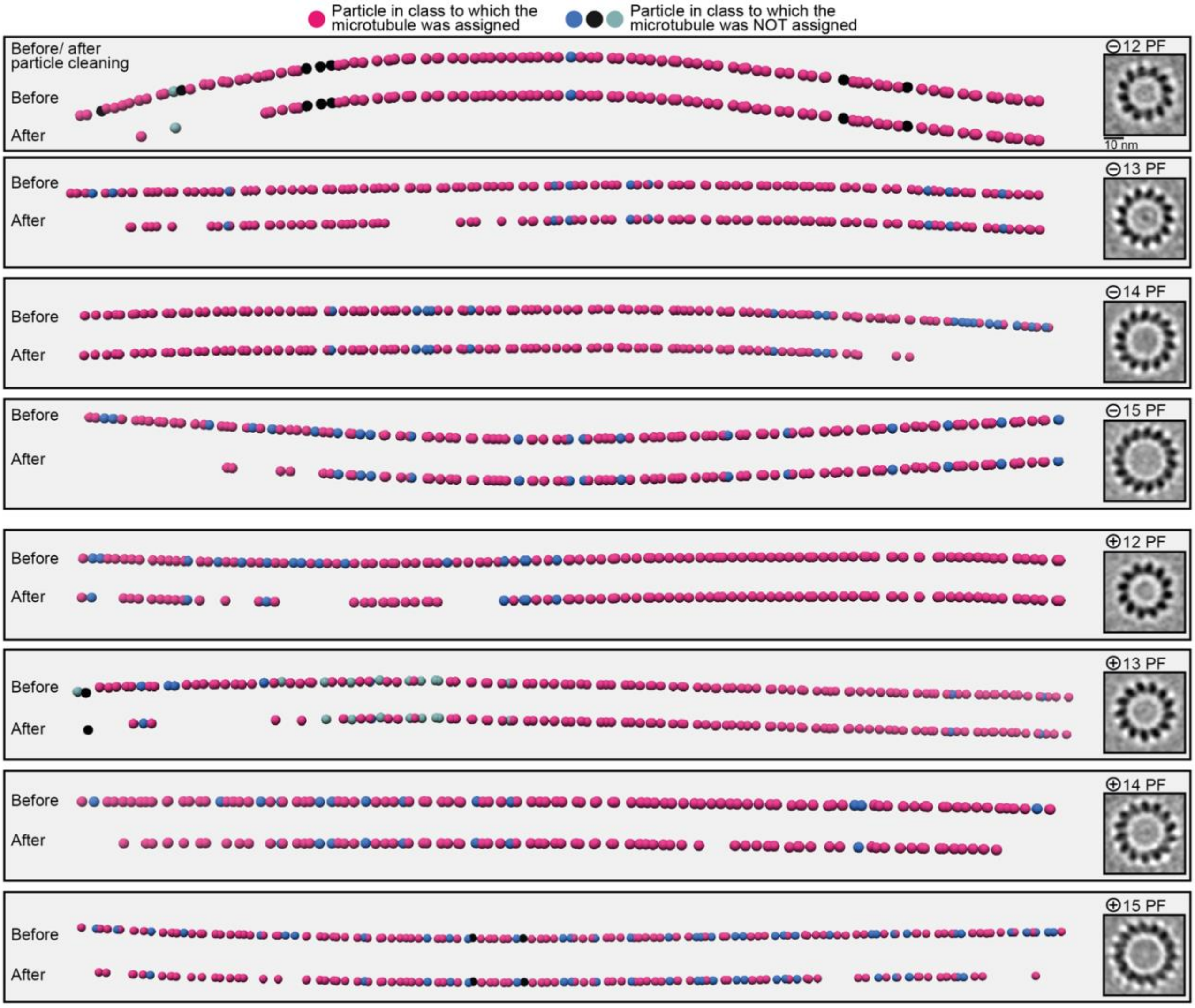

Appendix Figure S1. Validation of the microtubule orientation assignment. Microtubule particle positions and classes are shown for 8 representative microtubules with 12, 13, 14 or 15 protofilaments facing the minus and the plus ends. Pink spheres represent particles in the class, to which the microtubule was assigned. Blue, black and green spheres show particles in classes, to which the microtubule was not assigned. The particle class distribution before (top, 'Before') and after (bottom, 'After') removal of 20% of particles based on their cross-correlation scores ('cleaning') are shown for each microtubule. Projections of subtomogram averages of each of the microtubules before cleaning are shown on the right.

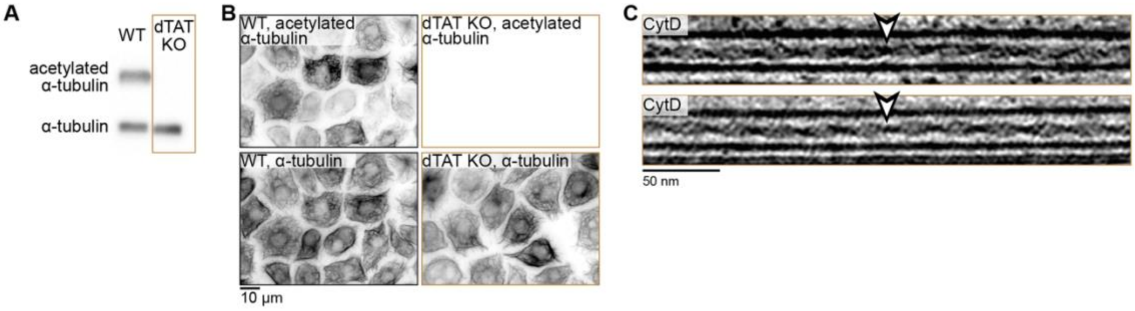

Appendix Figure S2. Validation of the *Drosophila* α-tubulin acetylase (dTAT) CRISPR knock-out. A) Western blot of wild-type (WT, left) or dTAT knock-out (KO, right) cells showing that dTAT KO cells lack acetylated α-tubulin (top). α-tubulin (bottom) was used as loading control. B) Immunofluorescence of WT (left) and dTAT KO (right) cells stained for acetylated α-tubulin (top) or α-tubulin (bottom) showing that dTAT KO cells lack acetylated α-tubulin. C) Tomogram slices of luminal filaments (white arrowheads) in dTAT KO cells showing that they have similar appearance to luminal filaments found in WT cells (Fig. 2A, B).

FAMILY OF ACTIN BINDING PROTEIN	EMD ENTRY NAME	EMD ID	HELICAL RISE [Å]	HELICAL TWIST [°]
**ACTIN (BARE)**	Helical ADP-F-actin	27114	28.1	-166.7
**ACTININ**	alpha-actinin CH1 bound to f-actin	5170	27.7	-166.8
**BETA-SPECTRIN**	F-actin with beta-III-spectrin actin-binding domain	8886	27.2	-166.9
**CATENIN**	alphaE-catenin - F-actin	21925	27.4	-166.9
**CORONIN**	coronin bound f-actin	6101	28.2	-166.3*
**FILAMIN**	FLNaABD-WT bound to phalloidin F-actin	7833	28.1	-166.8
**PLASTIN**	F-actin/Plastin2-ABD2 complex	21155	28.0	-166.5*
**MYOSIN**	cardiac myosin binding protein C C2 domain with f-actin	23497	27.3	-166.5
**TROPOMYOSIN**	F-actin-tropomyosin	6124	27.5	-166.4*
**VINCULIN**	vinculin-actin	6446	27.8	-166.8*
**COFILIN**	cofilactin	6844	27.6	-162.1
**THIS STUDY**	Subtomogram averaging structure of cofilactin filament inside microtubule lumen of Drosophila S2 cell protrusion	16877	28.0	-161.8

Appendix Table S1. EMDB entries of actin together with actin-binding proteins solved by helical reconstruction. Helical twist values marked with a star were deposited as positive values but are displayed with a negative sign because they describe left-handed helix symmetries. Helical parameters were rounded to one decimal.

MICROTUBULE ANALYSIS	**BIOLOGICAL REPLICATE 1**	**BIOLOGICAL REPLICATE 2**		**BIOLOGICAL REPLICATE 3**
**GRID ID**	**DZ4**	**DY2**	**DZ1**	**FB3/FB4**
**DATASET**	**dataset 1**	**dataset 2**	**dataset 3**	**dataset 4**
**GENOTYPE**	WT	WT		WT
**SAMPLE CONDITIONS**	4 h 2.5 µM CytD	4 h 2.5 µM CytD		4 h 5 µM CytD
**INCREMENTS/ NUMBER OF TILTS**	2°/ 61	2°/ 61	3°/ 41	3°/ 41
**FRAMES PER TILT IMAGE**	10	10	14	14
**PIXEL SIZE [Å/PIXEL], DETECTOR**	2.952, K2	2.952, K2		2.952, K2
**TOTAL DOSE [e^-/Å^2]**	123.44	121.68	123.57	122.25
**PROGRAM USED FOR TILT ALIGNMENT**	dautoalign and IMOD	IMOD		IMOD
**ACQUIRED TILT SERIES**	35	22	39	24
**ANALYSED TOMOGRAMS**	33	18	36	22
**NUMBER OF ANALYZED PROTRUSIONS**	34	56		22
**NUMBER OF ANALYZED MICROTUBULES**	109	299		166

Appendix Table S2. Acquisition and processing parameters for datasets 1 – 4 (EMPIAR-11450). These datasets were used for quantification and subtomogram averaging of microtubules in S2 cells.

CYTD & DMSO AND CYTD & TG	**BIOLOGICAL REPLICATE 1**		**BIOLOGICAL REPLICATE 2**		**BIOLOGICAL REPLICATE 3**		**DTAT KNOCK-OUT**
**ACQUISITION DATE/ GRID ID**	**17.06.2022**		**20.07.2022**		**21.07.2021**		**FE2/FE4**
**DATASET**	**dataset 5**		**dataset 6**		**dataset 7**		**dataset 8**
**GENOTYPE**	WT		WT		WT		dTAT KO
**SAMPLE CONDITIONS**	5 h 0.1% (v/v) DMSO, 4 h 2.5 µM CytD	5 h 2 µM TG, 4 h 2.5 µM CytD	5 h 0.1% (v/v) DMSO, 4 h 2.5 µM CytD	5 h 2 µM TG, 4 h 2.5 µM CytD	5 h 0.1% (v/v) DMSO, 4 h 2.5 µM CytD	5 h 2 µM TG, 4 h 2.5 µM	4 h 5 µM CytD
**INCREMENTS/ NUMBER OF TILTS**	3°/ 41		3°/ 41		3°/ 41		3°/ 41
**FRAMES PER TILT IMAGE**	14		14		14		14
**PIXEL SIZE [Å/PIXEL], DETECTOR**	2.952, K2		2.952, K2		2.659, K3		2.952, K2
**TOTAL DOSE [e^-/$Å^2$]**	118.08		122.19		121.54		122.25
**PROGRAM USED FOR TILT ALIGNMENT**	IMOD		AreTomo		AreTomo		IMOD
**ACQUIRED TILT SERIES**	12	14	23	18	25	18	65
**ANALYSED TOMOGRAMS**	12	12	19	14	20	13	62
**NUMBER OF ANALYSED PROTRUSIONS CONTAINING MICROTUBULES**	10	14	18	15	17	13	62
**NUMBER OF ANALYZED MICROTUBULES**	119		139		192		447

Appendix Table S3. Acquisition and processing parameters for datasets 5 – 7 (EMPIAR-11451) and dataset 8 (EMPIAR-11452). Datasets 5 – 7 were used for quantification of luminal filaments and had a final DMSO concentration of 0.125% (v/v). Datasets 5 – 8 were used for subtomogram averaging of luminal filaments. Dataset 8 was collected on the dTAT knock-out mutant.

CONTROL AND COFILIN KNOCK-DOWN	**BIOLOGICAL REPLICATE 1**		**BIOLOGICAL REPLICATE 2**		**BIOLOGICAL REPLICATE 3**		**BIOLOGICAL REPLICATE 4**	
**ACQUISITION DATE/ GRID ID**	**08.12.2022 (part 1) & 17.12.2022 (part 2)**		**16.12.2022**		**02.02.2023 (part 1) & 12.12.2022 (part 2)**		**03.02.2023**	
**DATASET**	**dataset 9**		**dataset 10**		**dataset 11**		**dataset 12**	
**DAY 1 OF KNOCK-DOWN**	01.12.2022		09.12.2022		24.11.2022		17.01.2023	
**GENOTYPE**	Control knock-down	Cofilin knock-down	Control knock-down	Cofilin knock-down	Control knock-down	Cofilin knock-down	Control knock-down	Cofilin knock-down
**SAMPLE CONDITIONS**	5 h 2 µM TG, 2 h 2.5 µM CytD		5 h 2 µM TG, 2 h 2.5 µM CytD		5 h 2 µM TG, 2 h 2.5 µM CytD		5 h 2 µM TG, 2 h 2.5 µM CytD	
**INCREMENTS/ NUMBER OF TILTS**	3°/ 41		3°/ 41		3°/ 41		3°/ 41	
**FRAMES PER TILT IMAGE**	14		14		14		14	
**PIXEL SIZE [Å/PIXEL], DETECTOR**	2.659, K3 (08.12.2022) & 2.952, K2 (17.12.2022)		2.952, K2		2.952, K2 (02.02.2023) & 2.659, K3 (12.12.2022)		2.952, K2	
**TOTAL DOSE [e^-/Å^2]**	113.74 (08.12.2022) & 111.61 (17.12.2022)		111.61		119.66 (part 1) & 111.61 (part 2)		120.91	
**PROGRAM USED FOR TILT ALIGNMENT**	IMOD & AreTomo		AreTomo		IMOD		IMOD	
**ACQUIRED TILT SERIES**	21	20	19	19	22	15	14	13
**ANALYSED TOMOGRAMS**	19	17	16	18	22	13	14	13
**NUMBER OF ANALYSED PROTRUSIONS**	20	17	18	18	22	14	14	13

Appendix Table S4. Acquisition and processing parameters for datasets 9 – 12 (EMPIAR-11453). These datasets were used for quantification of luminal filaments upon control or cofilin knock-down and treatment with CytD & TG (Fig. 3J – L). 8 tilt series from biological replicate 1 (dataset 11) and 3 tilt series from replicate 3 (dataset 13) were collected in different acquisition settings. All samples had a final DMSO concentration of 0.125% (v/v) DMSO.
