# Supplementary material for: CryoET shows cofilactin filaments inside the microtubule lumen
Source: EMBO Rep. 2023 Sep 13;24(11):e57264. doi: 10.15252/embr.202357264 (PMC10626427; doi:10.15252/embr.202357264)
Supplement: Supplementary file 2 — Expanded View Figures PDF [file EMBR-24-e57264-s011.pdf]

Expanded View Figures

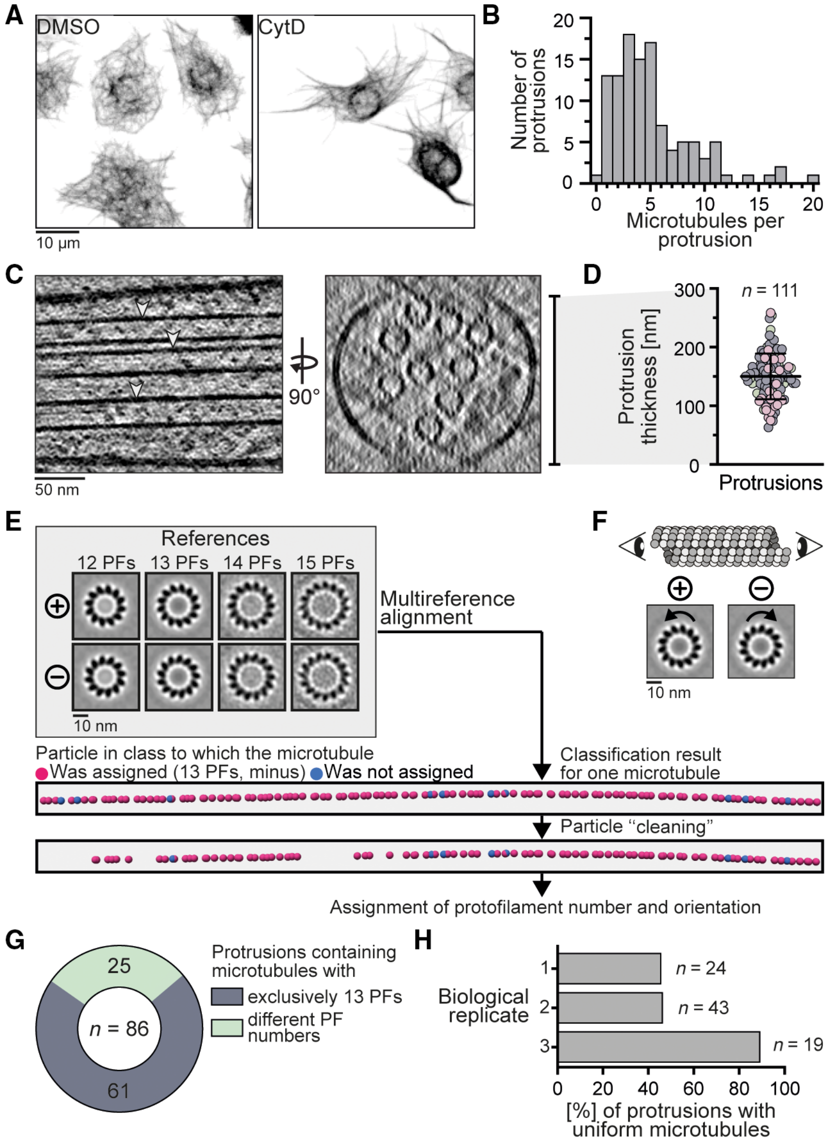

Figure EV1. Analysis of microtubules in induced S2 cell protrusions.

- A Immunofluorescence staining of α-tubulin in S2 cells shows that CytD treatment induces the formation of protrusions which contain microtubules. Control cells treated with a vehicle (DMSO) are shown on the left.
- B Histogram of the microtubule number per protrusion in targeted regions quantified from tomograms.
- C Tomogram slices of a protrusion in side-view (left) and cross-section (right) showing that microtubules (arrowheads) form parallel arrays.
- D Protrusion thicknesses (150.1 ± 39.0 nm, mean ± s.d., *N* = 3, colored accordingly) measured from cross-sections of tomograms exemplified by the bar in (C).
- E Classification workflow to determine microtubule protofilament number and orientation. Projections of references used in the multireference alignment (top, projections of plus-end facing microtubules are also used in Fig 1D, those of 13 protofilament microtubules are reused in (F)) and particle positions/classes from a representative microtubule (bottom) before and after removal of particles based on their cross-correlation score ("cleaning") are shown.
- F Cartoon and exemplary projections of microtubule subtomogram averages showing that microtubules appear to rotate in anti-clockwise and clockwise directions when viewed from the plus and minus end, respectively (Sosa & Chrétien, 1998). Axial 13 protofilament microtubule projections were reused from Fig 1D and (E).
- G Pie chart showing that the majority but not all protrusions contained exclusively 13 protofilament microtubules. 25 protrusions had microtubules with two or three different protofilament numbers.
- H Percentages of protrusions containing uniformly oriented microtubules from three biological replicates show that microtubule uniformity can be variable across datasets. *N*, *n*: number of biological replicates (*N*), analyzed protrusions (*n*).

Source data are available online for this figure.

 © 2023 MRC Laboratory of Molecular Biology and The Authors

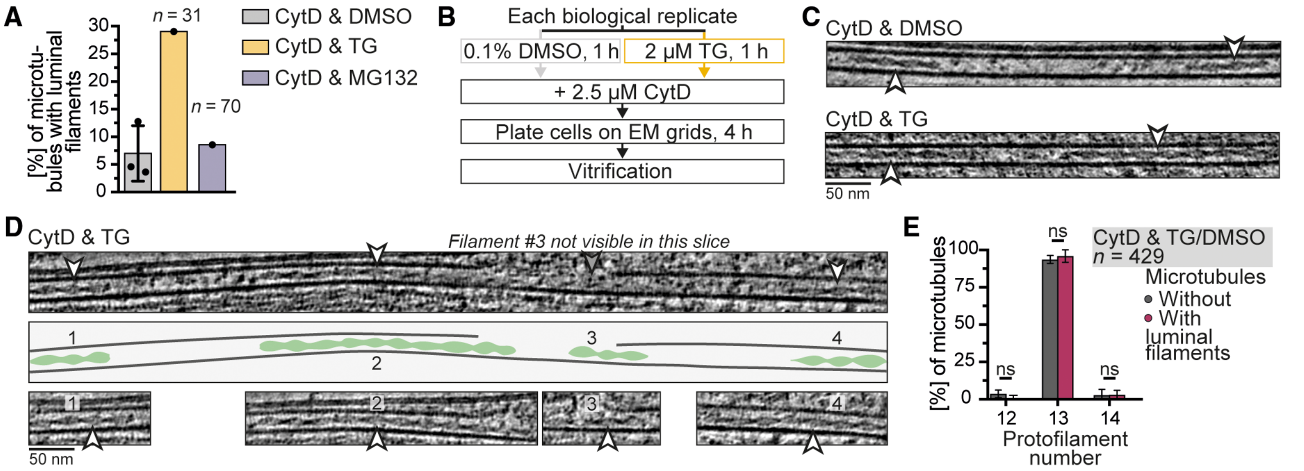

Figure EV2. Analysis of filaments inside the microtubule lumen.

A Percentage of microtubules with at least one luminal filament after treatment with CytD & TG (29.0%) or CytD & MG132 (8.6%) in a preliminary experiment (N = 1). The control (CytD & DMSO, 7.0 ± 5.0%) from Fig 2C is shown for comparison.

B Workflow for the preparation of biological replicates that were treated with CytD & DMSO/TG and used for the analysis of luminal filaments.

C Tomogram slices of microtubules with two luminal filaments (white arrowheads) from samples with indicated treatments.

D Tomogram slices and cartoon of a microtubule that contains four luminal filaments. Top panel shows an overview image of the microtubule where three out of four luminal filaments are visible (arrowheads). The third filament is not clear in this tomographic slice. Middle panel shows a cartoon of the microtubule and luminal filaments. The four luminal filaments are also depicted in the bottom panel with numbers corresponding to those in the cartoon. The example is from cells treated with CytD & TG.

E Percentage of microtubules without (gray) or with (red) luminal filaments that have 12 (3.6 ± 2.6% without, 1.0 ± 1.7% with), 13 (93.6 ± 2.6% without, 96.0 ± 4.3% with) or 14 (2.8 ± 3.8% without, 3.1 ± 2.9% with) protofilaments from CytD & DMSO and CytD & TG treated cells showing no significant difference (mean ± s.d., N = 3, 2-way ANOVA test with comparisons between microtubules of each protofilament number with and without luminal filaments: 12: P = 0.53, 13: P = 0.85, 14: P > 0.99, all ns). N, n: number of biological replicates (N), analyzed microtubules (n).

Source data are available online for this figure.

Figure EV3. Structural analysis of luminal filaments.

A, B Fourier transforms of the luminal filament (A) and cytoplasmic f-actin (B) in Fig 3A and B. Layer lines and corresponding frequencies are indicated. Left panels show a slice (A) or projection (B) of the filament used to generate Fourier transform.

C Cross-over distances of luminal filaments (27.4 ± 0.1 nm, N = 3) and cytoplasmic f-actin (35.7 ± 2.4 nm, N = 4) measured from Fourier transforms showing a significant difference (unpaired t-test, P = 0.0022, **).

D Workflow for subtomogram averaging of luminal filaments.

E Positions and orientations of particles from three microtubules (gray) and luminal filaments (red/orange) in a representative protrusion. Arrows point towards the plus/barbed ends of the filaments. Luminal filament 1 is oriented with its barbed end towards the microtubule minus end while luminal filaments 2 and 3 have their barbed ends oriented towards the microtubule plus end.

F Quantification showing that 46.8 ± 5.5% of luminal filaments had their barbed (plus) end pointing towards the plus end of the surrounding microtubule (N = 3).

G Averages after alignments with a mask (green, left) including the luminal filament (top) or the microtubule (bottom). In the first case, the luminal filament but not the surrounding microtubule was well resolved. In the latter case, the microtubule but not the luminal filament was well resolved.

H Fourier shell correlation of the luminal filament subtomogram average.

I, J Tomogram slices of individual (I) and bundled (J) putative cofilactin filaments in the cytoplasm of S2 protrusions from samples with indicated treatments.

K, L Exemplary subtomogram classification (K) and quantification (L) of luminal filaments from control and cofilin knock-down cells. Classes assigned to cofilactin, bare f-actin and other morphologies are labeled green, blue and gray, respectively. Averages of the percentages of particles with each morphology for each N are shown in Fig 3L.

M Percentage of luminal filament length resembling cofilactin (89.8 ± 7.2% control, 25.2 ± 21.8% cofilin dsRNA) assessed from visual inspection of the tomograms showing a decrease upon cofilin knock-down (N = 4, unpaired t-test, P = 0.0013, **). Panel on the right shows examples of cofilactin and non-cofilactin morphologies. 41.1 and 22.1 µm luminal filament length were sampled in control and cofilin knock-down cells, respectively.

N Tomogram slices of luminal filaments from cofilin knock-down cells with morphologies different from cofilactin or bare f-actin.

O Percentage of microtubules with luminal filaments in control (17.8 ± 7.7%) or cofilin (11.7 ± 3.4%) knock-down cells showing a non-significant reduction (N = 4, unpaired t-test, P = 0.2011, ns).

P Tomogram slice and cartoon of a luminal filament that transitions from a cofilactin (left) to a non-cofilactin (right) morphology.

Q Examples of linking densities (white arrowheads) between luminal filaments and the surrounding microtubule wall. Graphs and numbers show mean ± s.d. N, n: number of biological replicates (N) and analyzed filaments (n).

Source data are available online for this figure.

© 2023 MRC Laboratory of Molecular Biology and The Authors

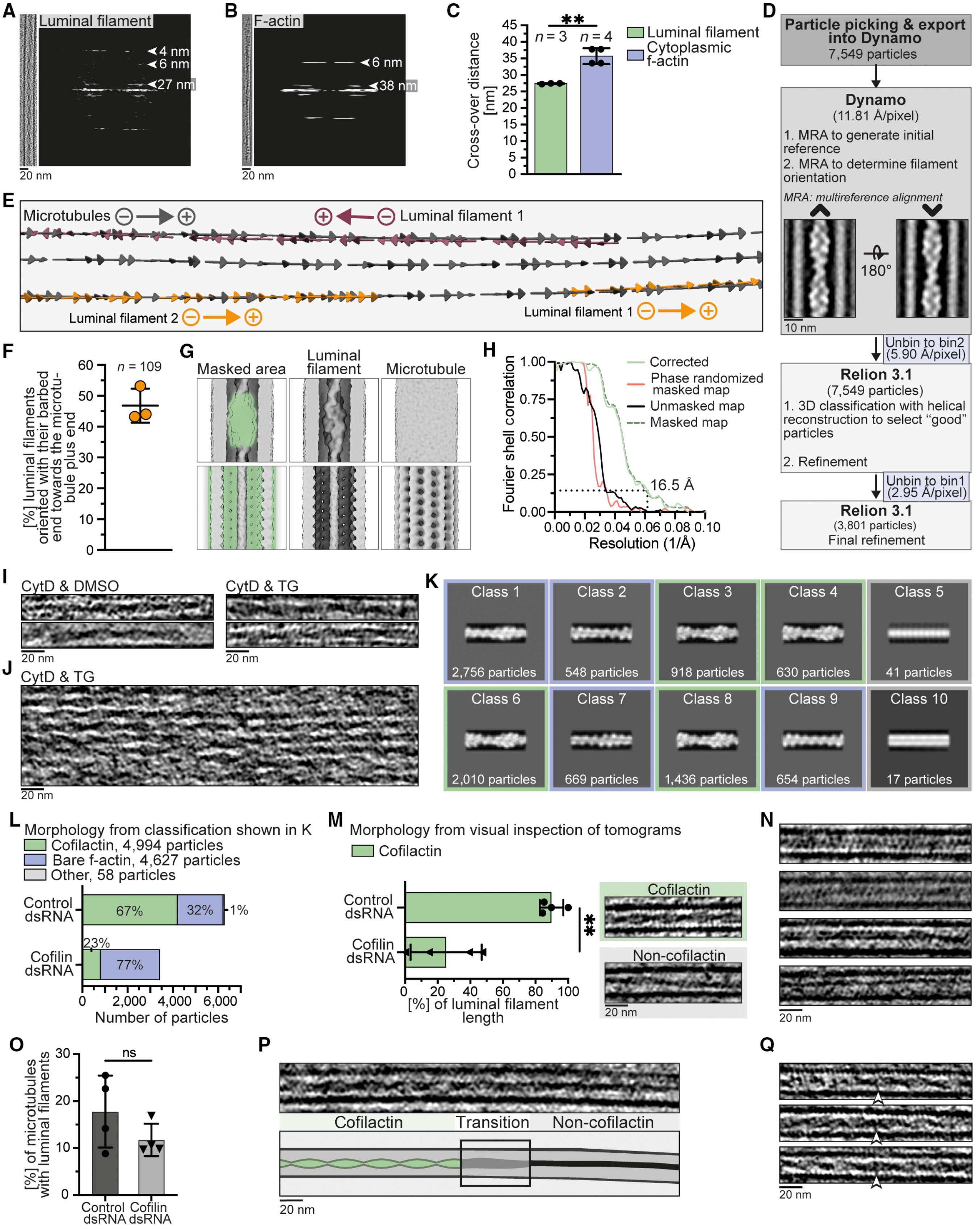

Figure EV3.

 © 2023 MRC Laboratory of Molecular Biology and The Authors
