# Supplementary material for: CryoET shows cofilactin filaments inside the microtubule lumen
Source: EMBO Rep. 2023 Sep 13;24(11):e57264. doi: 10.15252/embr.202357264 (PMC10626427; doi:10.15252/embr.202357264)
Supplement: Supplementary file 4 — Movie EV2 [file EMBR-24-e57264-s009.zip › EMBOR-2023-57264V1_MovieEV2/MovieEV2_Readme.rtf]

Movie EV2. Tomogram of an induced S2 cell protrusion.Slice view through the tomographic volume of a 4-times binned and deconvolved tomogram. The movie was generated from TIFF images of ~5.9 nm thick tomographic slices generated in IMOD (slicer window) and assembled in ImageJ.This tomogram (TS_096) belongs to dataset 3 (EMPIAR-11450) and the corresponding MRC file has been uploaded to the EMDB (EMD-16693).
