# Supplementary material for: CryoET shows cofilactin filaments inside the microtubule lumen
Source: EMBO Rep. 2023 Sep 13;24(11):e57264. doi: 10.15252/embr.202357264 (PMC10626427; doi:10.15252/embr.202357264)
Supplement: Supplementary file 5 — Movie EV3 [file EMBR-24-e57264-s006.zip › EMBOR-2023-57264V1_MovieEV3/MovieEV3_Readme.rtf]

Movie EV3. Tomogram of an induced S2 cell protrusion after SERCA inhibition with TG.Slice view through the tomographic volume of a 4-times binned and deconvolved tomogram of a cell protrusion after treatment with the SERCA inhibitor TG showing two luminal filaments inside microtubules (pink arrows). The movie was generated from TIFF images of ~5.3 nm thick tomographic slices generated in IMOD (slicer window) and assembled in ImageJ. Arrows were added with Adobe Premiere Pro.This tomogram (TS_297) belongs to dataset 7 (EMPIAR-11451) and the corresponding MRC file has been uploaded to the EMDB (EMD-16720).
