# Supplementary material for: CryoET shows cofilactin filaments inside the microtubule lumen
Source: EMBO Rep. 2023 Sep 13;24(11):e57264. doi: 10.15252/embr.202357264 (PMC10626427; doi:10.15252/embr.202357264)
Supplement: Supplementary file 6 — Movie EV4 [file EMBR-24-e57264-s005.zip › EMBOR-2023-57264V1_MovieEV4/MovieEV4_Readme.rtf]

Movie EV4. Tomogram of an induced S2 cell protrusion from a cofilin knock-down cell. Slice view through the tomographic volume of a 4-times binned and deconvolved tomogram of a cofilin knock-down cell protrusion showing luminal filaments with cofilactin (pink arrows) and non-cofilactin (blue arrows) morphology inside microtubules. The movie was generated from TIFF images of ~5.9 nm thick tomographic slices generated in IMOD (slicer window) and assembled in ImageJ. Arrow were added in Adobe Premiere Pro.This tomogram (TS_346) belongs to dataset 9 (EMPIAR-11453) and the corresponding MRC file has been uploaded to the EMDB (EMD-16800).
