# Supplementary material for: CryoET shows cofilactin filaments inside the microtubule lumen
Source: EMBO Rep. 2023 Sep 13;24(11):e57264. doi: 10.15252/embr.202357264 (PMC10626427; doi:10.15252/embr.202357264)
Supplement: Supplementary file 7 — Source Data for Expanded View and Appendix [file EMBR-24-e57264-s003.zip › EMBOR-2023-57264V1_SourceDataForExpandedViewAndAppendix/Figure_EV1/A/FigEV1A_Readme.rtf]

- 02-DMSO_022.czi is the raw data file for Fig. EV1A (left). 04-CytD_013.czi is the raw data file for Fig. EV1A (right). - SUM_02-DMSO_022-1.tif is them summed z-stack (ImageJ option ‘sum slices’) and cropped of 02-DMSO_022.czi- SUM_024-CytD_013-2-1.tif is them summed z-stack (ImageJ option ‘sum slices’) and cropped of 04-CytD_013.czi- PNG files were generated from TIF files and used to prepare figures.
