# Supplementary material for: CryoET shows cofilactin filaments inside the microtubule lumen
Source: EMBO Rep. 2023 Sep 13;24(11):e57264. doi: 10.15252/embr.202357264 (PMC10626427; doi:10.15252/embr.202357264)
Supplement: Supplementary file 7 — Source Data for Expanded View and Appendix [file EMBR-24-e57264-s003.zip › EMBOR-2023-57264V1_SourceDataForExpandedViewAndAppendix/Figure_EV1/F/FigEV1F_Readme.rtf]

PNG images of axial projections of 13 protofilament microtubule averages (plus- and minus-end-facing) were generated from the .em files in IMOD. The scale bar is given in pixels with the pixel size 11.808 Å/pixel.
