# Supplementary material for: CryoET shows cofilactin filaments inside the microtubule lumen
Source: EMBO Rep. 2023 Sep 13;24(11):e57264. doi: 10.15252/embr.202357264 (PMC10626427; doi:10.15252/embr.202357264)
Supplement: Supplementary file 7 — Source Data for Expanded View and Appendix [file EMBR-24-e57264-s003.zip › EMBOR-2023-57264V1_SourceDataForExpandedViewAndAppendix/Figure_EV3/N/FigEV3N_Readme.rtf]

Images of tomogram slices were generated in IMOD from tomograms 1. TS_442 (datasets 10, EMPIAR-11453)2. TS_528 (dataset 12, EMPIAR-11453)3. TS_346 (dataset 9, EMPIAR-11453)4. TS_509 (dataset 11, EMPIAR-11453)
