# Supplementary material for: CryoET shows cofilactin filaments inside the microtubule lumen
Source: EMBO Rep. 2023 Sep 13;24(11):e57264. doi: 10.15252/embr.202357264 (PMC10626427; doi:10.15252/embr.202357264)
Supplement: Supplementary file 7 — Source Data for Expanded View and Appendix [file EMBR-24-e57264-s003.zip › EMBOR-2023-57264V1_SourceDataForExpandedViewAndAppendix/Figure_EV3/M/FigEV3M_Readme.rtf]

- Images of tomogram slices were generated in IMOD from tomograms TS_361 of (cofilactin morphology, datasets 9, uploaded to EMPIAR-11453) and TS_380 (non-cofilactin morphology, dataset 11, uploaded to EMPIAR-11453) as PNG image. - Excel spreadsheets contain raw quantifications shown in Fig. EV3M, O- Prism file contains the graph shown in Fig. EV3M
