# Supplementary material for: CryoET shows cofilactin filaments inside the microtubule lumen
Source: EMBO Rep. 2023 Sep 13;24(11):e57264. doi: 10.15252/embr.202357264 (PMC10626427; doi:10.15252/embr.202357264)
Supplement: Supplementary file 7 — Source Data for Expanded View and Appendix [file EMBR-24-e57264-s003.zip › EMBOR-2023-57264V1_SourceDataForExpandedViewAndAppendix/Figure_EV3/J/FigEV2J_Readme.rtf]

Image was generated in IMOD from tomogram TS_155 of (datasets 6, uploaded to EMPIAR-11451) as PNG image. 
