# Supplementary material for: CryoET shows cofilactin filaments inside the microtubule lumen
Source: EMBO Rep. 2023 Sep 13;24(11):e57264. doi: 10.15252/embr.202357264 (PMC10626427; doi:10.15252/embr.202357264)
Supplement: Supplementary file 7 — Source Data for Expanded View and Appendix [file EMBR-24-e57264-s003.zip › EMBOR-2023-57264V1_SourceDataForExpandedViewAndAppendix/Figure_EV3/D/FigEV3D_Readme.rtf]

Images were generated in IMOD in the slicer window from volumes with a pixel size of 11.808 Å/pixel. The 10 pixel scale bar therefore corresponds to 11.81 nm.
