# Supplementary material for: CryoET shows cofilactin filaments inside the microtubule lumen
Source: EMBO Rep. 2023 Sep 13;24(11):e57264. doi: 10.15252/embr.202357264 (PMC10626427; doi:10.15252/embr.202357264)
Supplement: Supplementary file 7 — Source Data for Expanded View and Appendix [file EMBR-24-e57264-s003.zip › EMBOR-2023-57264V1_SourceDataForExpandedViewAndAppendix/Figure_EV2/C/FigEV2C_Readme.rtf]

Images were generated in IMOD from tomograms TS_214 (Top, CytD & DMSO, dataset 5, uploaded to EMPIAR-11451) and tomogram TS_250 (Bottom, CytD & TG, dataset 6, uploaded to EMPIAR-11451) as PNG images. 
