# Supplementary material for: CryoET shows cofilactin filaments inside the microtubule lumen
Source: EMBO Rep. 2023 Sep 13;24(11):e57264. doi: 10.15252/embr.202357264 (PMC10626427; doi:10.15252/embr.202357264)
Supplement: Supplementary file 7 — Source Data for Expanded View and Appendix [file EMBR-24-e57264-s003.zip › EMBOR-2023-57264V1_SourceDataForExpandedViewAndAppendix/Appendix/Appendix_Figure_S3/C/AppendixFigS3_Readme.rtf]

Images were generated in IMOD from tomograms TS_006 and TS_054 of dTAT KO cells (tomograms are from dataset 8 and have been uploaded to EMPIAR-11452).
