# Supplementary material for: CryoET shows cofilactin filaments inside the microtubule lumen
Source: EMBO Rep. 2023 Sep 13;24(11):e57264. doi: 10.15252/embr.202357264 (PMC10626427; doi:10.15252/embr.202357264)
Supplement: Supplementary file 7 — Source Data for Expanded View and Appendix [file EMBR-24-e57264-s003.zip › EMBOR-2023-57264V1_SourceDataForExpandedViewAndAppendix/Figure_EV1/E/microtubule_subtomogramAverage_projections/FigEV1E_Readme_microtubule_subtomogramAverageProjections.rtf]

PNG files were generated in IMOD as PNGs from the raw EM files. Raw files have a pixel size of 11.808 Å/pixel. The 5 pixel scale bar in PNG files corresponds to 5.9 nm.
