# Supplementary material for: CryoET shows cofilactin filaments inside the microtubule lumen
Source: EMBO Rep. 2023 Sep 13;24(11):e57264. doi: 10.15252/embr.202357264 (PMC10626427; doi:10.15252/embr.202357264)
Supplement: Supplementary file 7 — Source Data for Expanded View and Appendix [file EMBR-24-e57264-s003.zip › EMBOR-2023-57264V1_SourceDataForExpandedViewAndAppendix/Figure_EV1/E/ParticlePositions_and_Classes_DZ1_mod190/FigEV1E_MicrotubuleParticlePositionsClasses_Readme.rtf]

Images of microtubule particle positions and classes were generated in Chimera using the Plugin ‘Place Object’. Images generated in Chimera are PNG files. Motive lists used to generate these images are .em files.
