# Supplementary figures and images for: CryoET shows cofilactin filaments inside the microtubule lumen (part 2 of 2)
Source: EMBO Rep. 2023 Sep 13;24(11):e57264. doi: 10.15252/embr.202357264 (PMC10626427; doi:10.15252/embr.202357264)

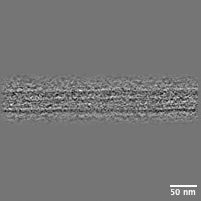

Supplement: Supplementary file 7 — Source Data for Expanded View and Appendix [file EMBR-24-e57264-s003.zip › EMBOR-2023-57264V1_SourceDataForExpandedViewAndAppendix/Figure_EV3/C/LuminalFilaments/FB3_TS_022/TS_022.mrc_11.81Apx_newstack_TRIM_masked_project_scale50.png]

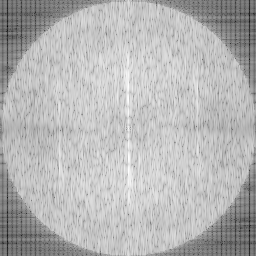

Supplement: Supplementary file 7 — Source Data for Expanded View and Appendix [file EMBR-24-e57264-s003.zip › EMBOR-2023-57264V1_SourceDataForExpandedViewAndAppendix/Figure_EV3/C/LuminalFilaments/FB3_TS_022/TS_022.mrc_11.81Apx_newstack_TRIM_masked_project_FFT_withLinePlotProfile.tif]

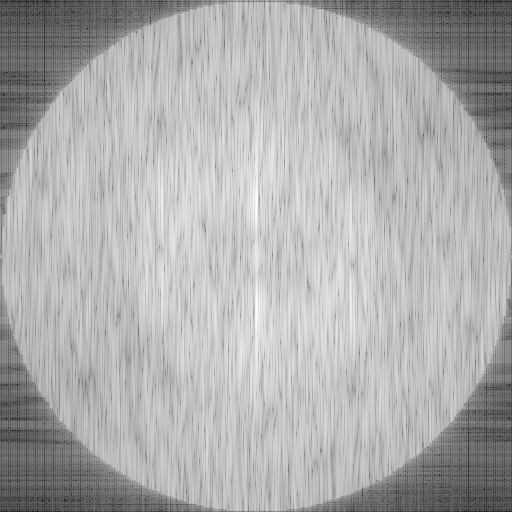

Supplement: Supplementary file 7 — Source Data for Expanded View and Appendix [file EMBR-24-e57264-s003.zip › EMBOR-2023-57264V1_SourceDataForExpandedViewAndAppendix/Figure_EV3/C/Cytoplasmic_F-actin/DY2_TS_55/TS_55.mrc_11.81Apx_Trim_Rot2_bx285_masked_project_FFT.tif]

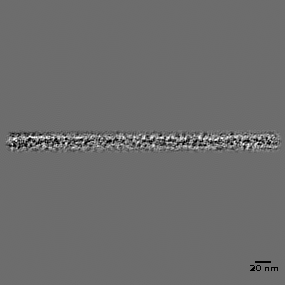

Supplement: Supplementary file 7 — Source Data for Expanded View and Appendix [file EMBR-24-e57264-s003.zip › EMBOR-2023-57264V1_SourceDataForExpandedViewAndAppendix/Figure_EV3/C/Cytoplasmic_F-actin/DY2_TS_55/TS_55.mrc_11.81Apx_Trim_Rot2_bx285_masked_project.png]

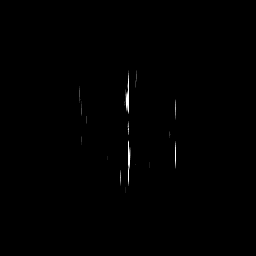

Supplement: Supplementary file 7 — Source Data for Expanded View and Appendix [file EMBR-24-e57264-s003.zip › EMBOR-2023-57264V1_SourceDataForExpandedViewAndAppendix/Figure_EV3/C/Cytoplasmic_F-actin/DY2_TS_55/TS_55.mrc_11.81Apx_Trim_Rot2_bx285_masked_project_FFT_BinBy2.png]

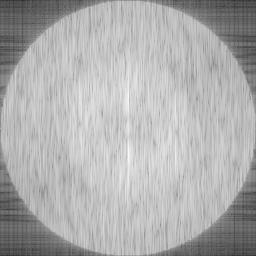

Supplement: Supplementary file 7 — Source Data for Expanded View and Appendix [file EMBR-24-e57264-s003.zip › EMBOR-2023-57264V1_SourceDataForExpandedViewAndAppendix/Figure_EV3/C/Cytoplasmic_F-actin/DY2_TS_55/TS_55.mrc_11.81Apx_Trim_Rot2_bx285_masked_project_FFT_BinBy2.tif]

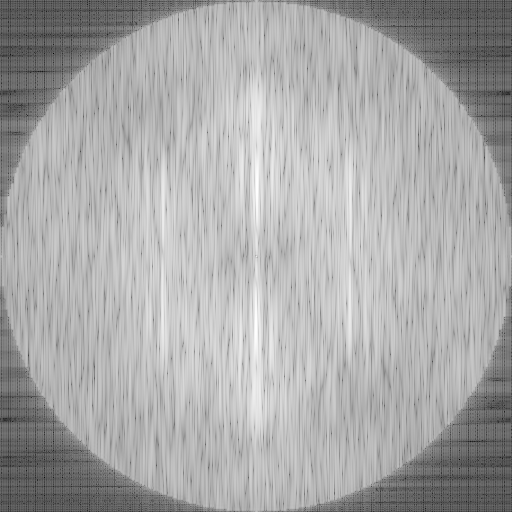

Supplement: Supplementary file 7 — Source Data for Expanded View and Appendix [file EMBR-24-e57264-s003.zip › EMBOR-2023-57264V1_SourceDataForExpandedViewAndAppendix/Figure_EV3/C/Cytoplasmic_F-actin/220617_TS_215_cytoActin/TS_215.mrc_11.81Apx_Trim_Rot2_bx285_masked_project_FFT.tif]

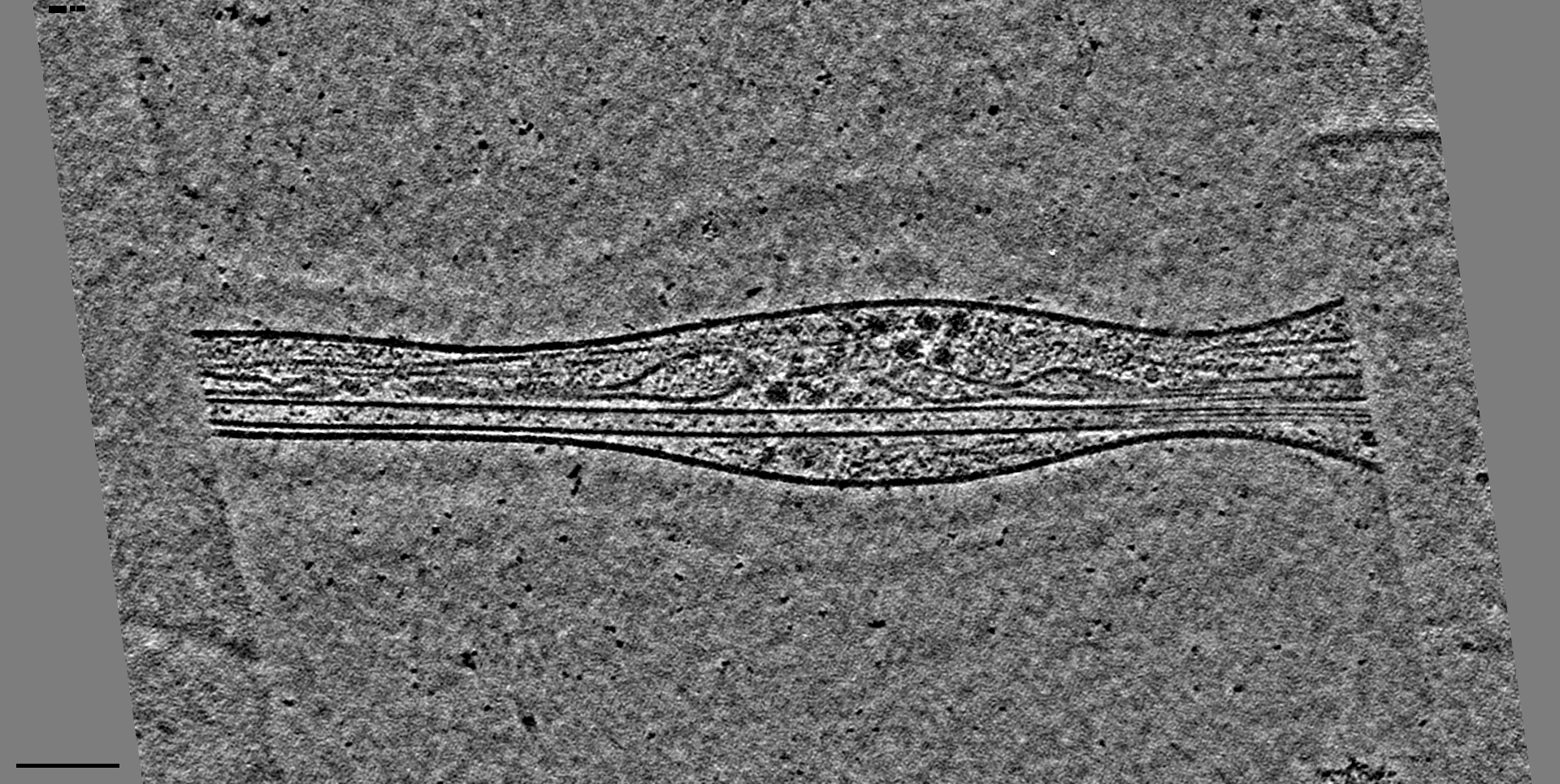

Supplement: Supplementary file 9 — Source Data for Figure 1 [file EMBR-24-e57264-s012.zip › EMBOR-2023-57264V1_SourceDataForFigure1B-E/C/Fig1C_TS_08_overviewPicture_01_scale100.png]

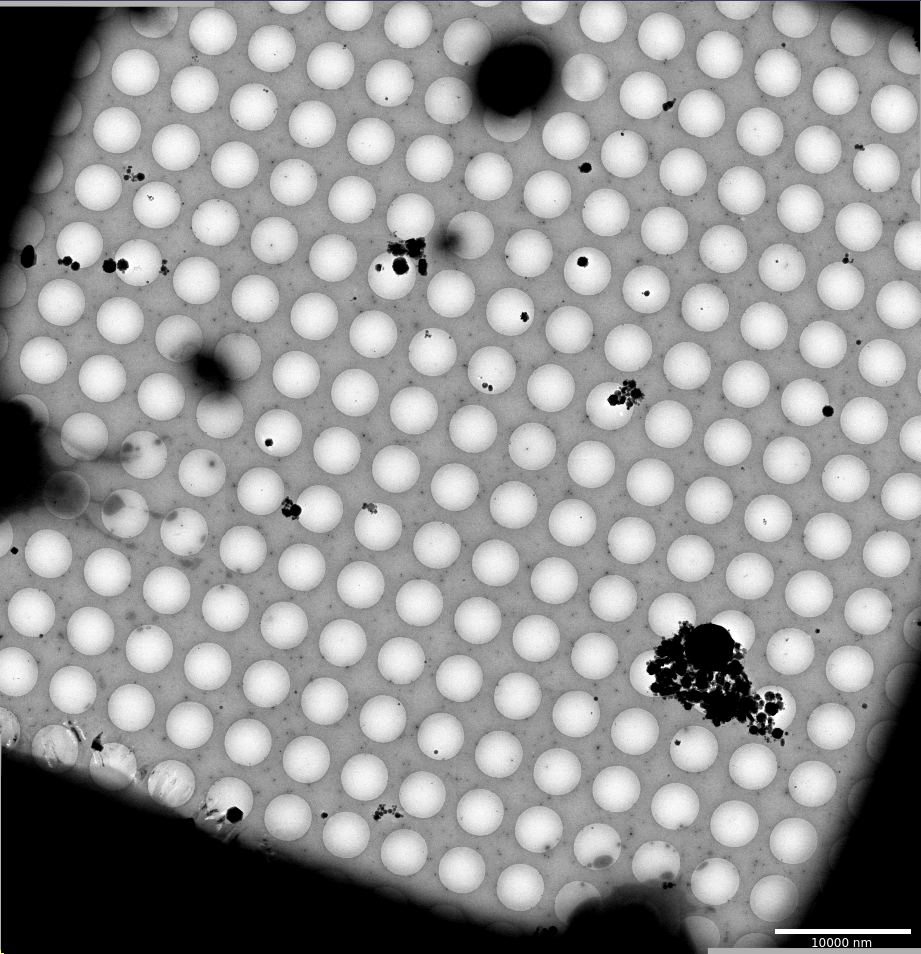

Supplement: Supplementary file 9 — Source Data for Figure 1 [file EMBR-24-e57264-s012.zip › EMBOR-2023-57264V1_SourceDataForFigure1B-E/B/DZ4_2A_TS_08.png]

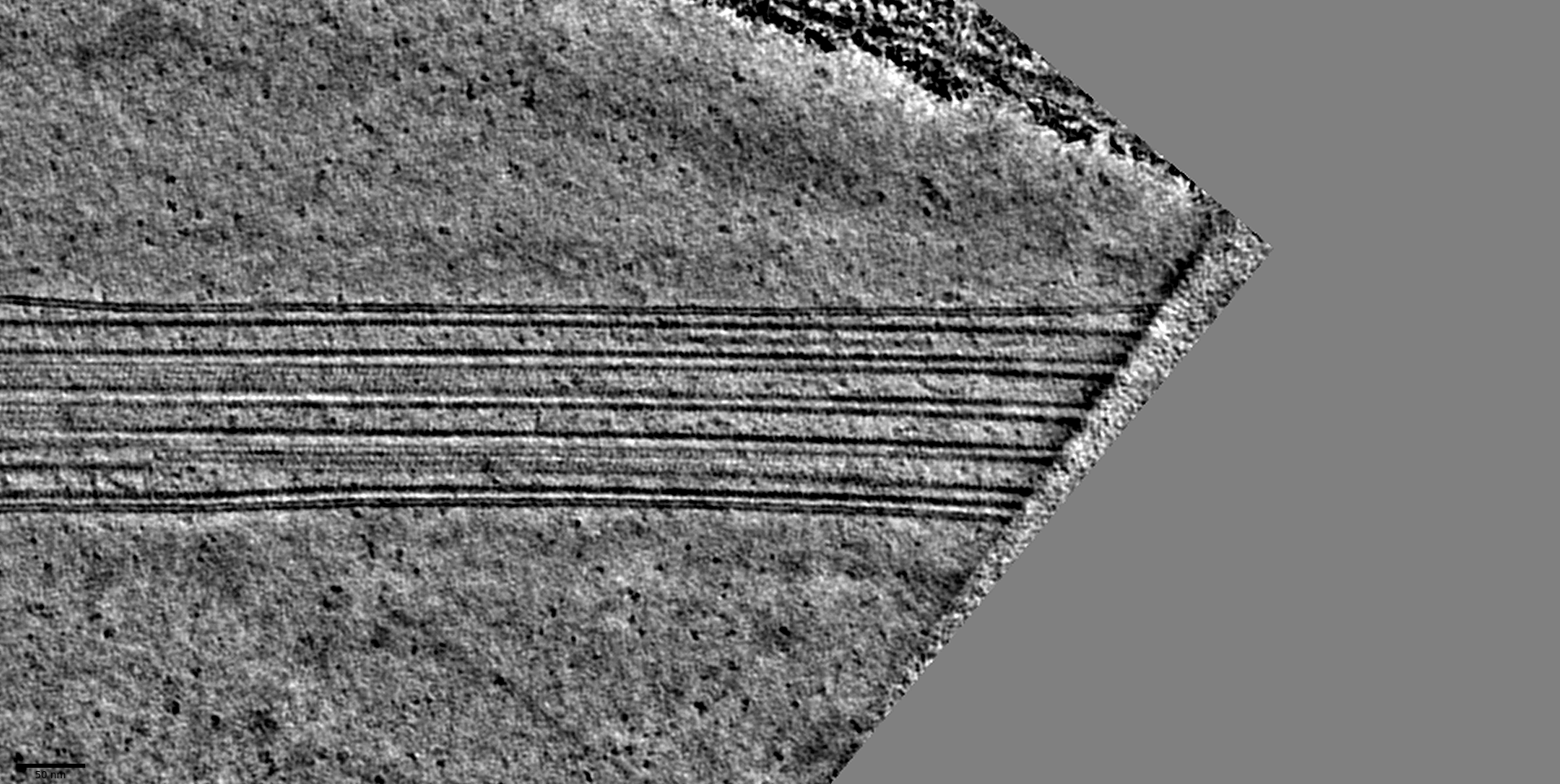

Supplement: Supplementary file 10 — Source Data for Figure 2 [file EMBR-24-e57264-s007.zip › EMBOR-2023-57264V1_SourceDataForFigure2A-E/A/210725_DZ1_TS_070_LumFil.png]

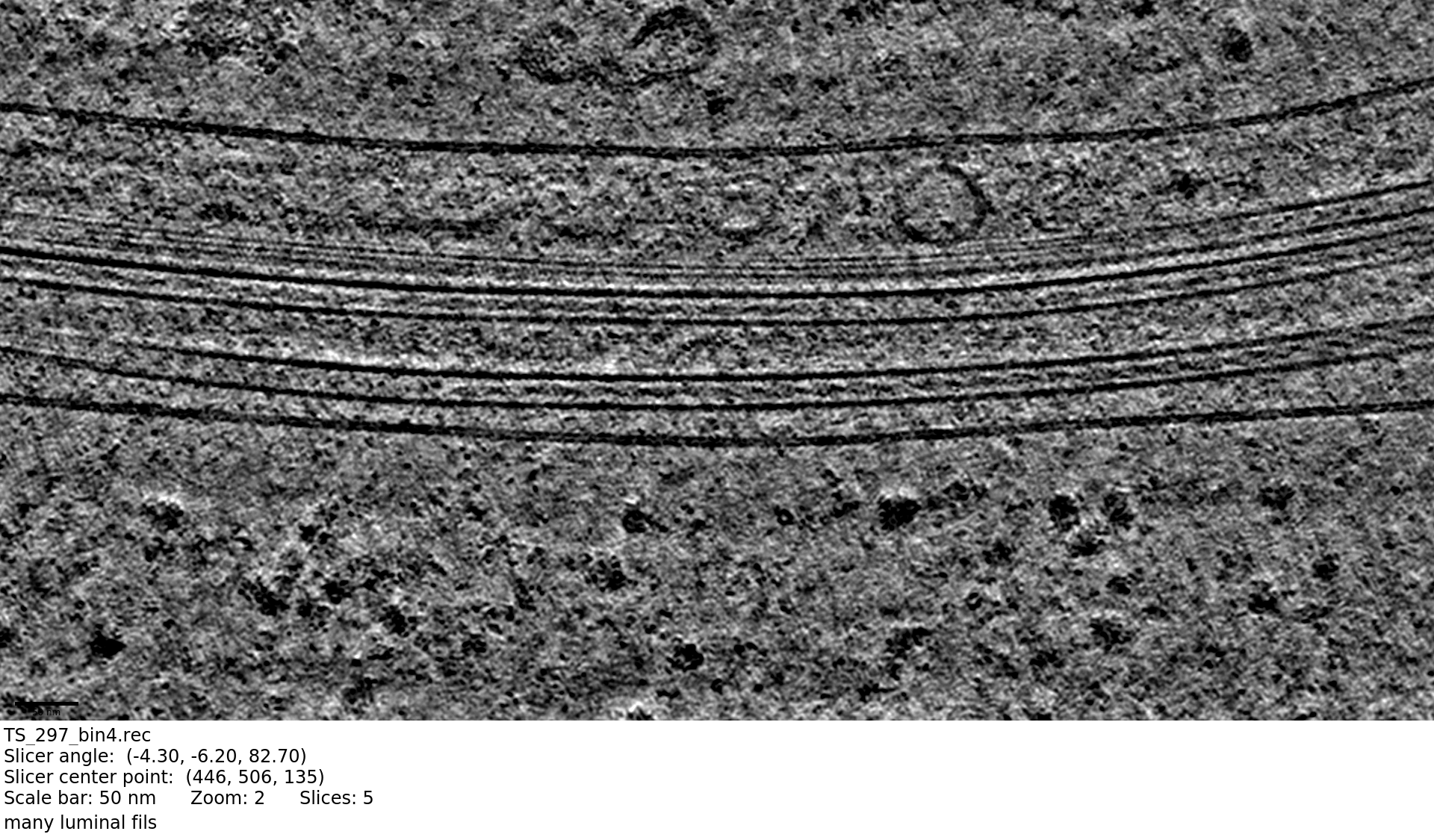

Supplement: Supplementary file 10 — Source Data for Figure 2 [file EMBR-24-e57264-s007.zip › EMBOR-2023-57264V1_SourceDataForFigure2A-E/B/220721_TS_297_2-3FilsFOV.png]

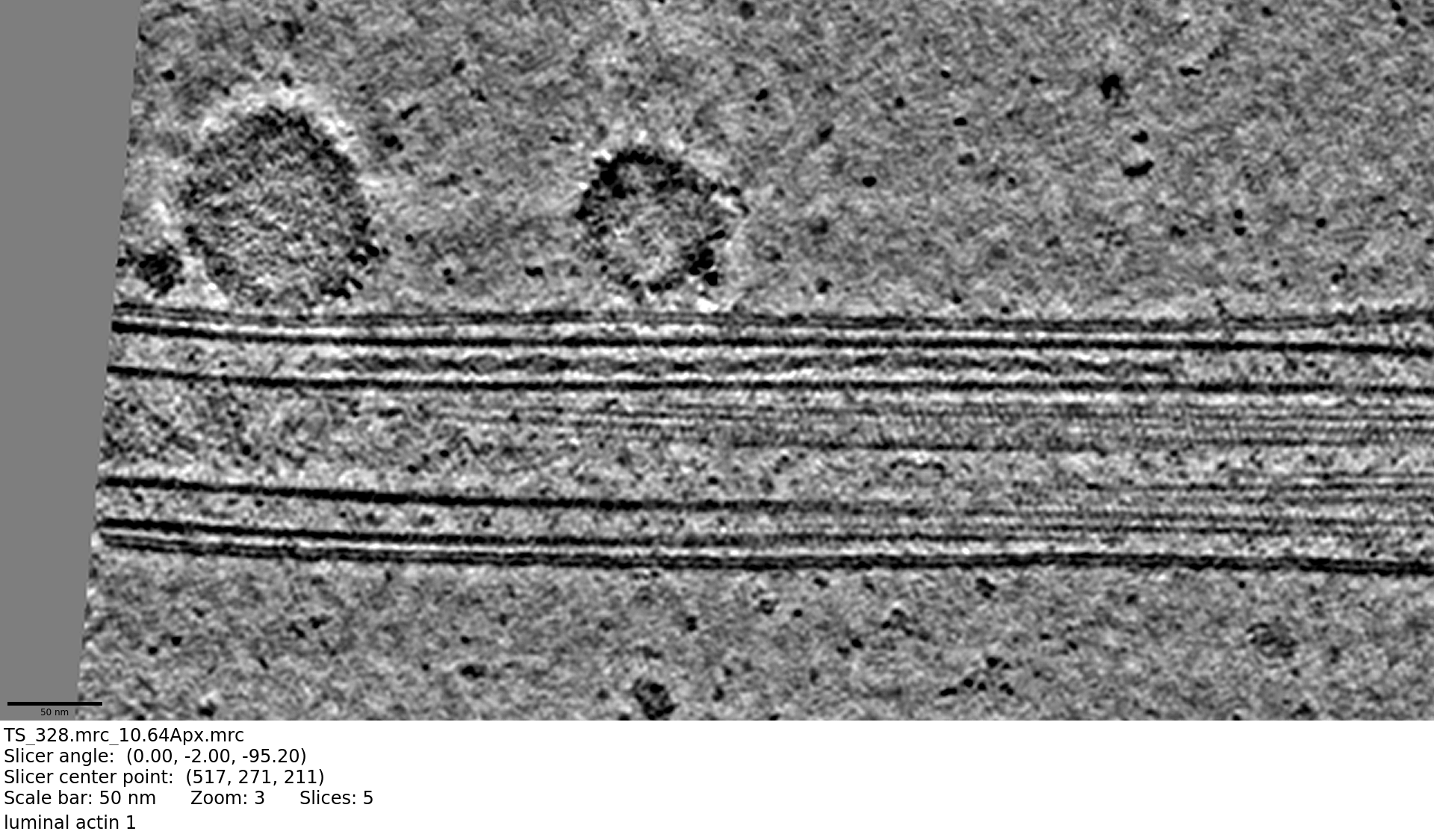

Supplement: Supplementary file 11 — Source Data for Figure 3 [file EMBR-24-e57264-s001.zip › EMBOR-2023-57264V1_SourceDataForFigure3A-B_H-L/A/Fig3A_LumFil_TS_328.png]

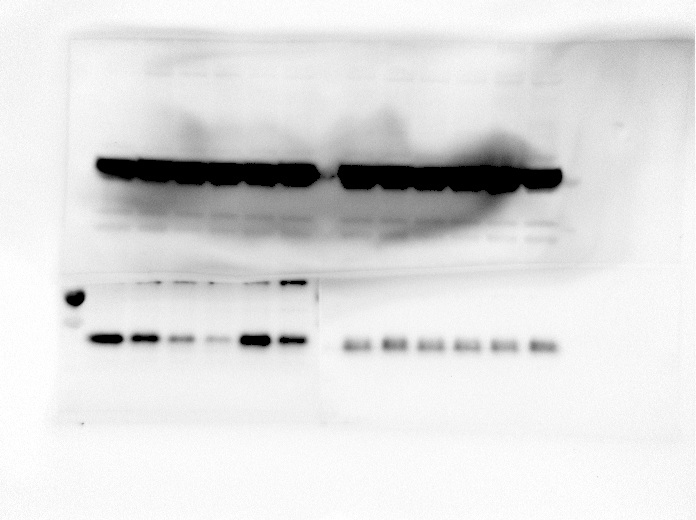

Supplement: Supplementary file 11 — Source Data for Figure 3 [file EMBR-24-e57264-s001.zip › EMBOR-2023-57264V1_SourceDataForFigure3A-B_H-L/H/Blot2_P-cofilin.png]

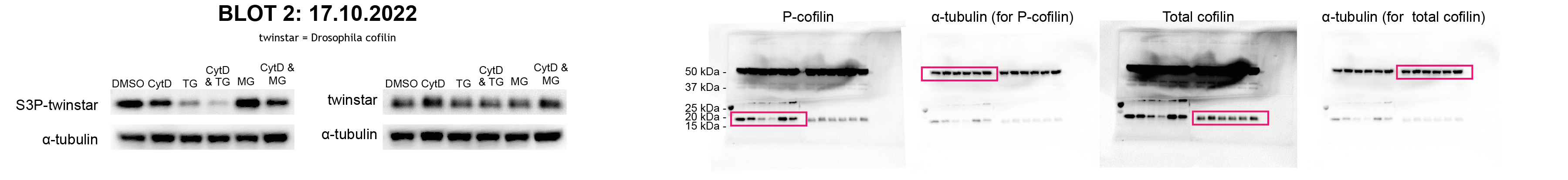

Supplement: Supplementary file 11 — Source Data for Figure 3 [file EMBR-24-e57264-s001.zip › EMBOR-2023-57264V1_SourceDataForFigure3A-B_H-L/H/Blot2_Annotated_Western_221017.png]

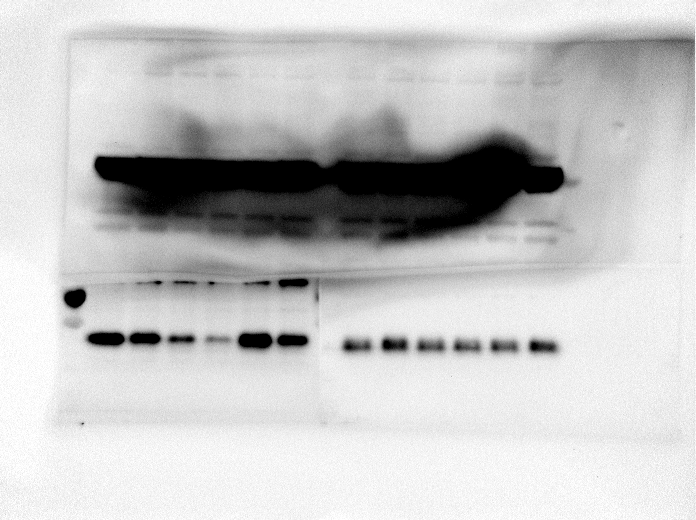

Supplement: Supplementary file 11 — Source Data for Figure 3 [file EMBR-24-e57264-s001.zip › EMBOR-2023-57264V1_SourceDataForFigure3A-B_H-L/H/Blot2_TotalCofilin.png]

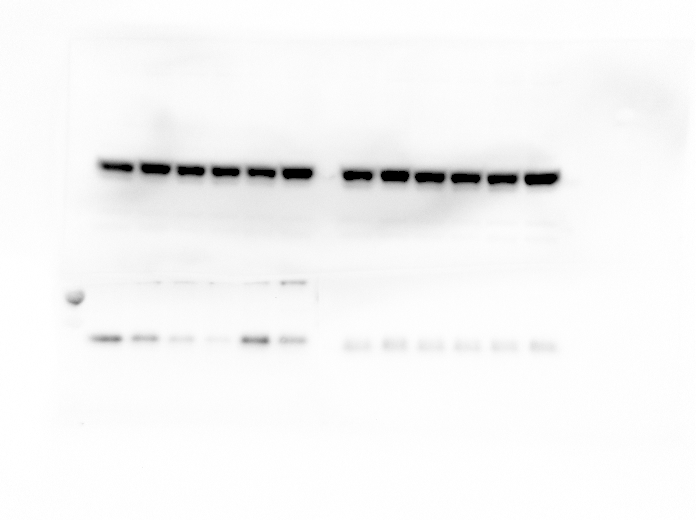

Supplement: Supplementary file 11 — Source Data for Figure 3 [file EMBR-24-e57264-s001.zip › EMBOR-2023-57264V1_SourceDataForFigure3A-B_H-L/H/Blot2_tubulin.png]

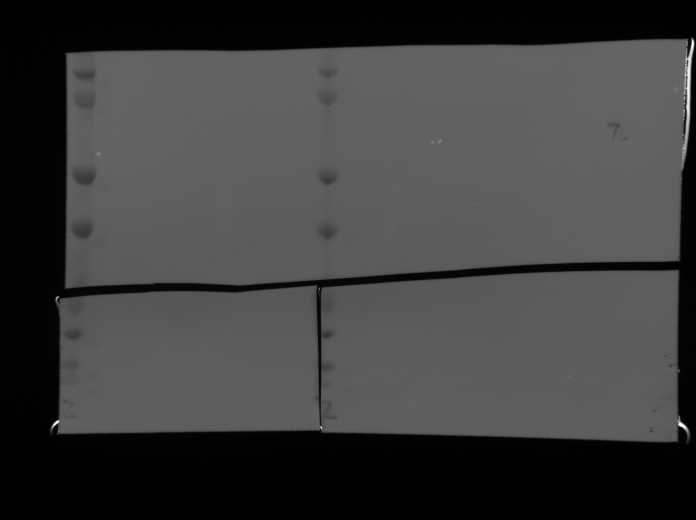

Supplement: Supplementary file 11 — Source Data for Figure 3 [file EMBR-24-e57264-s001.zip › EMBOR-2023-57264V1_SourceDataForFigure3A-B_H-L/H/Blot2_coliometric.png]

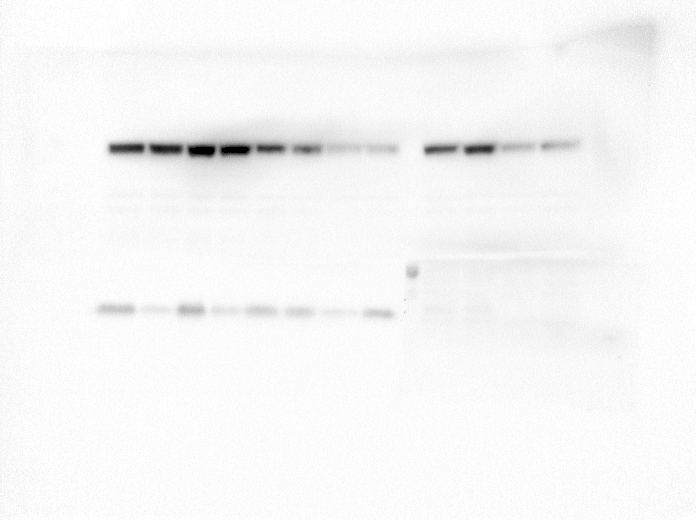

Supplement: Supplementary file 11 — Source Data for Figure 3 [file EMBR-24-e57264-s001.zip › EMBOR-2023-57264V1_SourceDataForFigure3A-B_H-L/J/Fig3J_alpha-tubulin_Dataset9.png]

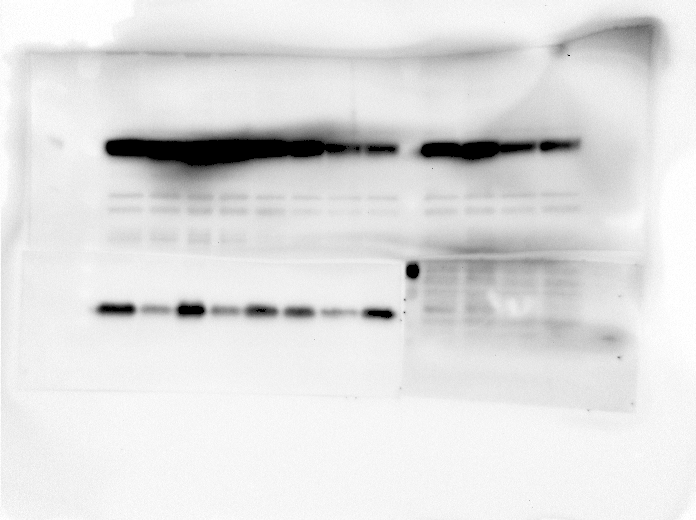

Supplement: Supplementary file 11 — Source Data for Figure 3 [file EMBR-24-e57264-s001.zip › EMBOR-2023-57264V1_SourceDataForFigure3A-B_H-L/J/Fig3J_cofilin_Dataset9.png]

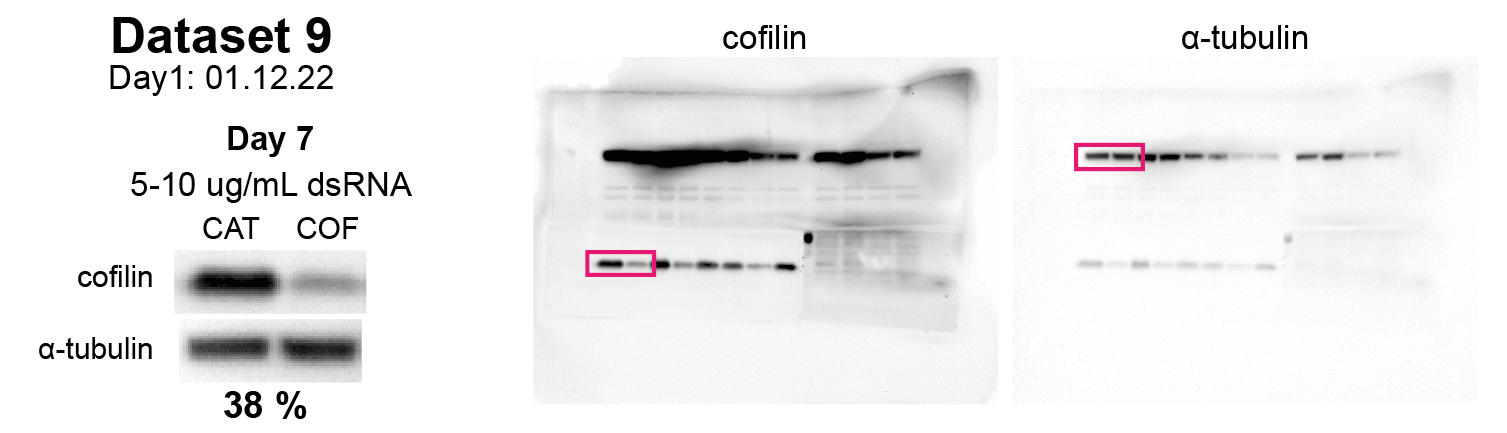

Supplement: Supplementary file 11 — Source Data for Figure 3 [file EMBR-24-e57264-s001.zip › EMBOR-2023-57264V1_SourceDataForFigure3A-B_H-L/J/Dataset9_Annotated_Western.png]

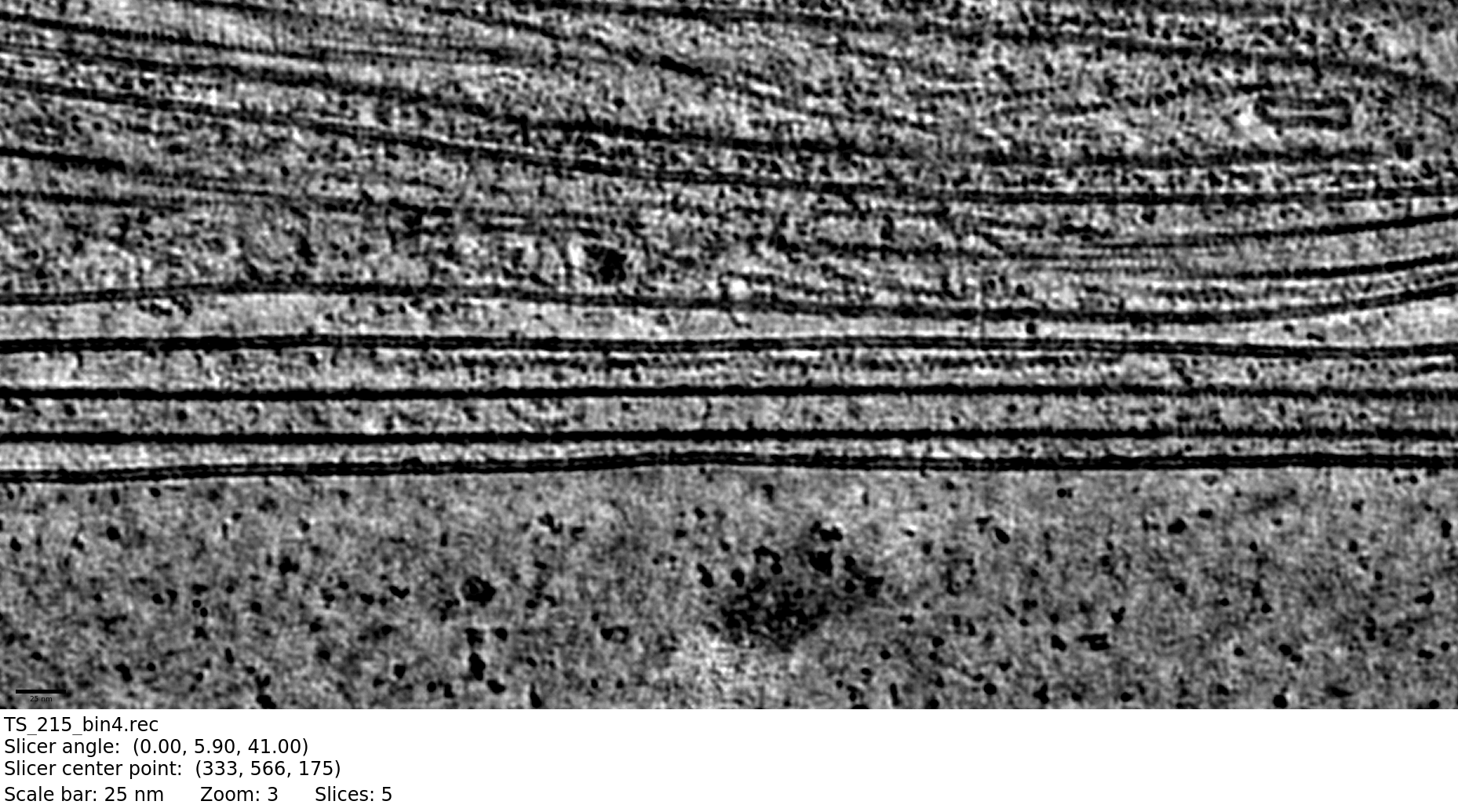

Supplement: Supplementary file 11 — Source Data for Figure 3 [file EMBR-24-e57264-s001.zip › EMBOR-2023-57264V1_SourceDataForFigure3A-B_H-L/B/Fig3B_Cytoplasmic_F-actin_TS_215.png]

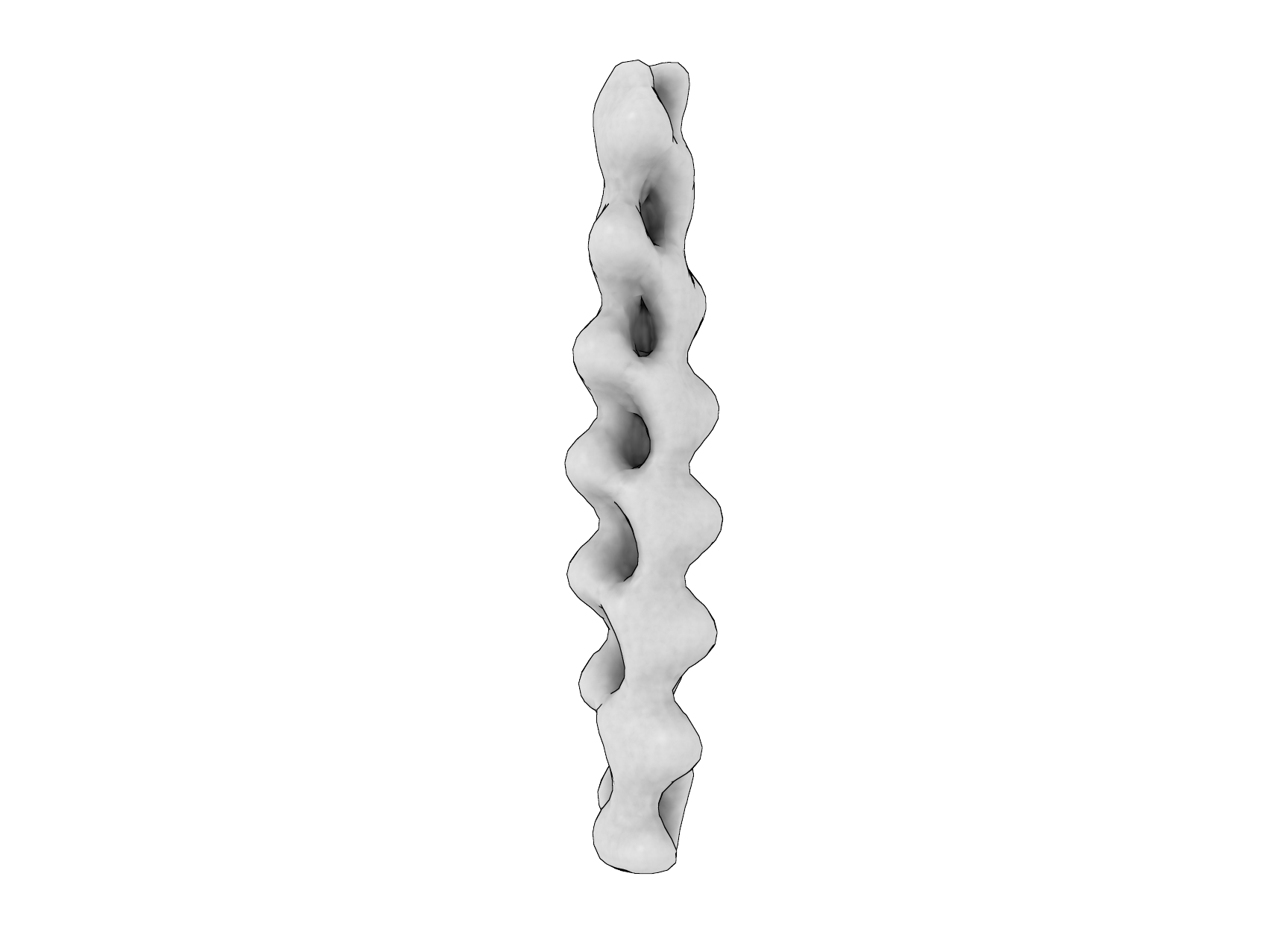

Supplement: Supplementary file 11 — Source Data for Figure 3 [file EMBR-24-e57264-s001.zip › EMBOR-2023-57264V1_SourceDataForFigure3A-B_H-L/L/Fig3L_bare_f-actin_job091_class007_transBG.png]

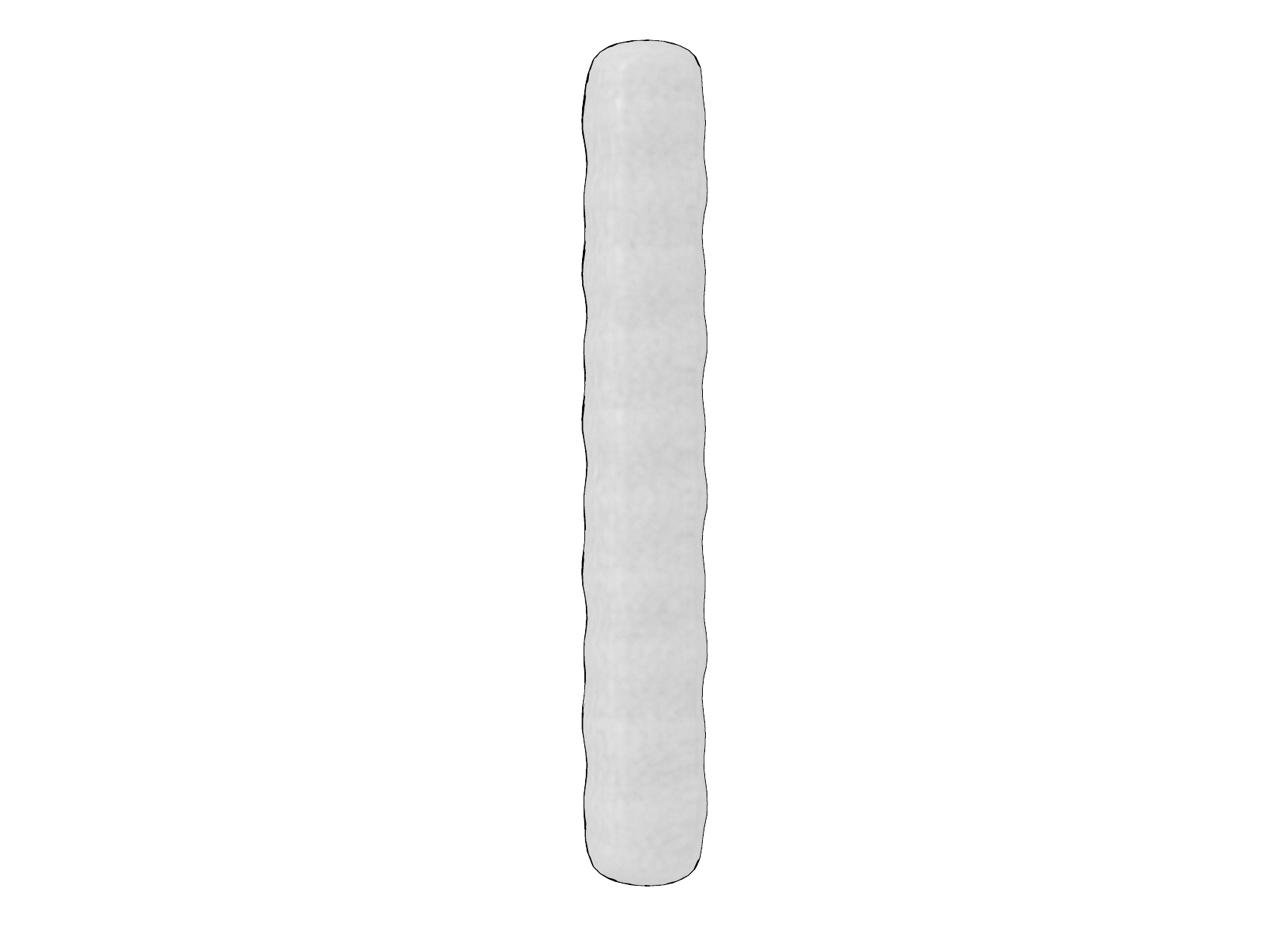

Supplement: Supplementary file 11 — Source Data for Figure 3 [file EMBR-24-e57264-s001.zip › EMBOR-2023-57264V1_SourceDataForFigure3A-B_H-L/L/Fig3L_Other_job091_class005_transBG.png]

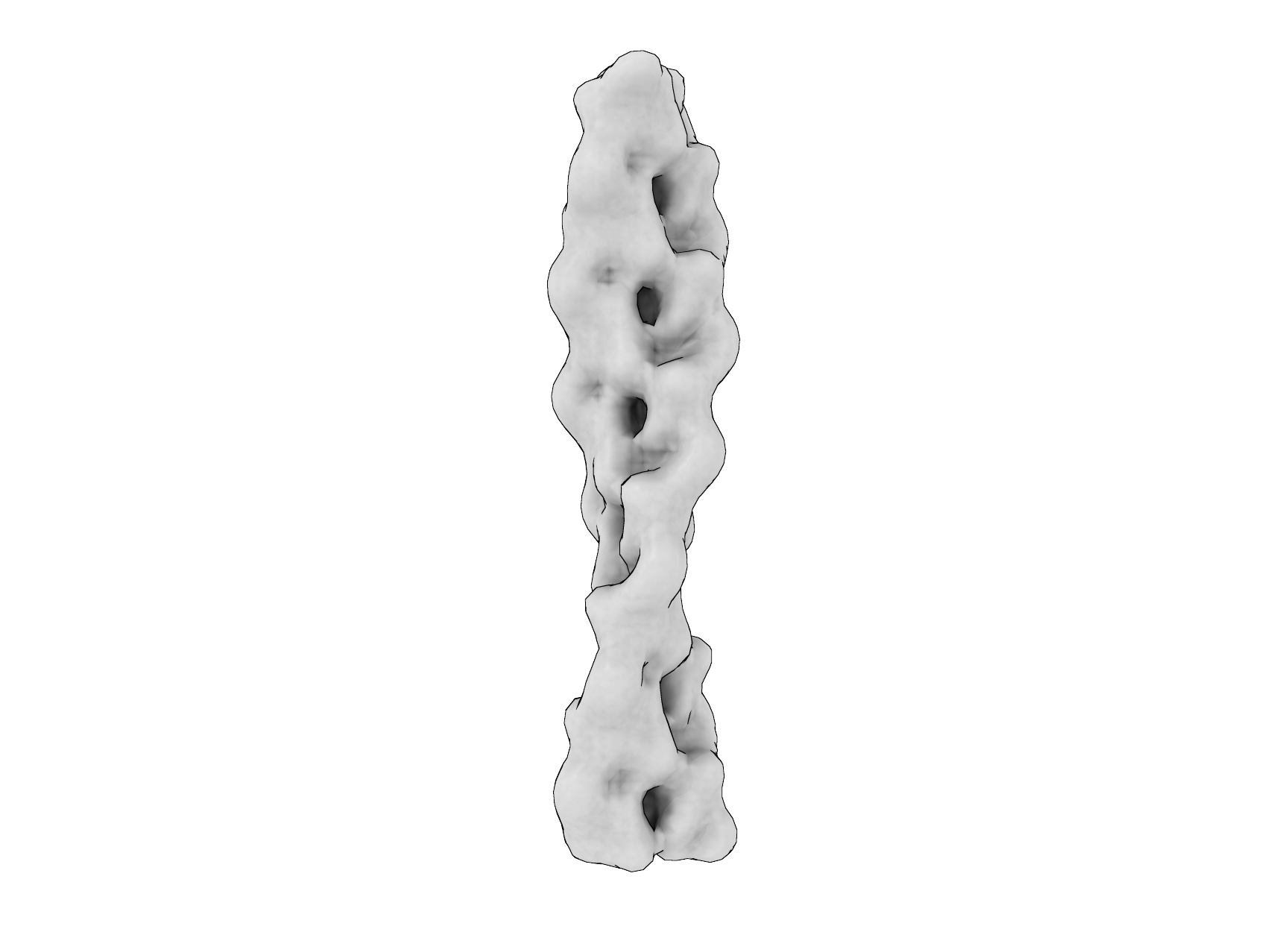

Supplement: Supplementary file 11 — Source Data for Figure 3 [file EMBR-24-e57264-s001.zip › EMBOR-2023-57264V1_SourceDataForFigure3A-B_H-L/L/Fig3L_Cofilactin_job091_class003_transBG.png]

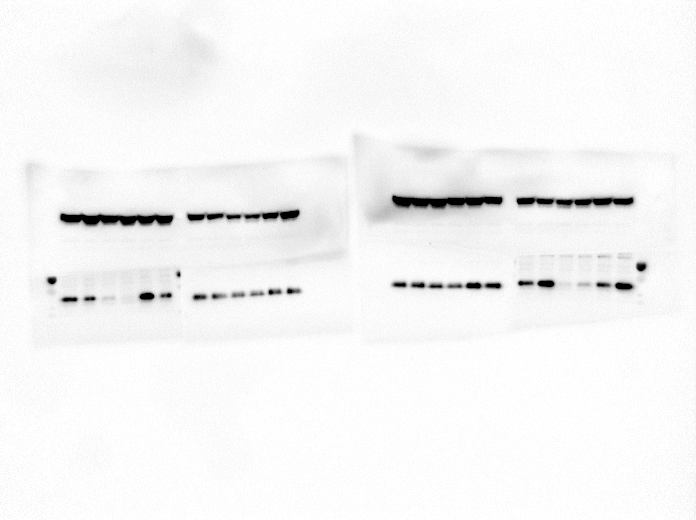

Supplement: Supplementary file 11 — Source Data for Figure 3 [file EMBR-24-e57264-s001.zip › EMBOR-2023-57264V1_SourceDataForFigure3A-B_H-L/I/221113_blot4/Blot4_P-cofilin_TotalCofilin_tubulin_quantification.tif]

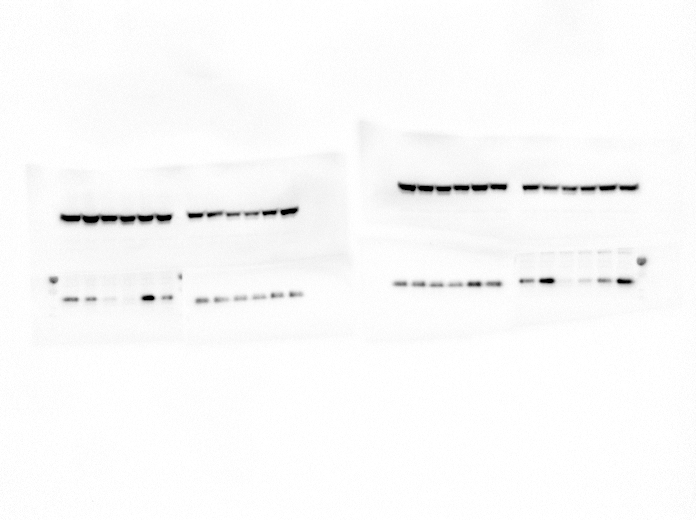

Supplement: Supplementary file 11 — Source Data for Figure 3 [file EMBR-24-e57264-s001.zip › EMBOR-2023-57264V1_SourceDataForFigure3A-B_H-L/I/221113_blot4/Blot4_tubulin_quantification.tif]

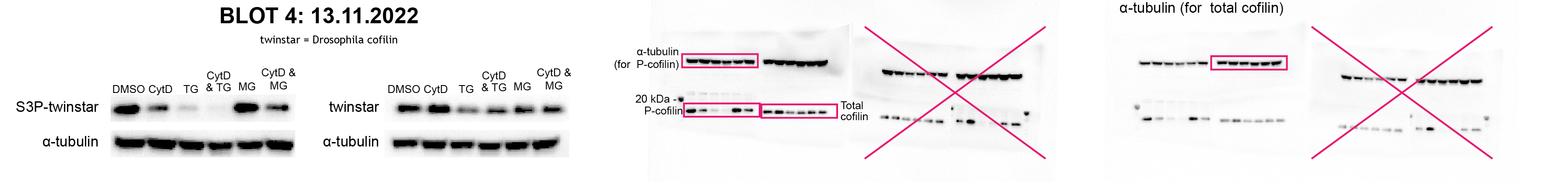

Supplement: Supplementary file 11 — Source Data for Figure 3 [file EMBR-24-e57264-s001.zip › EMBOR-2023-57264V1_SourceDataForFigure3A-B_H-L/I/221113_blot4/Blot4_Annotated.png]

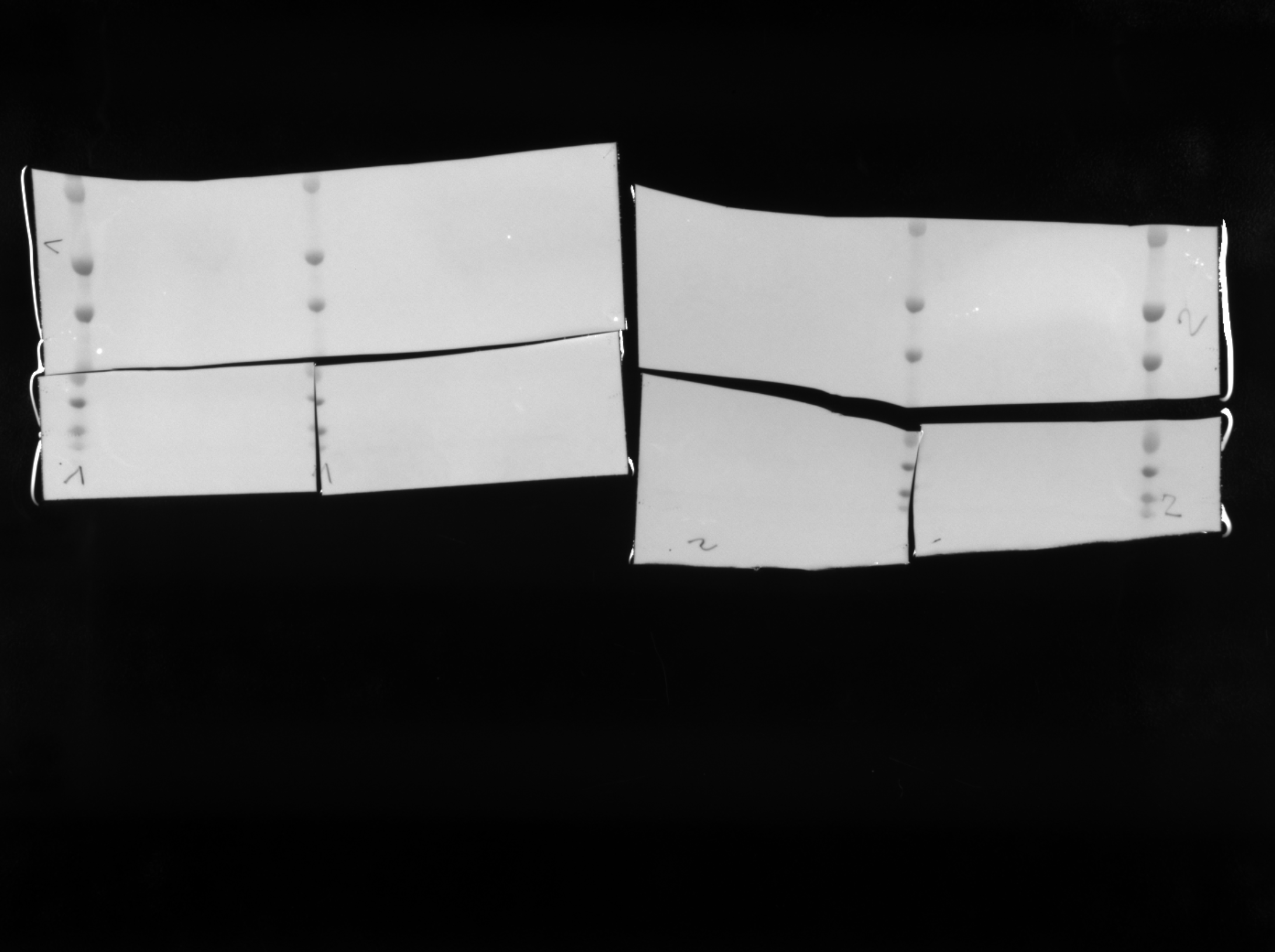

Supplement: Supplementary file 11 — Source Data for Figure 3 [file EMBR-24-e57264-s001.zip › EMBOR-2023-57264V1_SourceDataForFigure3A-B_H-L/I/221113_blot4/Blot4_coliometric.tif]

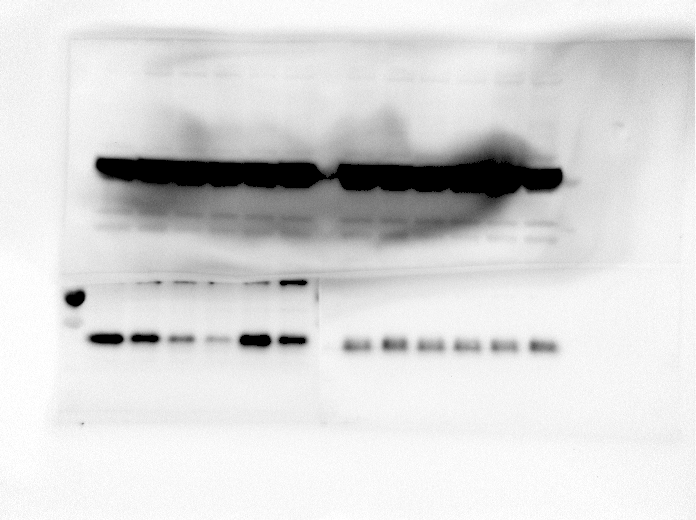

Supplement: Supplementary file 11 — Source Data for Figure 3 [file EMBR-24-e57264-s001.zip › EMBOR-2023-57264V1_SourceDataForFigure3A-B_H-L/I/221017_blot2/Blot2_TotalCofilinRight_P-cofilinLeft_quantification.tif]

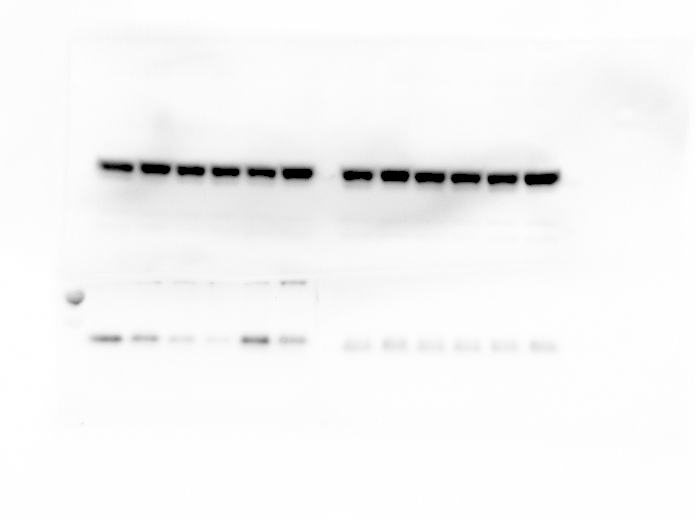

Supplement: Supplementary file 11 — Source Data for Figure 3 [file EMBR-24-e57264-s001.zip › EMBOR-2023-57264V1_SourceDataForFigure3A-B_H-L/I/221017_blot2/Blot2_tubulin_quantification.tif]

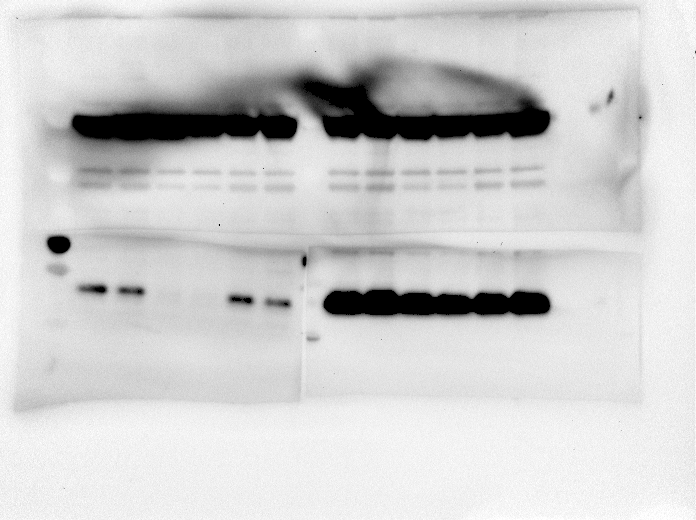

Supplement: Supplementary file 11 — Source Data for Figure 3 [file EMBR-24-e57264-s001.zip › EMBOR-2023-57264V1_SourceDataForFigure3A-B_H-L/I/221020_blot3/Blot3_P-cofilin_quantification.tif]

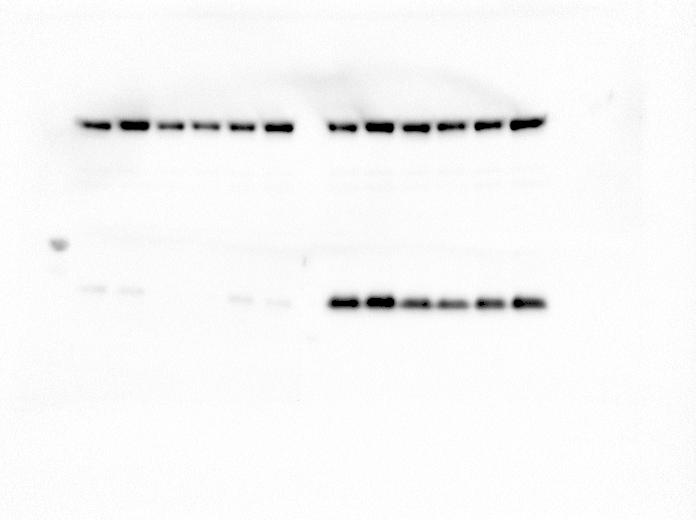

Supplement: Supplementary file 11 — Source Data for Figure 3 [file EMBR-24-e57264-s001.zip › EMBOR-2023-57264V1_SourceDataForFigure3A-B_H-L/I/221020_blot3/Blot3_tubulinTop_TotalCofilinBottom_quantification.tif]

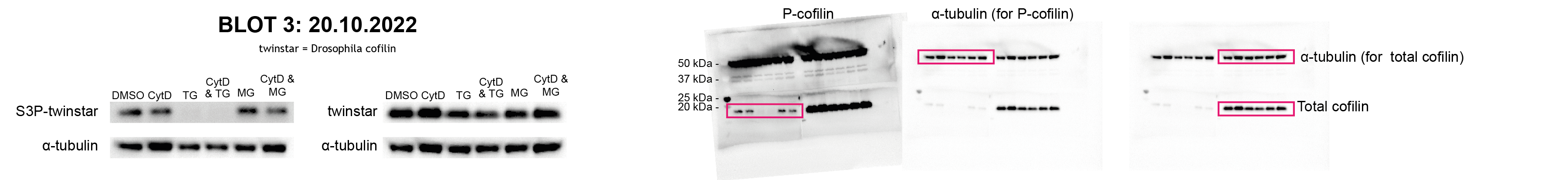

Supplement: Supplementary file 11 — Source Data for Figure 3 [file EMBR-24-e57264-s001.zip › EMBOR-2023-57264V1_SourceDataForFigure3A-B_H-L/I/221020_blot3/Blot3_Annotated_Western_221020.png]

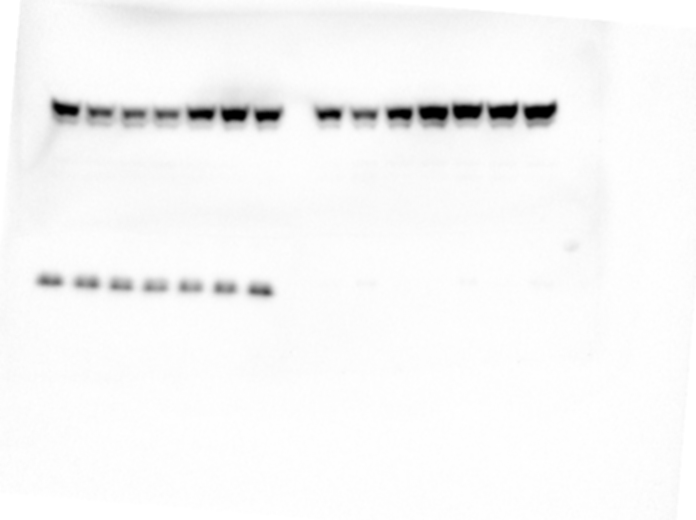

Supplement: Supplementary file 11 — Source Data for Figure 3 [file EMBR-24-e57264-s001.zip › EMBOR-2023-57264V1_SourceDataForFigure3A-B_H-L/I/221014_blot1/Blot1_tubulinTop_quantification.tif]

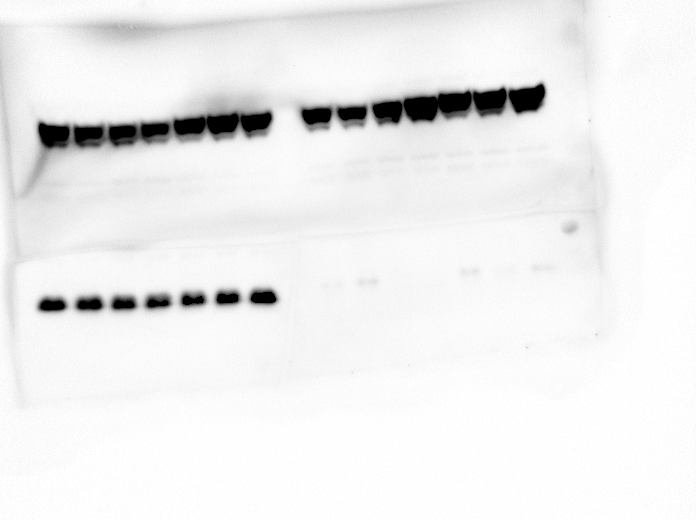

Supplement: Supplementary file 11 — Source Data for Figure 3 [file EMBR-24-e57264-s001.zip › EMBOR-2023-57264V1_SourceDataForFigure3A-B_H-L/I/221014_blot1/Blot1_TotalCofilin_quantification.tif]

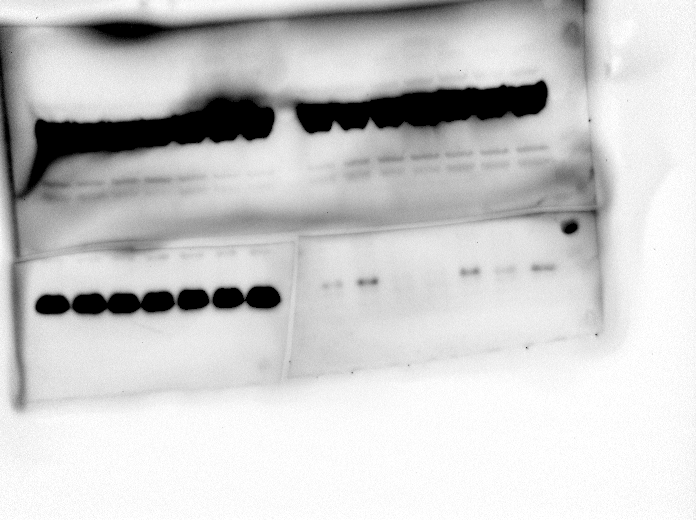

Supplement: Supplementary file 11 — Source Data for Figure 3 [file EMBR-24-e57264-s001.zip › EMBOR-2023-57264V1_SourceDataForFigure3A-B_H-L/I/221014_blot1/Blot1_P-cofilin_quantification.tif]

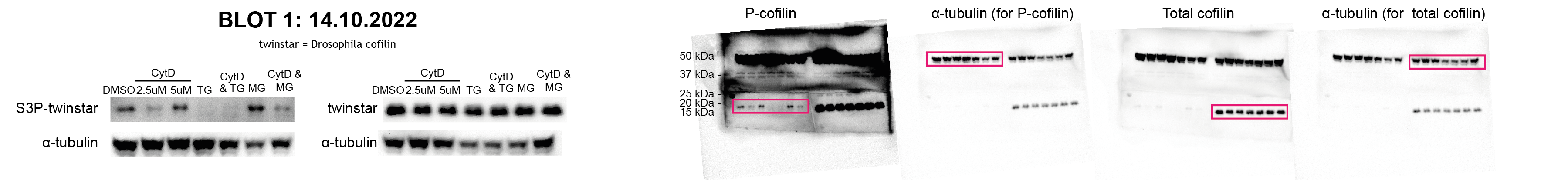

Supplement: Supplementary file 11 — Source Data for Figure 3 [file EMBR-24-e57264-s001.zip › EMBOR-2023-57264V1_SourceDataForFigure3A-B_H-L/I/221014_blot1/Blot1_Annotated_Western_221014.png]

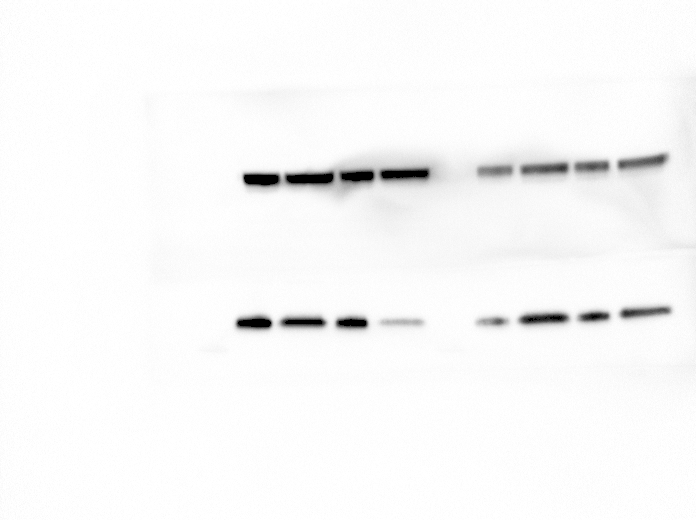

Supplement: Supplementary file 11 — Source Data for Figure 3 [file EMBR-24-e57264-s001.zip › EMBOR-2023-57264V1_SourceDataForFigure3A-B_H-L/K/Dataset11_D1_221124_Acq_230202/Dataset11_cofilin_tubulin_quantification_lanes3_4.tif]

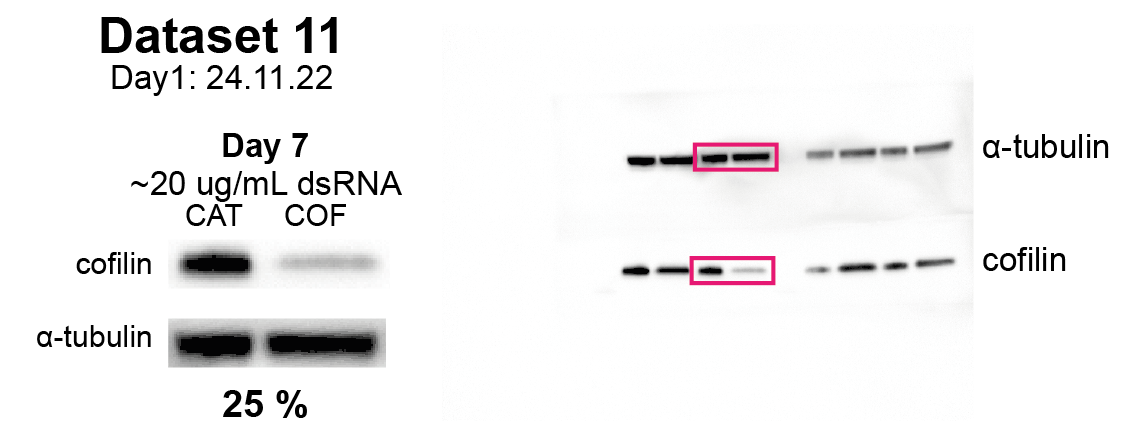

Supplement: Supplementary file 11 — Source Data for Figure 3 [file EMBR-24-e57264-s001.zip › EMBOR-2023-57264V1_SourceDataForFigure3A-B_H-L/K/Dataset11_D1_221124_Acq_230202/Dataset11_Annotated_Western.png.png]

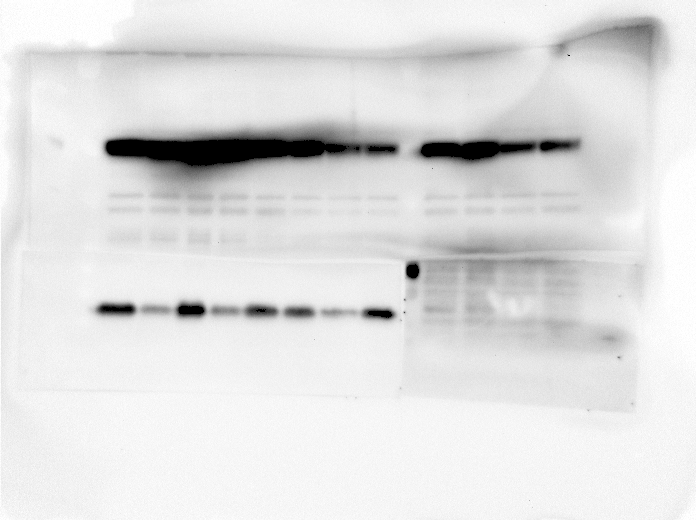

Supplement: Supplementary file 11 — Source Data for Figure 3 [file EMBR-24-e57264-s001.zip › EMBOR-2023-57264V1_SourceDataForFigure3A-B_H-L/K/Dataset9_D1_221201_western_221210_Acq_221208/Dataset9_Cofilin_quantification_Lanes1_2.tif]

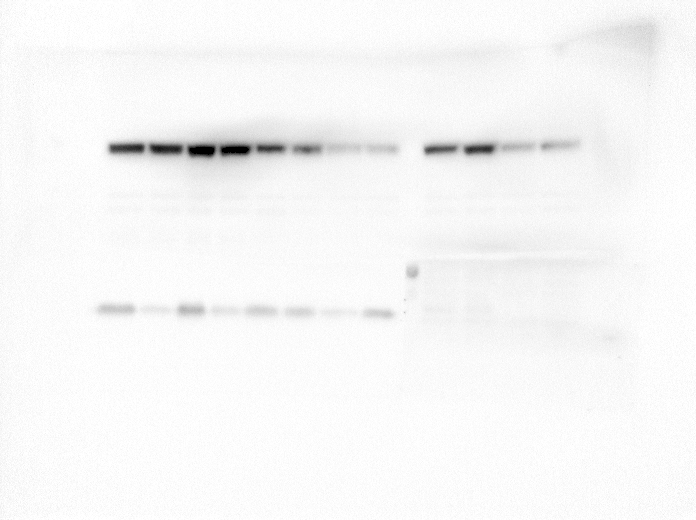

Supplement: Supplementary file 11 — Source Data for Figure 3 [file EMBR-24-e57264-s001.zip › EMBOR-2023-57264V1_SourceDataForFigure3A-B_H-L/K/Dataset9_D1_221201_western_221210_Acq_221208/Dataset9_Tubulin_quantification_Lanes1_2.tif]

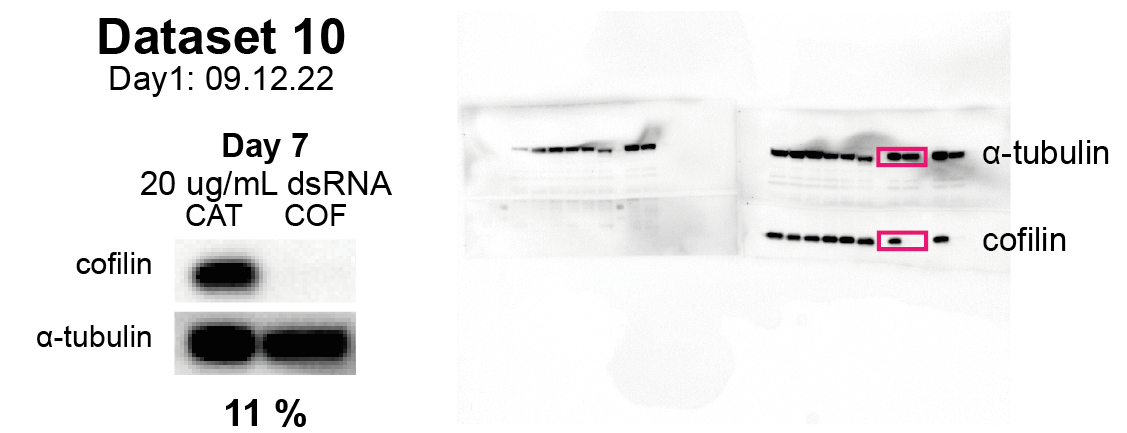

Supplement: Supplementary file 11 — Source Data for Figure 3 [file EMBR-24-e57264-s001.zip › EMBOR-2023-57264V1_SourceDataForFigure3A-B_H-L/K/Dataset10_D1_221209_western_Acq_221216/Dataset10_Annotated_Western.png.png]

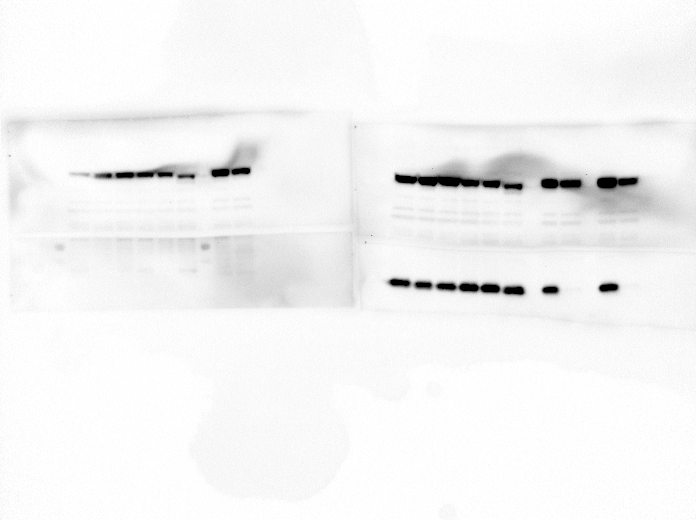

Supplement: Supplementary file 11 — Source Data for Figure 3 [file EMBR-24-e57264-s001.zip › EMBOR-2023-57264V1_SourceDataForFigure3A-B_H-L/K/Dataset10_D1_221209_western_Acq_221216/Dataset10_cofilin_tubulin_quantification_lanes8_9.tif]

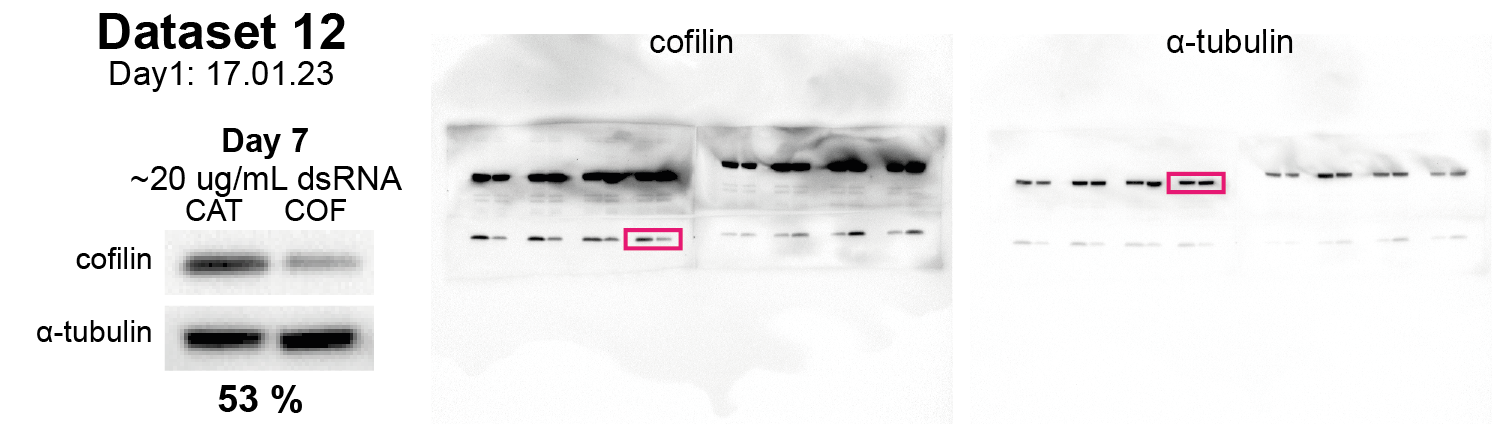

Supplement: Supplementary file 11 — Source Data for Figure 3 [file EMBR-24-e57264-s001.zip › EMBOR-2023-57264V1_SourceDataForFigure3A-B_H-L/K/Dataset12_D1_230117_Acq_230203/Dataset12_Annotated_Western.png.png]

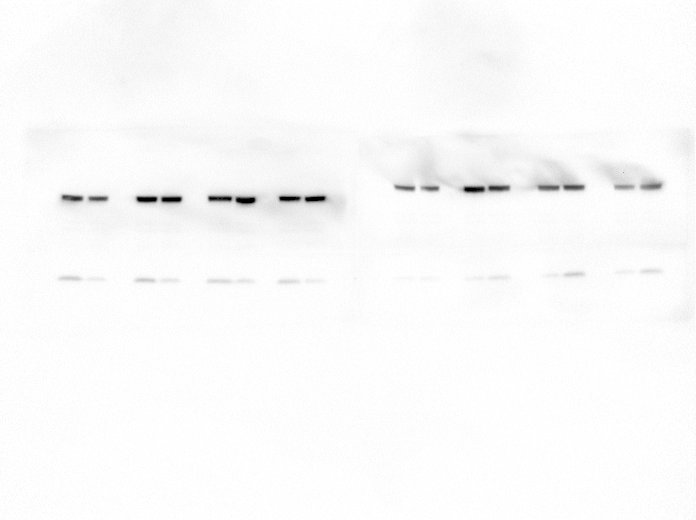

Supplement: Supplementary file 11 — Source Data for Figure 3 [file EMBR-24-e57264-s001.zip › EMBOR-2023-57264V1_SourceDataForFigure3A-B_H-L/K/Dataset12_D1_230117_Acq_230203/Dataset12_tubulin_quantification_lanes7_8_gel1.tif]

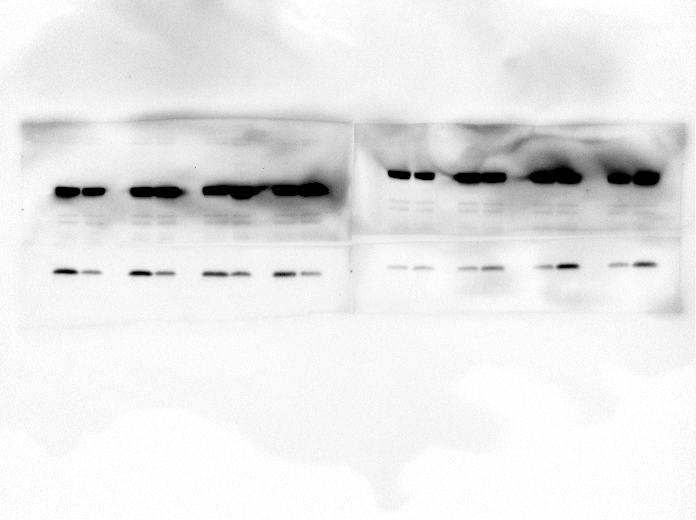

Supplement: Supplementary file 11 — Source Data for Figure 3 [file EMBR-24-e57264-s001.zip › EMBOR-2023-57264V1_SourceDataForFigure3A-B_H-L/K/Dataset12_D1_230117_Acq_230203/Dataset12_cofilin_quantification_lanes7_8_gel1.tif.tif]
