# Supplementary figures and images for: CryoET shows cofilactin filaments inside the microtubule lumen (part 1 of 2)
Source: EMBO Rep. 2023 Sep 13;24(11):e57264. doi: 10.15252/embr.202357264 (PMC10626427; doi:10.15252/embr.202357264)

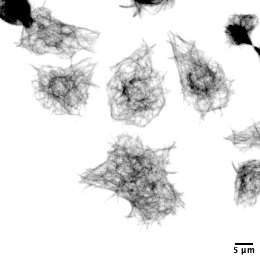

Supplement: Supplementary file 7 — Source Data for Expanded View and Appendix [file EMBR-24-e57264-s003.zip › EMBOR-2023-57264V1_SourceDataForExpandedViewAndAppendix/Figure_EV1/A/SUM_02-DMSO_022-1.png]

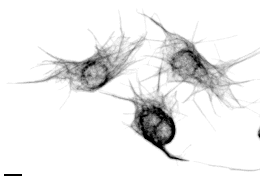

Supplement: Supplementary file 7 — Source Data for Expanded View and Appendix [file EMBR-24-e57264-s003.zip › EMBOR-2023-57264V1_SourceDataForExpandedViewAndAppendix/Figure_EV1/A/SUM_04-CytD_013-2-1_5umScale.png]

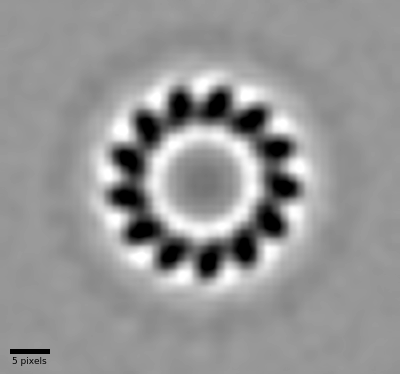

Supplement: Supplementary file 7 — Source Data for Expanded View and Appendix [file EMBR-24-e57264-s003.zip › EMBOR-2023-57264V1_SourceDataForExpandedViewAndAppendix/Figure_EV1/F/ref_13PF_minus_11.808Apx.png]

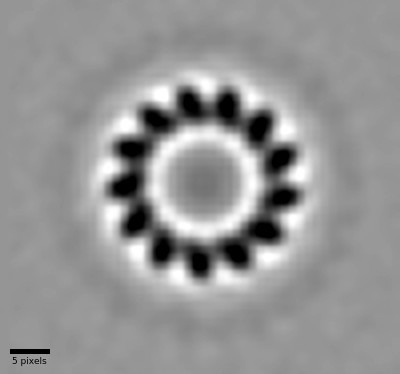

Supplement: Supplementary file 7 — Source Data for Expanded View and Appendix [file EMBR-24-e57264-s003.zip › EMBOR-2023-57264V1_SourceDataForExpandedViewAndAppendix/Figure_EV1/F/ref_13PF_plus_11.808Apx.png]

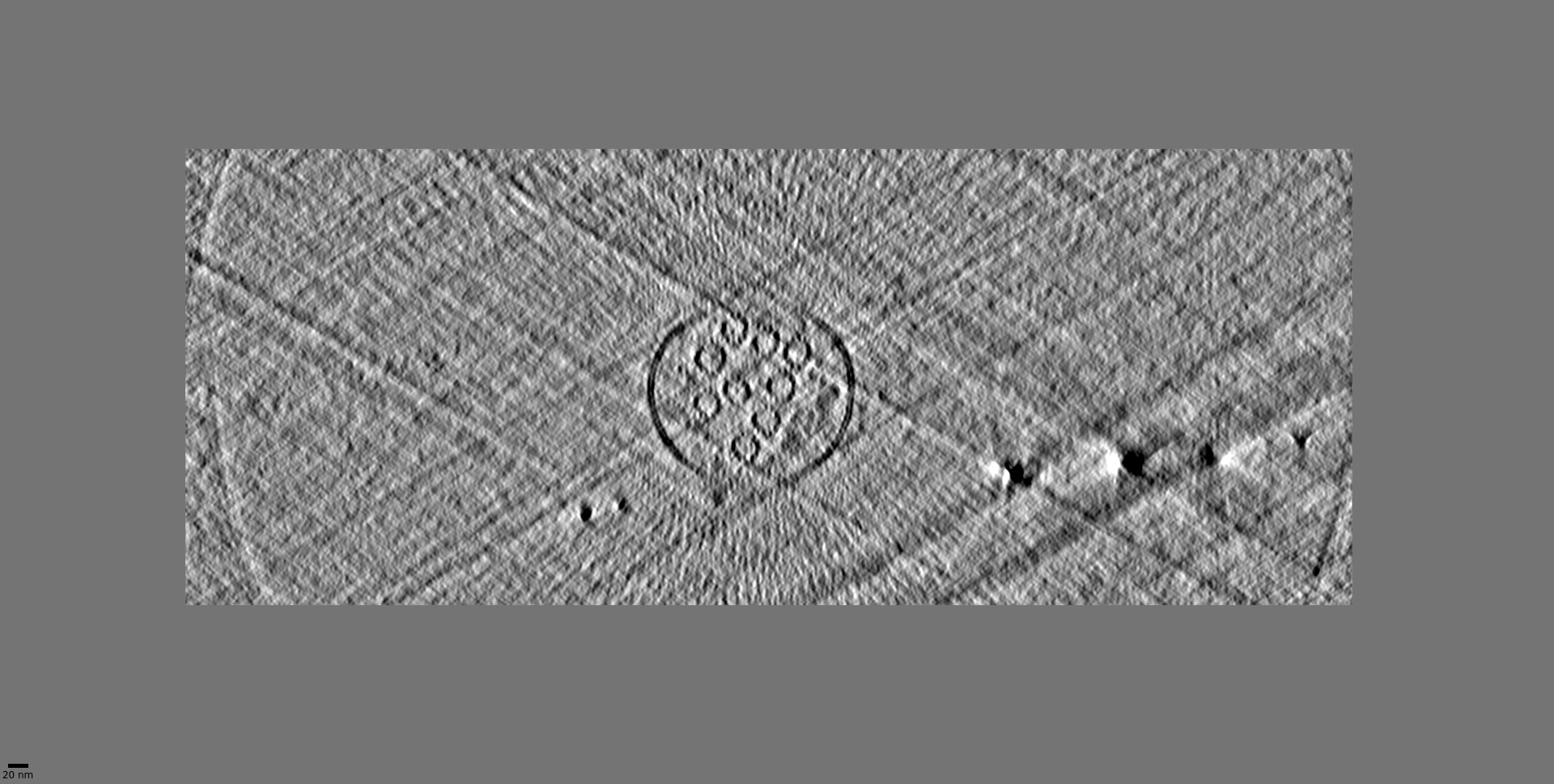

Supplement: Supplementary file 7 — Source Data for Expanded View and Appendix [file EMBR-24-e57264-s003.zip › EMBOR-2023-57264V1_SourceDataForExpandedViewAndAppendix/Figure_EV1/C/Fig_EV1C_DZ1_TS_069_cross_thick20_scale.png]

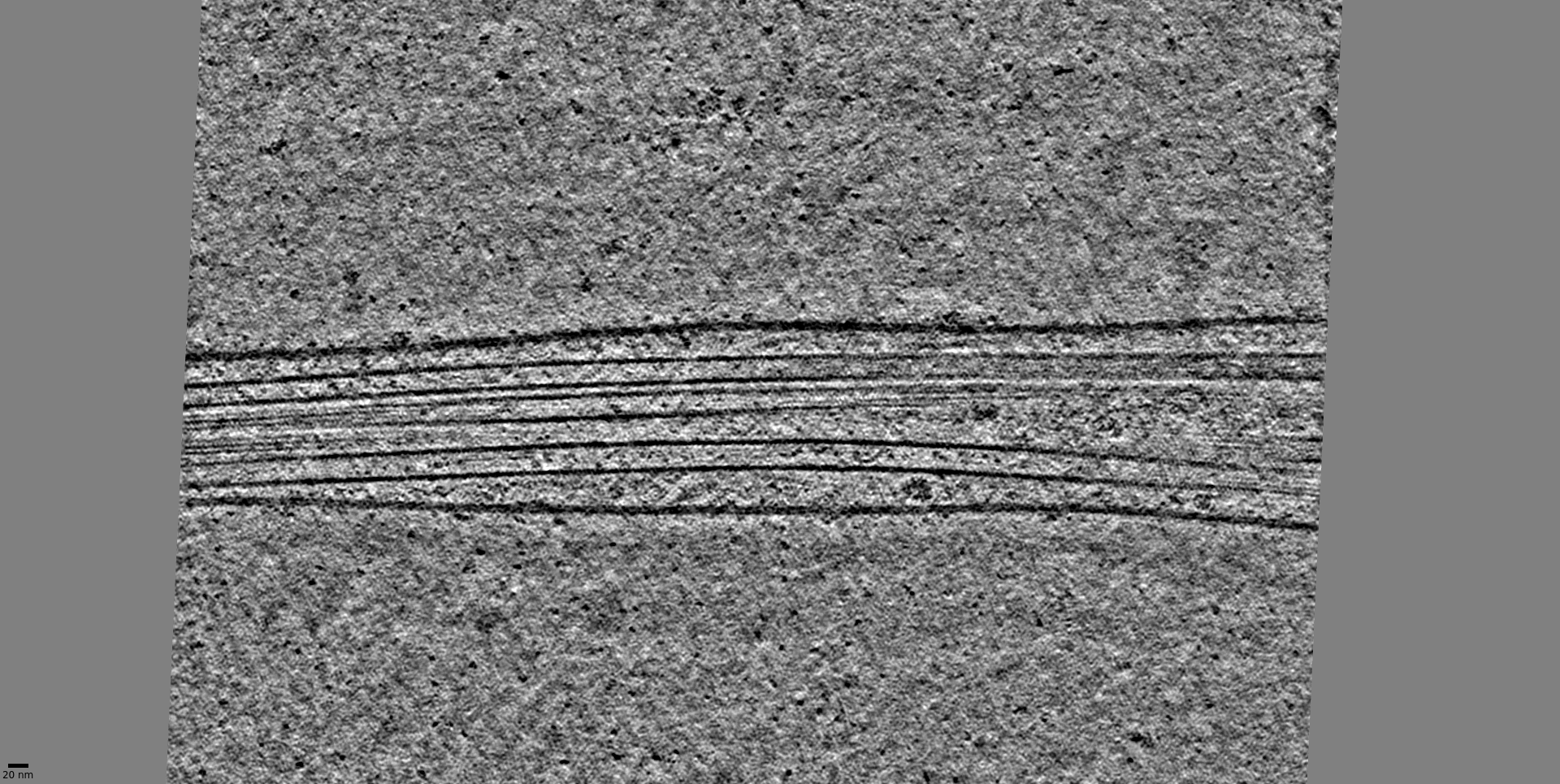

Supplement: Supplementary file 7 — Source Data for Expanded View and Appendix [file EMBR-24-e57264-s003.zip › EMBOR-2023-57264V1_SourceDataForExpandedViewAndAppendix/Figure_EV1/C/Fig_EV1C_DZ1_TS_069_side_thick20_scale.png]

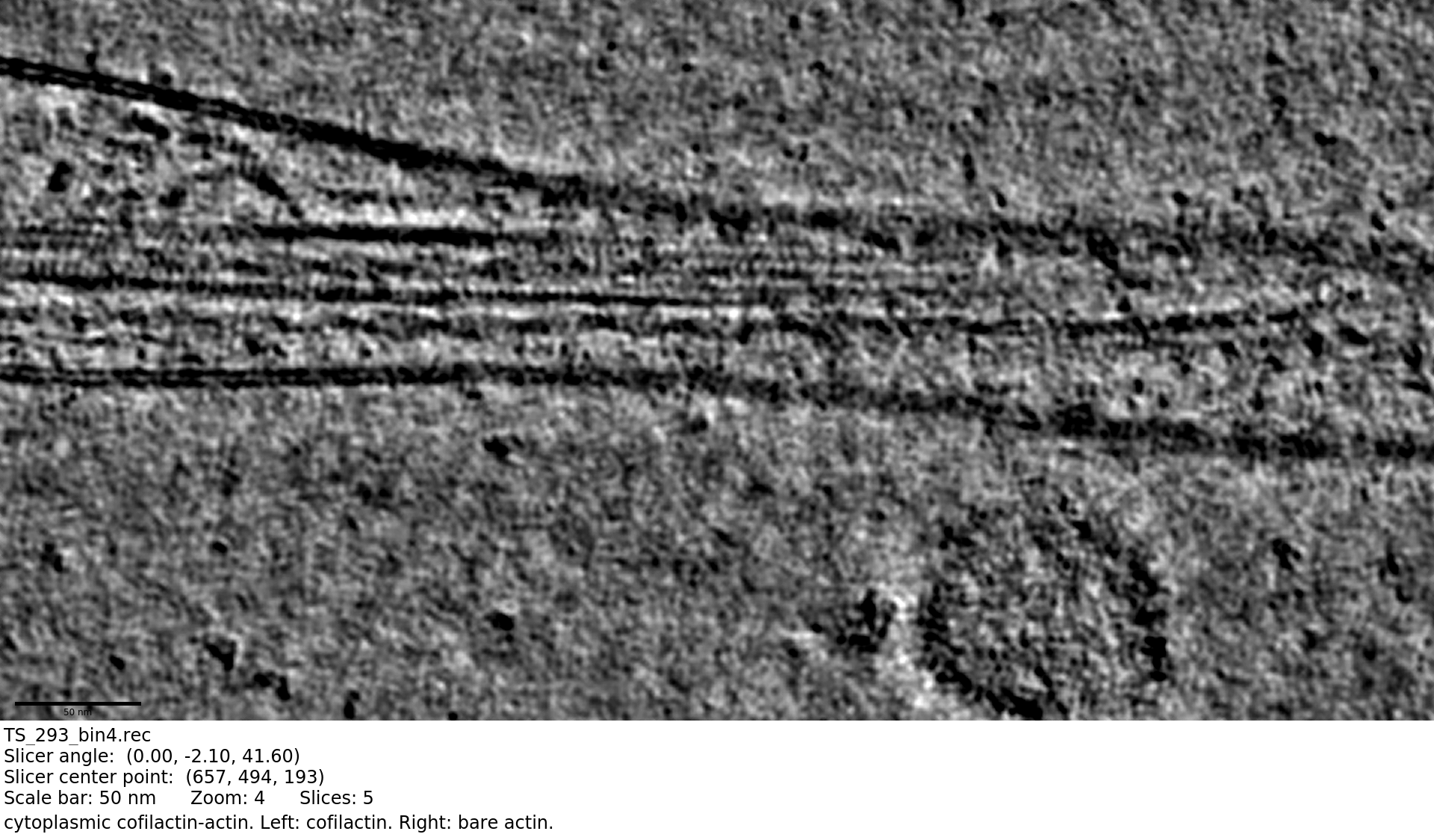

Supplement: Supplementary file 7 — Source Data for Expanded View and Appendix [file EMBR-24-e57264-s003.zip › EMBOR-2023-57264V1_SourceDataForExpandedViewAndAppendix/Figure_EV3/I/FigEV3I_CytD&TG_top_dataset7_220721_TS_293.png]

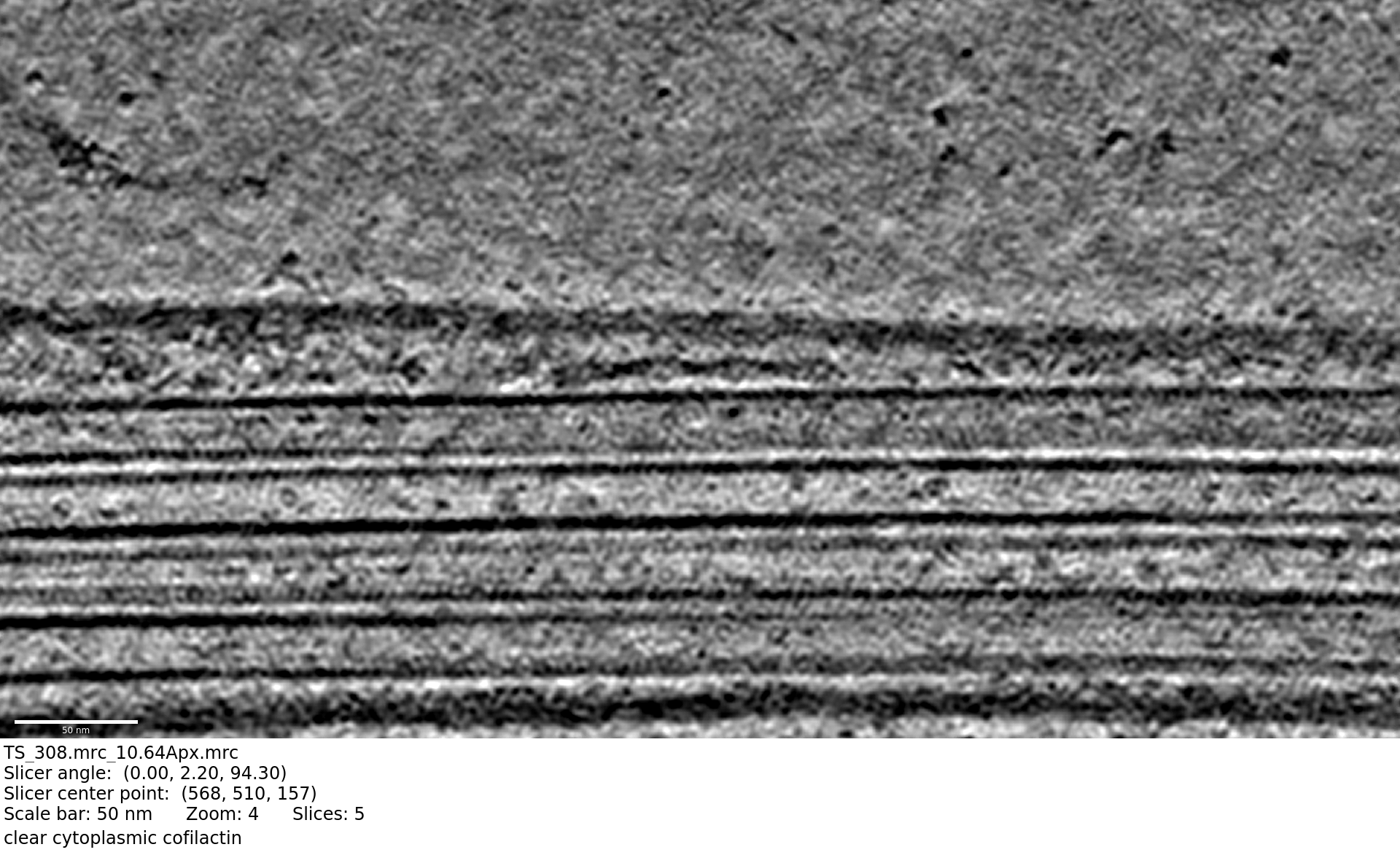

Supplement: Supplementary file 7 — Source Data for Expanded View and Appendix [file EMBR-24-e57264-s003.zip › EMBOR-2023-57264V1_SourceDataForExpandedViewAndAppendix/Figure_EV3/I/FigEV3I_CytD&DMSO_bottom_dataset7_220721_TS_308.png]

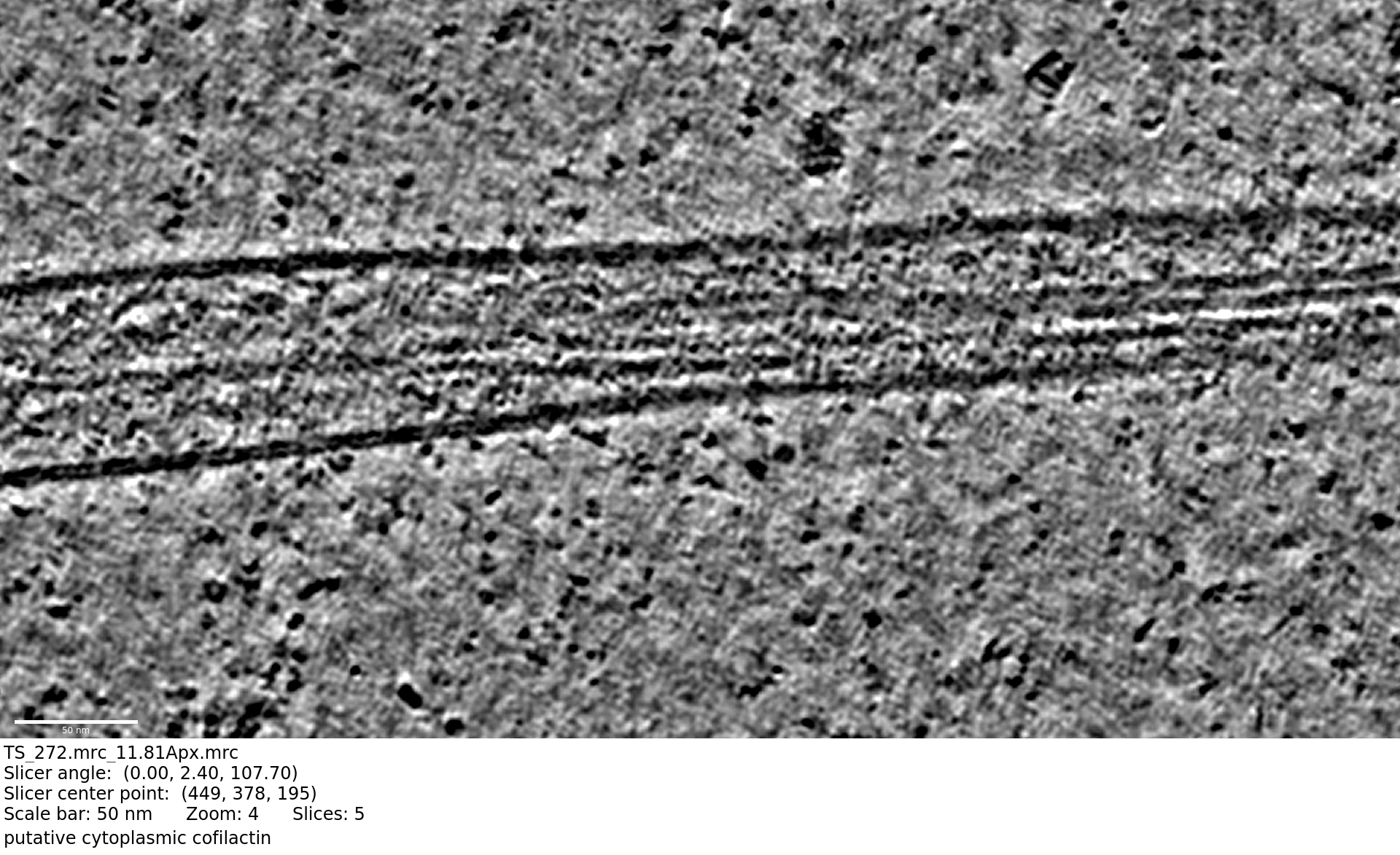

Supplement: Supplementary file 7 — Source Data for Expanded View and Appendix [file EMBR-24-e57264-s003.zip › EMBOR-2023-57264V1_SourceDataForExpandedViewAndAppendix/Figure_EV3/I/FigEV3I_CytD&DMSO_top_dataset6_220720_TS_272.png]

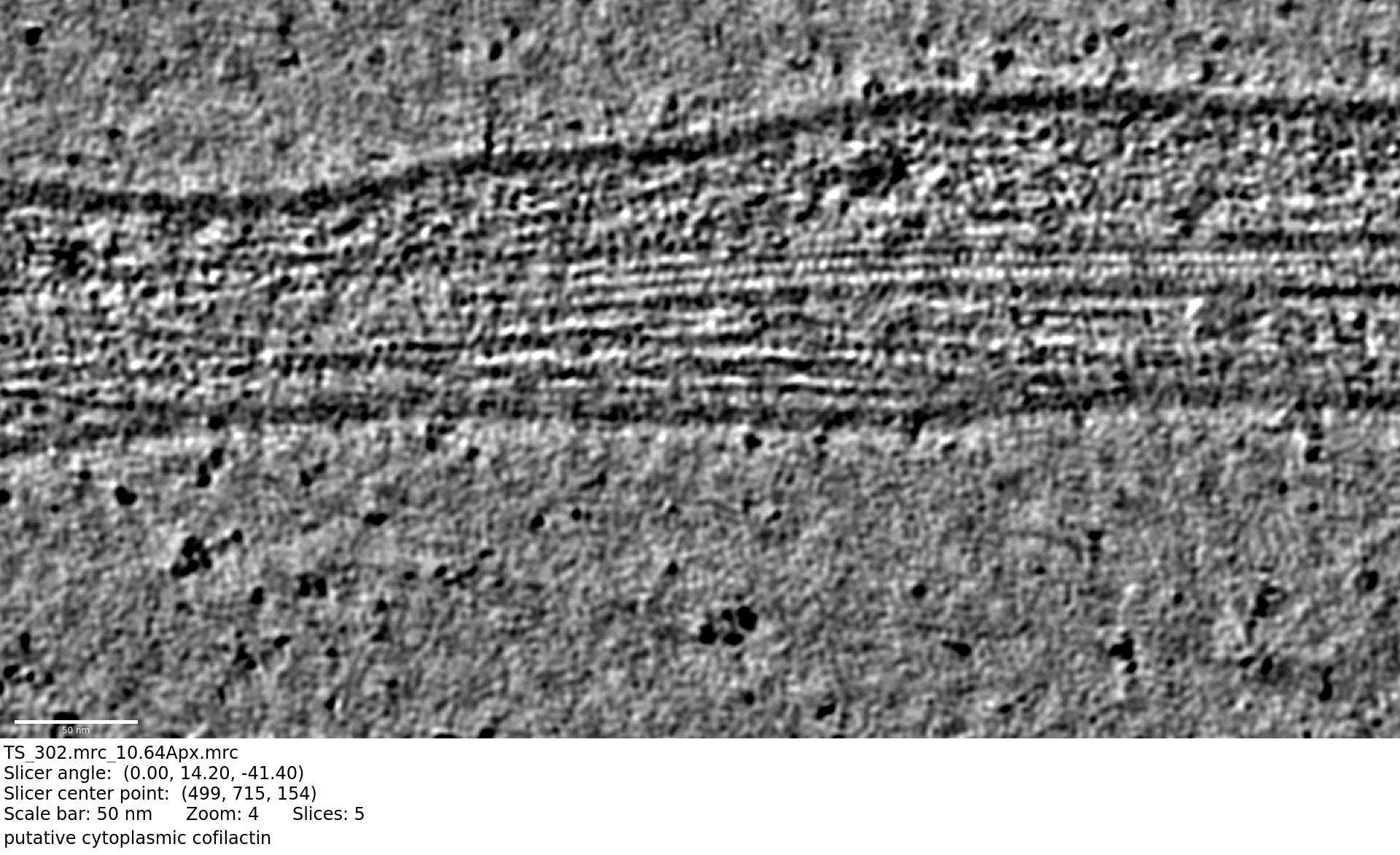

Supplement: Supplementary file 7 — Source Data for Expanded View and Appendix [file EMBR-24-e57264-s003.zip › EMBOR-2023-57264V1_SourceDataForExpandedViewAndAppendix/Figure_EV3/I/FigEV3I_CytD&TG_bottom_dataset7_220721_TS_302.png]

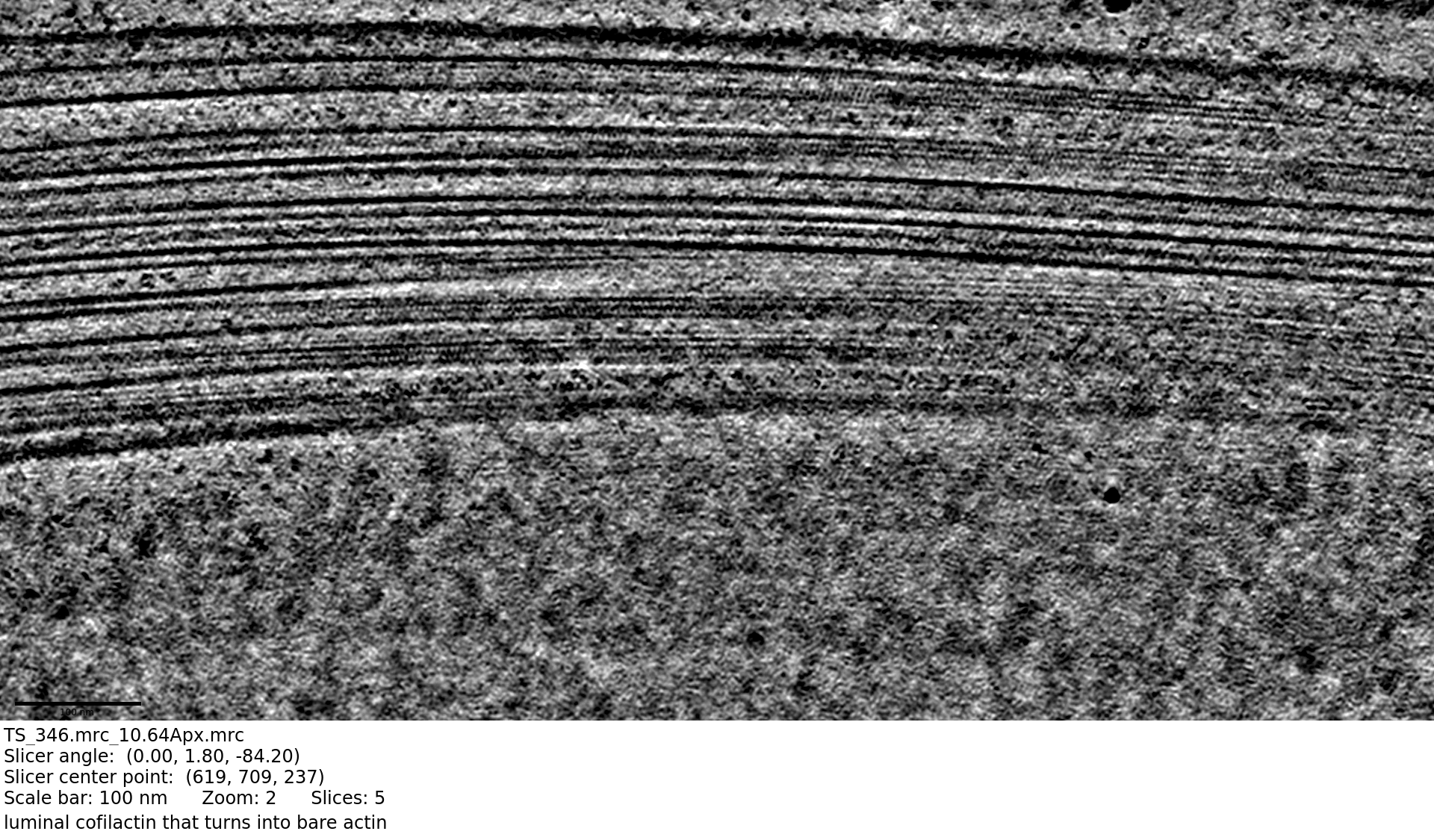

Supplement: Supplementary file 7 — Source Data for Expanded View and Appendix [file EMBR-24-e57264-s003.zip › EMBOR-2023-57264V1_SourceDataForExpandedViewAndAppendix/Figure_EV3/N/FigEV3N_TS_346_LumFilAlternativeMorphology_3rd.png]

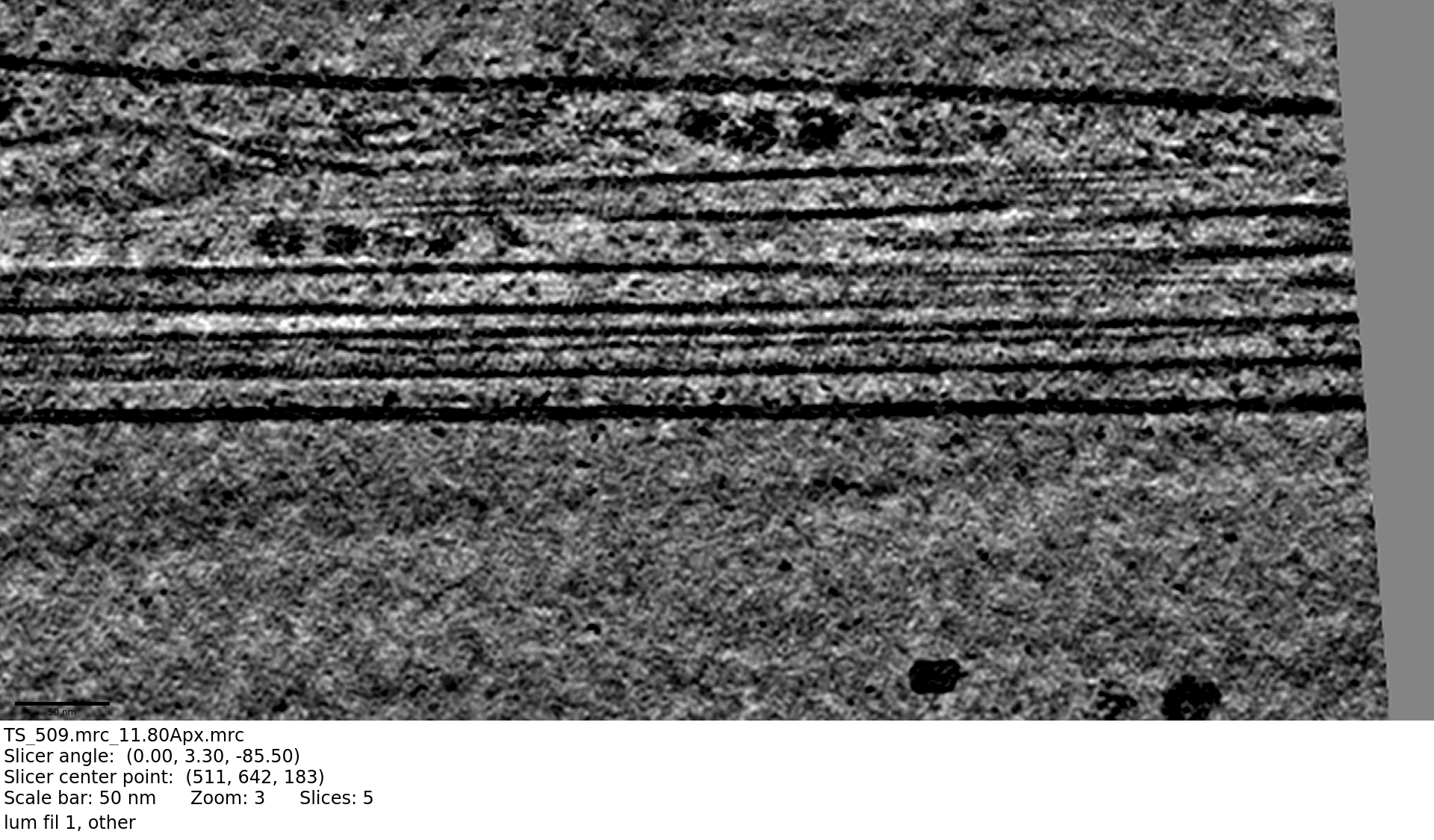

Supplement: Supplementary file 7 — Source Data for Expanded View and Appendix [file EMBR-24-e57264-s003.zip › EMBOR-2023-57264V1_SourceDataForExpandedViewAndAppendix/Figure_EV3/N/FigEV3N_TS_509_LumFilAlternativeMorphology_4th.png]

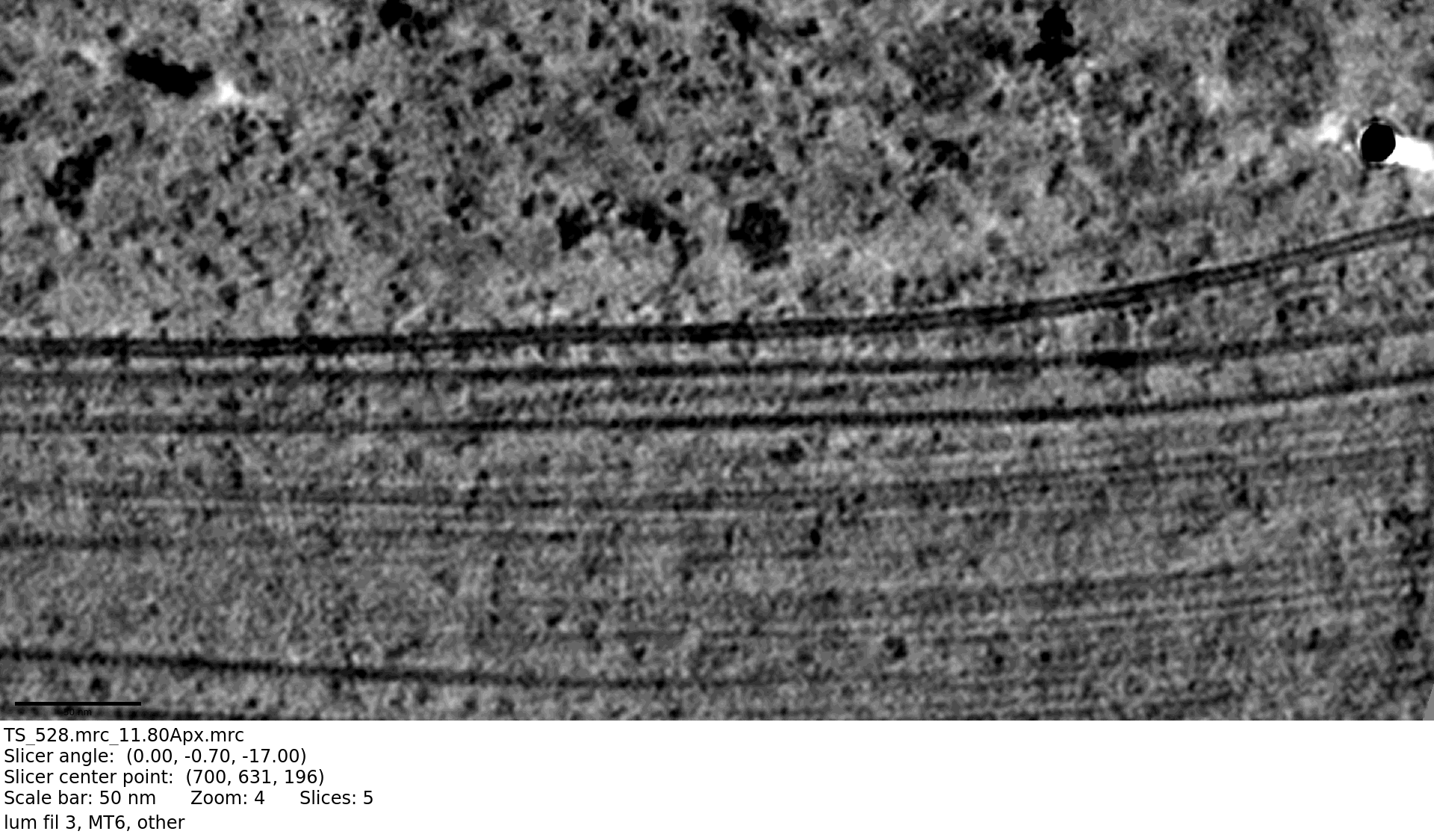

Supplement: Supplementary file 7 — Source Data for Expanded View and Appendix [file EMBR-24-e57264-s003.zip › EMBOR-2023-57264V1_SourceDataForExpandedViewAndAppendix/Figure_EV3/N/FigEV3N_TS_528_LumFilAlternativeMorphology_2nd.png]

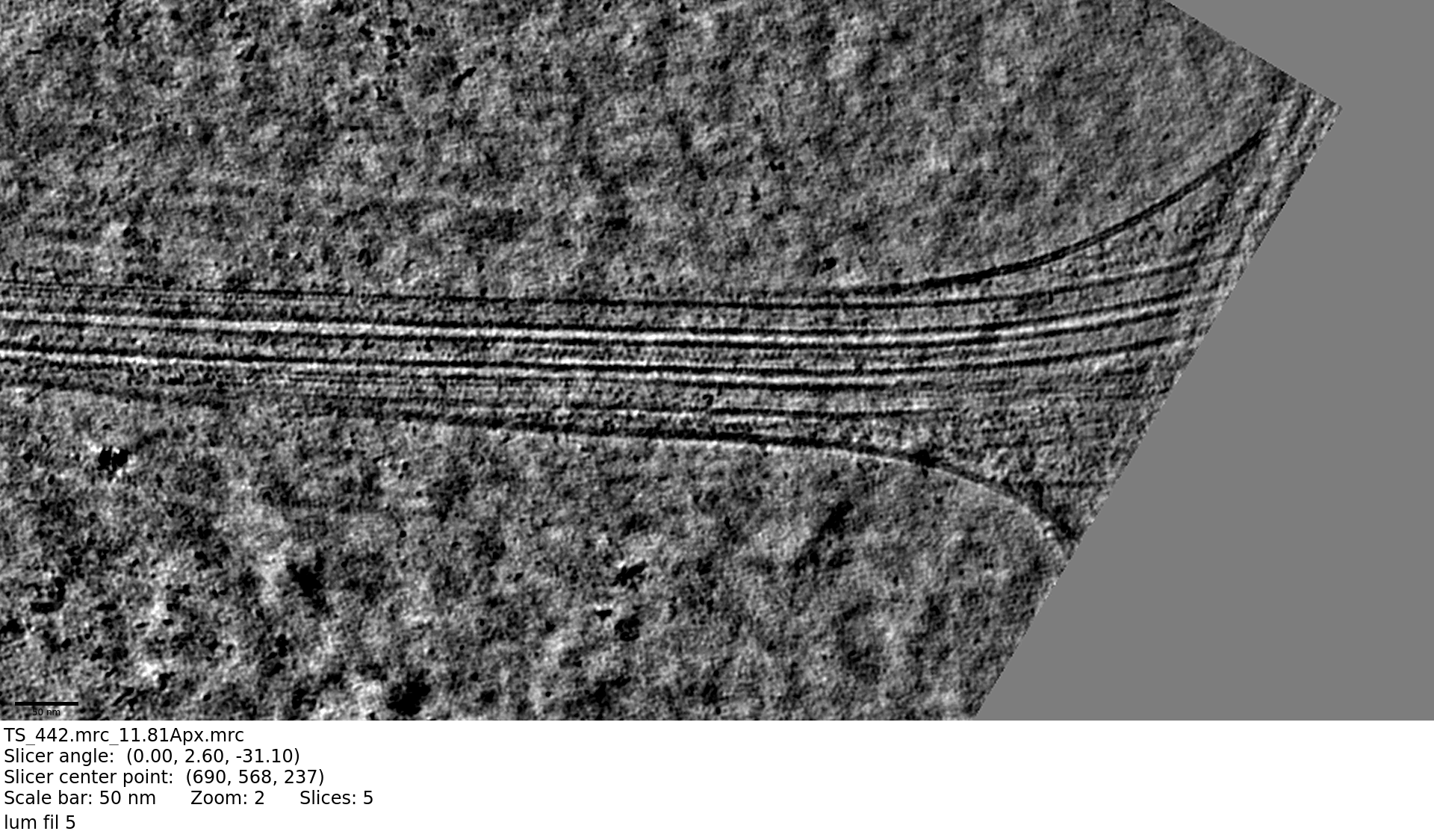

Supplement: Supplementary file 7 — Source Data for Expanded View and Appendix [file EMBR-24-e57264-s003.zip › EMBOR-2023-57264V1_SourceDataForExpandedViewAndAppendix/Figure_EV3/N/FigEV3N_TS_442_LumFilAlternativeMorphology_1st.png]

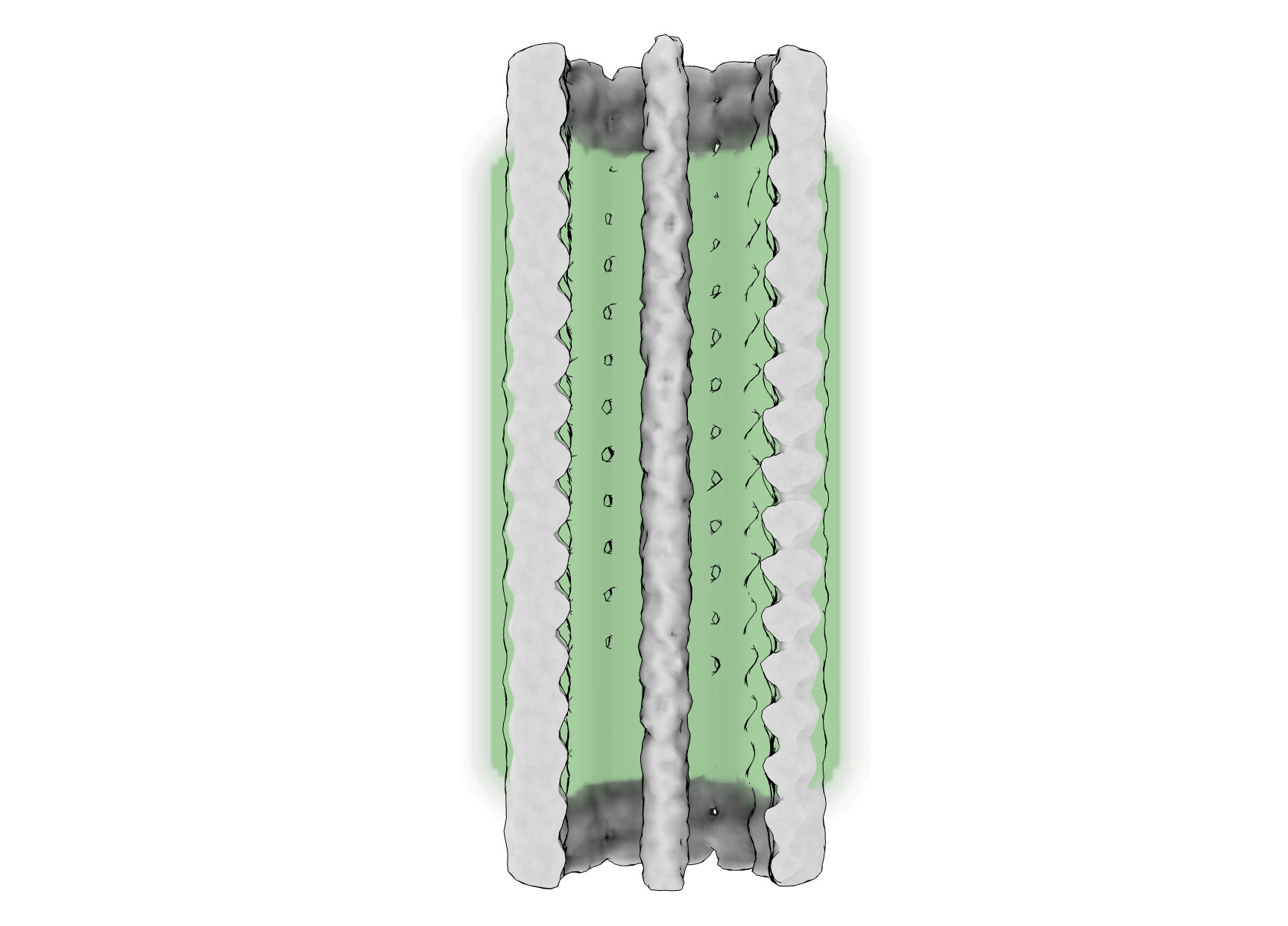

Supplement: Supplementary file 7 — Source Data for Expanded View and Appendix [file EMBR-24-e57264-s003.zip › EMBOR-2023-57264V1_SourceDataForExpandedViewAndAppendix/Figure_EV3/G/FigEV3G_BottomPanel_Left.png]

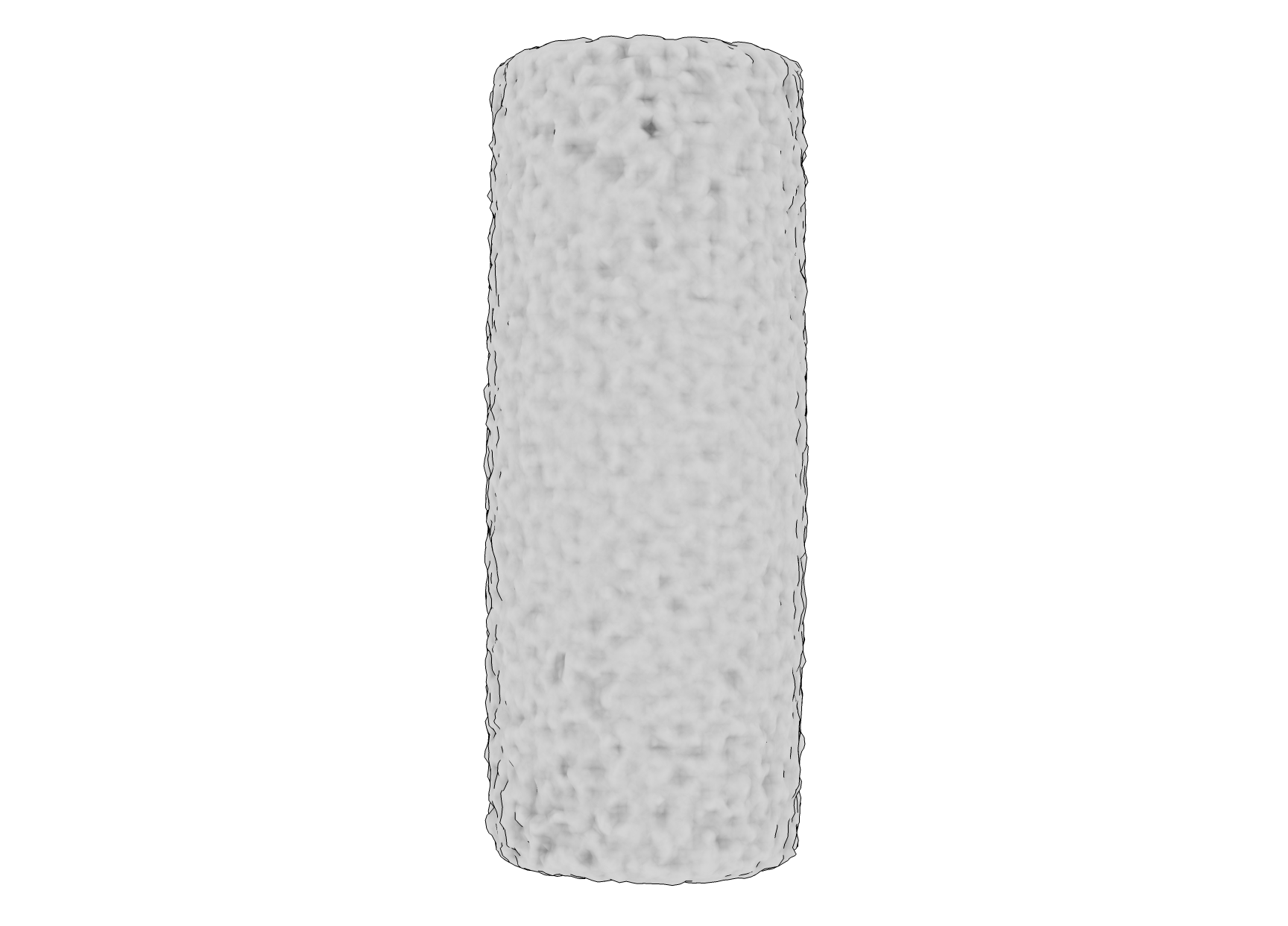

Supplement: Supplementary file 7 — Source Data for Expanded View and Appendix [file EMBR-24-e57264-s003.zip › EMBOR-2023-57264V1_SourceDataForExpandedViewAndAppendix/Figure_EV3/G/FigEV3G_TopPanel_Right.png]

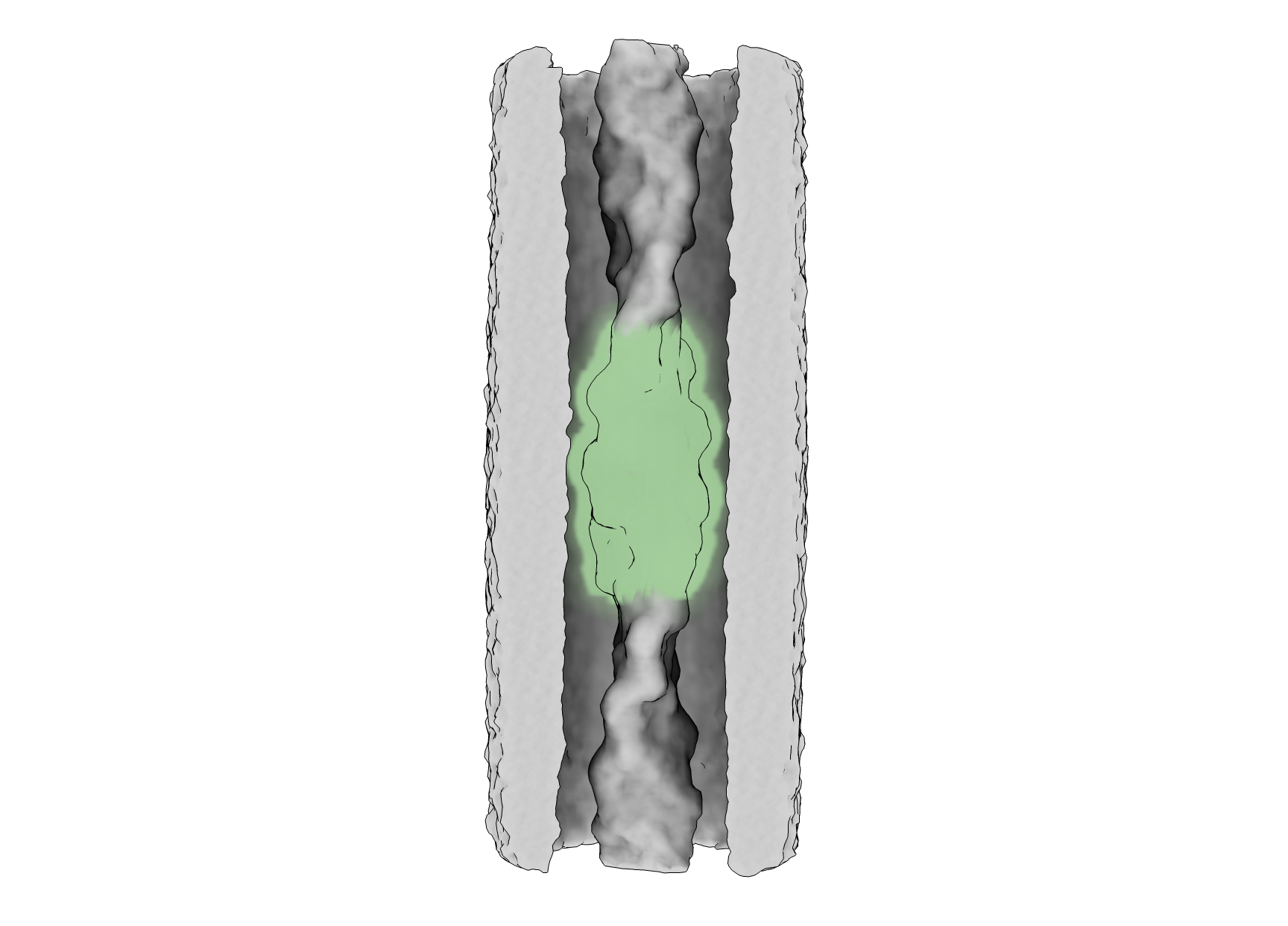

Supplement: Supplementary file 7 — Source Data for Expanded View and Appendix [file EMBR-24-e57264-s003.zip › EMBOR-2023-57264V1_SourceDataForExpandedViewAndAppendix/Figure_EV3/G/FigEV3G_TopPanel_Left.png]

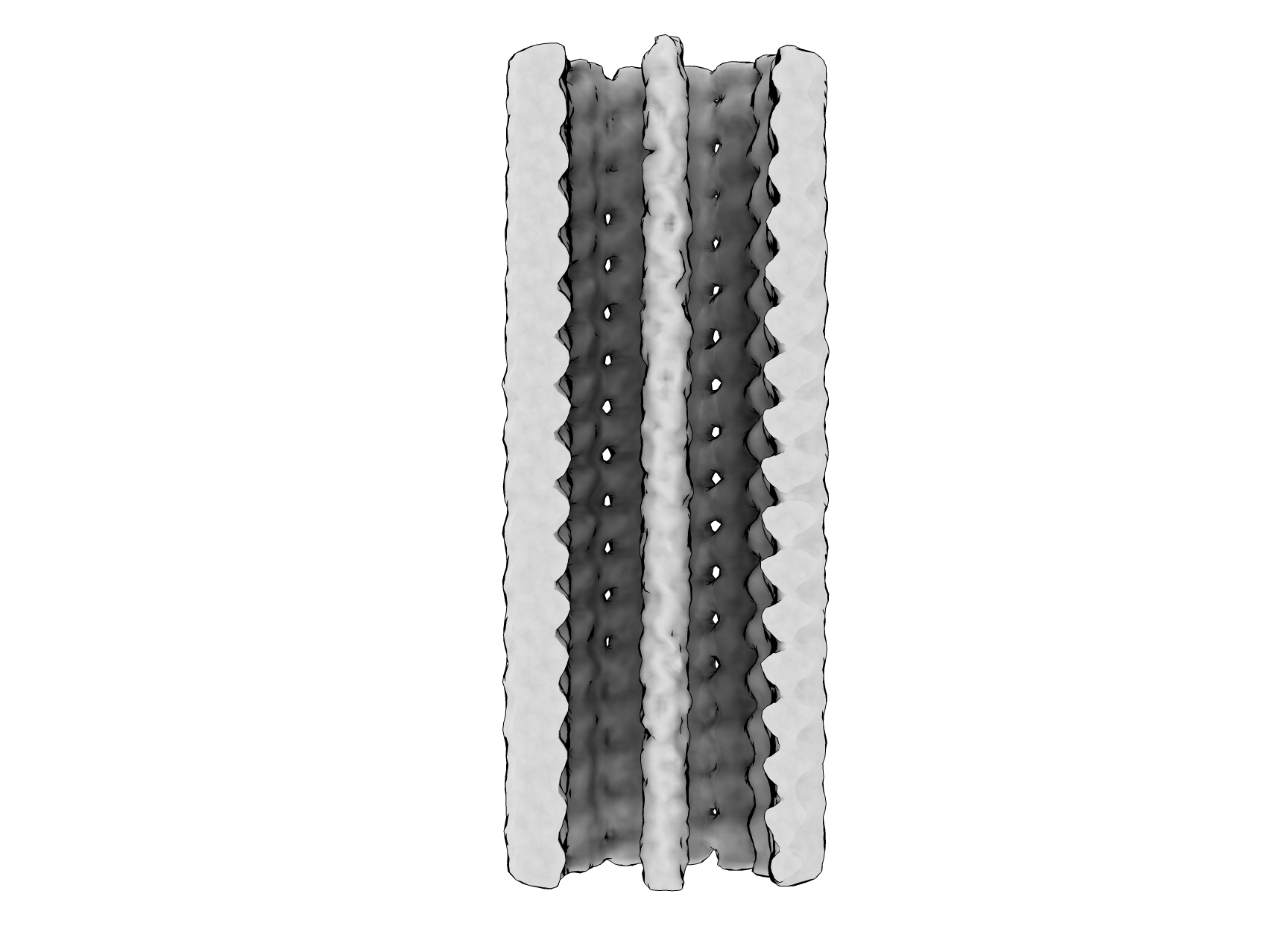

Supplement: Supplementary file 7 — Source Data for Expanded View and Appendix [file EMBR-24-e57264-s003.zip › EMBOR-2023-57264V1_SourceDataForExpandedViewAndAppendix/Figure_EV3/G/FigEV3G_BottomPanel_Middle.png]

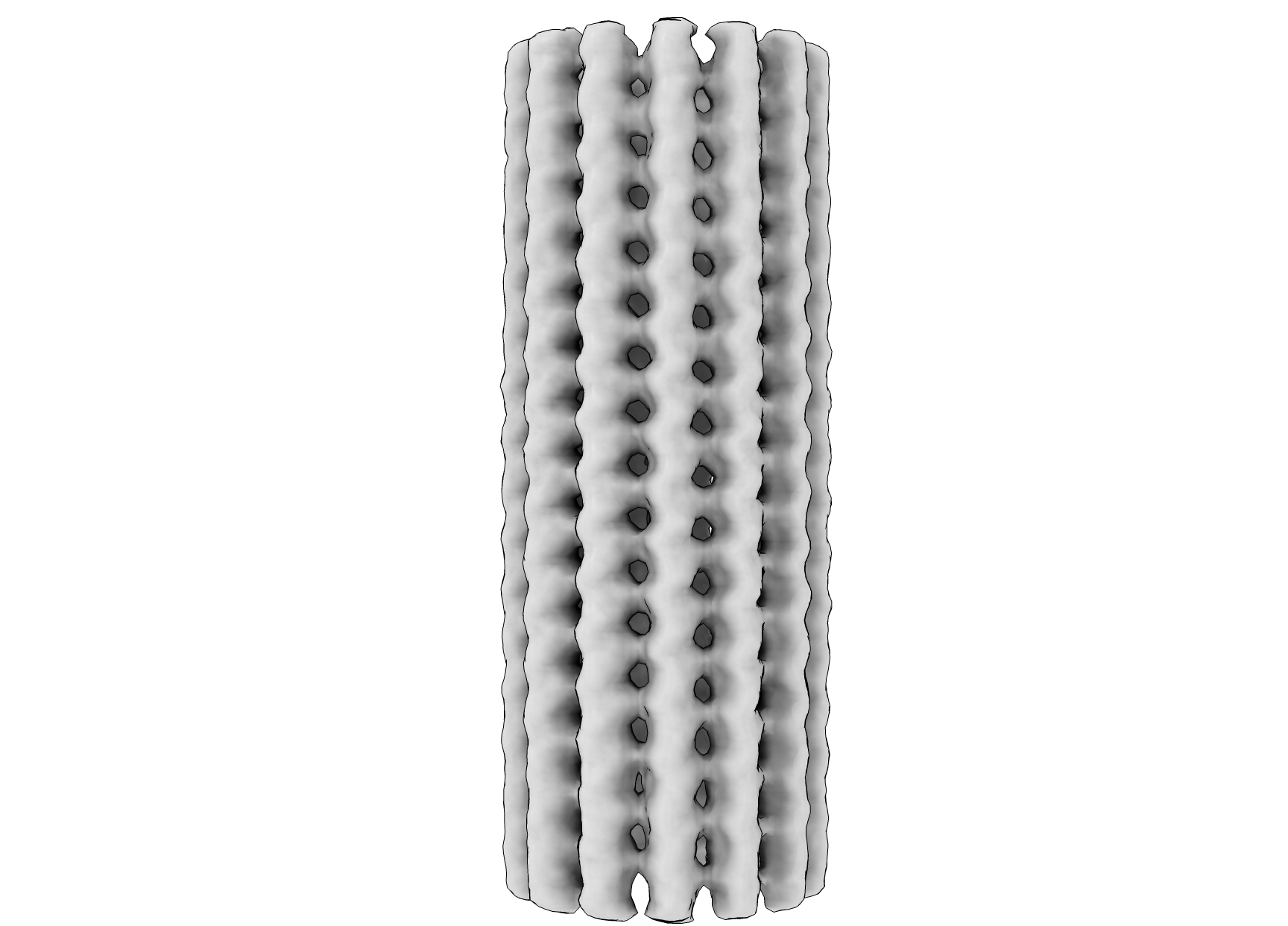

Supplement: Supplementary file 7 — Source Data for Expanded View and Appendix [file EMBR-24-e57264-s003.zip › EMBOR-2023-57264V1_SourceDataForExpandedViewAndAppendix/Figure_EV3/G/FigEV3G_BottomPanel_Right.png]

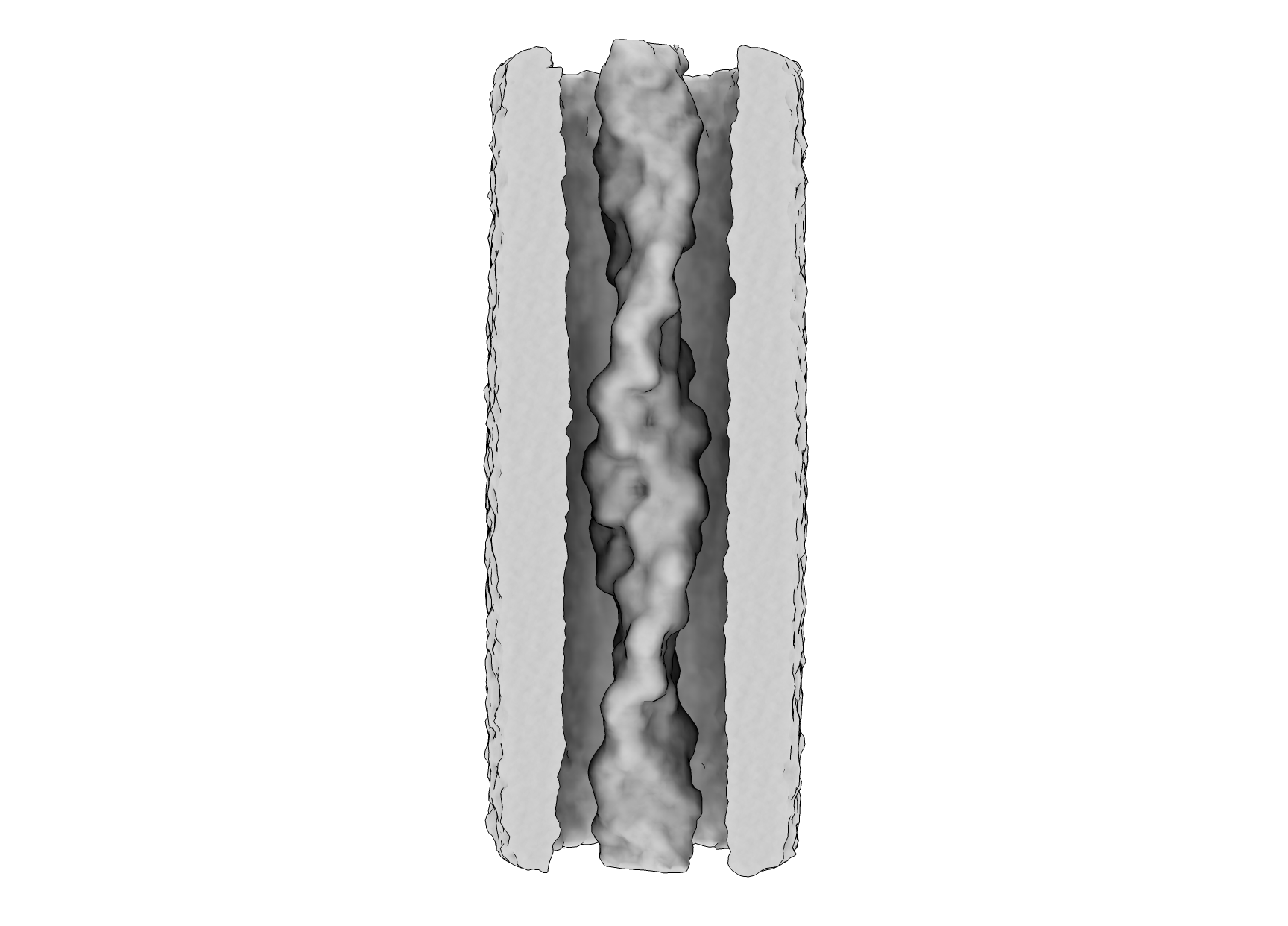

Supplement: Supplementary file 7 — Source Data for Expanded View and Appendix [file EMBR-24-e57264-s003.zip › EMBOR-2023-57264V1_SourceDataForExpandedViewAndAppendix/Figure_EV3/G/FigEV3G_TopPanel_MIddle.png]

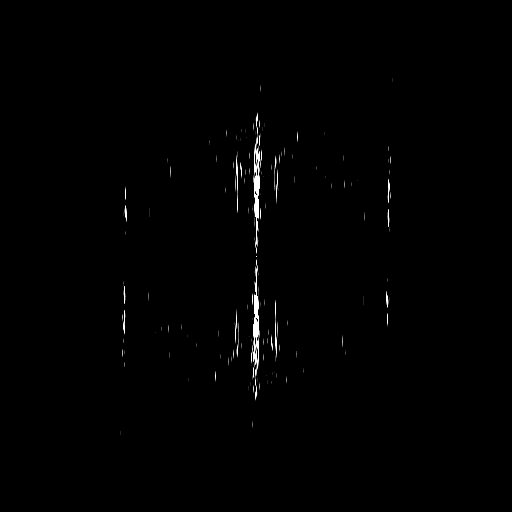

Supplement: Supplementary file 7 — Source Data for Expanded View and Appendix [file EMBR-24-e57264-s003.zip › EMBOR-2023-57264V1_SourceDataForExpandedViewAndAppendix/Figure_EV3/A/FigEV3A_TS_328_FFT.png]

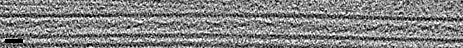

Supplement: Supplementary file 7 — Source Data for Expanded View and Appendix [file EMBR-24-e57264-s003.zip › EMBOR-2023-57264V1_SourceDataForExpandedViewAndAppendix/Figure_EV3/A/FigEV3A_TS_328_10.64nmThick_forFFT_scale20nm.png]

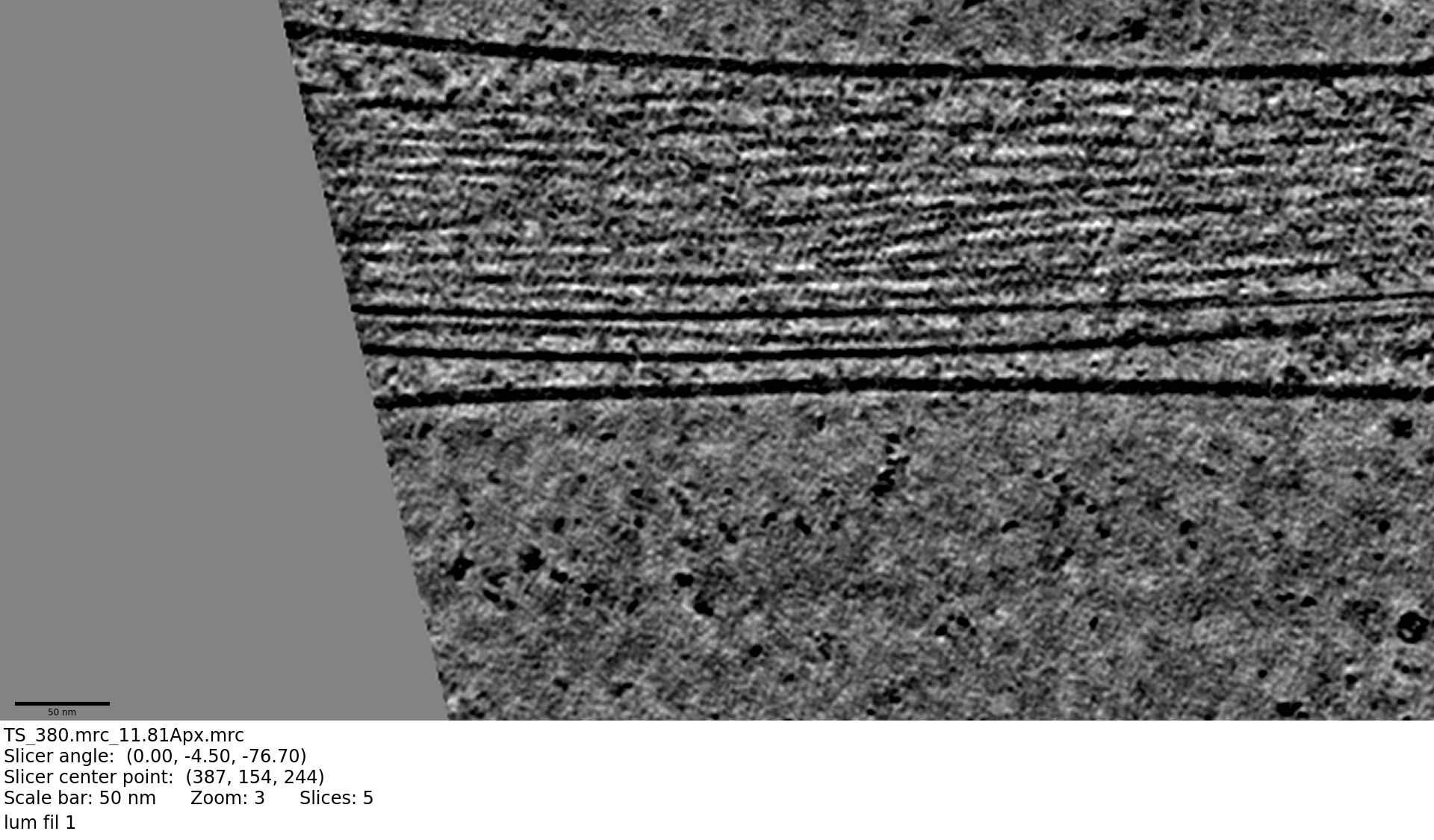

Supplement: Supplementary file 7 — Source Data for Expanded View and Appendix [file EMBR-24-e57264-s003.zip › EMBOR-2023-57264V1_SourceDataForExpandedViewAndAppendix/Figure_EV3/M/FigEV3M_Non-CofilactinLumFil_TS_380.png]

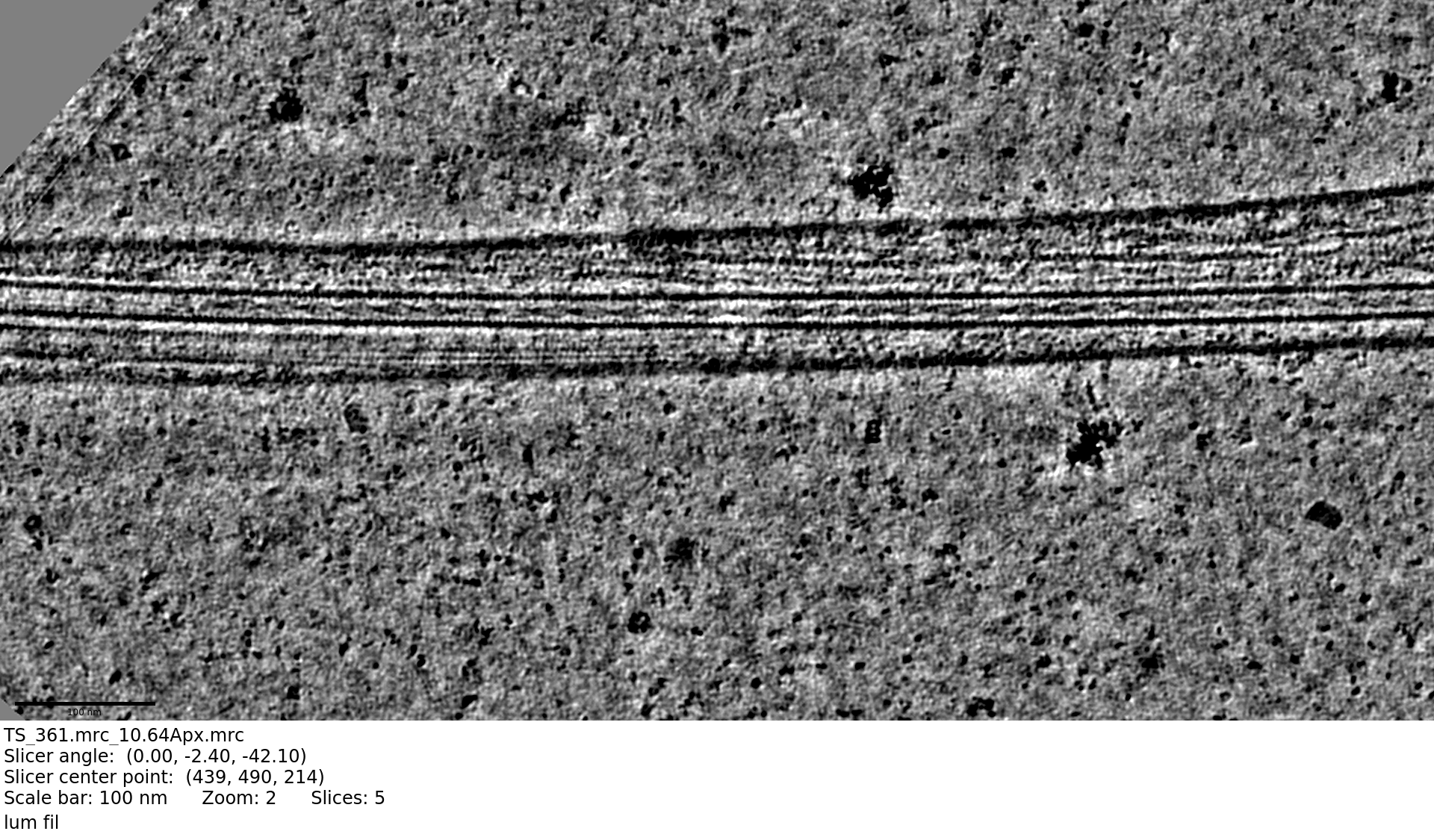

Supplement: Supplementary file 7 — Source Data for Expanded View and Appendix [file EMBR-24-e57264-s003.zip › EMBOR-2023-57264V1_SourceDataForExpandedViewAndAppendix/Figure_EV3/M/FigEV3M_CofilactinLumFil_TS_361.png]

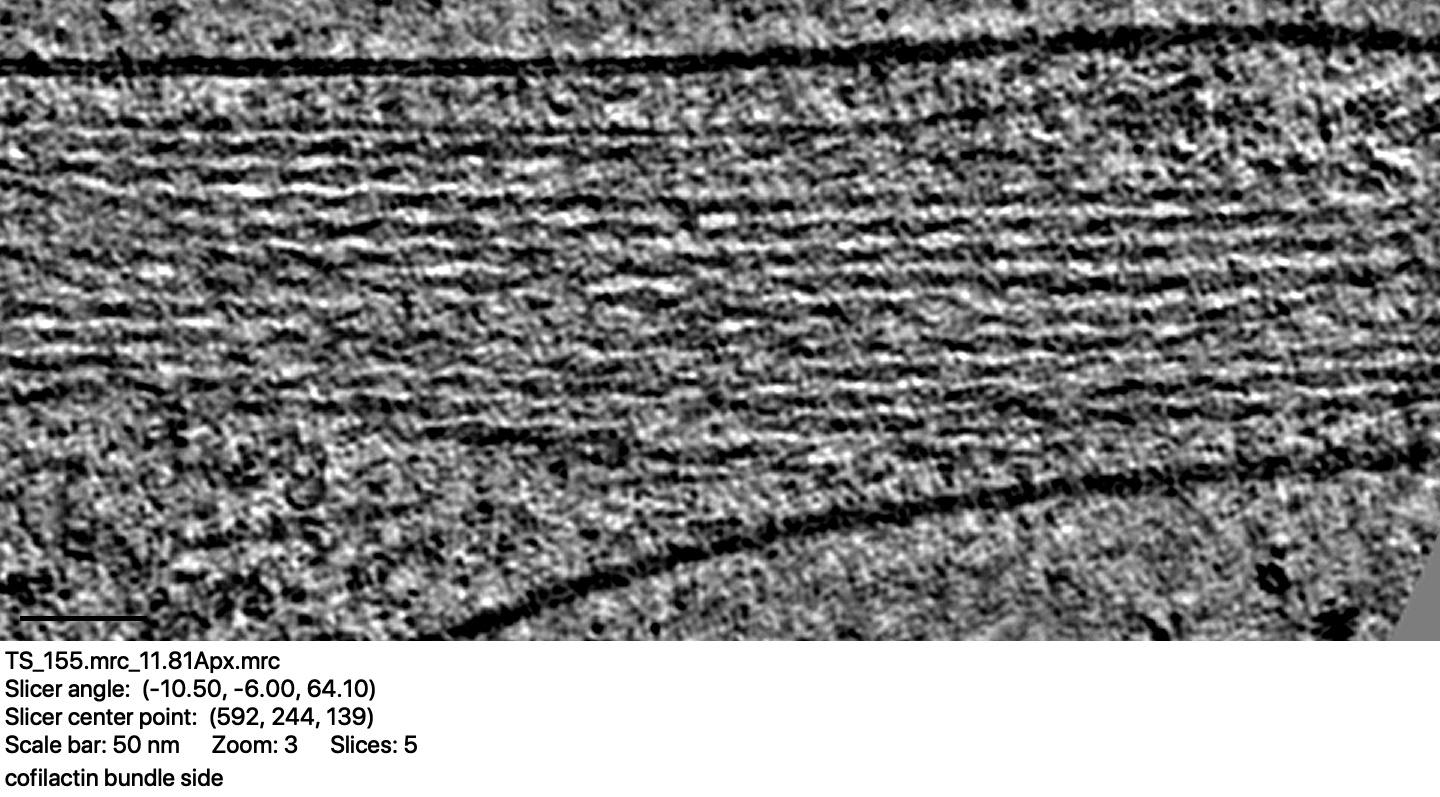

Supplement: Supplementary file 7 — Source Data for Expanded View and Appendix [file EMBR-24-e57264-s003.zip › EMBOR-2023-57264V1_SourceDataForExpandedViewAndAppendix/Figure_EV3/J/FigEV3J_220720_TS_155_CofilactinBundle.png]

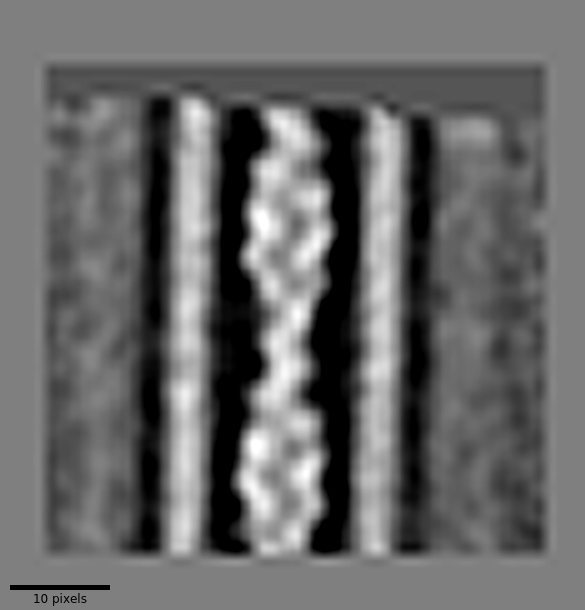

Supplement: Supplementary file 7 — Source Data for Expanded View and Appendix [file EMBR-24-e57264-s003.zip › EMBOR-2023-57264V1_SourceDataForExpandedViewAndAppendix/Figure_EV3/D/220412_FE2FE4_mod039_IndFilAvg_rot180Resmpl_inv_thick5_brighter.png]

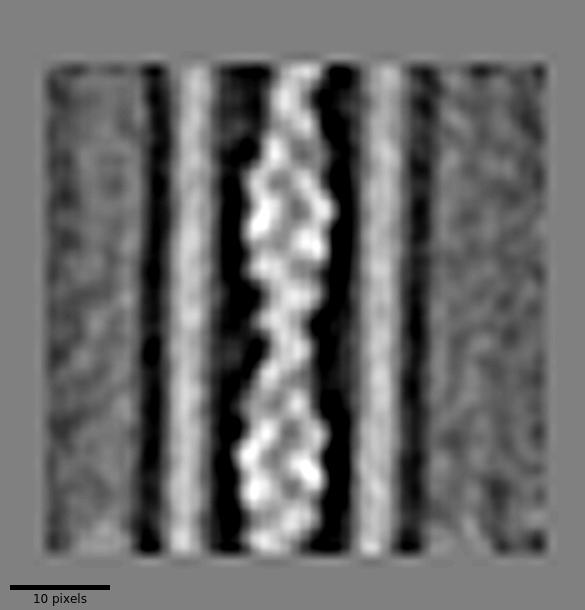

Supplement: Supplementary file 7 — Source Data for Expanded View and Appendix [file EMBR-24-e57264-s003.zip › EMBOR-2023-57264V1_SourceDataForExpandedViewAndAppendix/Figure_EV3/D/220412_FE2FE4_mod039_IndFilAvg_inv_thick5.png]

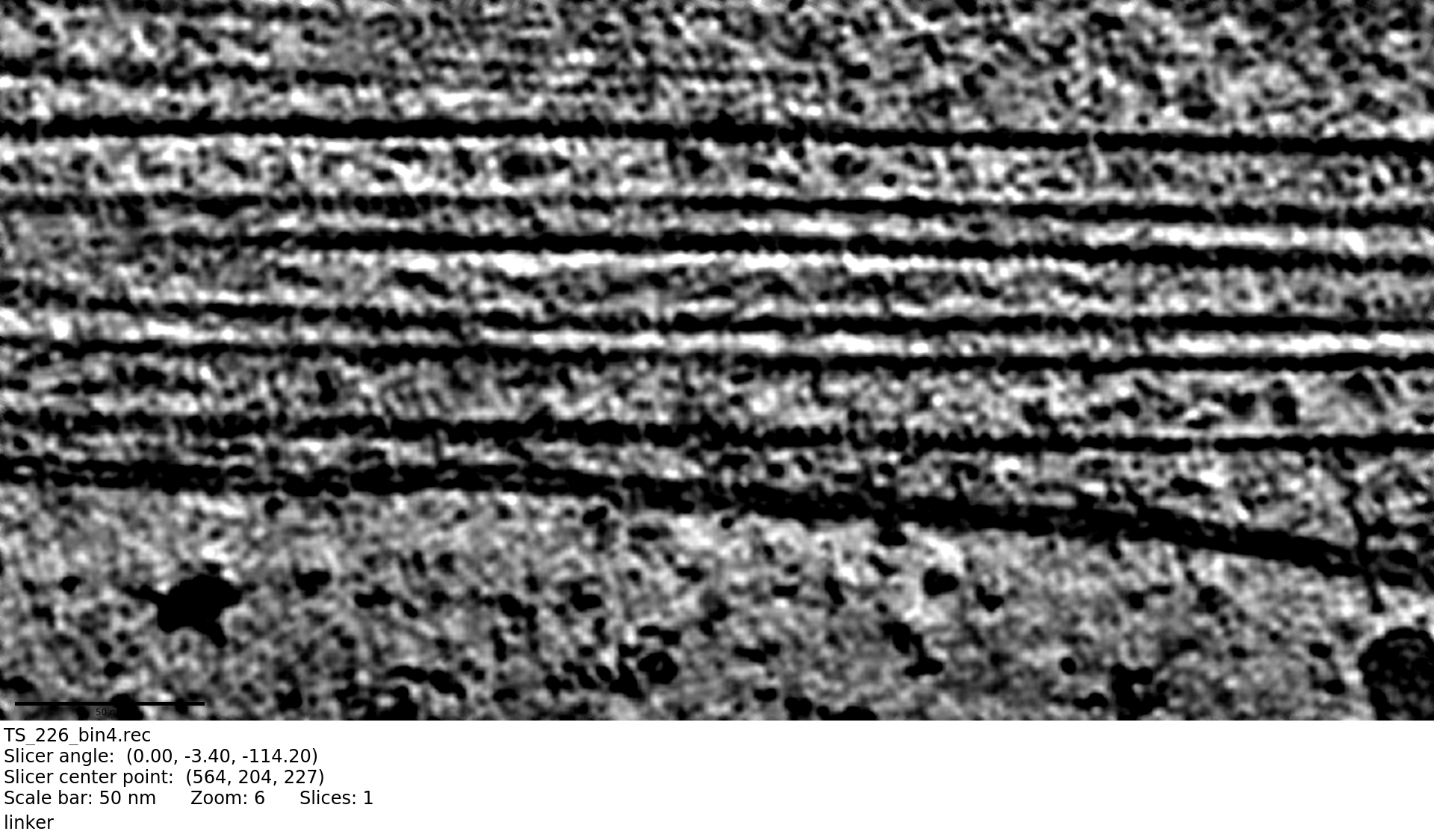

Supplement: Supplementary file 7 — Source Data for Expanded View and Appendix [file EMBR-24-e57264-s003.zip › EMBOR-2023-57264V1_SourceDataForExpandedViewAndAppendix/Figure_EV3/Q/FigEV3Q_Linker_TS_226_2nd.png]

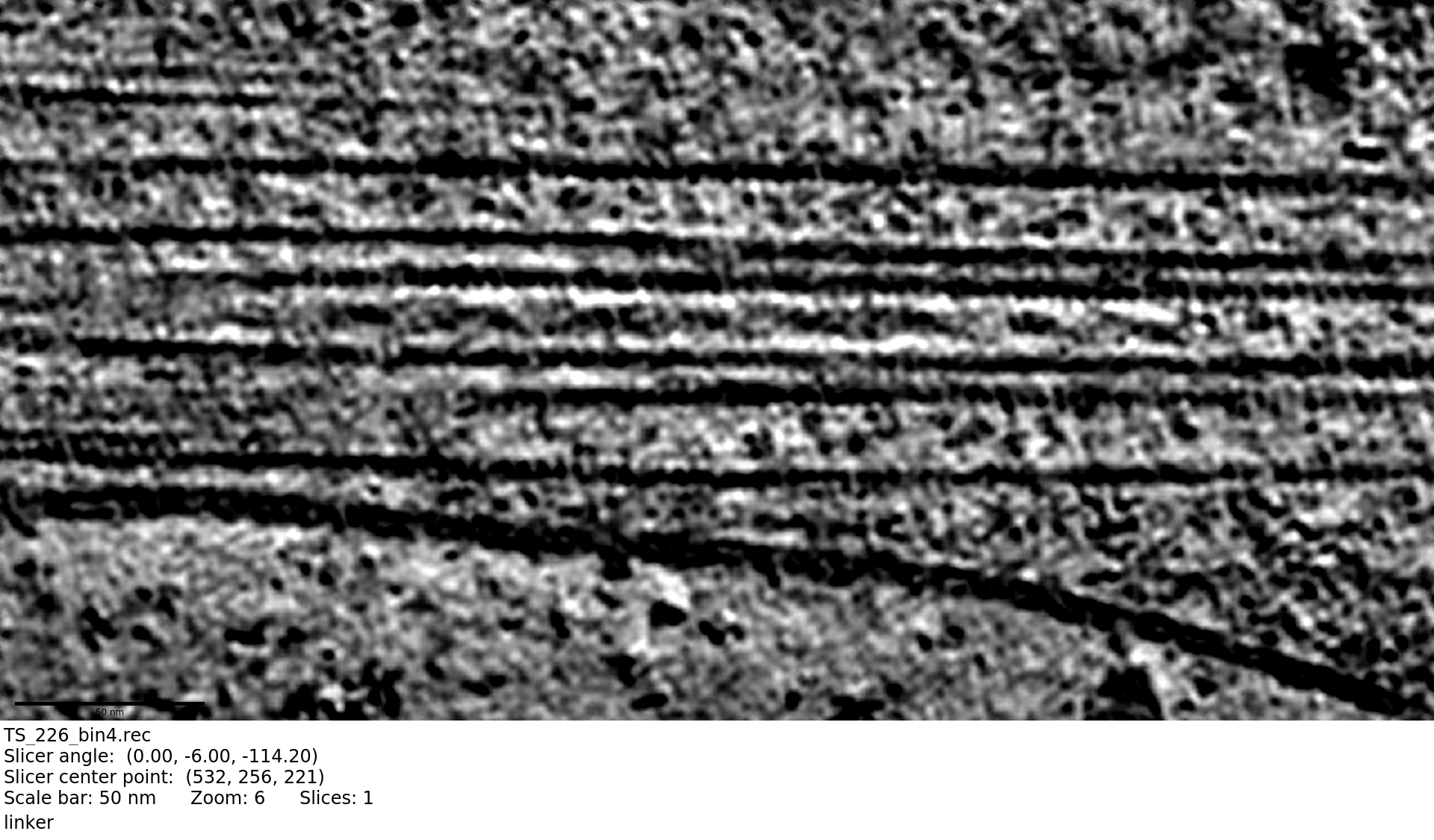

Supplement: Supplementary file 7 — Source Data for Expanded View and Appendix [file EMBR-24-e57264-s003.zip › EMBOR-2023-57264V1_SourceDataForExpandedViewAndAppendix/Figure_EV3/Q/FigEV3Q_Linker_TS_226_3rd.png]

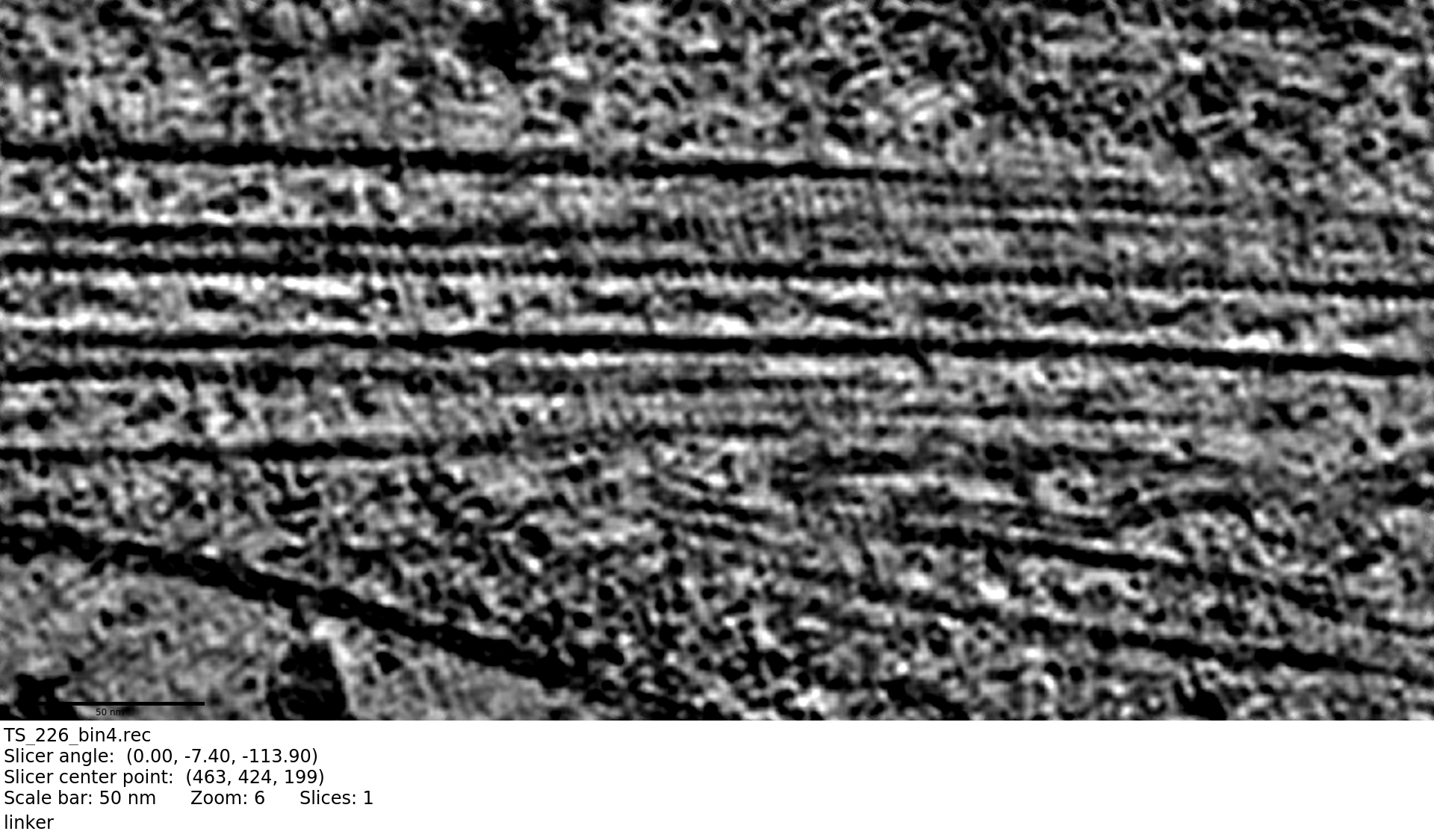

Supplement: Supplementary file 7 — Source Data for Expanded View and Appendix [file EMBR-24-e57264-s003.zip › EMBOR-2023-57264V1_SourceDataForExpandedViewAndAppendix/Figure_EV3/Q/FigEV3Q_Linker_TS_226_1st.png]

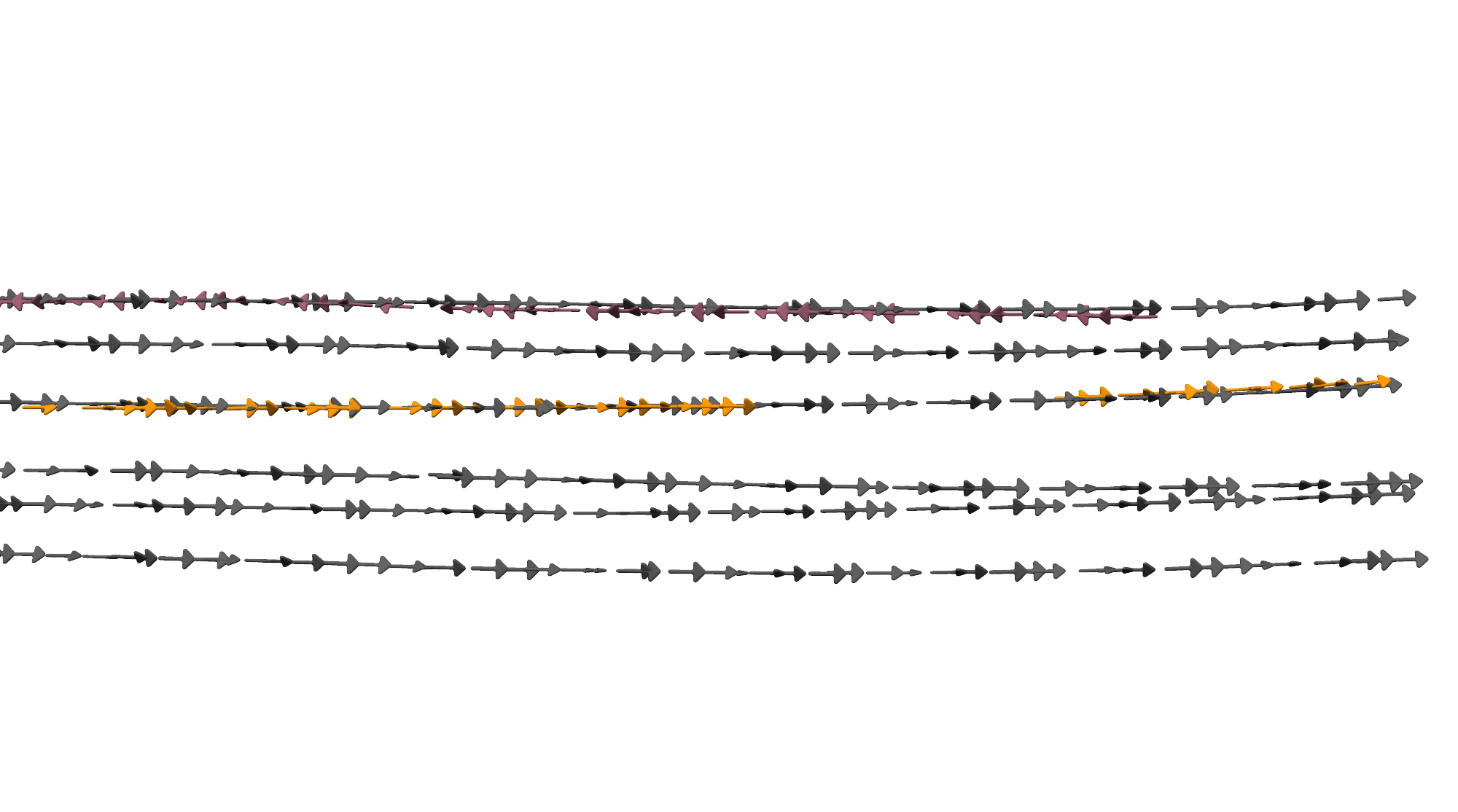

Supplement: Supplementary file 7 — Source Data for Expanded View and Appendix [file EMBR-24-e57264-s003.zip › EMBOR-2023-57264V1_SourceDataForExpandedViewAndAppendix/Figure_EV3/E/230615_TS_328_MTsGrey_LumFilPurple_MT176TOP_Orange_MT177BottomLeft_BottomRight178.png]

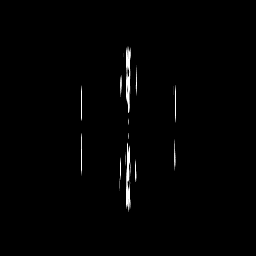

Supplement: Supplementary file 7 — Source Data for Expanded View and Appendix [file EMBR-24-e57264-s003.zip › EMBOR-2023-57264V1_SourceDataForExpandedViewAndAppendix/Figure_EV3/B/FigEV3B_TS_215_masked_project_FFT_BinBy2.png]

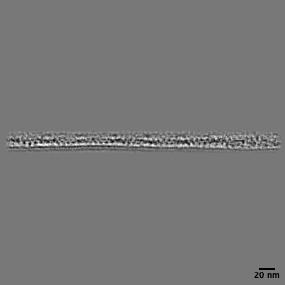

Supplement: Supplementary file 7 — Source Data for Expanded View and Appendix [file EMBR-24-e57264-s003.zip › EMBOR-2023-57264V1_SourceDataForExpandedViewAndAppendix/Figure_EV3/B/TS_215.mrc_11.81Apx_Trim_Rot2_bx285_masked_project.png]

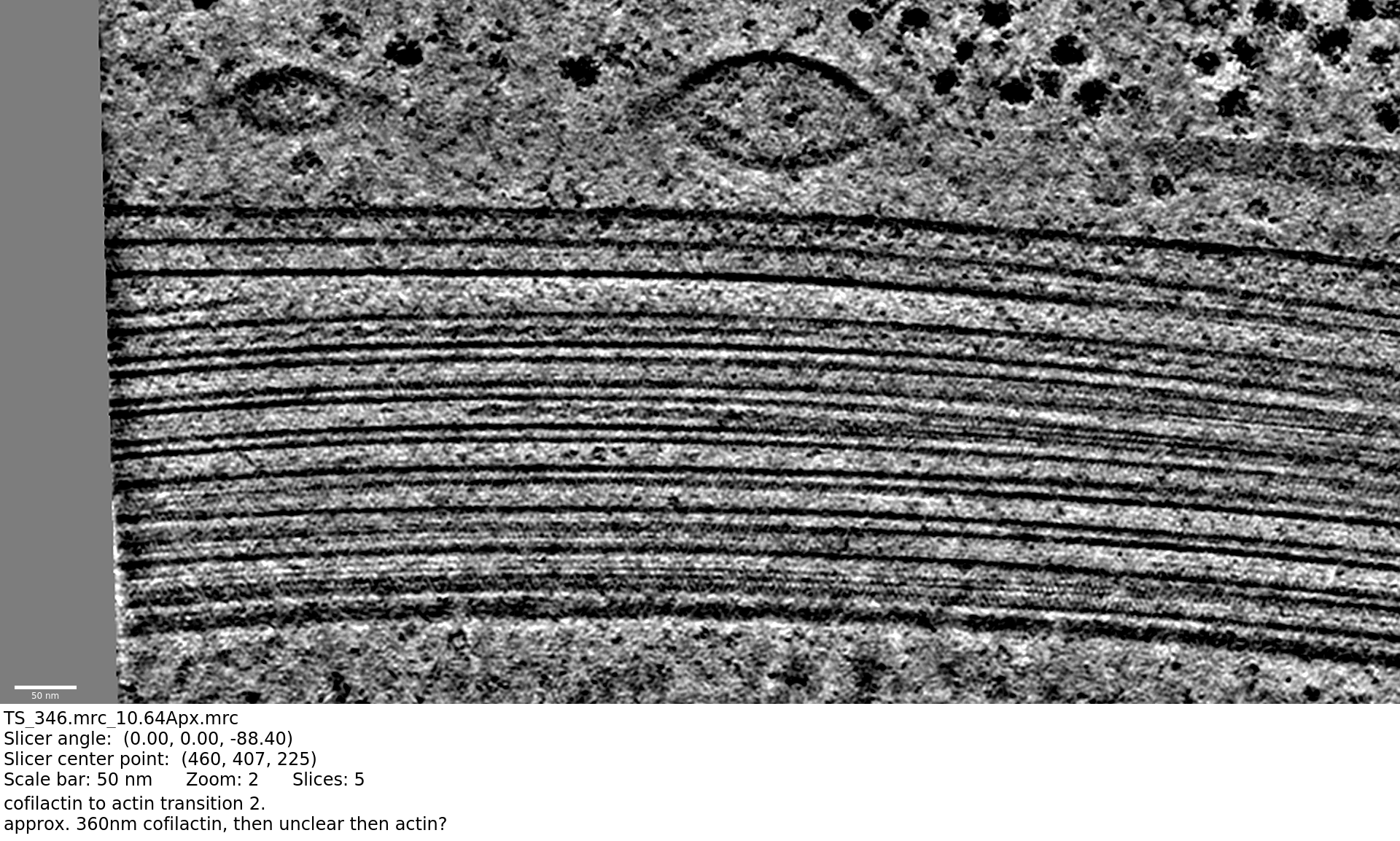

Supplement: Supplementary file 7 — Source Data for Expanded View and Appendix [file EMBR-24-e57264-s003.zip › EMBOR-2023-57264V1_SourceDataForExpandedViewAndAppendix/Figure_EV3/P/FigEV3P_TS_346_LumFilTransition_Cofilactin_non-Cofilactin.png]

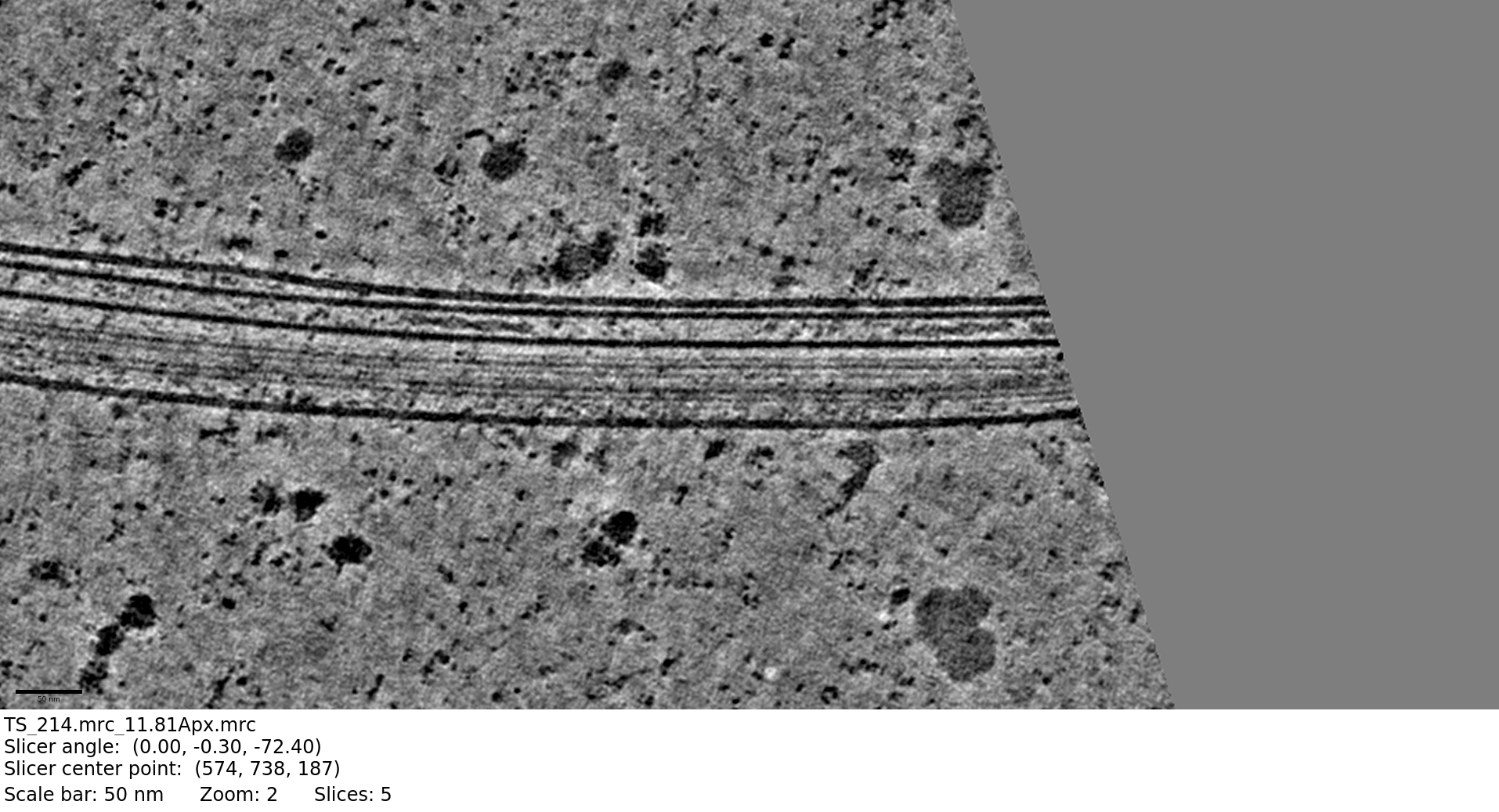

Supplement: Supplementary file 7 — Source Data for Expanded View and Appendix [file EMBR-24-e57264-s003.zip › EMBOR-2023-57264V1_SourceDataForExpandedViewAndAppendix/Figure_EV2/C/FigEV2C_TOP_220617_TS_214_2FilsPerMT.png]

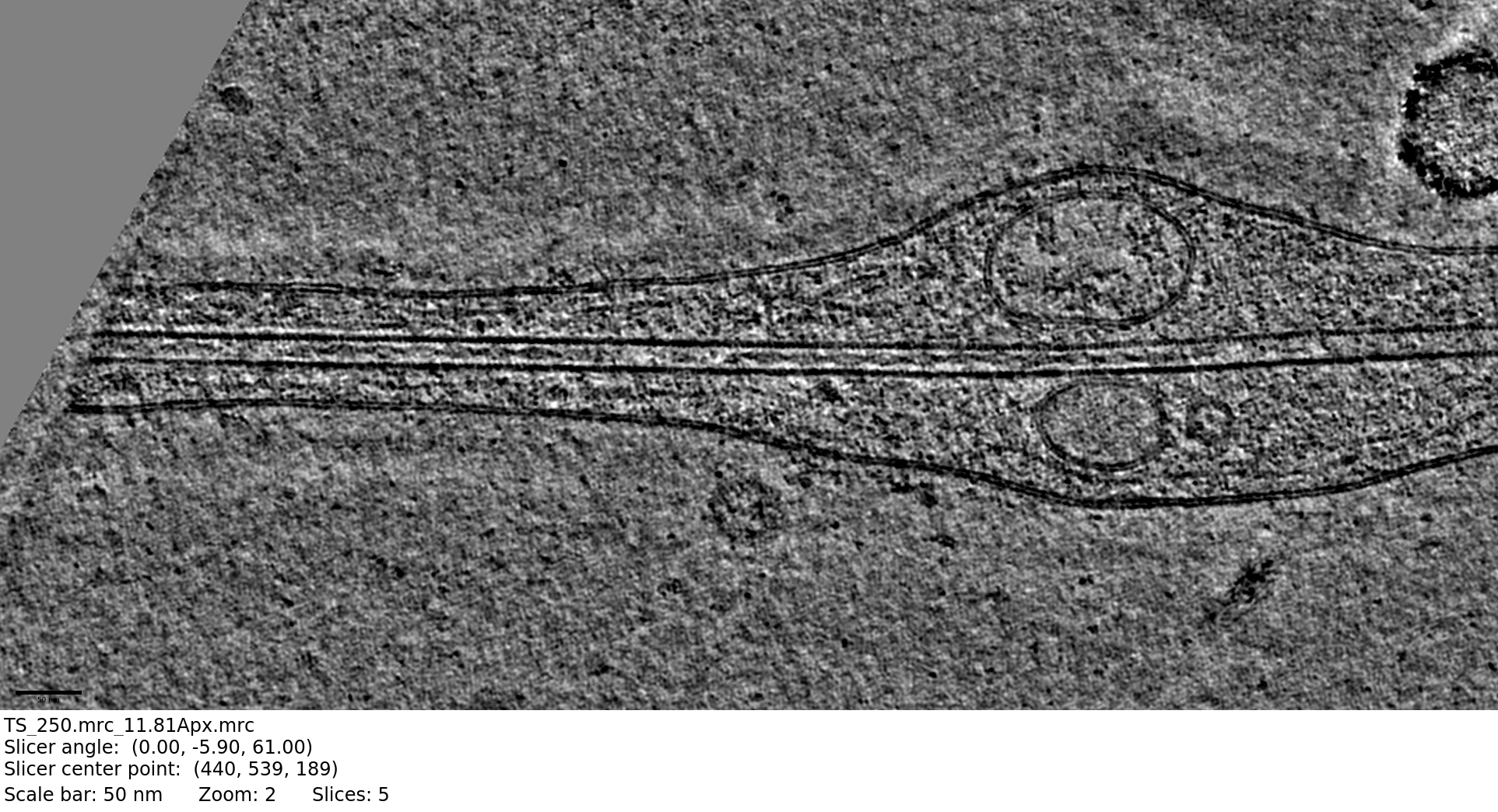

Supplement: Supplementary file 7 — Source Data for Expanded View and Appendix [file EMBR-24-e57264-s003.zip › EMBOR-2023-57264V1_SourceDataForExpandedViewAndAppendix/Figure_EV2/C/FigEV2C_BOTTOM_220720_TS_250_2FilsPerMT.png]

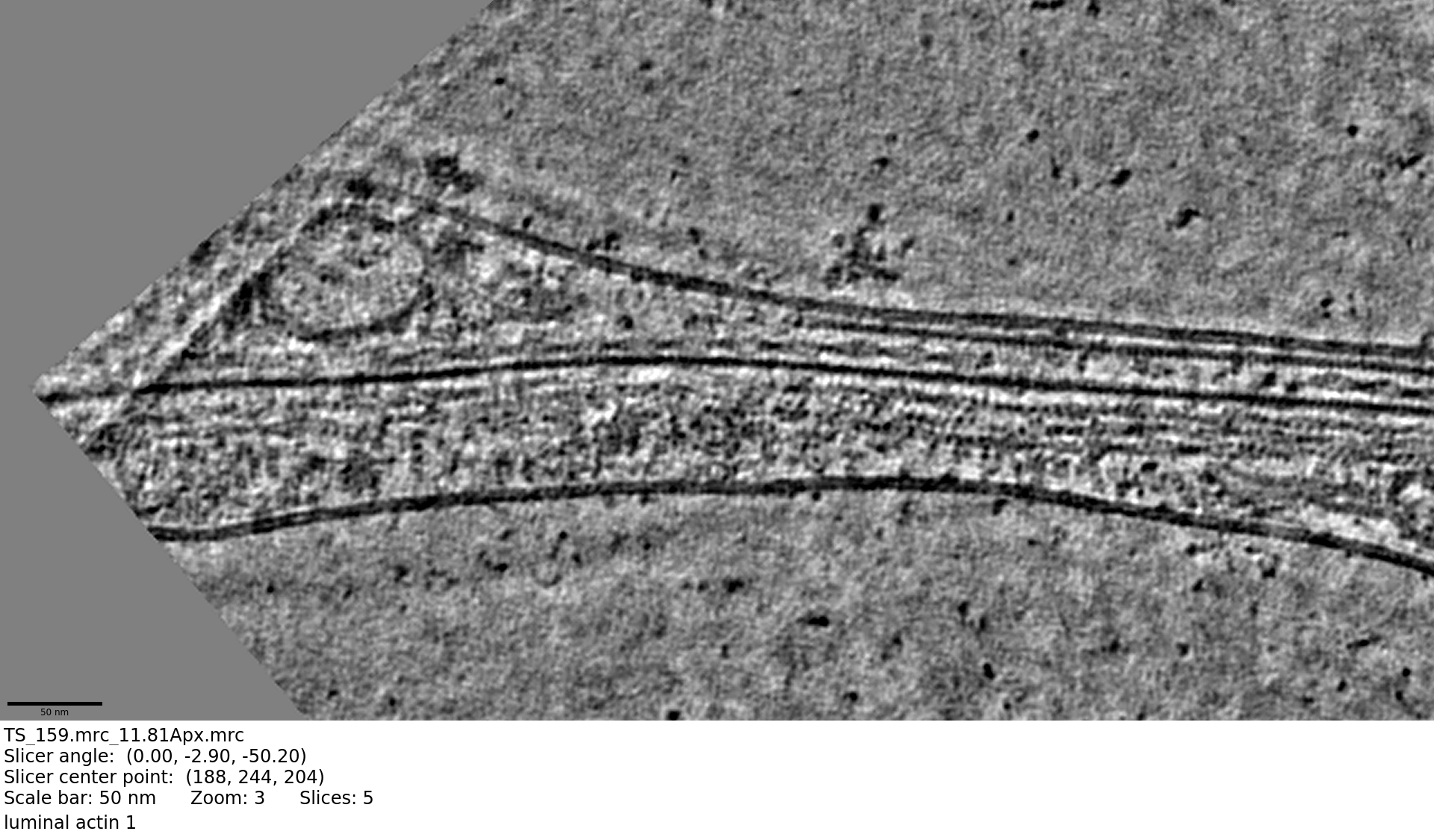

Supplement: Supplementary file 7 — Source Data for Expanded View and Appendix [file EMBR-24-e57264-s003.zip › EMBOR-2023-57264V1_SourceDataForExpandedViewAndAppendix/Figure_EV2/D/FigEV2D_LumFil1.png]

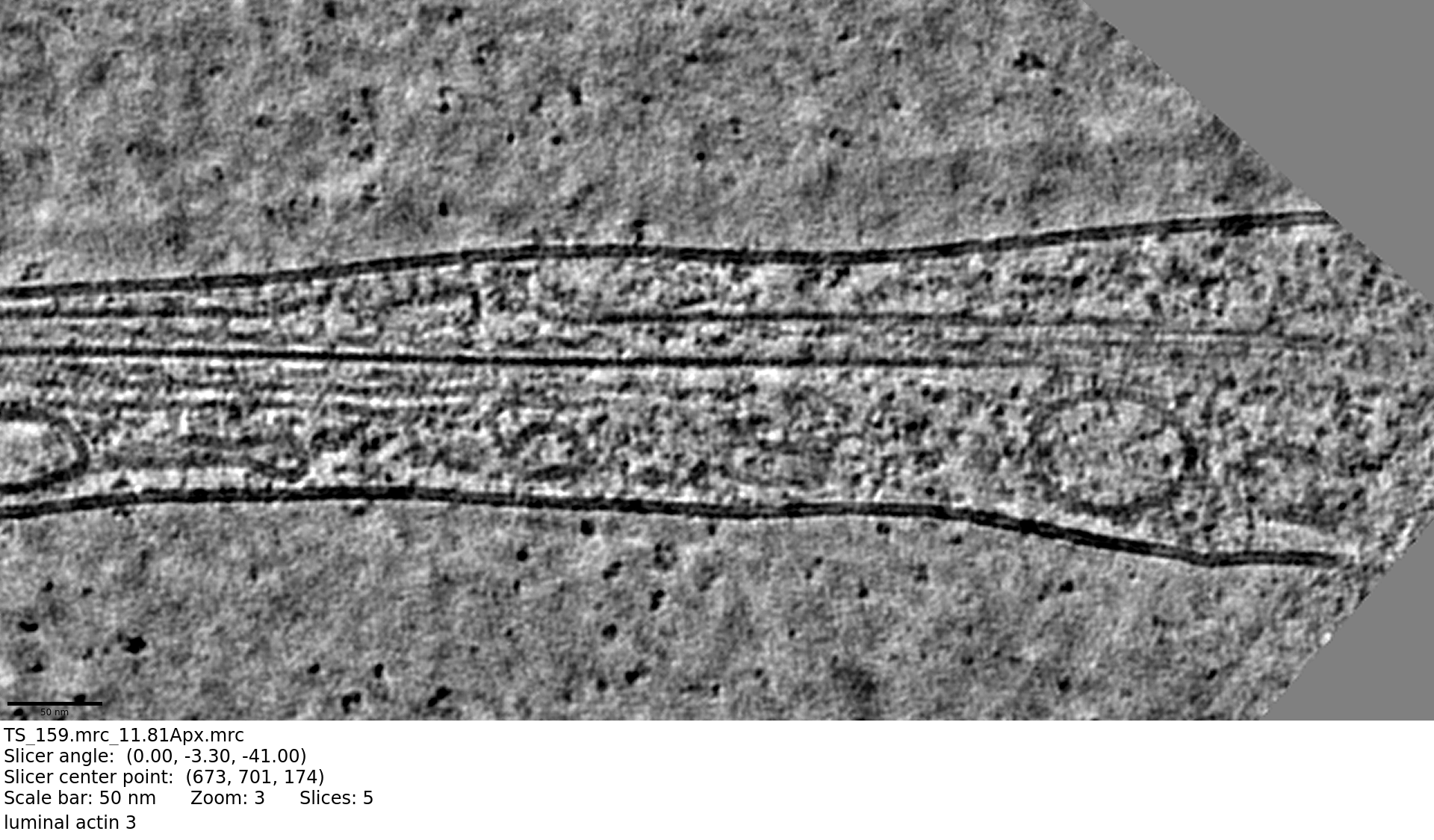

Supplement: Supplementary file 7 — Source Data for Expanded View and Appendix [file EMBR-24-e57264-s003.zip › EMBOR-2023-57264V1_SourceDataForExpandedViewAndAppendix/Figure_EV2/D/FigEV2D_LumFil3.png]

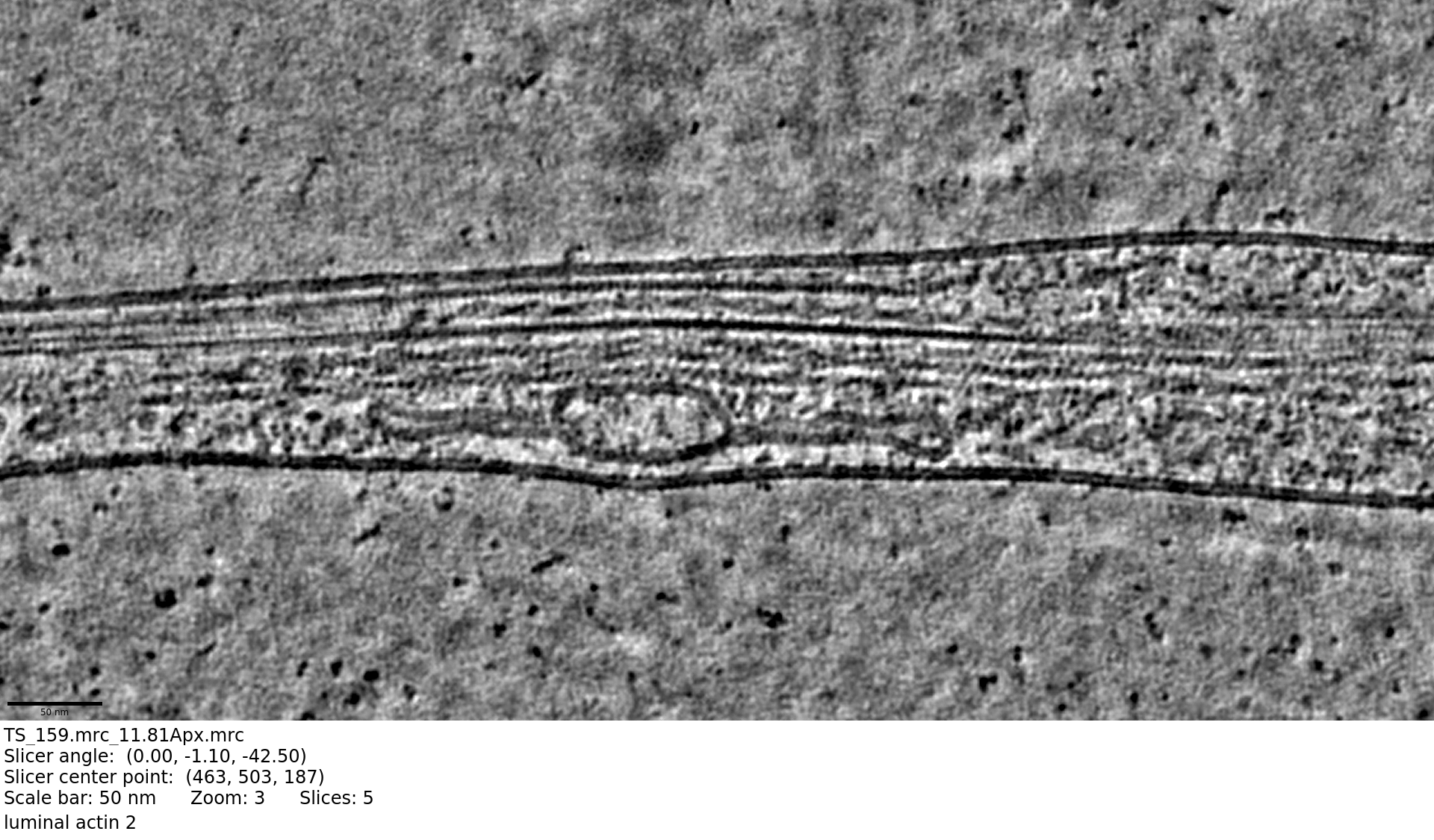

Supplement: Supplementary file 7 — Source Data for Expanded View and Appendix [file EMBR-24-e57264-s003.zip › EMBOR-2023-57264V1_SourceDataForExpandedViewAndAppendix/Figure_EV2/D/FigEV2D_LumFil2.png]

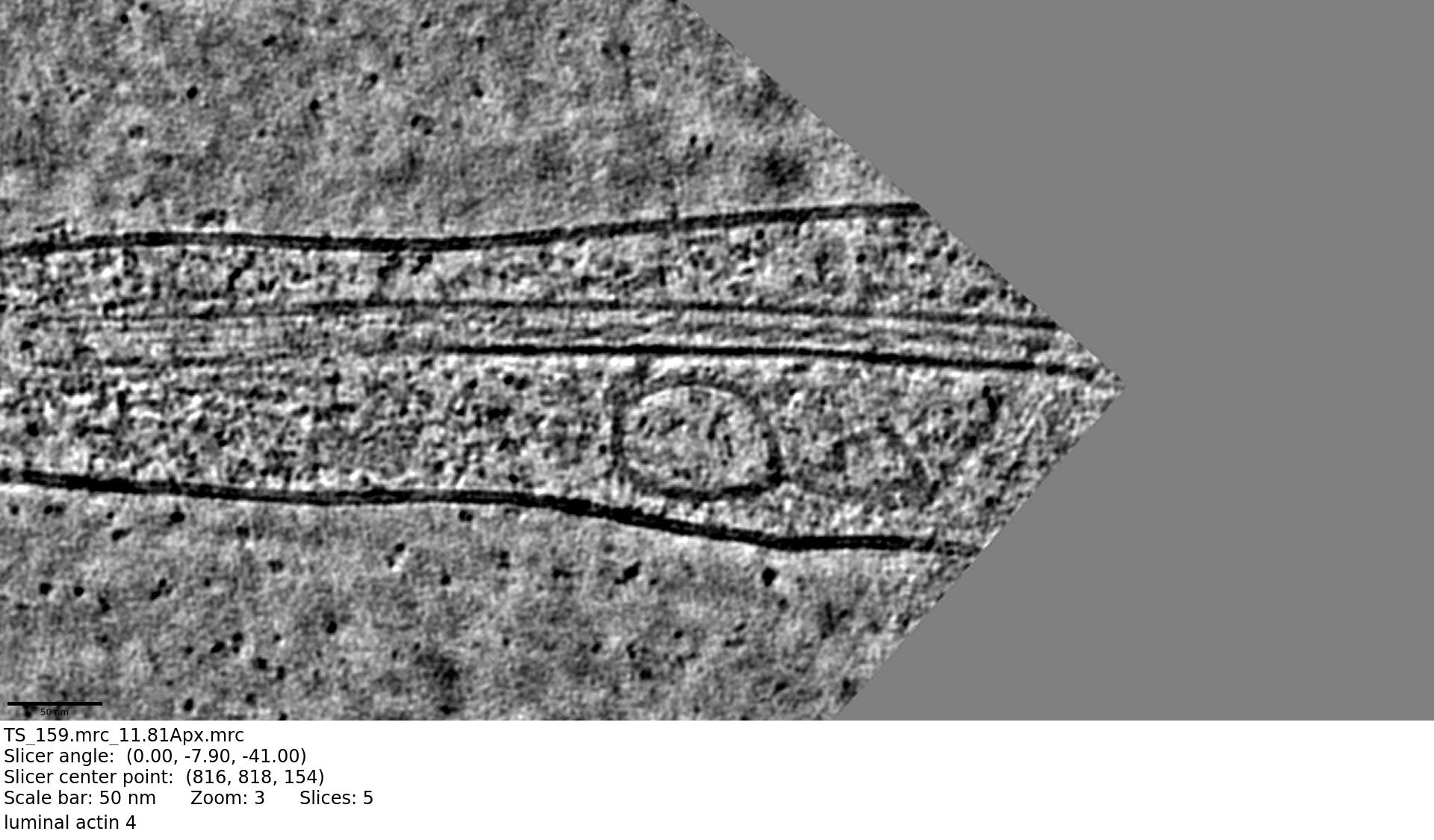

Supplement: Supplementary file 7 — Source Data for Expanded View and Appendix [file EMBR-24-e57264-s003.zip › EMBOR-2023-57264V1_SourceDataForExpandedViewAndAppendix/Figure_EV2/D/FigEV2D_LumFil4.png]

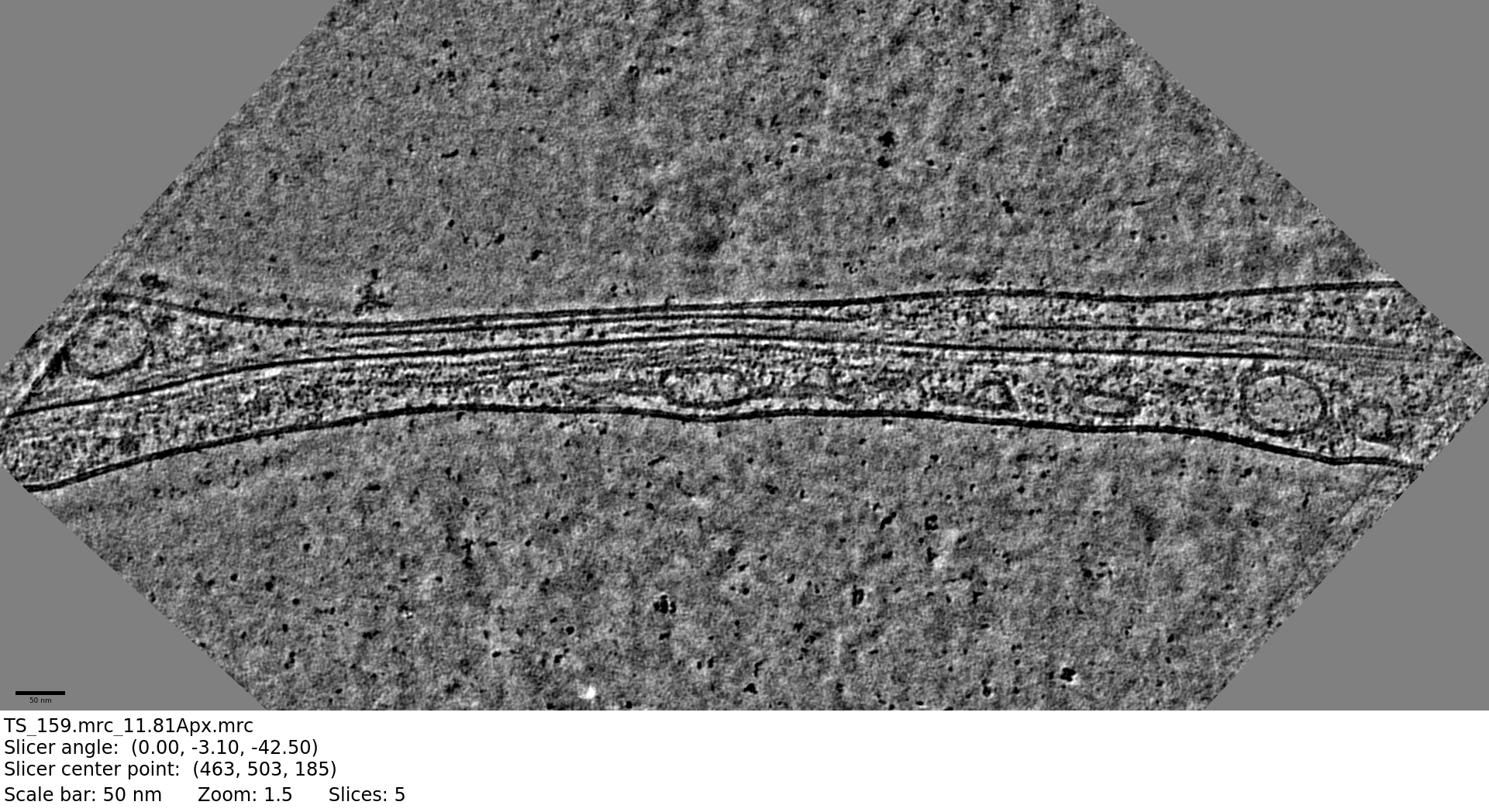

Supplement: Supplementary file 7 — Source Data for Expanded View and Appendix [file EMBR-24-e57264-s003.zip › EMBOR-2023-57264V1_SourceDataForExpandedViewAndAppendix/Figure_EV2/D/FigEV2D_Dataset6_220720_TS_159_4FilsPerMT.png]

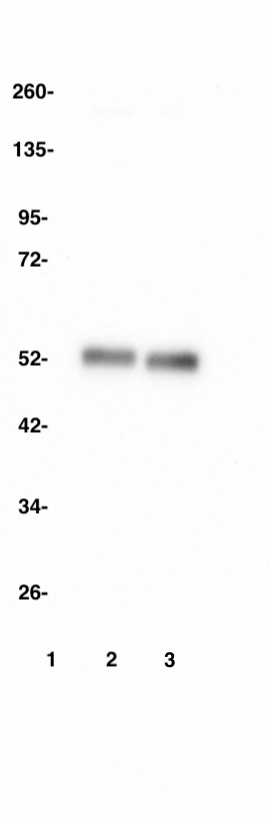

Supplement: Supplementary file 7 — Source Data for Expanded View and Appendix [file EMBR-24-e57264-s003.zip › EMBOR-2023-57264V1_SourceDataForExpandedViewAndAppendix/Appendix/Appendix_Figure_S3/A/alpha-tubulin-blot-original.tif]

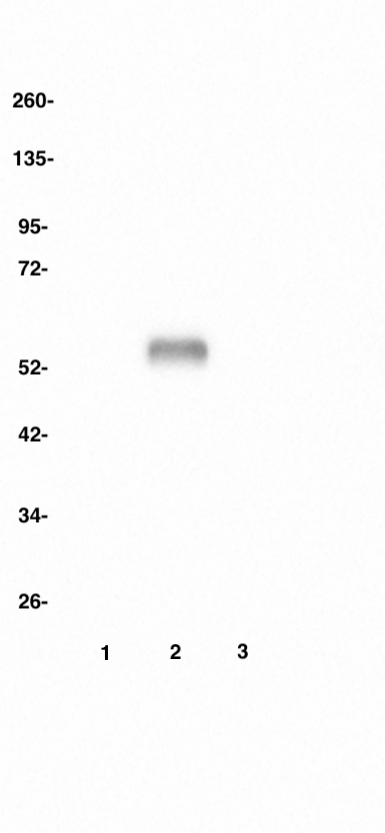

Supplement: Supplementary file 7 — Source Data for Expanded View and Appendix [file EMBR-24-e57264-s003.zip › EMBOR-2023-57264V1_SourceDataForExpandedViewAndAppendix/Appendix/Appendix_Figure_S3/A/acetylated-tubulin-blot-original.tif]

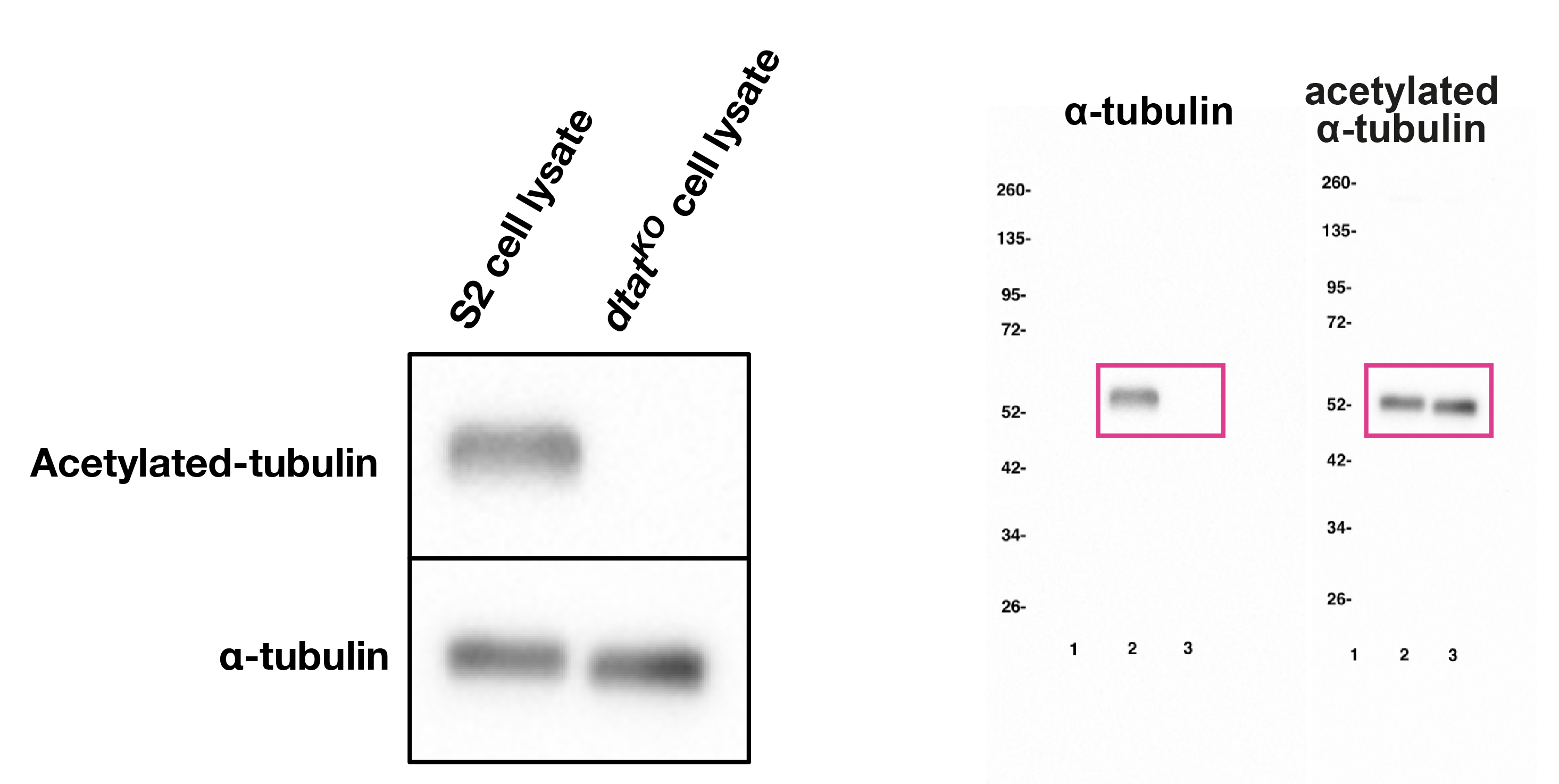

Supplement: Supplementary file 7 — Source Data for Expanded View and Appendix [file EMBR-24-e57264-s003.zip › EMBOR-2023-57264V1_SourceDataForExpandedViewAndAppendix/Appendix/Appendix_Figure_S3/A/AnnotatedBlot_dTAT_acMT_blot.png]

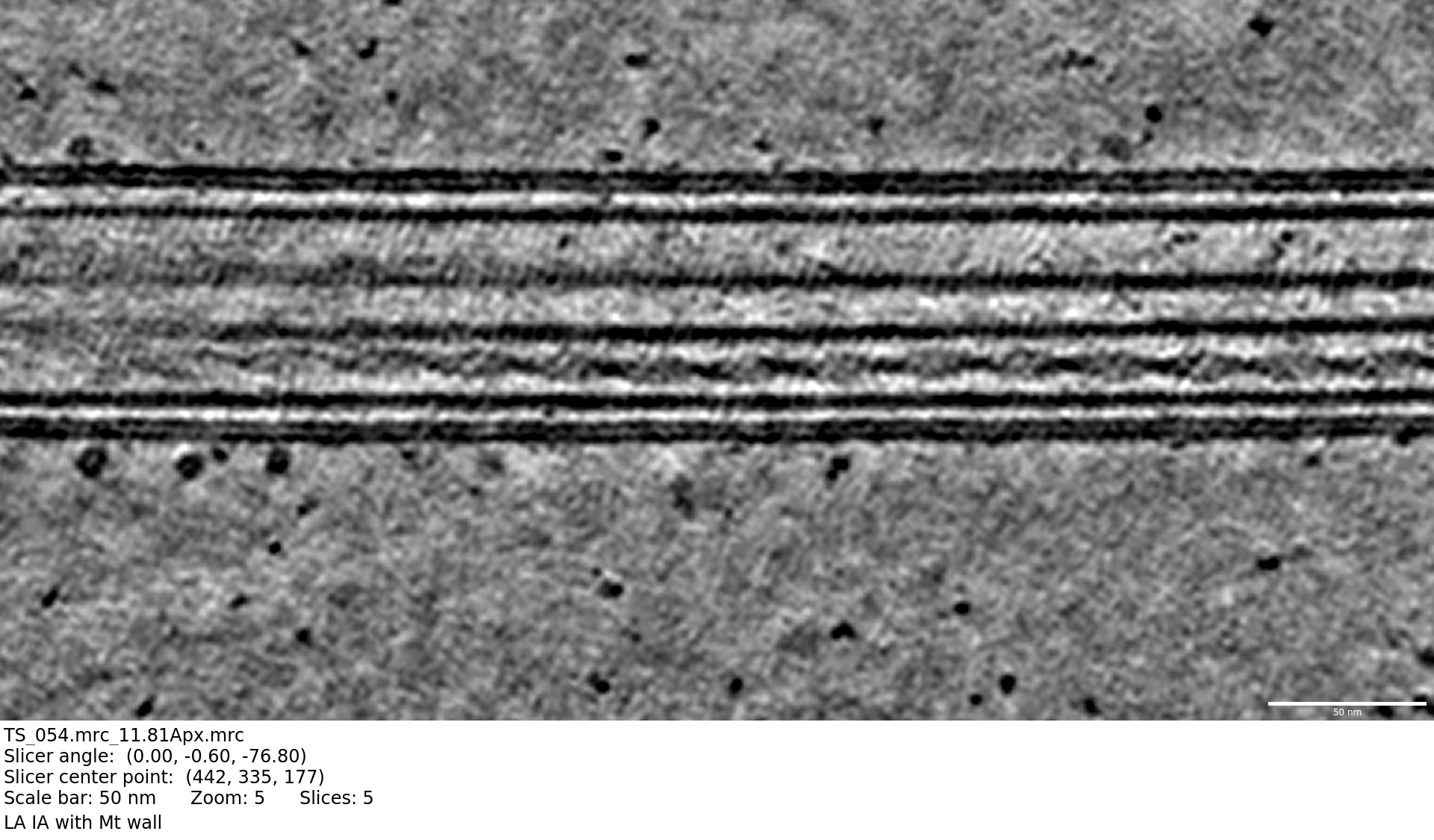

Supplement: Supplementary file 7 — Source Data for Expanded View and Appendix [file EMBR-24-e57264-s003.zip › EMBOR-2023-57264V1_SourceDataForExpandedViewAndAppendix/Appendix/Appendix_Figure_S3/C/AppendixFigS3_TS_054_dTATKO_Linkers.png]

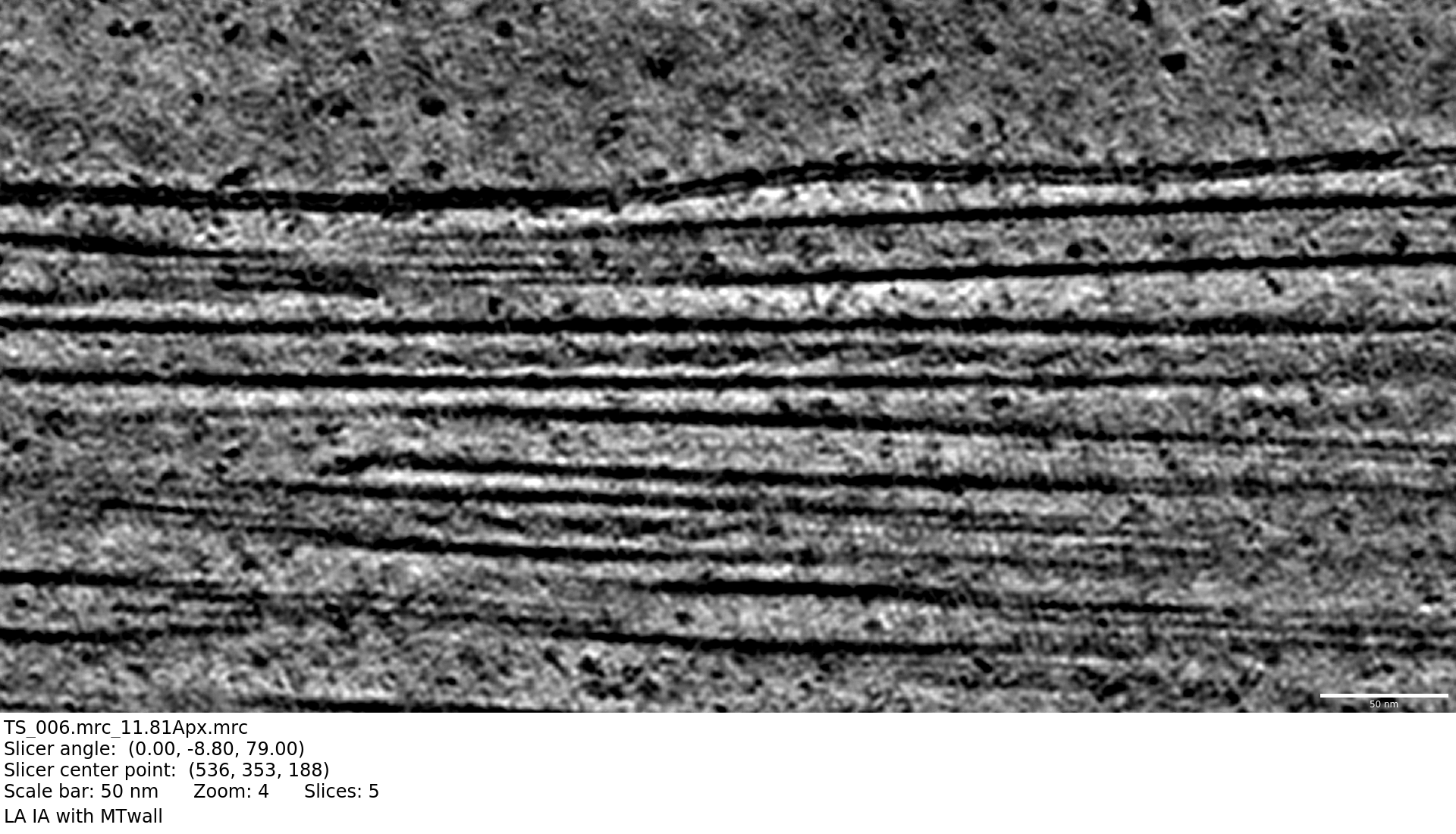

Supplement: Supplementary file 7 — Source Data for Expanded View and Appendix [file EMBR-24-e57264-s003.zip › EMBOR-2023-57264V1_SourceDataForExpandedViewAndAppendix/Appendix/Appendix_Figure_S3/C/AppendixFigS3_TS_006_dTATKO_Linkers.png]

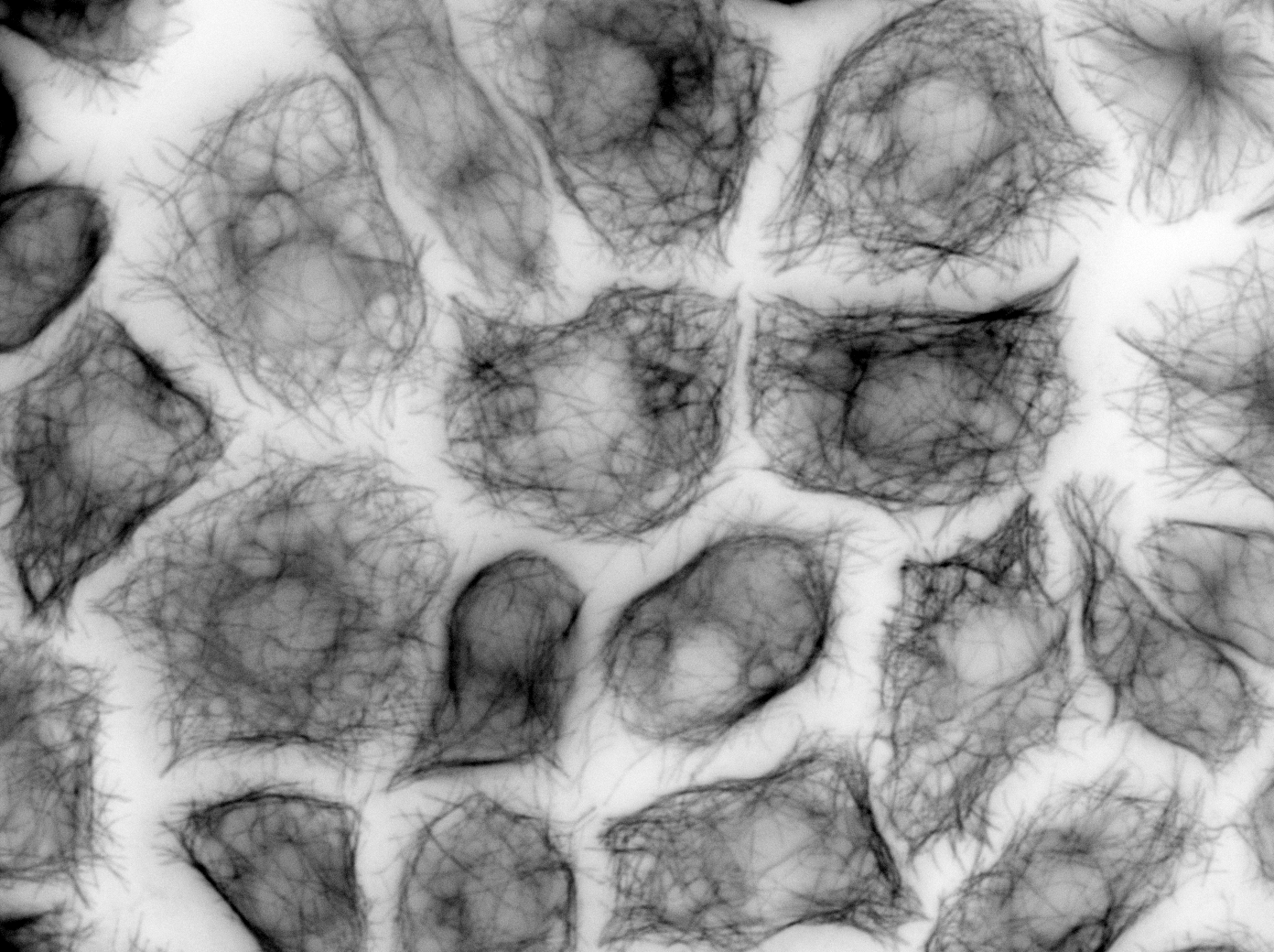

Supplement: Supplementary file 7 — Source Data for Expanded View and Appendix [file EMBR-24-e57264-s003.zip › EMBOR-2023-57264V1_SourceDataForExpandedViewAndAppendix/Appendix/Appendix_Figure_S3/B/WT_005_a-tub_577-1622.png]

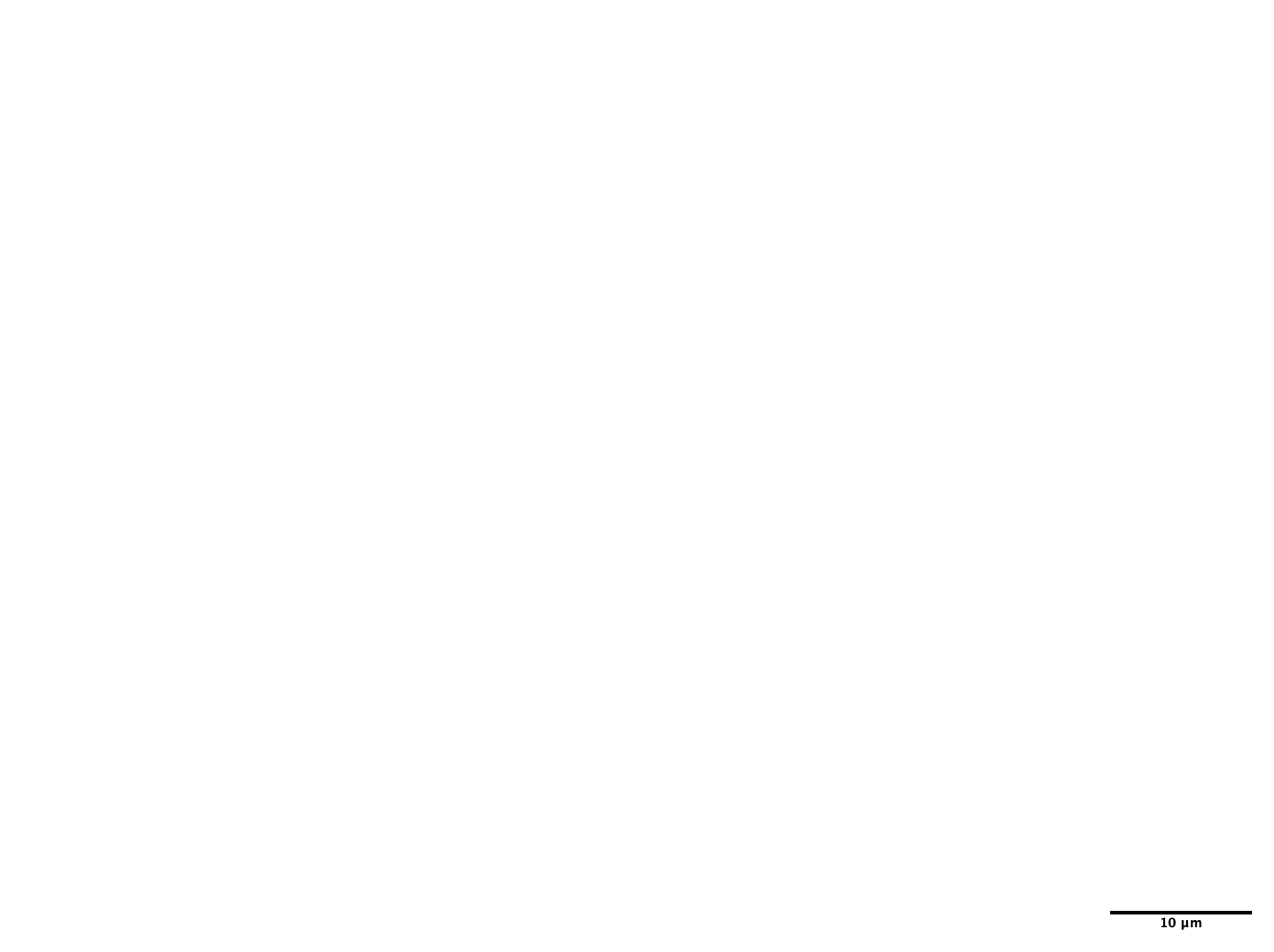

Supplement: Supplementary file 7 — Source Data for Expanded View and Appendix [file EMBR-24-e57264-s003.zip › EMBOR-2023-57264V1_SourceDataForExpandedViewAndAppendix/Appendix/Appendix_Figure_S3/B/dTAT_KO_006_acet-tub_641-5529_scale.png]

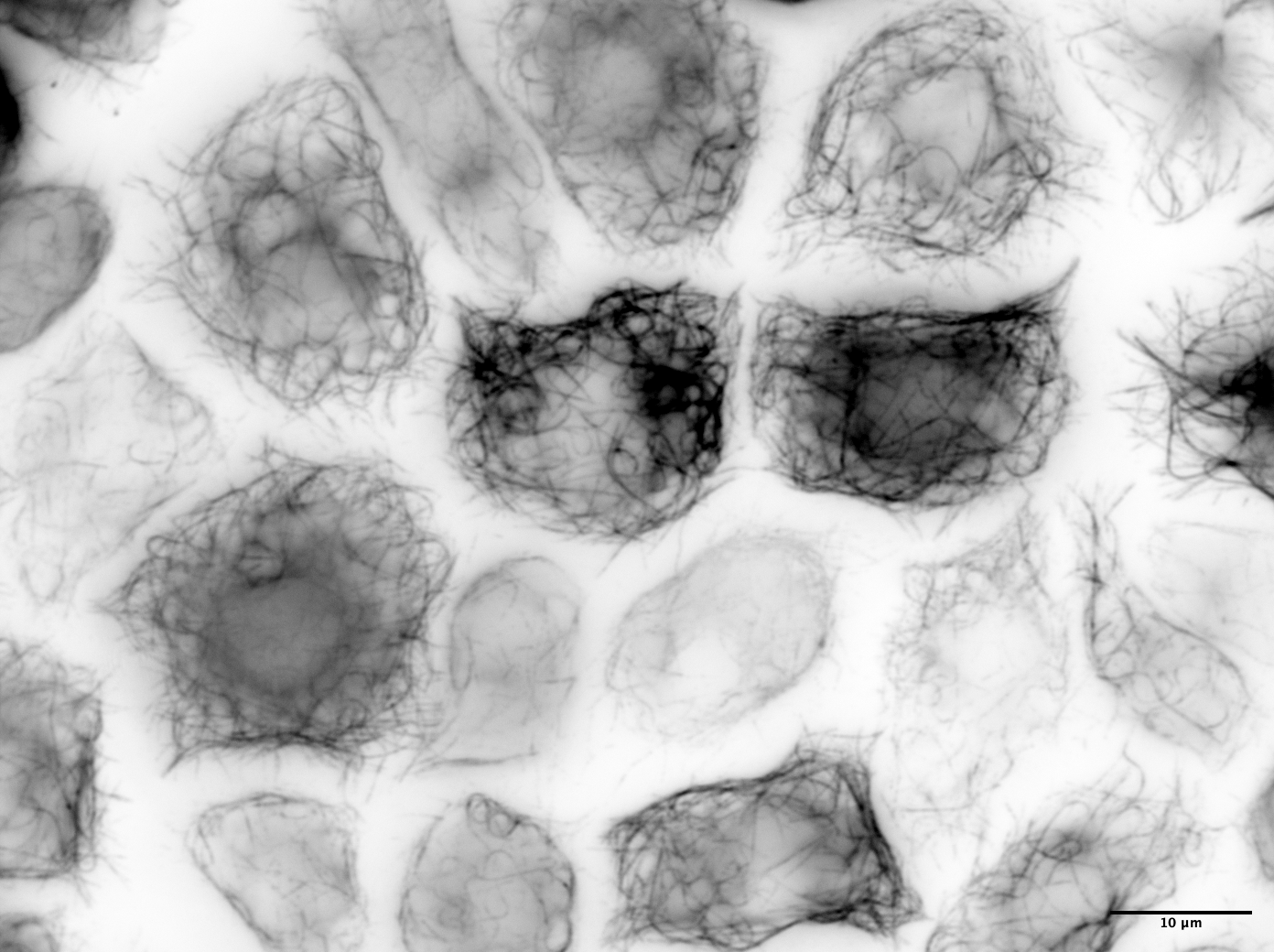

Supplement: Supplementary file 7 — Source Data for Expanded View and Appendix [file EMBR-24-e57264-s003.zip › EMBOR-2023-57264V1_SourceDataForExpandedViewAndAppendix/Appendix/Appendix_Figure_S3/B/WT_005_acet-tub_641-5529_scale.png]

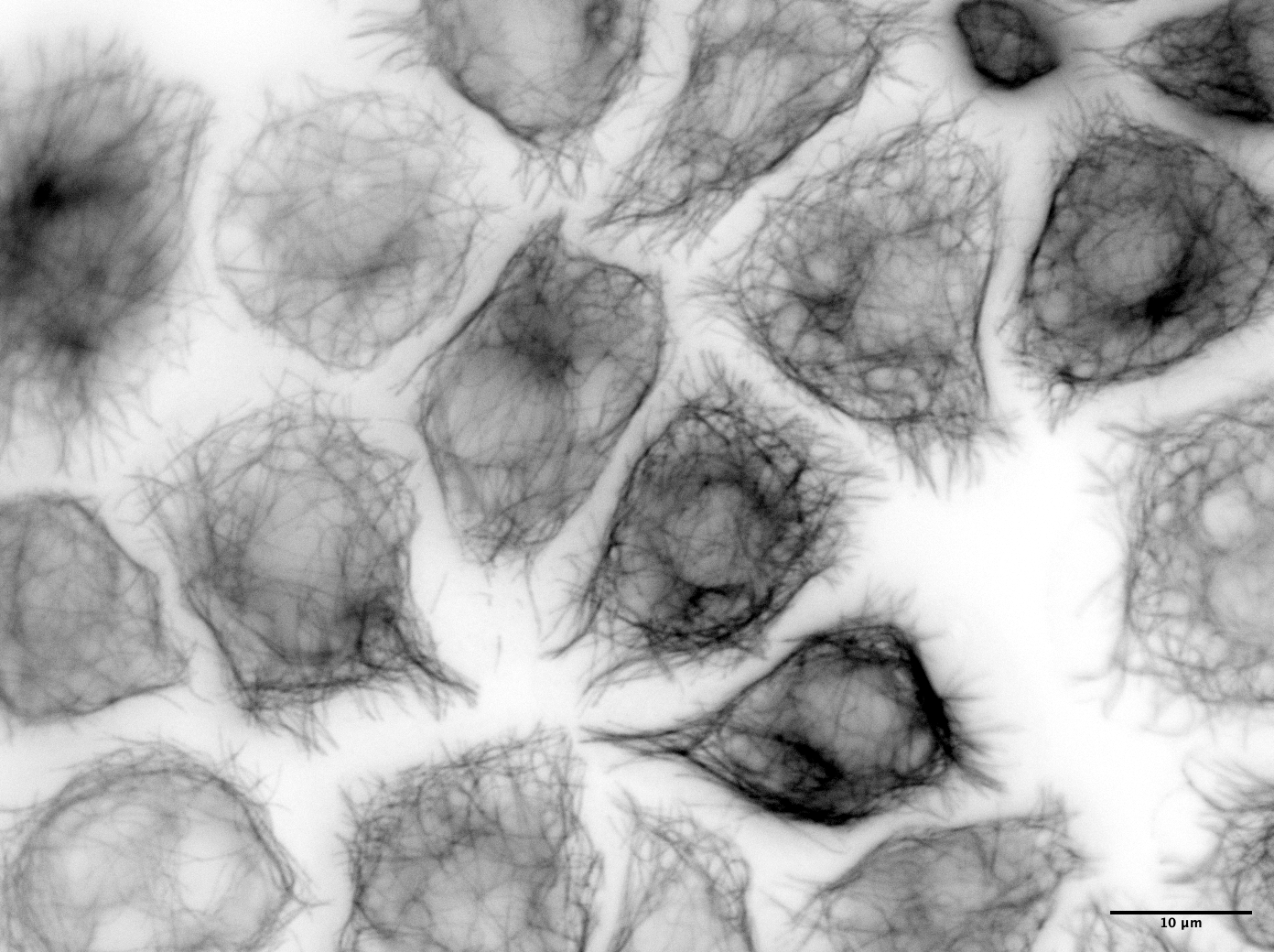

Supplement: Supplementary file 7 — Source Data for Expanded View and Appendix [file EMBR-24-e57264-s003.zip › EMBOR-2023-57264V1_SourceDataForExpandedViewAndAppendix/Appendix/Appendix_Figure_S3/B/dTAT_KO_006_a-tub_577-1622_scale.png]

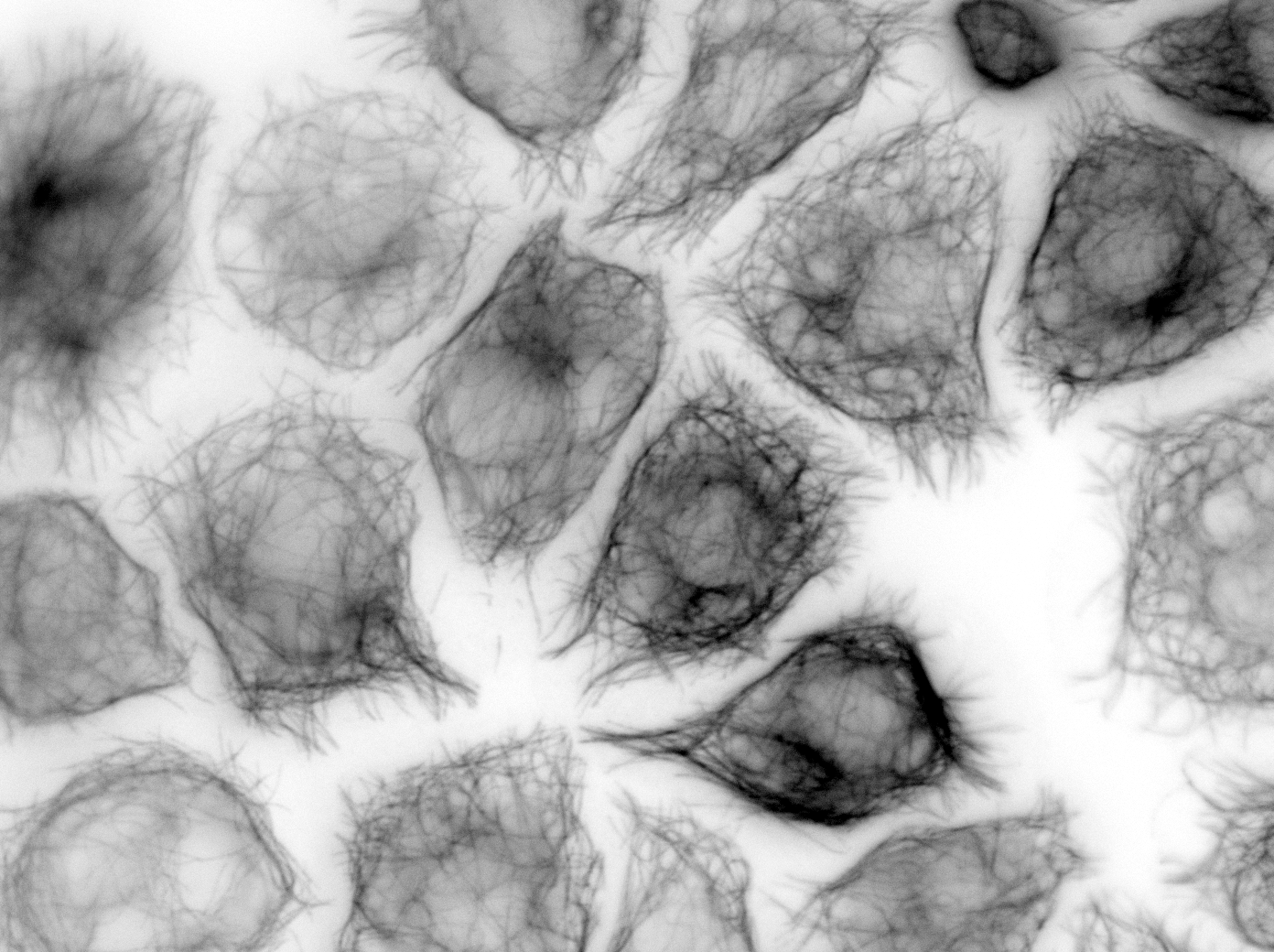

Supplement: Supplementary file 7 — Source Data for Expanded View and Appendix [file EMBR-24-e57264-s003.zip › EMBOR-2023-57264V1_SourceDataForExpandedViewAndAppendix/Appendix/Appendix_Figure_S3/B/dTAT_KO_006_a-tub_577-1622.png]

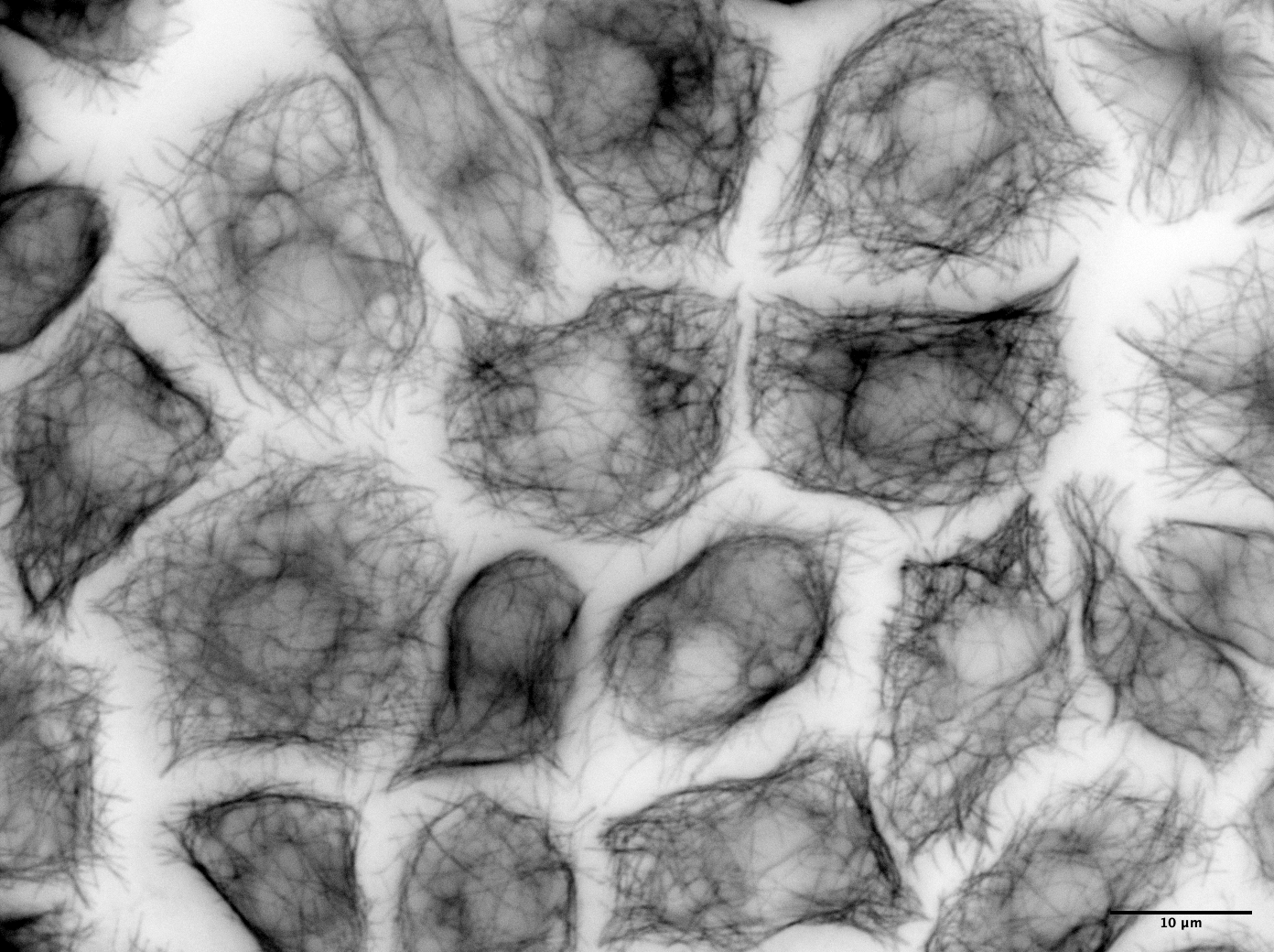

Supplement: Supplementary file 7 — Source Data for Expanded View and Appendix [file EMBR-24-e57264-s003.zip › EMBOR-2023-57264V1_SourceDataForExpandedViewAndAppendix/Appendix/Appendix_Figure_S3/B/WT_005_a-tub_577-1622_scale.png]

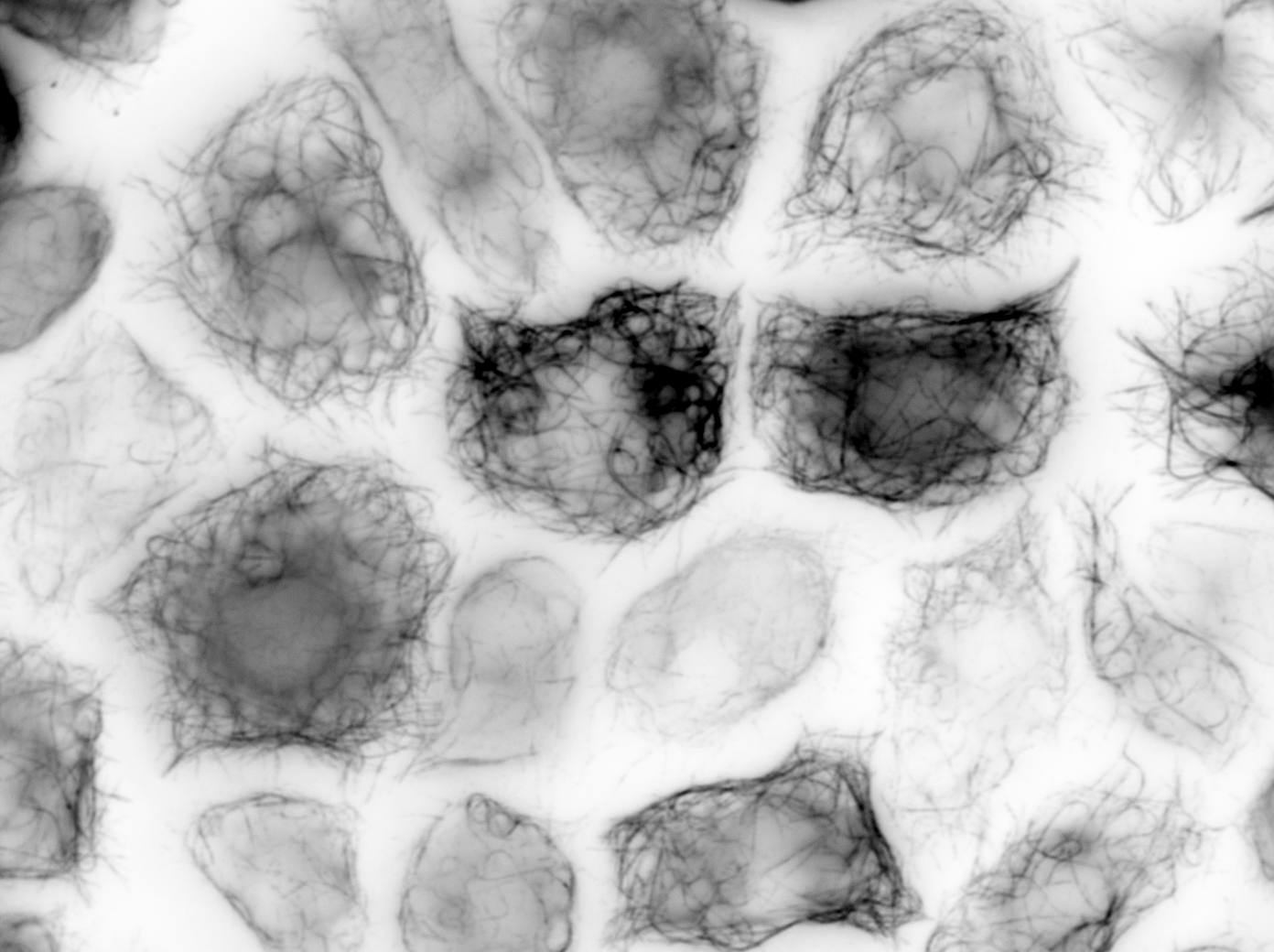

Supplement: Supplementary file 7 — Source Data for Expanded View and Appendix [file EMBR-24-e57264-s003.zip › EMBOR-2023-57264V1_SourceDataForExpandedViewAndAppendix/Appendix/Appendix_Figure_S3/B/WT_005_acet-tub_641-5529.png]

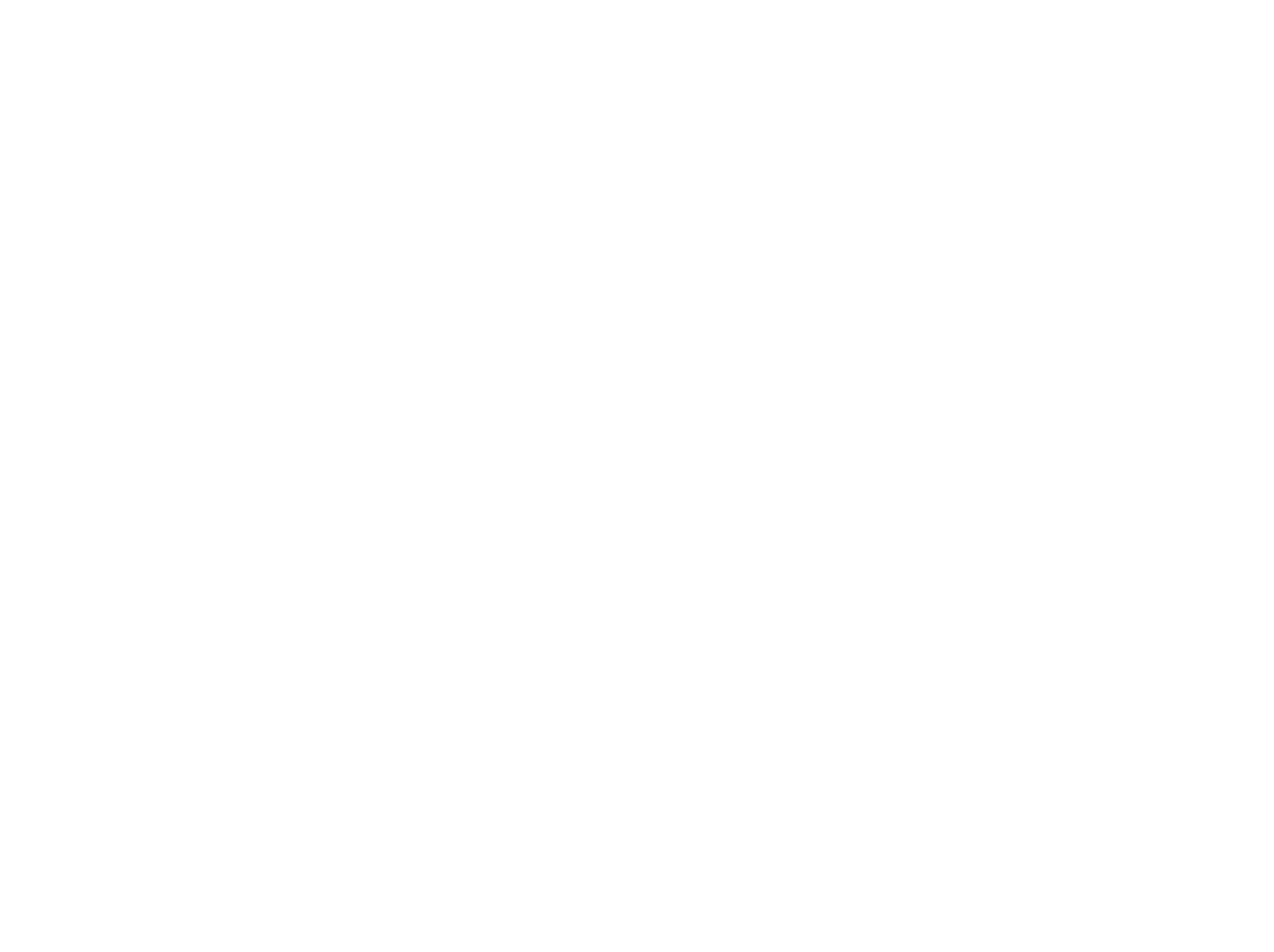

Supplement: Supplementary file 7 — Source Data for Expanded View and Appendix [file EMBR-24-e57264-s003.zip › EMBOR-2023-57264V1_SourceDataForExpandedViewAndAppendix/Appendix/Appendix_Figure_S3/B/dTAT_KO_006_acet-tub_641-5529.png]

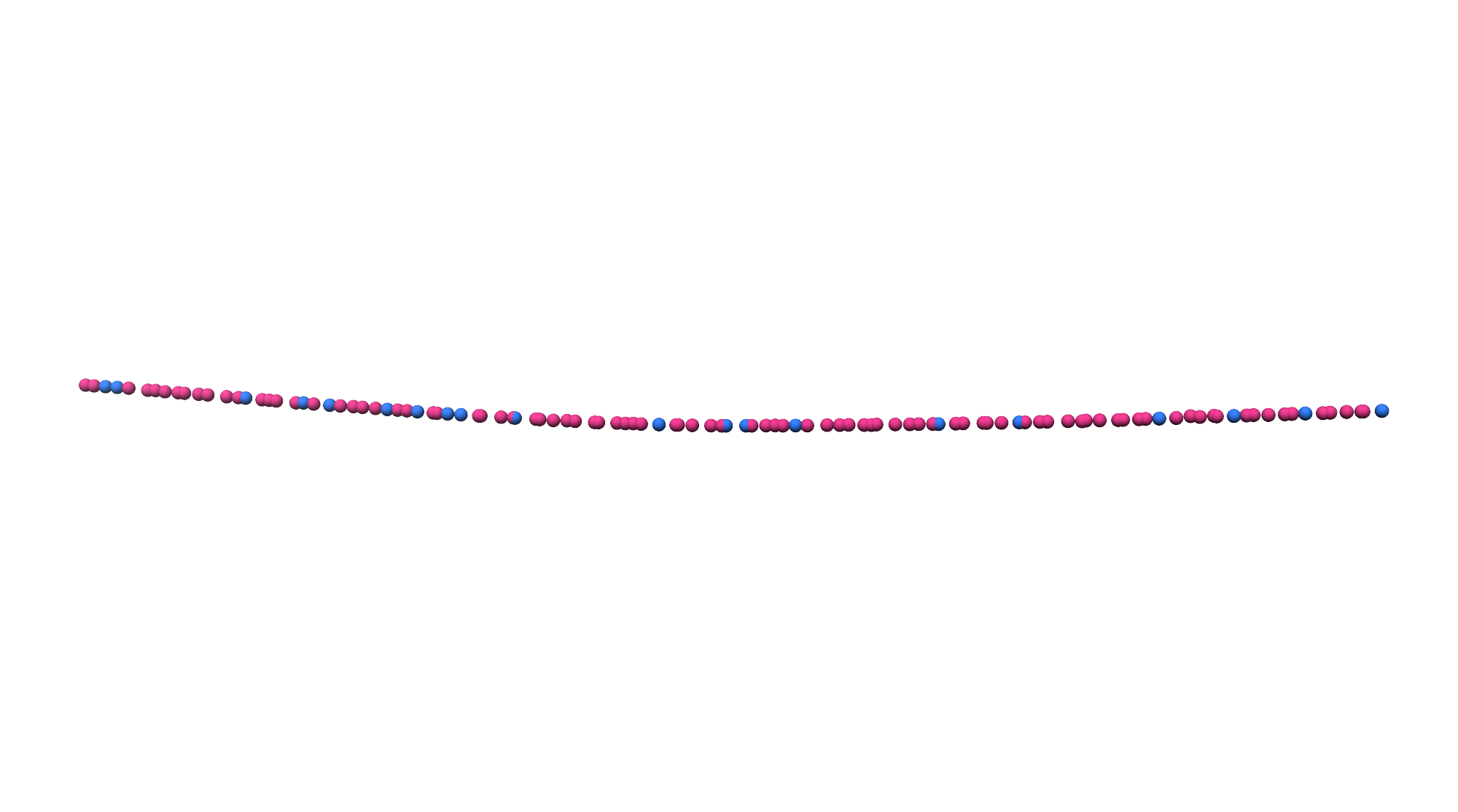

Supplement: Supplementary file 7 — Source Data for Expanded View and Appendix [file EMBR-24-e57264-s003.zip › EMBOR-2023-57264V1_SourceDataForExpandedViewAndAppendix/Appendix/Appendix_Figure_S1/Before_Cleaning/15PF_minus_DZ1_MTmod016_pinkCl7_blueCl8_BeforeClean_230711.png]

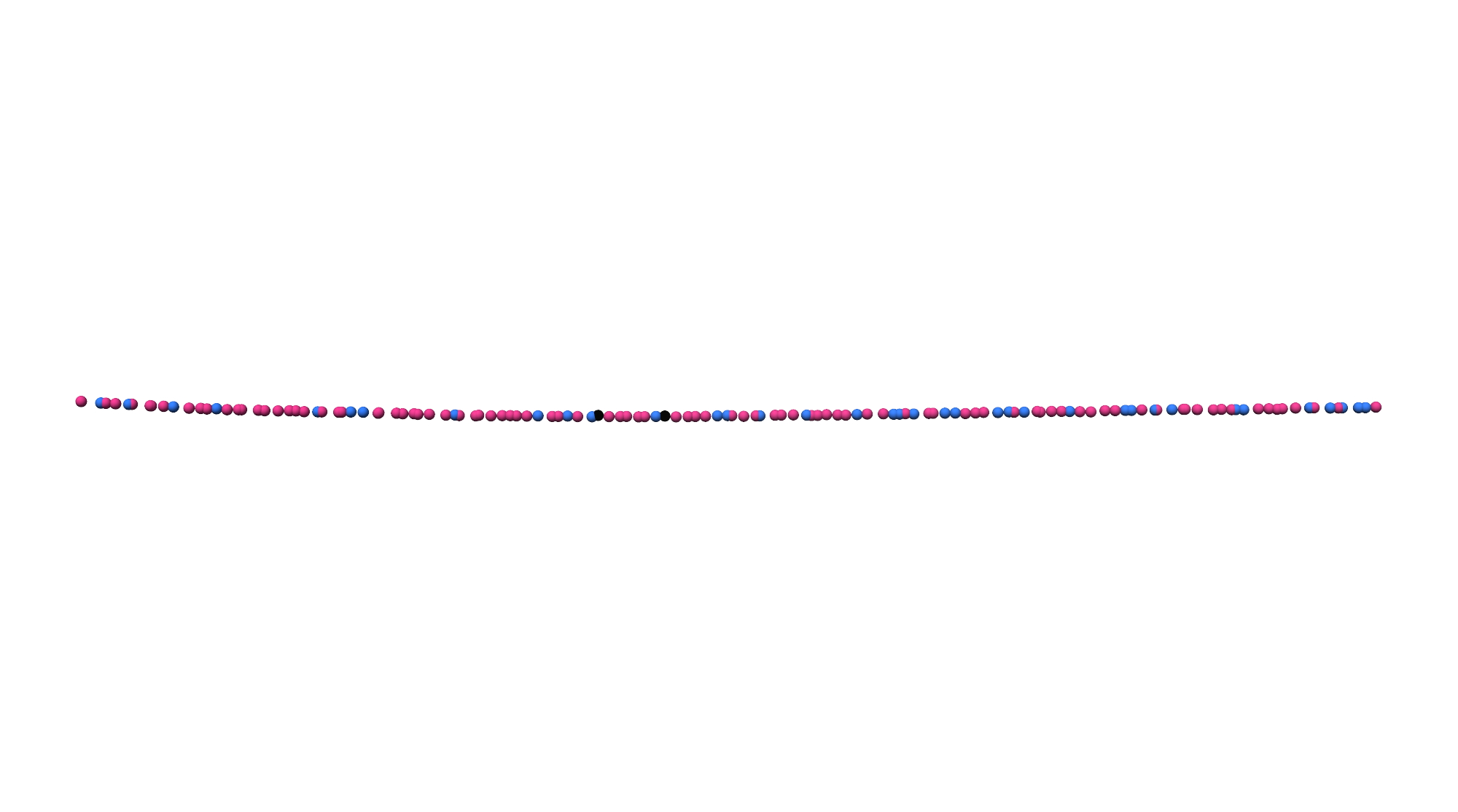

Supplement: Supplementary file 7 — Source Data for Expanded View and Appendix [file EMBR-24-e57264-s003.zip › EMBOR-2023-57264V1_SourceDataForExpandedViewAndAppendix/Appendix/Appendix_Figure_S1/Before_Cleaning/15PF_plus_DZ1_MTmod079_pinkCl8_blueCl7_blackCl6_BeforeClean_230711.png]

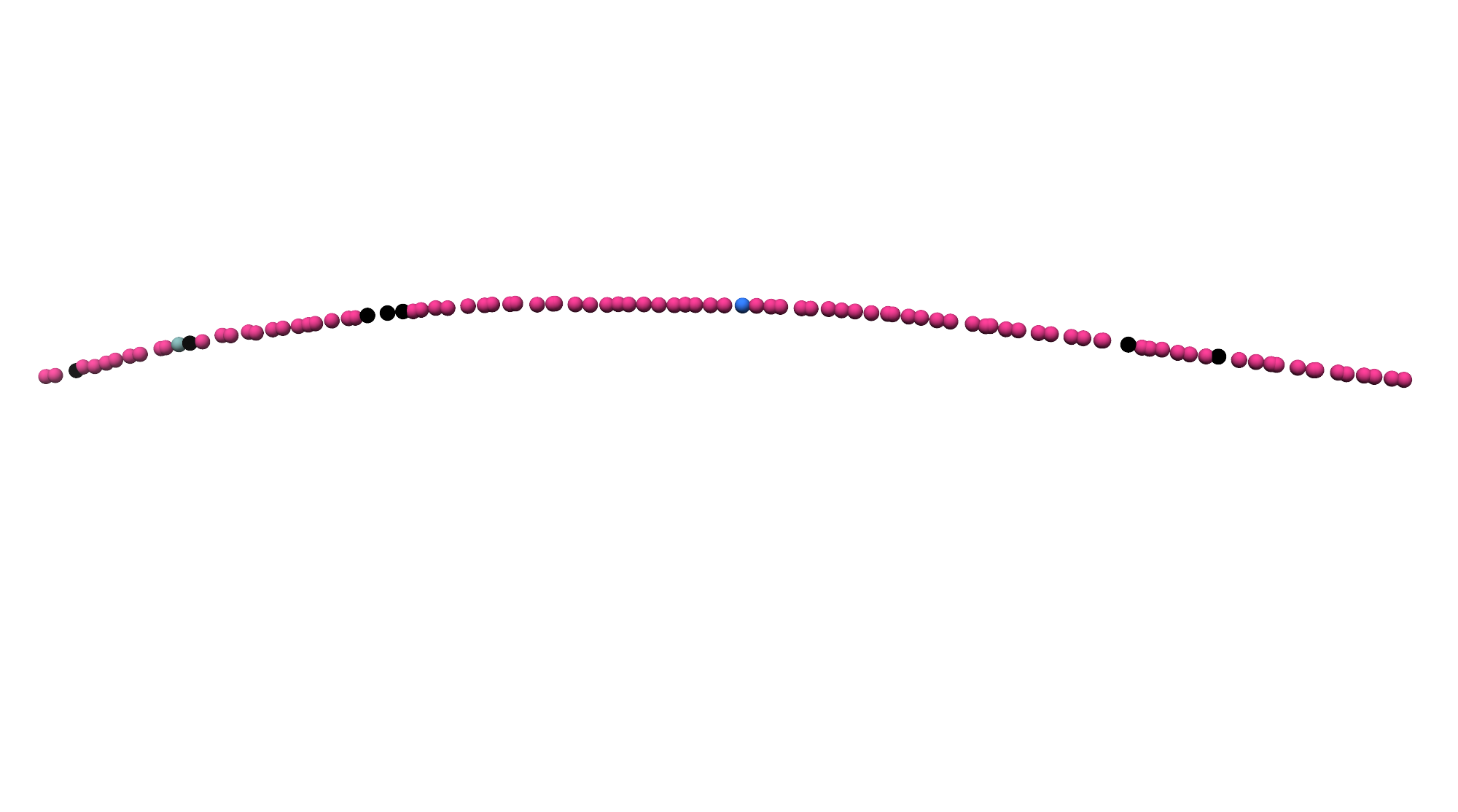

Supplement: Supplementary file 7 — Source Data for Expanded View and Appendix [file EMBR-24-e57264-s003.zip › EMBOR-2023-57264V1_SourceDataForExpandedViewAndAppendix/Appendix/Appendix_Figure_S1/Before_Cleaning/12PF_minus_DZ4_MTmod011_pinkCl1_blueCl2_blackCl3_greenCl4_BeforeClean_230711.png]

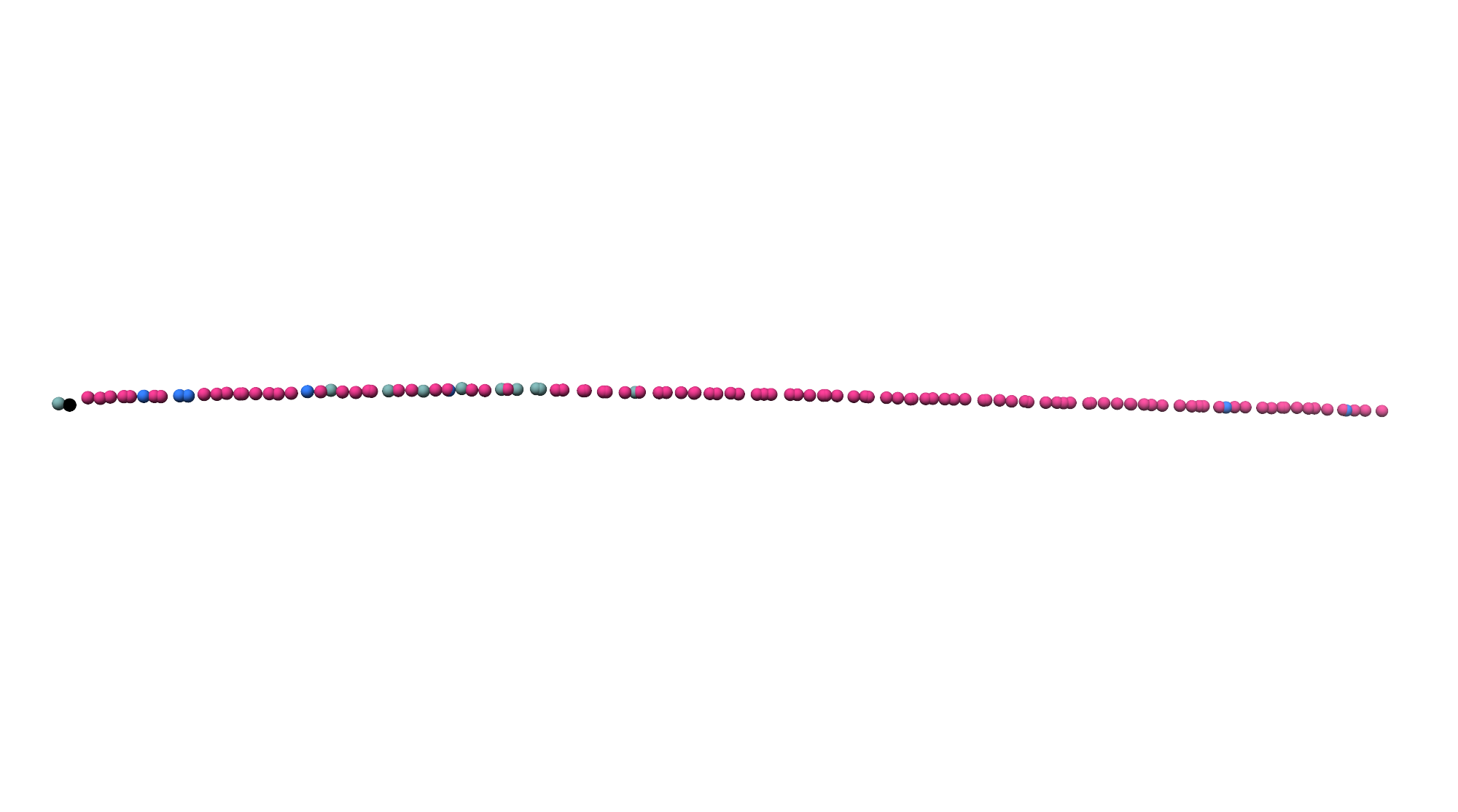

Supplement: Supplementary file 7 — Source Data for Expanded View and Appendix [file EMBR-24-e57264-s003.zip › EMBOR-2023-57264V1_SourceDataForExpandedViewAndAppendix/Appendix/Appendix_Figure_S1/Before_Cleaning/13PF_plus_FB34_MTmod135_pinkCl4_blueCl3_blackCl5_greenCl6_BeforeClean_230711.png]

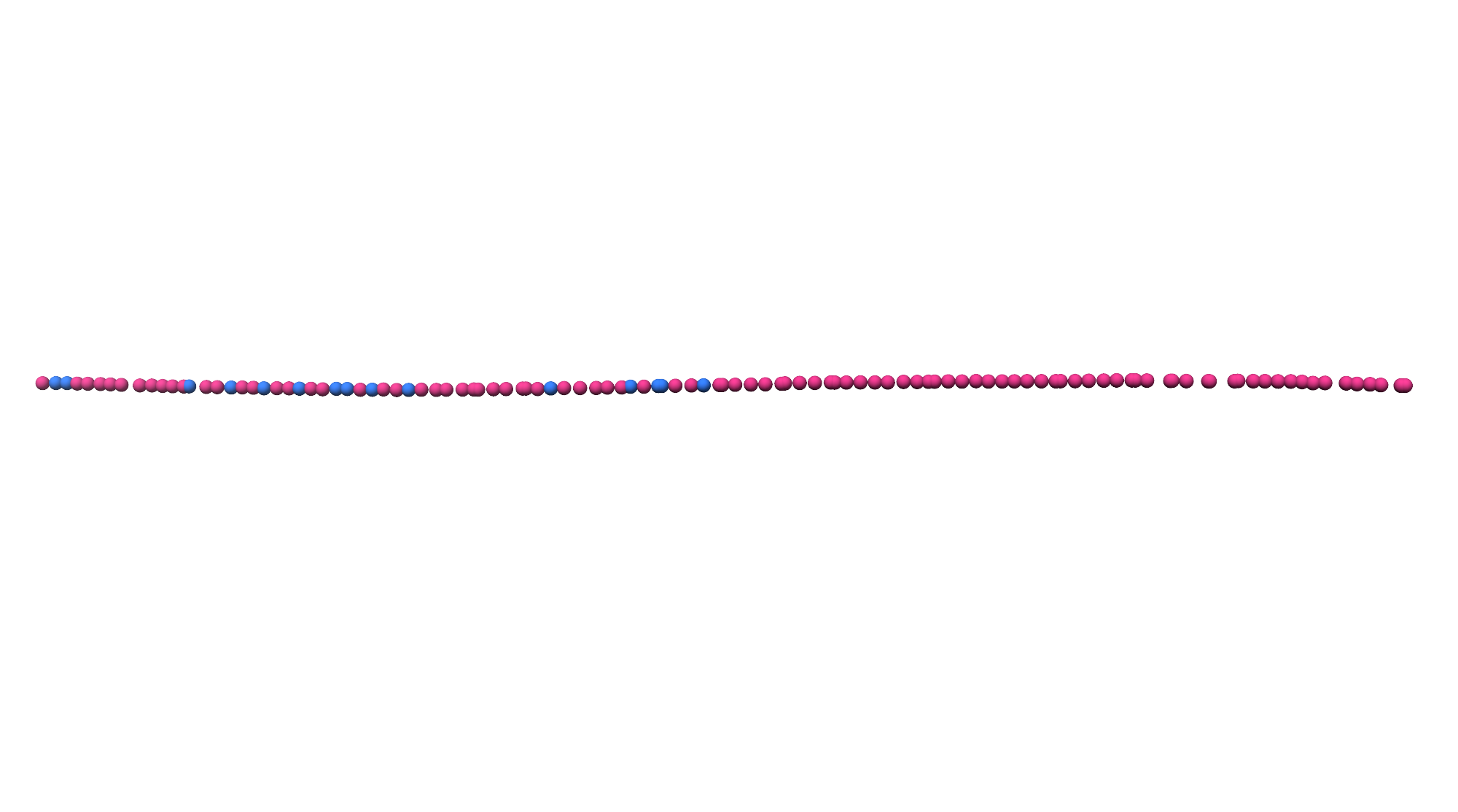

Supplement: Supplementary file 7 — Source Data for Expanded View and Appendix [file EMBR-24-e57264-s003.zip › EMBOR-2023-57264V1_SourceDataForExpandedViewAndAppendix/Appendix/Appendix_Figure_S1/Before_Cleaning/12PF_plus_DZ1_MTmod101_pinkCl2_blueCl1_Before_230711.png]

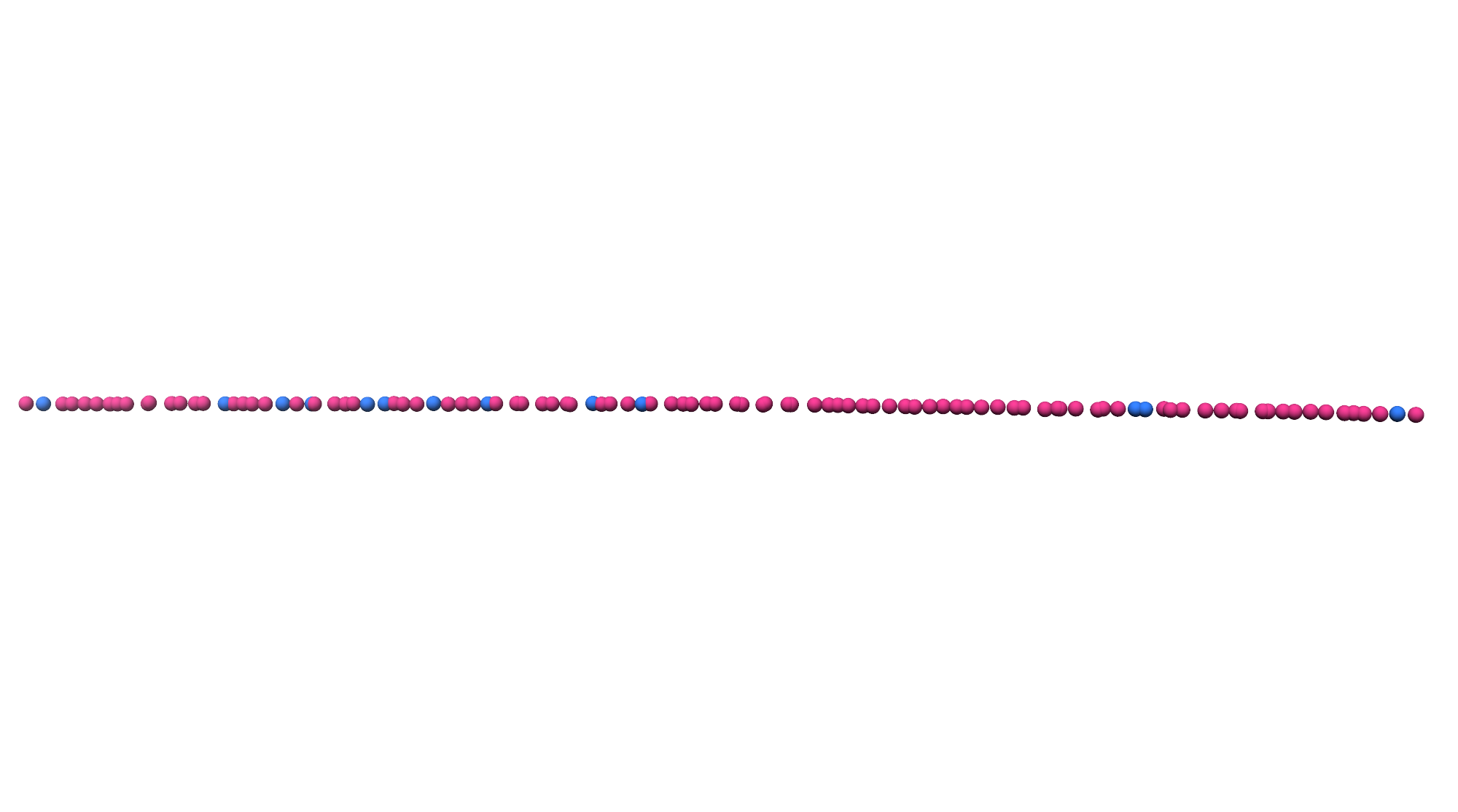

Supplement: Supplementary file 7 — Source Data for Expanded View and Appendix [file EMBR-24-e57264-s003.zip › EMBOR-2023-57264V1_SourceDataForExpandedViewAndAppendix/Appendix/Appendix_Figure_S1/Before_Cleaning/14PF_plus_DZ1_MTmod135_pinkCl6_blueCl5_BeforeClean_230711.png]

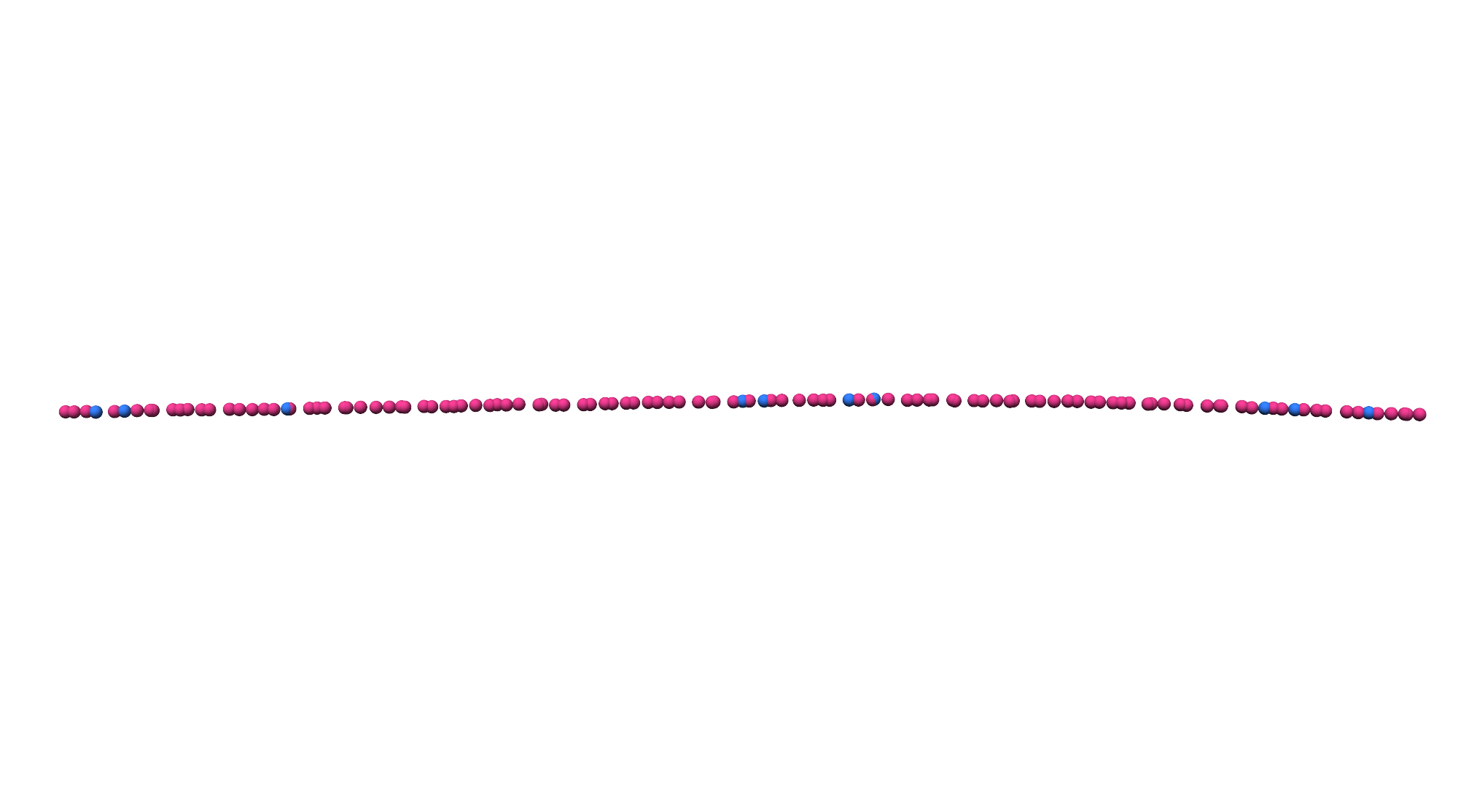

Supplement: Supplementary file 7 — Source Data for Expanded View and Appendix [file EMBR-24-e57264-s003.zip › EMBOR-2023-57264V1_SourceDataForExpandedViewAndAppendix/Appendix/Appendix_Figure_S1/Before_Cleaning/13PF_minus_DZ1_MTmod190_pinkCl3_blueCl4_BeforeClean_230711.png]

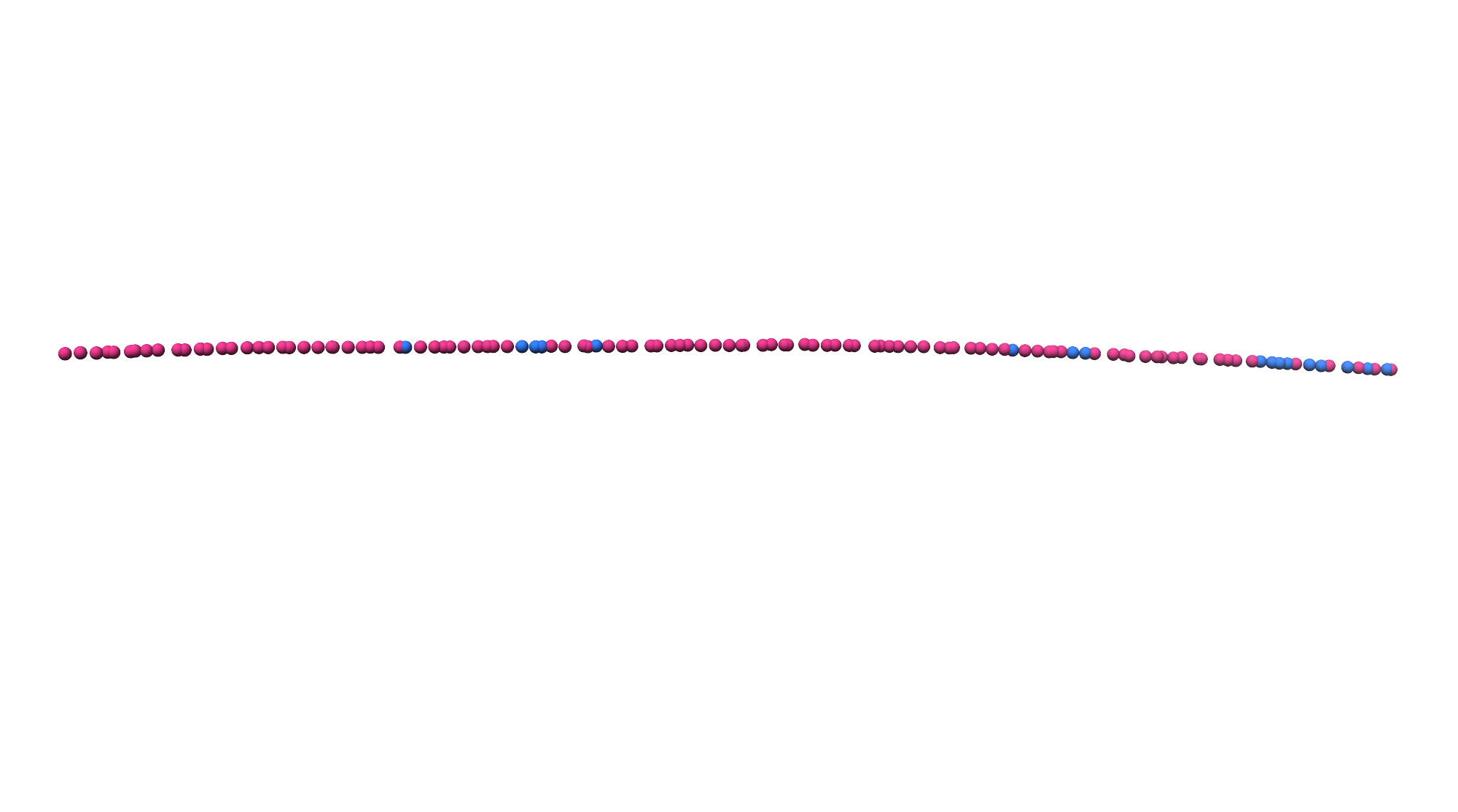

Supplement: Supplementary file 7 — Source Data for Expanded View and Appendix [file EMBR-24-e57264-s003.zip › EMBOR-2023-57264V1_SourceDataForExpandedViewAndAppendix/Appendix/Appendix_Figure_S1/Before_Cleaning/14PF_minus_DZ1_MTmod142_pinkCl5_blueCl6_BeforeClean_230711.png]

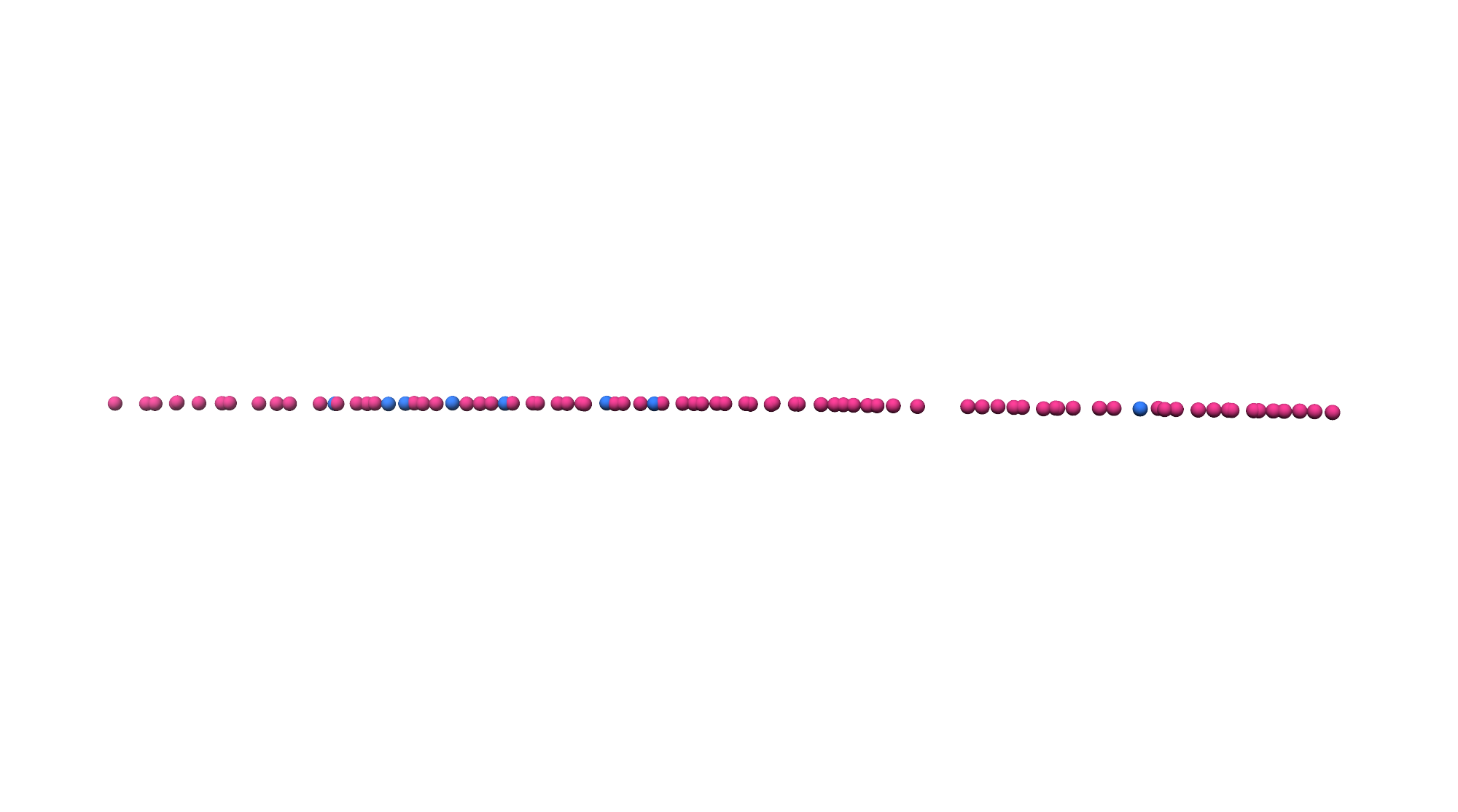

Supplement: Supplementary file 7 — Source Data for Expanded View and Appendix [file EMBR-24-e57264-s003.zip › EMBOR-2023-57264V1_SourceDataForExpandedViewAndAppendix/Appendix/Appendix_Figure_S1/After_Cleaning/14PF_plus_DZ1_MTmod135_pinkCl6_blueCl5_AterClean_230711.png]

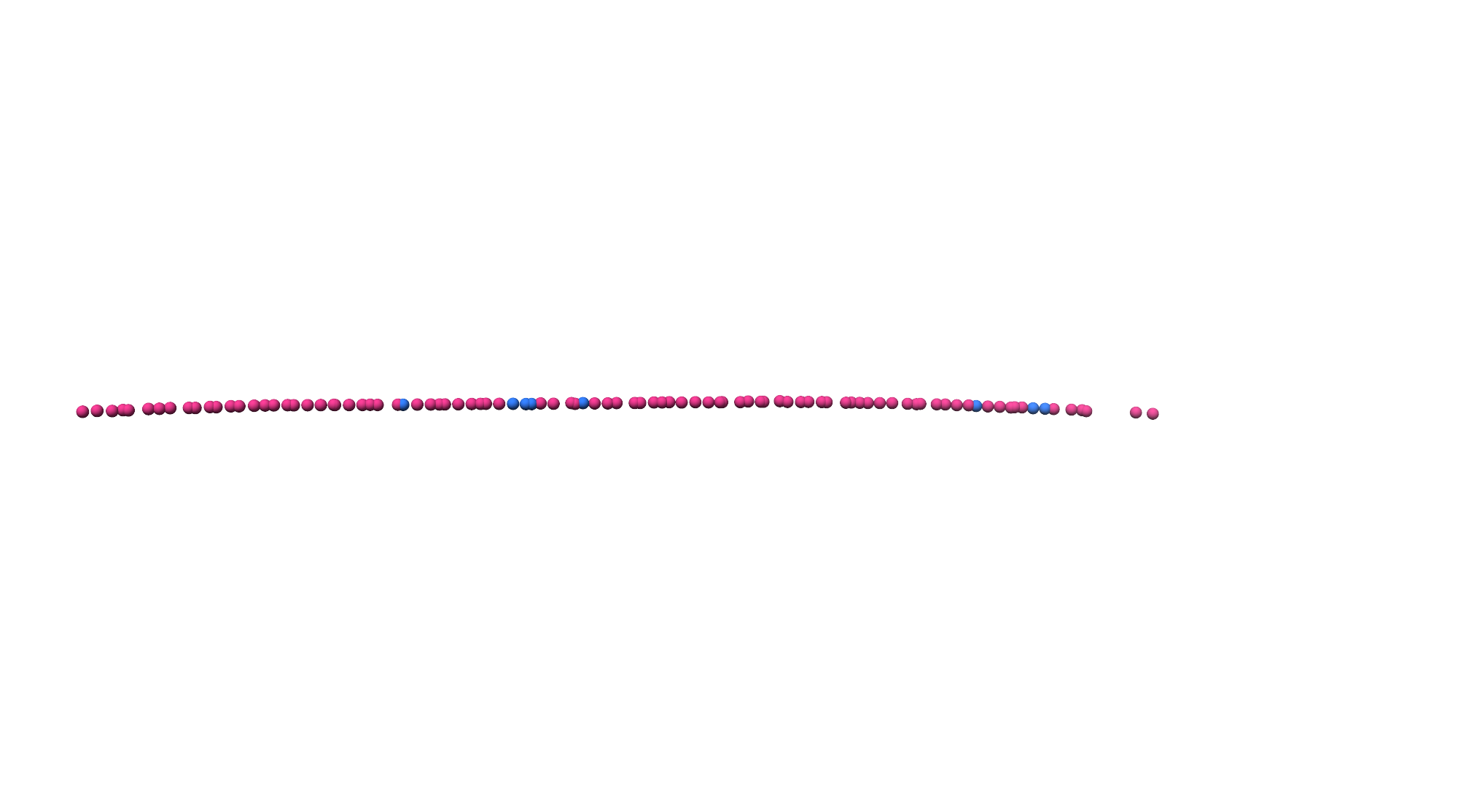

Supplement: Supplementary file 7 — Source Data for Expanded View and Appendix [file EMBR-24-e57264-s003.zip › EMBOR-2023-57264V1_SourceDataForExpandedViewAndAppendix/Appendix/Appendix_Figure_S1/After_Cleaning/14PF_minus_DZ1_MTmod142_pinkCl5_blueCl6_AfterClean_230711.png]

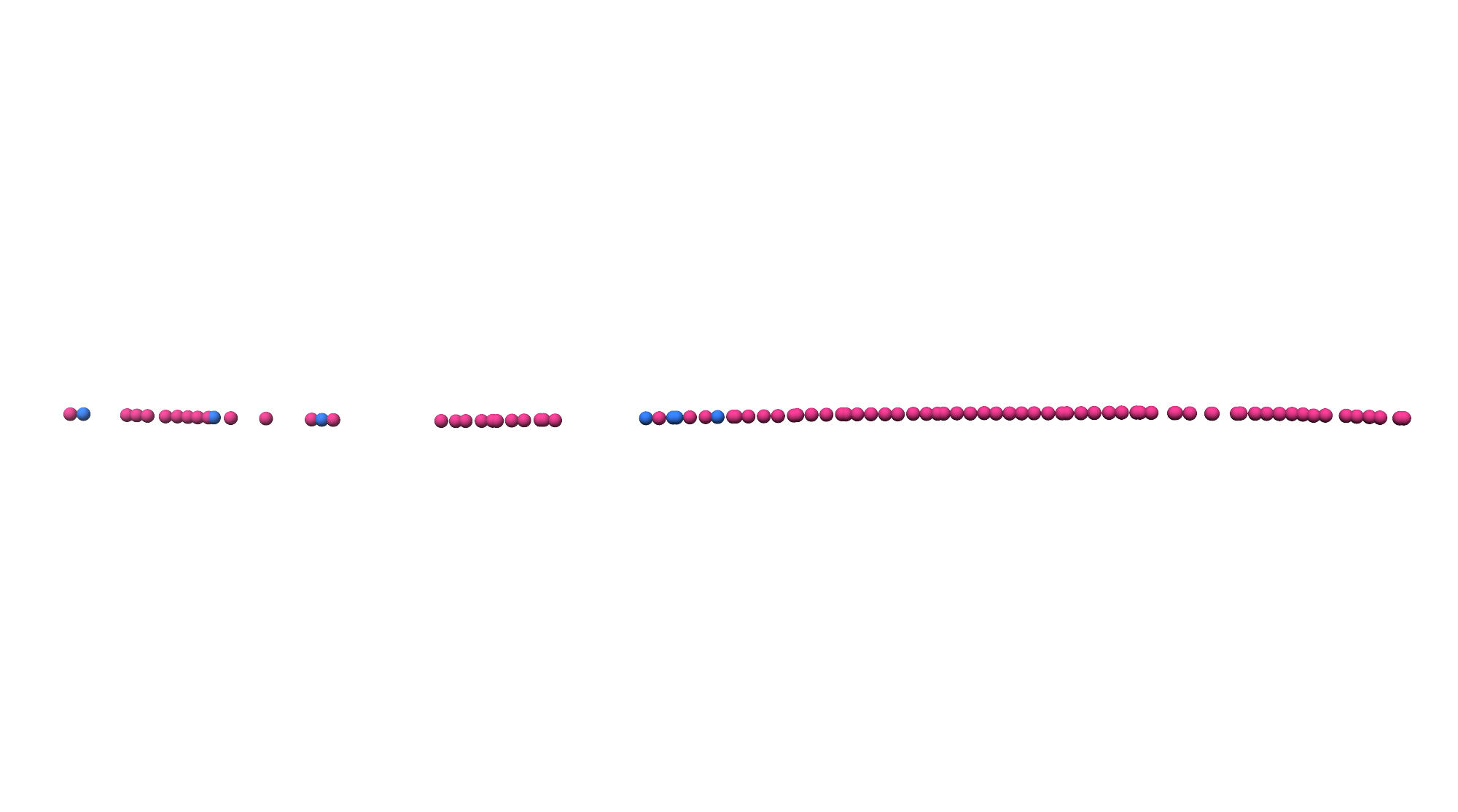

Supplement: Supplementary file 7 — Source Data for Expanded View and Appendix [file EMBR-24-e57264-s003.zip › EMBOR-2023-57264V1_SourceDataForExpandedViewAndAppendix/Appendix/Appendix_Figure_S1/After_Cleaning/12PF_plus_DZ1_MTmod101_pinkCl2_blueCl1_AterClean_230711.png]

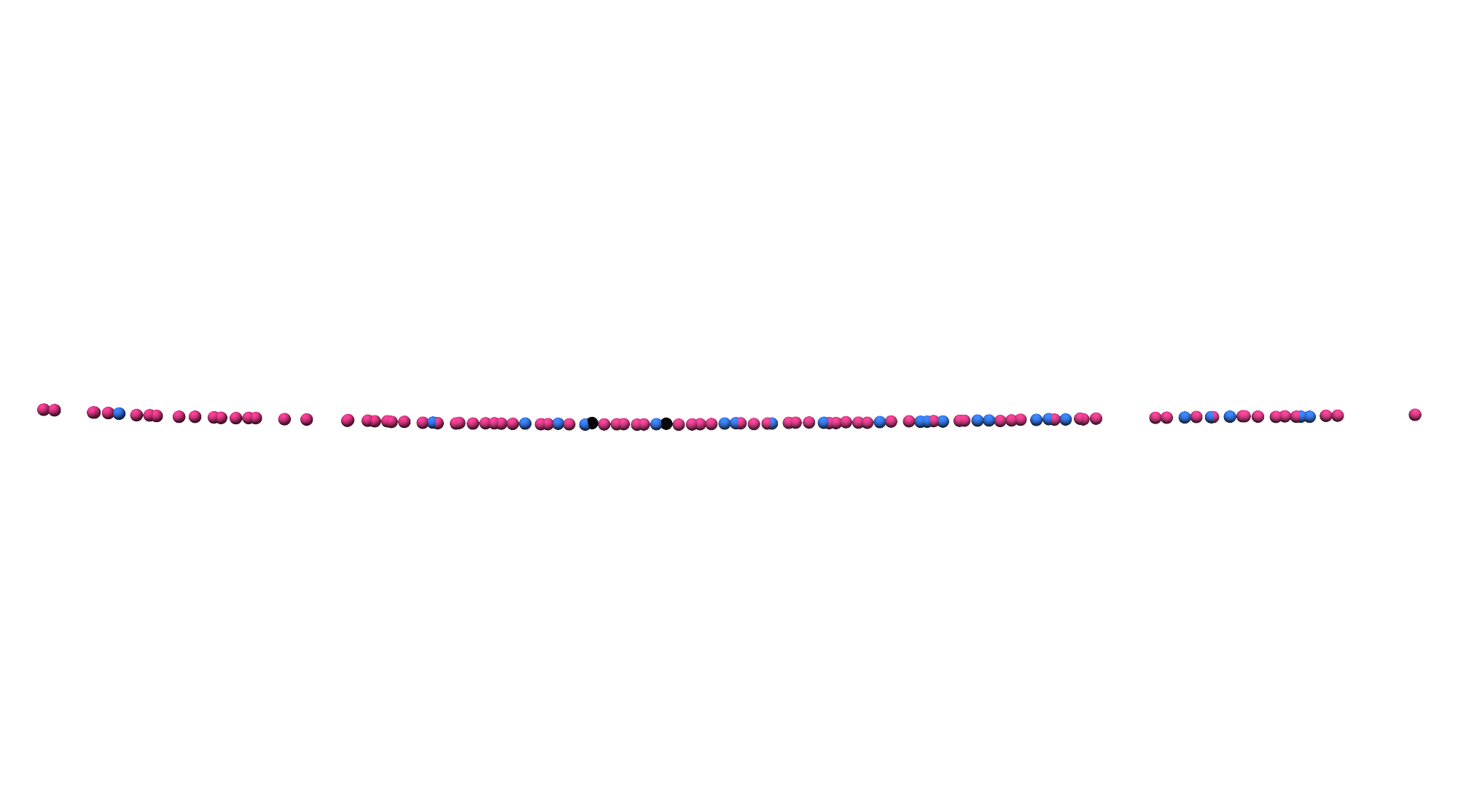

Supplement: Supplementary file 7 — Source Data for Expanded View and Appendix [file EMBR-24-e57264-s003.zip › EMBOR-2023-57264V1_SourceDataForExpandedViewAndAppendix/Appendix/Appendix_Figure_S1/After_Cleaning/15PF_plus_DZ1_MTmod079_pinkCl8_blueCl7_blackCl6_AterClean_230711.png]

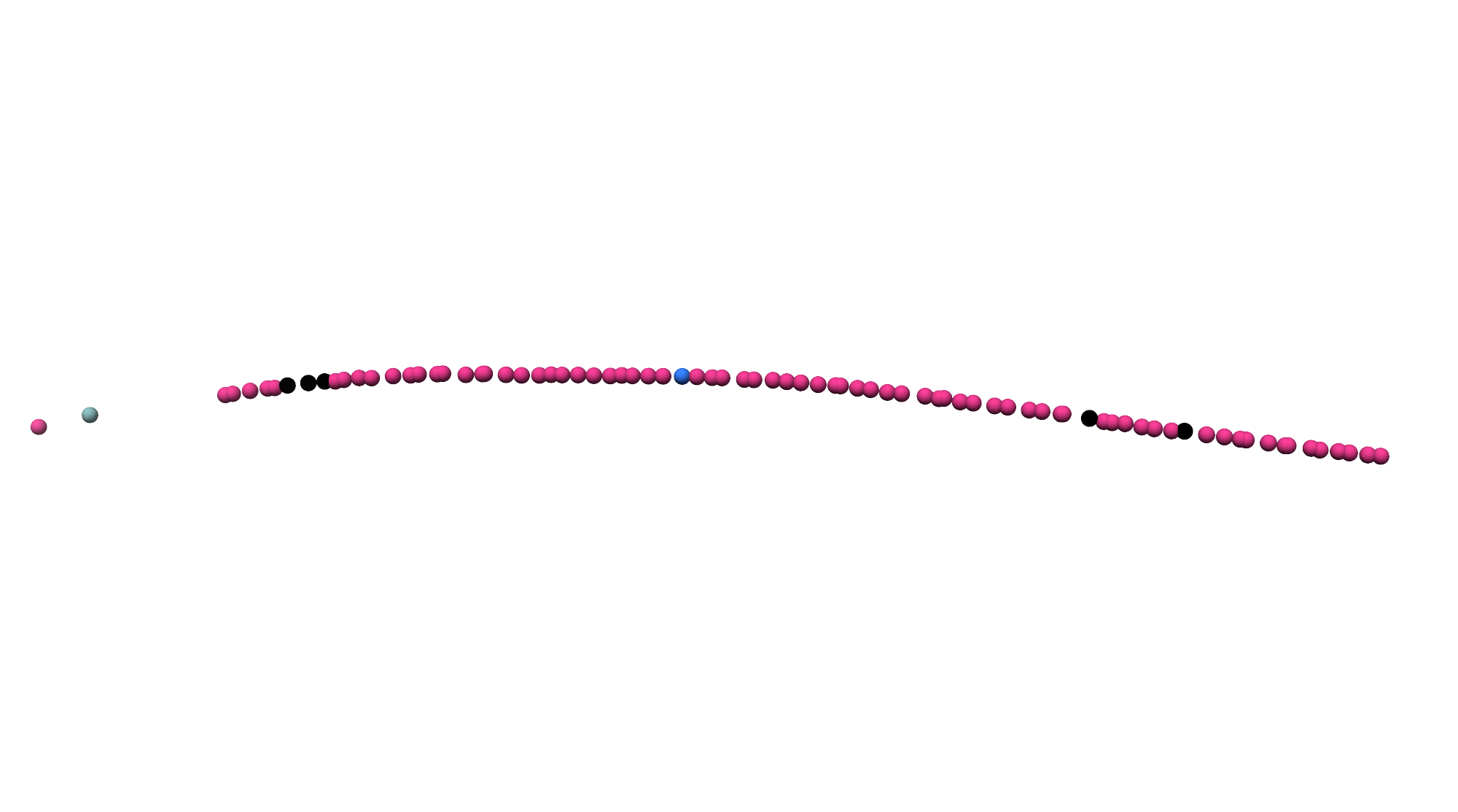

Supplement: Supplementary file 7 — Source Data for Expanded View and Appendix [file EMBR-24-e57264-s003.zip › EMBOR-2023-57264V1_SourceDataForExpandedViewAndAppendix/Appendix/Appendix_Figure_S1/After_Cleaning/12PF_minus_DZ4_MTmod011_pinkCl1_blueCl2_blackCl3_AfterClean_230711.png]

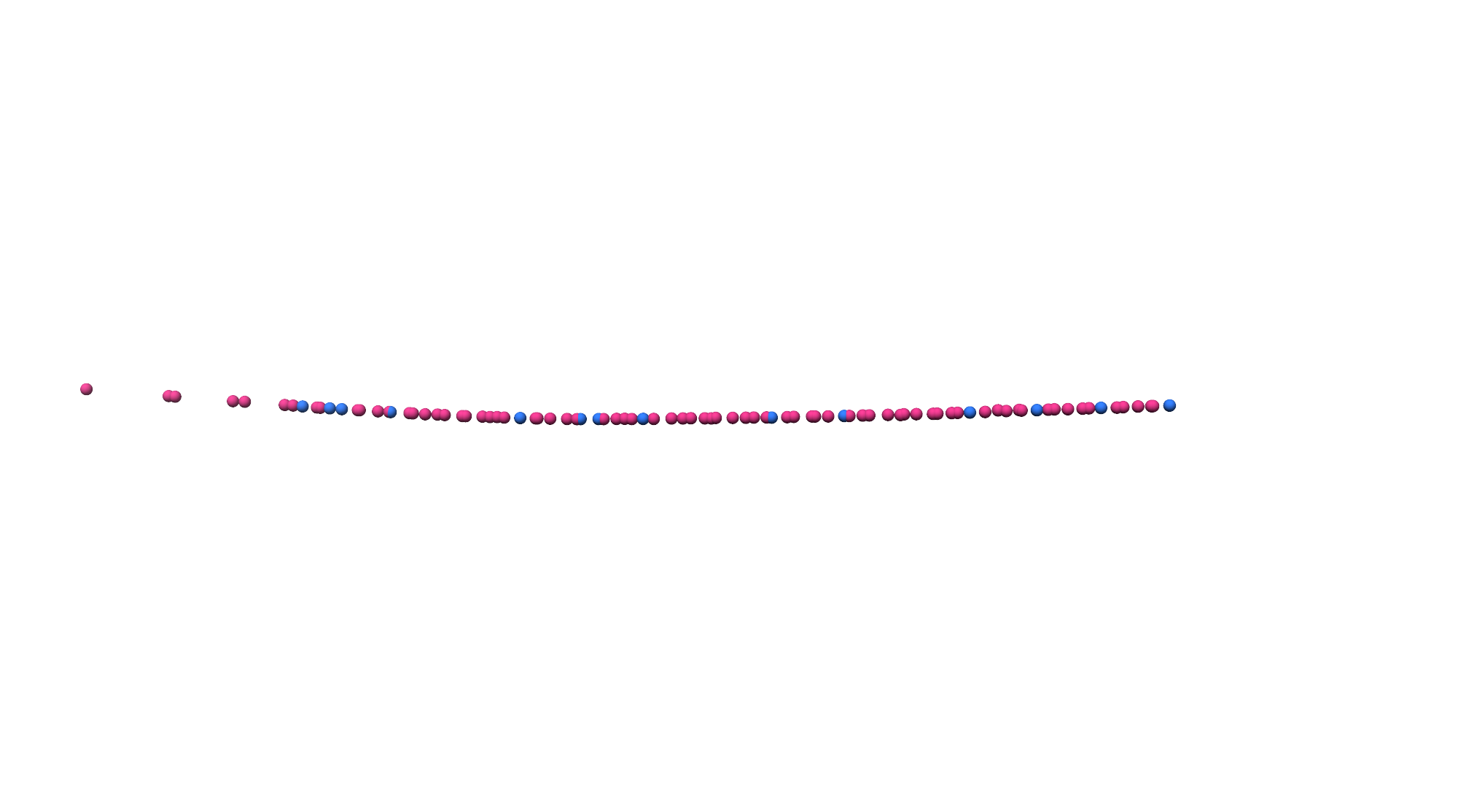

Supplement: Supplementary file 7 — Source Data for Expanded View and Appendix [file EMBR-24-e57264-s003.zip › EMBOR-2023-57264V1_SourceDataForExpandedViewAndAppendix/Appendix/Appendix_Figure_S1/After_Cleaning/15PF_minus_DZ1_MTmod016_pinkCl7_blueCl8_AfterClean_230711.png]

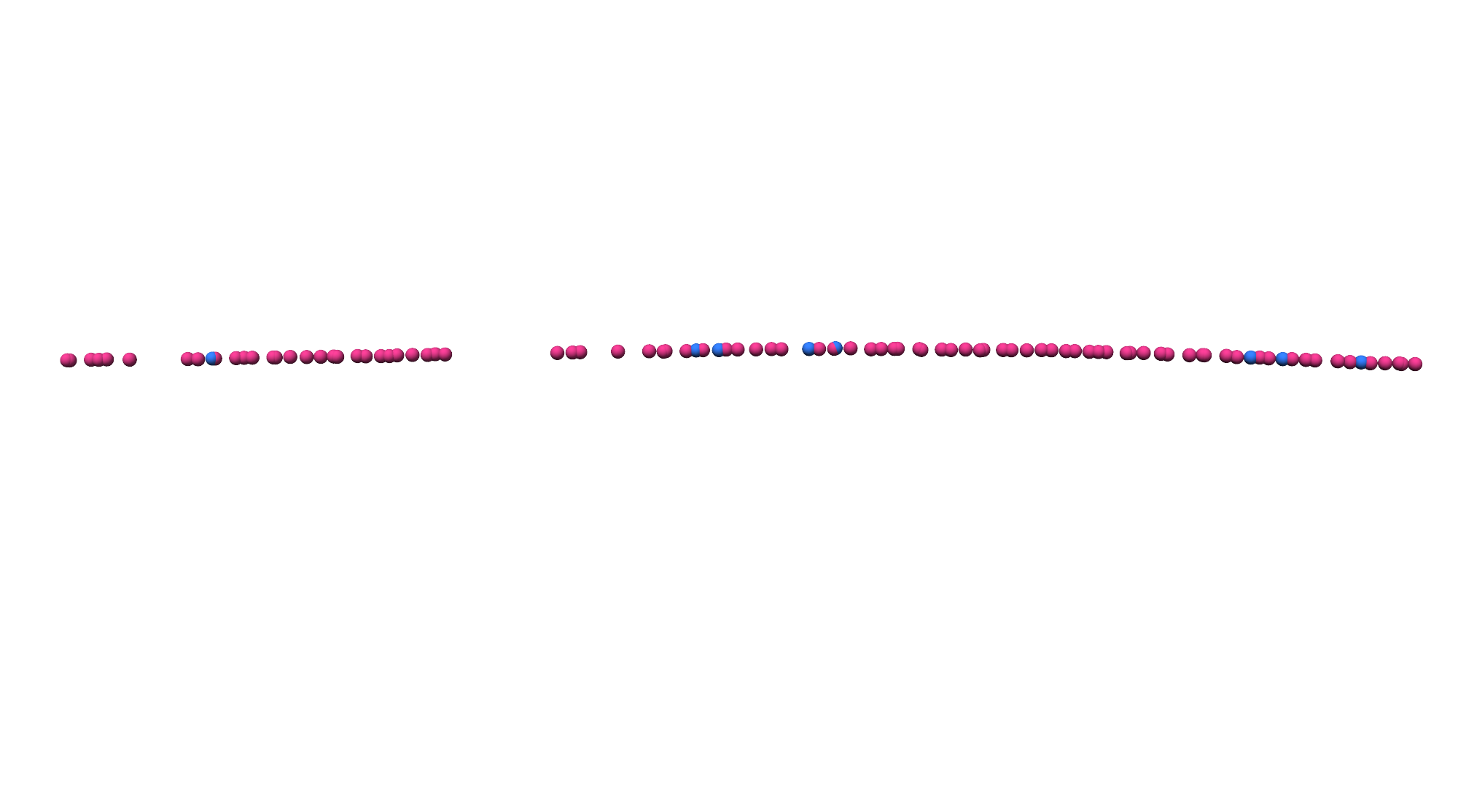

Supplement: Supplementary file 7 — Source Data for Expanded View and Appendix [file EMBR-24-e57264-s003.zip › EMBOR-2023-57264V1_SourceDataForExpandedViewAndAppendix/Appendix/Appendix_Figure_S1/After_Cleaning/13PF_minus_DZ1_MTmod190_pinkCl3_blueCl4_AfterClean_230711.png]

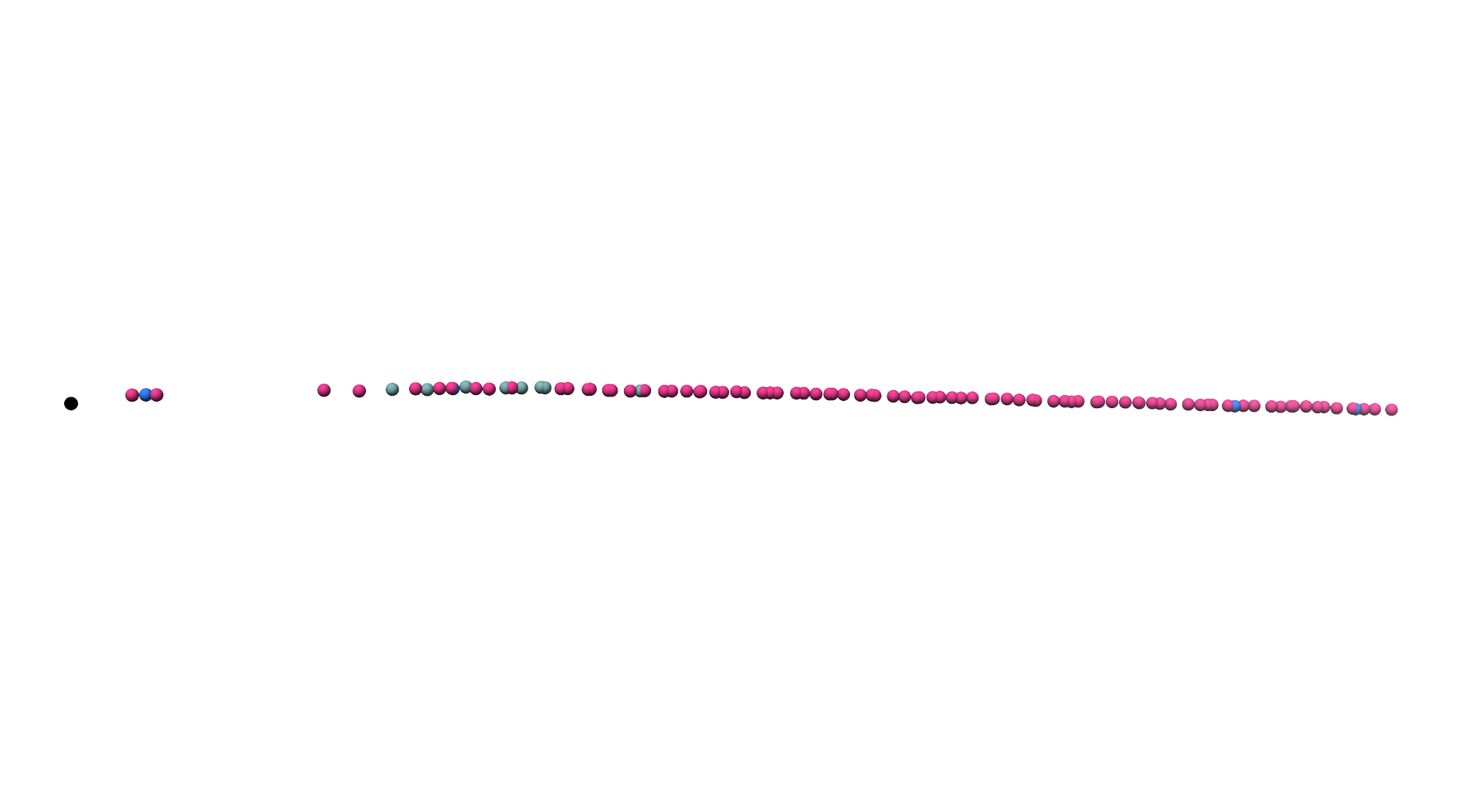

Supplement: Supplementary file 7 — Source Data for Expanded View and Appendix [file EMBR-24-e57264-s003.zip › EMBOR-2023-57264V1_SourceDataForExpandedViewAndAppendix/Appendix/Appendix_Figure_S1/After_Cleaning/13PF_plus_FB34_MTmod135_pinkCl4_blueCl3_blackCl5_greenCl6_AfterClean_230711.png]

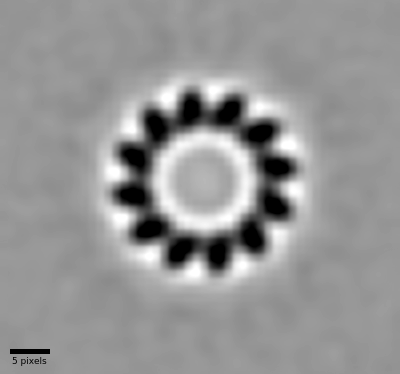

Supplement: Supplementary file 7 — Source Data for Expanded View and Appendix [file EMBR-24-e57264-s003.zip › EMBOR-2023-57264V1_SourceDataForExpandedViewAndAppendix/Figure_EV1/E/microtubule_subtomogramAverage_projections/ref_12PF_minus_11.808Apx.png]

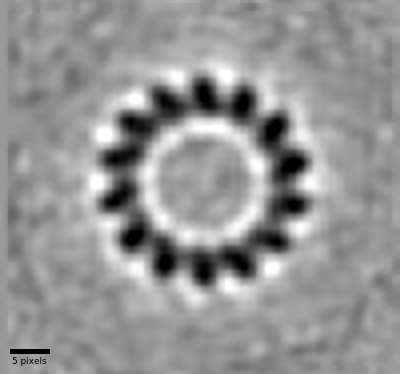

Supplement: Supplementary file 7 — Source Data for Expanded View and Appendix [file EMBR-24-e57264-s003.zip › EMBOR-2023-57264V1_SourceDataForExpandedViewAndAppendix/Figure_EV1/E/microtubule_subtomogramAverage_projections/ref_14PF_plus_11.808Apx.png]

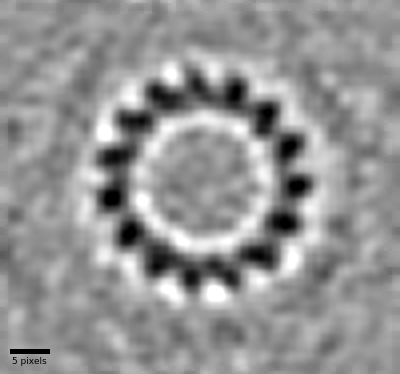

Supplement: Supplementary file 7 — Source Data for Expanded View and Appendix [file EMBR-24-e57264-s003.zip › EMBOR-2023-57264V1_SourceDataForExpandedViewAndAppendix/Figure_EV1/E/microtubule_subtomogramAverage_projections/ref_15PF_plus_11.808Apx.png]

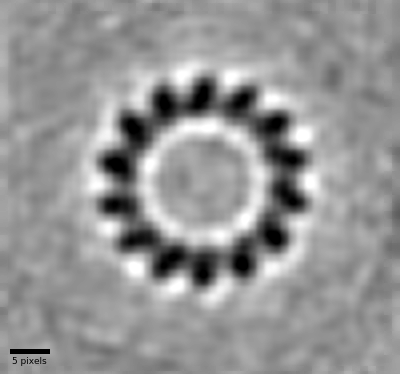

Supplement: Supplementary file 7 — Source Data for Expanded View and Appendix [file EMBR-24-e57264-s003.zip › EMBOR-2023-57264V1_SourceDataForExpandedViewAndAppendix/Figure_EV1/E/microtubule_subtomogramAverage_projections/ref_14PF_minus_11.808Apx.png]

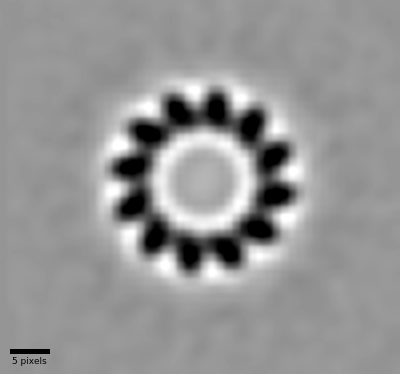

Supplement: Supplementary file 7 — Source Data for Expanded View and Appendix [file EMBR-24-e57264-s003.zip › EMBOR-2023-57264V1_SourceDataForExpandedViewAndAppendix/Figure_EV1/E/microtubule_subtomogramAverage_projections/ref_12PF_plus_11.808Apx.png]

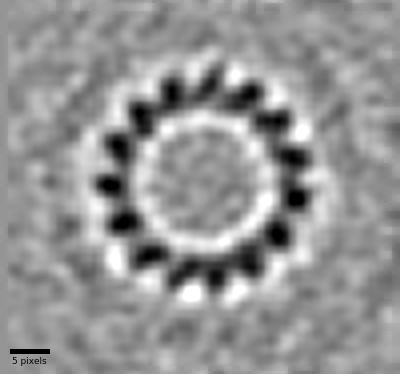

Supplement: Supplementary file 7 — Source Data for Expanded View and Appendix [file EMBR-24-e57264-s003.zip › EMBOR-2023-57264V1_SourceDataForExpandedViewAndAppendix/Figure_EV1/E/microtubule_subtomogramAverage_projections/ref_15PF_minus_11.808Apx.png]

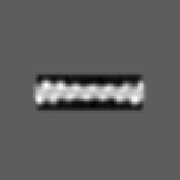

Supplement: Supplementary file 7 — Source Data for Expanded View and Appendix [file EMBR-24-e57264-s003.zip › EMBOR-2023-57264V1_SourceDataForExpandedViewAndAppendix/Figure_EV3/K/FigEV3K_Classification_projectionImages_job091/230324_job091_10Cl_-165_class009.png]

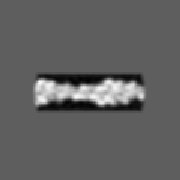

Supplement: Supplementary file 7 — Source Data for Expanded View and Appendix [file EMBR-24-e57264-s003.zip › EMBOR-2023-57264V1_SourceDataForExpandedViewAndAppendix/Figure_EV3/K/FigEV3K_Classification_projectionImages_job091/230324_job091_10Cl_-165_class008.png]

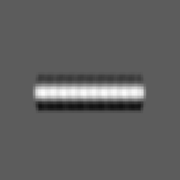

Supplement: Supplementary file 7 — Source Data for Expanded View and Appendix [file EMBR-24-e57264-s003.zip › EMBOR-2023-57264V1_SourceDataForExpandedViewAndAppendix/Figure_EV3/K/FigEV3K_Classification_projectionImages_job091/230324_job091_10Cl_-165_class005.png]

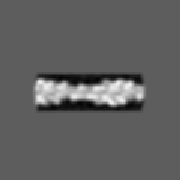

Supplement: Supplementary file 7 — Source Data for Expanded View and Appendix [file EMBR-24-e57264-s003.zip › EMBOR-2023-57264V1_SourceDataForExpandedViewAndAppendix/Figure_EV3/K/FigEV3K_Classification_projectionImages_job091/230324_job091_10Cl_-165_class004.png]

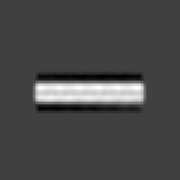

Supplement: Supplementary file 7 — Source Data for Expanded View and Appendix [file EMBR-24-e57264-s003.zip › EMBOR-2023-57264V1_SourceDataForExpandedViewAndAppendix/Figure_EV3/K/FigEV3K_Classification_projectionImages_job091/230324_job091_10Cl_-165_class010.png]

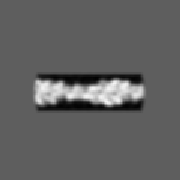

Supplement: Supplementary file 7 — Source Data for Expanded View and Appendix [file EMBR-24-e57264-s003.zip › EMBOR-2023-57264V1_SourceDataForExpandedViewAndAppendix/Figure_EV3/K/FigEV3K_Classification_projectionImages_job091/230324_job091_10Cl_-165_class006.png]

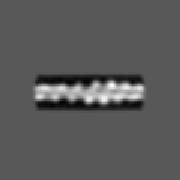

Supplement: Supplementary file 7 — Source Data for Expanded View and Appendix [file EMBR-24-e57264-s003.zip › EMBOR-2023-57264V1_SourceDataForExpandedViewAndAppendix/Figure_EV3/K/FigEV3K_Classification_projectionImages_job091/230324_job091_10Cl_-165_class007.png]

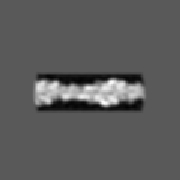

Supplement: Supplementary file 7 — Source Data for Expanded View and Appendix [file EMBR-24-e57264-s003.zip › EMBOR-2023-57264V1_SourceDataForExpandedViewAndAppendix/Figure_EV3/K/FigEV3K_Classification_projectionImages_job091/230324_job091_10Cl_-165_class003.png]

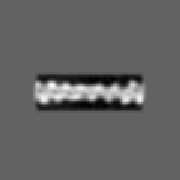

Supplement: Supplementary file 7 — Source Data for Expanded View and Appendix [file EMBR-24-e57264-s003.zip › EMBOR-2023-57264V1_SourceDataForExpandedViewAndAppendix/Figure_EV3/K/FigEV3K_Classification_projectionImages_job091/230324_job091_10Cl_-165_class002.png]

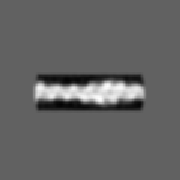

Supplement: Supplementary file 7 — Source Data for Expanded View and Appendix [file EMBR-24-e57264-s003.zip › EMBOR-2023-57264V1_SourceDataForExpandedViewAndAppendix/Figure_EV3/K/FigEV3K_Classification_projectionImages_job091/230324_job091_10Cl_-165_class001.png]

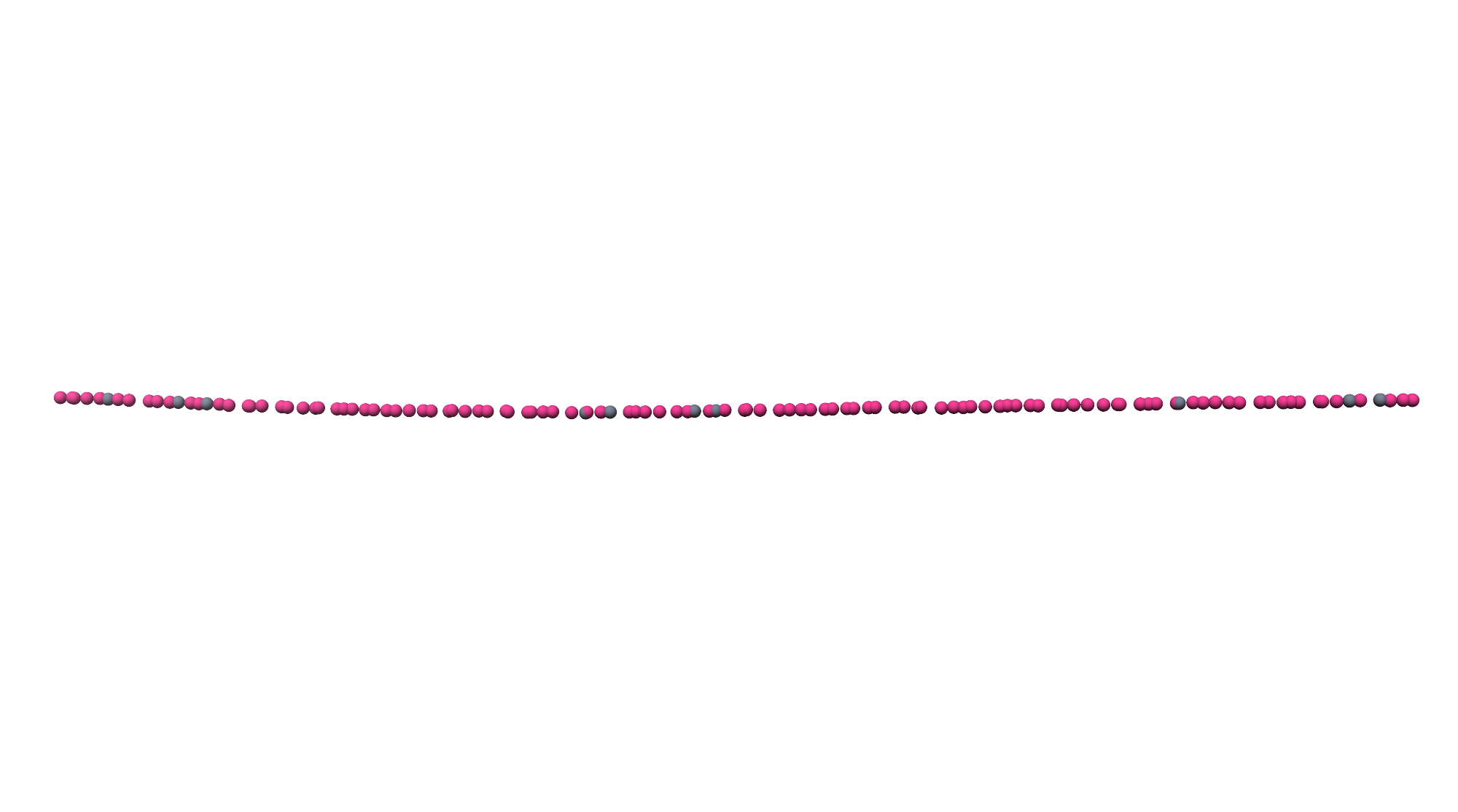

Supplement: Supplementary file 7 — Source Data for Expanded View and Appendix [file EMBR-24-e57264-s003.zip › EMBOR-2023-57264V1_SourceDataForExpandedViewAndAppendix/Figure_EV1/E/ParticlePositions_and_Classes_DZ1_mod190/Before_Cleaning/DZ1_MTmod190_pinkCl3_greyCl4.png]

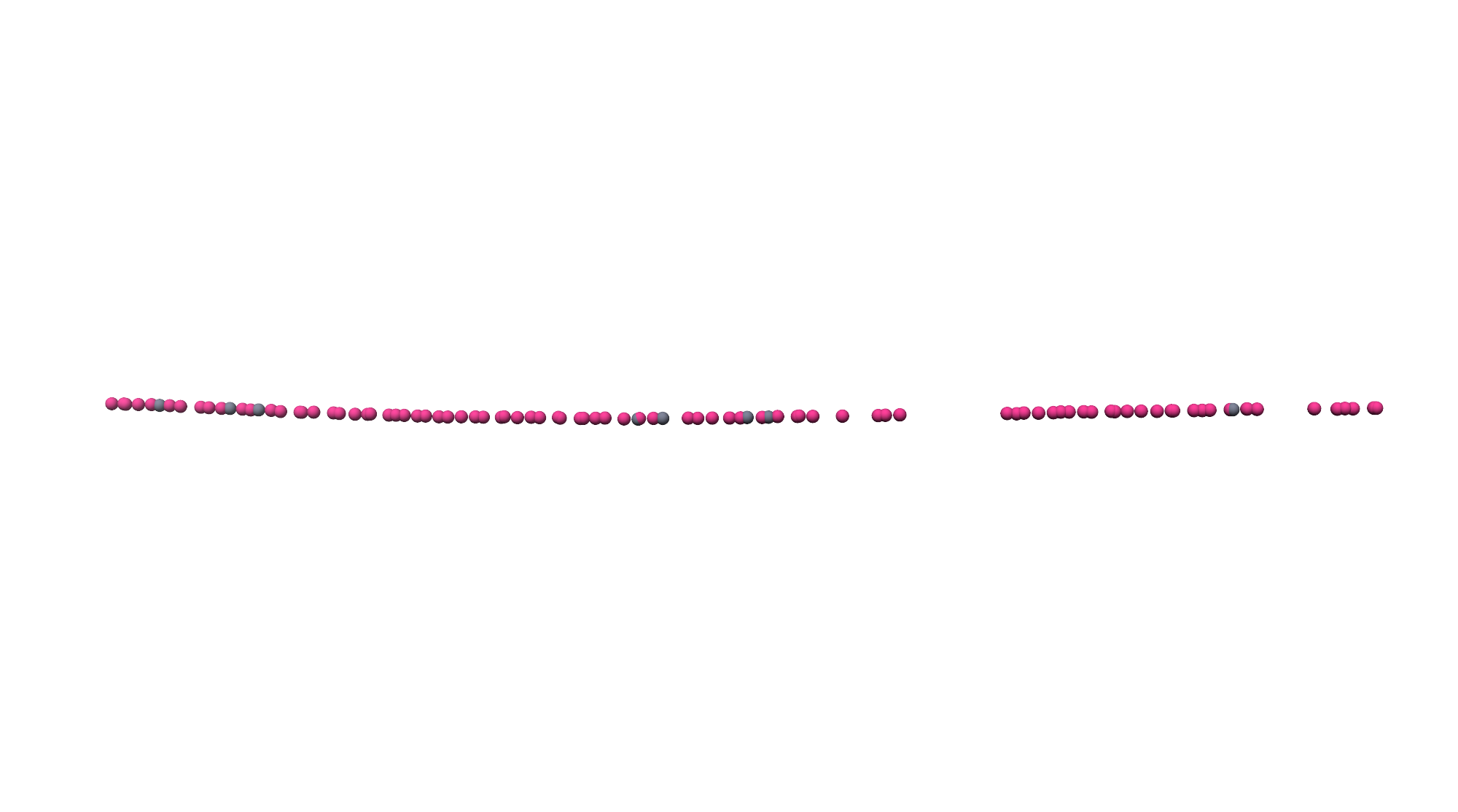

Supplement: Supplementary file 7 — Source Data for Expanded View and Appendix [file EMBR-24-e57264-s003.zip › EMBOR-2023-57264V1_SourceDataForExpandedViewAndAppendix/Figure_EV1/E/ParticlePositions_and_Classes_DZ1_mod190/After_Cleaning/DZ1_MTmod190_pinkCl3_greyCl4_AterClean.png]

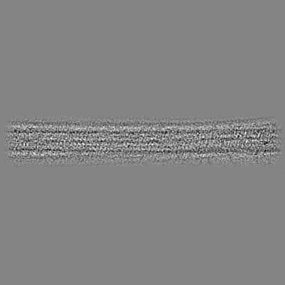

Supplement: Supplementary file 7 — Source Data for Expanded View and Appendix [file EMBR-24-e57264-s003.zip › EMBOR-2023-57264V1_SourceDataForExpandedViewAndAppendix/Figure_EV3/C/LuminalFilaments/FE2_TS_005/TS_005.mrc_11.81Apx_newstack_X548_832_Y331_615_Z48_332_mask_06_project.png]

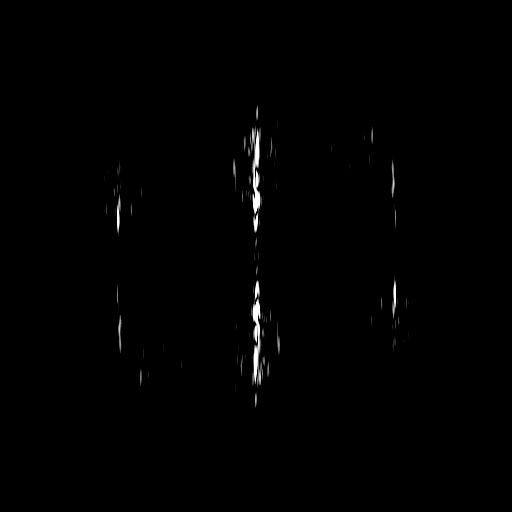

Supplement: Supplementary file 7 — Source Data for Expanded View and Appendix [file EMBR-24-e57264-s003.zip › EMBOR-2023-57264V1_SourceDataForExpandedViewAndAppendix/Figure_EV3/C/LuminalFilaments/FE2_TS_005/TS_005.mrc_11.81Apx_newstack_X548_832_Y331_615_Z48_332_mask_06_project_FFT.png]

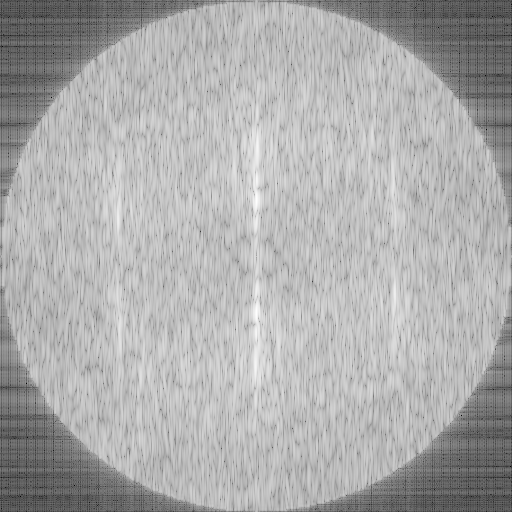

Supplement: Supplementary file 7 — Source Data for Expanded View and Appendix [file EMBR-24-e57264-s003.zip › EMBOR-2023-57264V1_SourceDataForExpandedViewAndAppendix/Figure_EV3/C/LuminalFilaments/FE2_TS_005/TS_005.mrc_11.81Apx_newstack_X548_832_Y331_615_Z48_332_mask_06_project_FFT.tif]

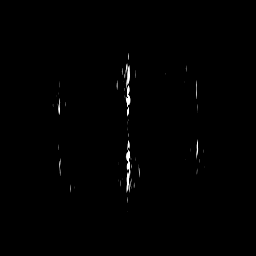

Supplement: Supplementary file 7 — Source Data for Expanded View and Appendix [file EMBR-24-e57264-s003.zip › EMBOR-2023-57264V1_SourceDataForExpandedViewAndAppendix/Figure_EV3/C/LuminalFilaments/FE2_TS_005/TS_005.mrc_11.81Apx_newstack_X548_832_Y331_615_Z48_332_mask_06_project_FFT_binnedBy2.png]

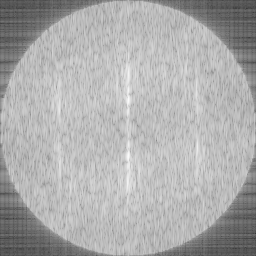

Supplement: Supplementary file 7 — Source Data for Expanded View and Appendix [file EMBR-24-e57264-s003.zip › EMBOR-2023-57264V1_SourceDataForExpandedViewAndAppendix/Figure_EV3/C/LuminalFilaments/FE2_TS_005/TS_005.mrc_11.81Apx_newstack_X548_832_Y331_615_Z48_332_mask_06_project_FFT_binnedBy2.tif]

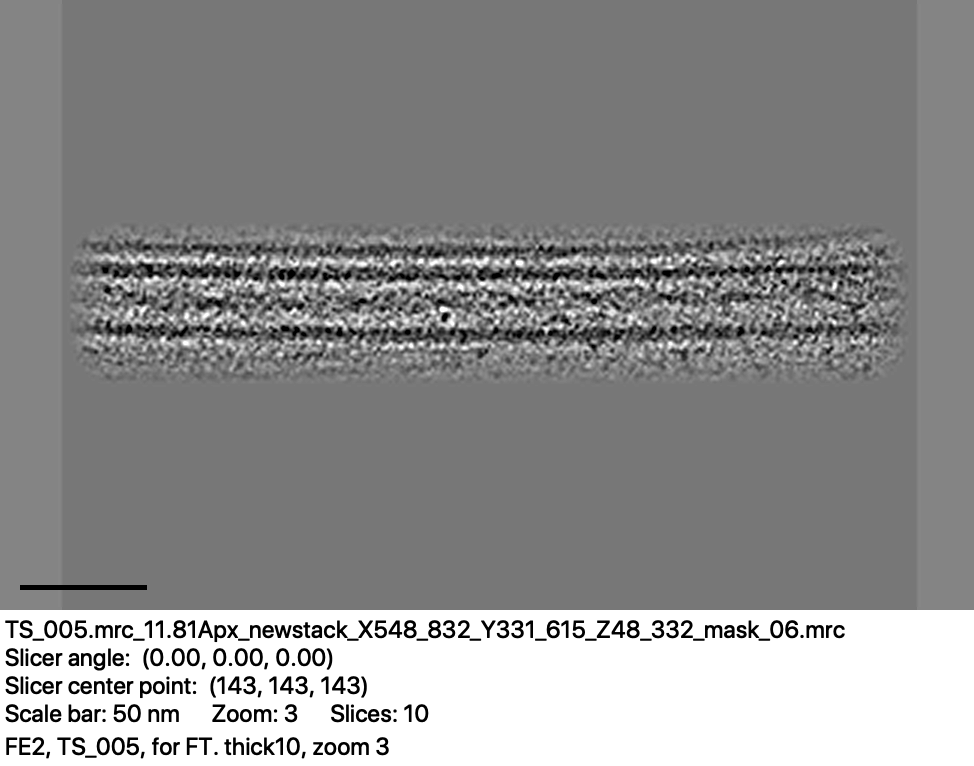

Supplement: Supplementary file 7 — Source Data for Expanded View and Appendix [file EMBR-24-e57264-s003.zip › EMBOR-2023-57264V1_SourceDataForExpandedViewAndAppendix/Figure_EV3/C/LuminalFilaments/FE2_TS_005/FE2_TS_005_masked_forFFT_06_01.png]

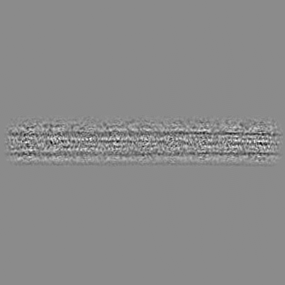

Supplement: Supplementary file 7 — Source Data for Expanded View and Appendix [file EMBR-24-e57264-s003.zip › EMBOR-2023-57264V1_SourceDataForExpandedViewAndAppendix/Figure_EV3/C/LuminalFilaments/TS_328/TS_328.mrc_10.64Apx_bx144_TRIMz_newstack_TRIM_rotx_newboxSHIFT_masked_project.png]

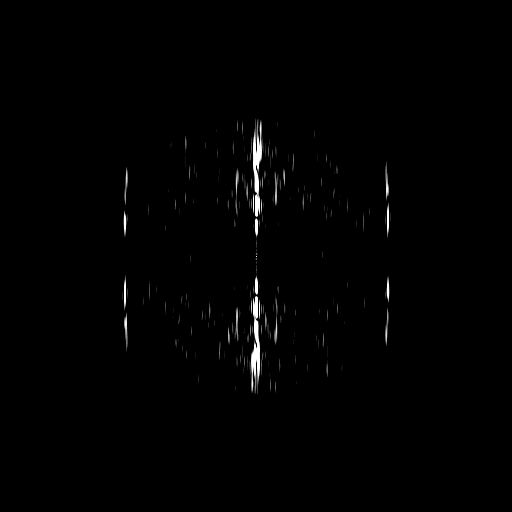

Supplement: Supplementary file 7 — Source Data for Expanded View and Appendix [file EMBR-24-e57264-s003.zip › EMBOR-2023-57264V1_SourceDataForExpandedViewAndAppendix/Figure_EV3/C/LuminalFilaments/TS_328/TS_328.mrc_10.64Apx_bx144_TRIMz_newstack_TRIM_rotx_newboxSHIFT_masked_project_FFT.png]

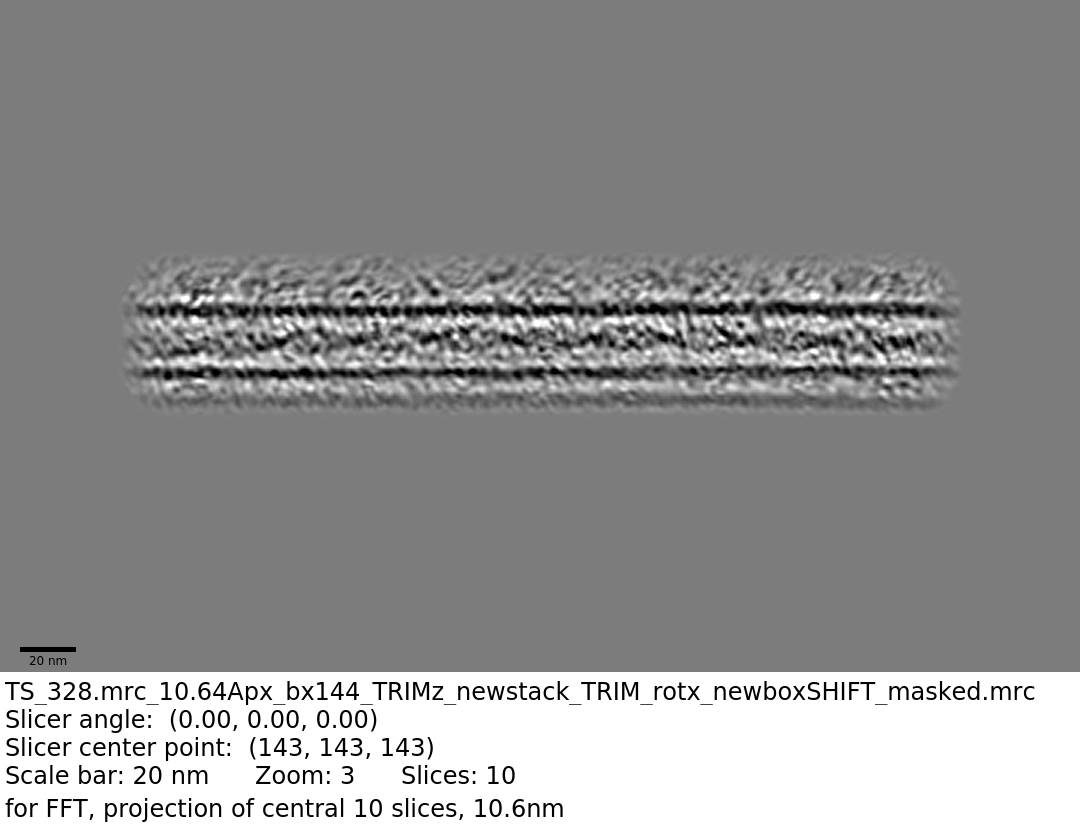

Supplement: Supplementary file 7 — Source Data for Expanded View and Appendix [file EMBR-24-e57264-s003.zip › EMBOR-2023-57264V1_SourceDataForExpandedViewAndAppendix/Figure_EV3/C/LuminalFilaments/TS_328/TS_328.mrc_10.64Apx_bx144_TRIMz_newstack_TRIM_rotx_newboxSHIFT_masked_11nmSlice.png]

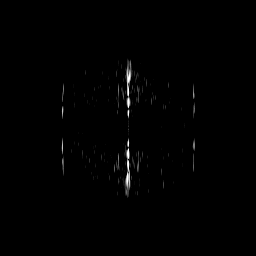

Supplement: Supplementary file 7 — Source Data for Expanded View and Appendix [file EMBR-24-e57264-s003.zip › EMBOR-2023-57264V1_SourceDataForExpandedViewAndAppendix/Figure_EV3/C/LuminalFilaments/TS_328/FFT of TS_328.mrc_10.64Apx_bx144_TRIMz_newstack_TRIM_rotx_newboxSHIFT_masked_project_binnedBy2.png]

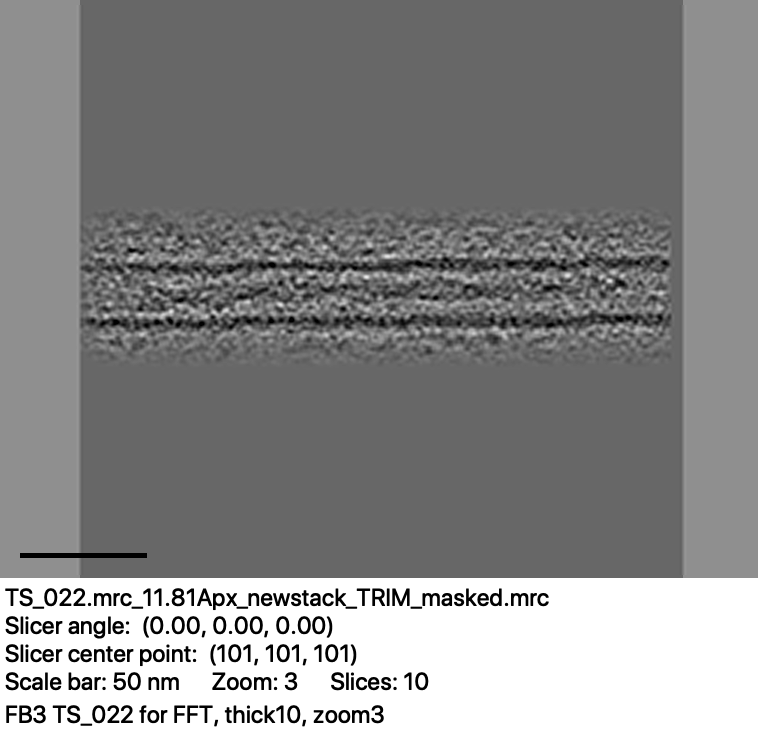

Supplement: Supplementary file 7 — Source Data for Expanded View and Appendix [file EMBR-24-e57264-s003.zip › EMBOR-2023-57264V1_SourceDataForExpandedViewAndAppendix/Figure_EV3/C/LuminalFilaments/FB3_TS_022/FB3_TS_022_thick10_forFFT_beforeProject.png]

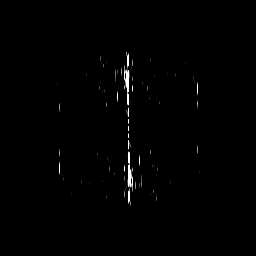

Supplement: Supplementary file 7 — Source Data for Expanded View and Appendix [file EMBR-24-e57264-s003.zip › EMBOR-2023-57264V1_SourceDataForExpandedViewAndAppendix/Figure_EV3/C/LuminalFilaments/FB3_TS_022/TS_022.mrc_11.81Apx_newstack_TRIM_masked_project_FFT_highThreshold.png]
