# Supplementary material for: CryoET shows cofilactin filaments inside the microtubule lumen
Source: EMBO Rep. 2023 Sep 13;24(11):e57264. doi: 10.15252/embr.202357264 (PMC10626427; doi:10.15252/embr.202357264)
Supplement: Supplementary file 8 — PDF+ [file EMBR-24-e57264-s004.pdf]

Report

SOURCE DATA | TRANSPARENT PROCESS | OPEN ACCESS

EMBO reports

CryoET shows cofilactin filaments inside the microtubule lumen

Camilla Ventura Santos[1], Stephen L Rogers[2] & Andrew P Carter[1,*]

Abstract

Cytoplasmic microtubules are tubular polymers that can harbor small proteins or filaments inside their lumen. The identities of these objects and mechanisms for their accumulation have not been conclusively established. Here, we used cryogenic electron tomography of *Drosophila* S2 cell protrusions and found filaments inside the microtubule lumen, which resemble those reported recently in human HAP1 cells. The frequency of these filaments increased upon inhibition of the sarco/endoplasmic reticulum Ca^{2+} ATPase with the small molecule drug thapsigargin. Subtomogram averaging showed that the luminal filaments adopt a helical structure reminiscent of cofilin-bound actin (cofilactin). Consistent with this, we observed cofilin dephosphorylation, an activating modification, in cells under the same conditions that increased luminal filament occurrence. Furthermore, RNA interference knock-down of cofilin reduced the frequency of luminal filaments with cofilactin morphology. These results suggest that cofilin activation stimulates its accumulation on actin filaments inside the microtubule lumen.

Keywords cofilactin; cryoET; luminal filaments; microtubules
Subject Categories Cell Adhesion, Polarity & Cytoskeleton; Post-translational Modifications & Proteolysis; Structural Biology
DOI 10.15252/embr.202357264 | Received 29 March 2023 | Revised 25 August 2023 | Accepted 30 August 2023 | Published online 13 September 2023
EMBO Reports (2023) 24: e57264

Introduction

Microtubules are cytoskeletal filaments that provide mechanical stability and serve as tracks for cargo transport. They consist of α and β-tubulin dimers that assemble into multiple protofilaments and come together to form the polar, tube-shaped filament.

The lumen of the microtubule provides a sheltered, ~15 nm wide space that can accommodate small proteins (Nogales *et al*, 1999). Electron microscopy (EM) studies indeed revealed the presence of distinct, globular densities inside the microtubule lumen of neurons, whose identity and functional role remain unclear (Burton, 1984; Garvalov *et al*, 2006; Bouchet-Marquis *et al*, 2007; Atherton *et al*, 2022; preprint: Chakraborty *et al*, 2022; Foster *et al*, 2022).

Recently, a cryogenic electron tomography (cryoET) study on small molecule-induced protrusions of human HAP1 cells reported the presence of actin-based filaments inside the microtubule lumen (Paul *et al*, 2020). These luminal filaments displayed non-canonical actin morphologies, suggesting that they may be bound by further, unidentified factors. In addition to their unknown composition, microtubule luminal filaments have not been described in cells without induced protrusions, raising the question under which conditions they appear.

Cryogenic electron tomography has been used to characterize objects inside the microtubule lumen. Thin (< 200 nm) samples generate higher signal-to-noise ratios, allowing for subtomogram averages at higher resolution. The model organism *Drosophila melanogaster* provides a set of well-characterized cell types many of which are smaller and thinner than their mammalian counterparts (Cherbas & Gong, 2014). Schneider 2 (S2) cells are derived from the *Drosophila* immune system, are highly susceptible to expression or knock-down of proteins, and have been used to study the microtubule and actin cytoskeleton (Schneider, 1972; Rogers *et al*, 2003; Rogers & Rogers, 2008). They form microtubule-rich protrusions when treated with low concentrations of the actin polymerization inhibitor Cytochalasin D (CytD) (Lu *et al*, 2013), thereby providing an accessible target for microtubule-based studies with cryoET.

Using the particularly thin, CytD-induced protrusions of *Drosophila* S2 cells, we set out to answer (i) whether luminal filaments appear inside S2 cell protrusions and are therefore conserved across species, (ii) what the filaments are composed of and (iii) what causes their occurrence. We found rare occasions of luminal filaments in S2 cell protrusions and increased their frequency by adding thapsigargin (TG), a small molecule inhibitor of the sarco/endoplasmic reticulum Ca^{2+} ATPase (SERCA). Subtomogram averaging suggests that the luminal filaments are formed of cofilactin. In agreement with this, double-stranded RNA (dsRNA) mediated depletion of cofilin led to a change in the morphology of luminal filaments. Taken together, our study suggests luminal cofilactin filaments can occur inside microtubules of induced protrusions in response to specific stress.

1 MRC Laboratory of Molecular Biology, Cambridge, UK
2 Department of Biology and Integrative Program for Biological and Genome Sciences, The University of North Carolina at Chapel Hill, Chapel Hill, NC, USA
*Corresponding author. Tel: +44 1223267060; E-mail: cartera@mrc-lmb.cam.ac.uk

© 2023 MRC Laboratory of Molecular Biology and The Authors. Published under the terms of the CC BY 4.0 license

Results and Discussion

CryoET of induced protrusions of *Drosophila* S2 cells

We incubated *Drosophila* S2 cells with low concentrations of CytD to induce the formation of thin protrusions (Lu *et al*, 2013). Immunofluorescence staining for α-tubulin showed that the protrusions were filled with microtubules (Fig EV1A). We replicated this workflow on EM grids, vitrified the samples, and acquired tilt series on the thinnest regions (Fig 1A and B). We obtained tomograms in which we could unambiguously identify individual organelles and protein complexes such as the endoplasmic reticulum and ribosomes (Fig 1C, Movies EV1 and EV2). The imaged protrusions contained parallel arrays of up to 20 microtubules and were on average 150 nm thick (Fig EV1B–D).

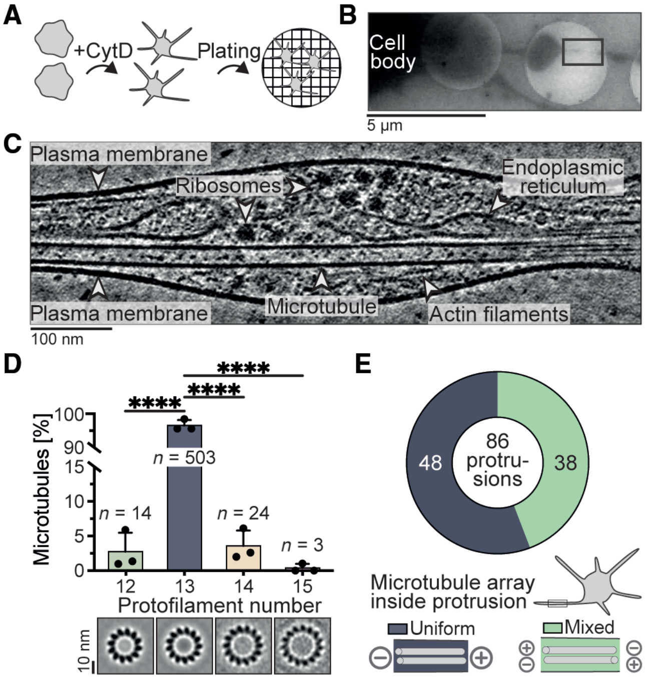

Figure 1. CryoET of microtubule-rich S2 cell protrusions.

A Cartoon showing cellular sample preparation for cryoET. S2 cells were mixed with CytD and then plated on Concanavalin A-coated cryoEM grids.

B Overview image of a vitrified sample.

C Tomogram slice of an S2 cell protrusion acquired at the site in (B) (black box) with labeled cytoskeletal filaments, organelles, and protein complexes.

D Percentage of microtubules with 12 (2.8 ± 2.7%), 13 (93.3 ± 3.2%), 14 (3.6 ± 2.2%), or 15 (0.3 ± 0.6%) protofilaments determined by subtomogram classification of 544 microtubules (mean ± s.d., $N = 3$, ordinary ANOVA test with multiple comparisons, only significant results are labeled with stars, $P < 0.0001$, ****). Bottom panel shows projections of microtubule averages with different protofilament numbers for visualization (projection images are also used in Fig EV1E and F).

E Pie chart of the number of protrusions with uniform (dark blue) or mixed (green) microtubule orientations determined from subtomogram classification. Only protrusions with at least 2 microtubules of resolved orientation were included (86/111). Bottom panel shows cartoons of uniform and mixed microtubule arrays. *N*, *n*: number of biological replicates (*N*), analyzed filaments (*n*).

Source data are available online for this figure.

We performed per-particle subtomogram classification to resolve the protofilament numbers of 544 microtubules inside protrusions, similar to procedures described in Foster *et al* (2022) (Fig EV1E and F). This revealed that the majority (93.3%) had 13 protofilaments whereas minor fractions had 12 (2.8%), 14 (3.6%), or 15 (0.3%) protofilaments (Fig 1D). In 25 of 86 cases, we found microtubules with different protofilament numbers within the same protrusion (Fig EV1G). We previously showed that microtubules from *Drosophila* neurons can have 12 or 13 protofilaments and coexist within the same neurite (Foster *et al*, 2022). Our results from S2 cells provide another example of a cell type in which different protofilament architectures can coincide within the same cell.

We next determined microtubule orientations inside each protrusion from the per-particle classification (Sosa & Chrétien, 1998; Bouchet-Marquis *et al*, 2007; Foster *et al*, 2022) (Fig EV1E and F, Appendix Fig S1). We analyzed protrusions in which we could resolve the polarity of all microtubules. These contained up to 16 microtubules per protrusion. We found 48 of 86 contained a uniform array of microtubules (Fig 1E). The remainder had between one and six microtubules with opposite orientation compared to the majority. In this analysis, we observed a variation across biological replicates, with two datasets showing ~46% uniform orientation and one dataset where 17 of 19 protrusions had microtubules pointing in the same direction (Fig EV1H).

Studies using the fluorescently tagged, tip-tracking protein EB1, found that 80–95% of microtubules are uniformly oriented inside S2 protrusions and that microtubule-based motors are involved in generating this parallel array (Kural *et al*, 2005; del Castillo *et al*, 2015, 2020). We speculate that microtubules with opposite orientation to the majority were captured in our samples prior to motor-mediated sorting (del Castillo *et al*, 2015). In conclusion, our data suggest that many, but not all, *Drosophila* S2 cell protrusions contain uniformly oriented microtubules.

Filaments inside the microtubule lumen increase upon inhibition of the SERCA

We inspected the microtubules in our tomograms and found rare instances of filaments inside their lumen (Fig 2A). These filaments had a similar appearance to filamentous actin (f-actin) found in the microtubule lumen of human HAP1 cells (Paul *et al*, 2020).

Luminal filaments were too rare for an analysis of their distribution and structure and so we set out to increase their frequency. We hypothesized that the filaments may form in response to specific cell stresses and tested if the addition of a drug increased their occurrence. We tested inhibitors of the proteasome (MG132) or the SERCA (TG) to disturb protein and Ca^{2+} homeostasis, respectively (Thastrup *et al*, 1990; Lee *et al*, 1998). While there was no increase in luminal filaments upon proteasome inhibition, SERCA inhibition increased their occurrence in a preliminary experiment (Fig EV2A). We, therefore, prepared samples for cryoET from three biological replicates by treating cells with CytD and either a vehicle (DMSO) or TG (Fig EV2B). We acquired tilt series of these samples and found 105 luminal filaments in TG-treated cells (Fig 2B, Movie EV3). In contrast, only 19 luminal filaments were present in cells treated with a vehicle. We analyzed how many microtubules contained luminal filaments. 7.0 ± 5.0% of microtubules had luminal filaments in DMSO-treated cells (Fig 2C). In contrast, treatment with TG

 © 2023 MRC Laboratory of Molecular Biology and The Authors

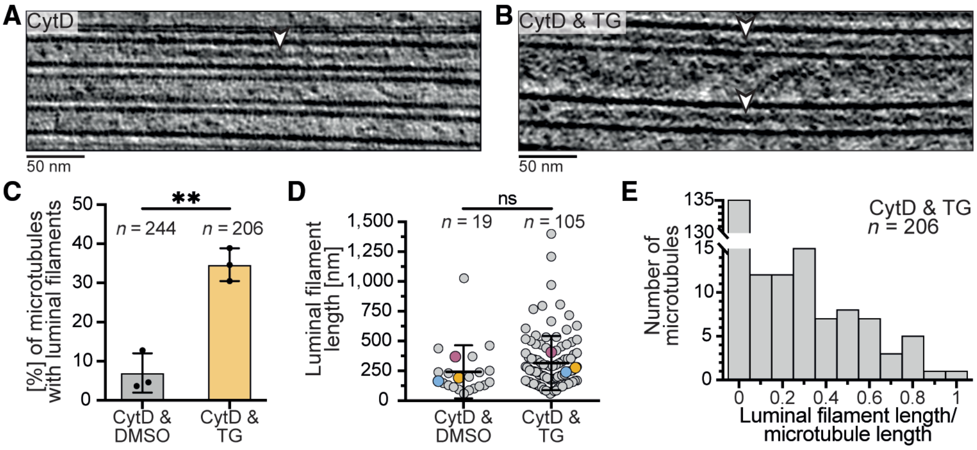

Figure 2. SERCA inhibition with TG increases occurrence of luminal filaments.

A, B Tomogram slices showing filaments inside the microtubule lumen (white arrowheads) in cells treated with CytD (A) or CytD & TG (B).

C Percentage of microtubules containing at least one luminal filament in cells treated with CytD & DMSO (7.0 ± 5.0%) or CytD & TG (34.7 ± 4.2%) showing a significant increase upon TG treatment (mean ± s.d., $N = 3$, unpaired t-test, $P = 0.0019$, **).

D Length of luminal filaments from CytD & DMSO (241.8 ± 110.9 nm) or CytD & TG (308.1 ± 87.1 nm) treated cells showing no significant difference ($N = 3$, means are colored accordingly, non-parametric Mann–Whitney test, $P = 0.4$, ns).

E Histogram of the fraction of microtubule length that is encompassed by luminal filaments for each microtubule in cells treated with CytD & TG. 71 of 206 analyzed microtubules from cells treated with CytD & TG contained at least one luminal filament. Of these, the majority (53.5%) had a length coverage of ≤ 0.34. Graphs and numbers show mean ± s.d. N, n: number of biological replicates (N), analyzed filaments (n).

Source data are available online for this figure.

significantly increased this value five-fold to 34.7 ± 4.2% (Fig 2C). Taken together, we observed filaments inside the microtubule lumen whose frequency increased upon inhibition of the SERCA with TG.

The filaments had varying lengths with the majority (~75%) ranging from 58 to 400 nm (Fig 2D). There was no significant difference between control and TG-treated cells, suggesting that SERCA inhibition increases the frequency but not the length of luminal filaments. Our analysis of the filaments in cells treated with TG showed that most of them only spanned a fraction of the microtubule length (Fig 2E). In rare cases, microtubules contained two and up to four filaments inside their lumen (Fig EV2C and D), consistent with their short length compared to the microtubules.

The luminal filaments occupied a large volume of the microtubule lumen and we asked if this affected the ultrastructure of the surrounding microtubule, for example, by favoring microtubules with larger diameters. We analyzed the protofilament number distribution and found no difference between microtubules with and without luminal filaments (Fig EV2E). We even found a single case where a luminal filament was present in a 12-protofilament microtubule. These results suggest that luminal filaments do not affect the structure of the surrounding microtubule.

Structural analysis suggests that luminal filaments are formed of cofilactin

We inspected the luminal filaments using Fourier transforms to identify repeating units and their frequencies. The luminal filaments periodically widened and compacted every 27.4 ± 0.1 nm (Figs 3A and EV3A). This so-called cross-over distance was significantly shorter than the 35.7 ± 2.4 nm repeat determined for cytoplasmic f-actin (Figs 3B and EV3B and C). The luminal filaments also had wider diameters than cytoplasmic actin (Fig 3A and B), consistent with the hypothesis that they are formed of actin and additionally bound proteins.

We applied subtomogram averaging to gain structural information on the luminal filaments. To acquire sufficient particles, we combined luminal filaments from multiple datasets (see Section Materials and Methods). We manually picked particles, performed initial alignments in Dynamo (Castaño-Díez *et al*, 2012), and then classified and refined particles in Relion 3 (Scheres, 2012; Zivanov *et al*, 2018) (Fig EV3D). Averaging of individual filaments showed that they were polar (Fig EV3D). We, therefore, determined the polarity of each filament and rotated particles accordingly to obtain a uniformly oriented particle set. We noticed that the orientation of luminal filaments did not correlate with the orientation of the microtubules, in which they were contained (Fig EV3E and F). In addition, alignment of the luminal filaments led to smearing of the surrounding microtubule density and *vice versa* (Fig EV3G). These observations indicate that the luminal filaments were not regularly positioned with respect to the microtubule. This misalignment made the filaments a challenging target for subtomogram averaging.

Our structure at 16.5 Å revealed a helical filament inside the microtubule lumen (Figs 3C and D, and EV3H). We first fit an actin filament into our structure and found repeating densities on the edges of the filament that were unaccounted for (Fig 3E and F). We noticed that the helical twist, which describes the angle between two neighboring subunits around the filament axis, was reduced in the luminal filament compared to bare f-actin. To identify actin-binding candidates that could induce a change in helical symmetry and bind at the position of the unaccounted density, we searched

© 2023 MRC Laboratory of Molecular Biology and The Authors

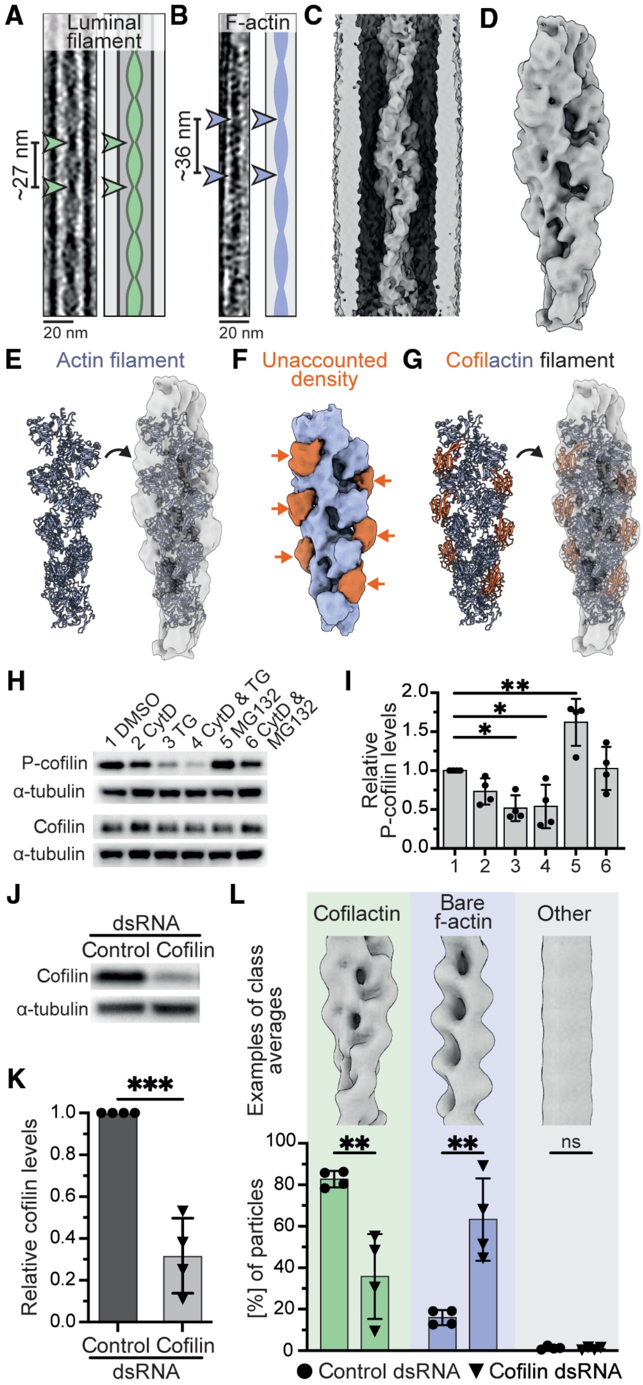

Figure 3. Subtomogram averaging of luminal filaments suggests they are formed of cofilactin.

A, B Tomogram slices and cartoons of a luminal filament (A) and cytoplasmic f-actin (B) inside S2 cell protrusions. Cross-over distances derived from Fourier transforms (Fig EV3A–C) are indicated (arrowheads).

C, D Unmasked (C) and masked (D) subtomogram average structure of the luminal filament at 16.5 Å.

E Fit of an actin filament into the subtomogram average density shown in (D).

F Density representation showing "unaccounted density" from (E) in orange.

G Fit of a cofilactin model (actin: blue, cofilin: orange) into the subtomogram average structure of the luminal filament.

H, I Western blot (H) and quantification (I) showing that TG decreases P-cofilin levels. Top: P-cofilin, bottom: Total cofilin. α-tubulin was used as loading control. Quantification as follows: 1: DMSO (1 ± 0), 2: CytD (0.73 ± 0.17), 3: TG (0.52 ± 0.16), 4: CytD & TG (0.54 ± 0.28), 5: MG132 (1.62 ± 0.30), 6: CytD & MG132 (1.03 ± 0.28), N = 4, ordinary ANOVA test with comparisons to DMSO: DMSO versus TG, P = 0.0276, *; DMSO versus CytD & TG, P = 0.0370, *; DMSO versus MG132, P = 0.0043, **; other comparisons: ns.

J, K Western blot (J) and quantification (K) showing that cofilin levels were reduced to 31.8 ± 18.0% on day 7 or 8 of dsRNA treatment in S2 cells (N = 4, unpaired t-test, P = 0.0003, ***).

L Top: Examples of class averages of different luminal filament types. Bottom: Percentages of luminal filament particles classified into cofilactin (green, 82.7 ± 3.9% control, 35.8 ± 20.5% cofilin dsRNA), bare f-actin (blue, 15.9 ± 3.6% control, 63.3 ± 19.9% cofilin dsRNA) or other (gray, 1.3 ± 0.8% control, 0.9 ± 0.7% cofilin dsRNA) classes. Unpaired t-tests were used to compare classifications from control (spheres) and cofilin dsRNA (triangles) samples (N = 4, cofilactin: P = 0.0041, **, bare f-actin: P = 0.0034, **, other: P = 0.4784, ns). Graphs and numbers show mean ± s.d., N: number of biological replicates.

Source data are available online for this figure.

the EM database (EMDB) for helical filaments containing actin. Bare f-actin and f-actin bound to different families of actin-binding proteins had consistent helical parameters with a helical twist of approx. −167° (Appendix Table S1). Only entries of cofilactin had a reduced helical twist of −162° (McGough *et al*, 1997; Tanaka *et al*, 2018) and this value matched the parameter determined for our luminal filament structure (−161.8°). In agreement with this, a cofilactin model fits well into our structure and the cofilin moiety occupies the extra density that had been unaccounted for when fitting actin alone (Fig 3G).

Cofilin is activated upon SERCA but not proteasome inhibition

Cofilin is a key regulator of the actin cytoskeleton and is highly conserved from yeast to humans with a single homolog in *Drosophila* called *twinstar* (Edwards *et al*, 1994; Gunsalus *et al*, 1995). The affinity of cofilin for actin is strongly increased upon dephosphorylation at a conserved site (Ser3) and cells use this post-translational modification to control cofilin's actin binding and severing activity (Agnew *et al*, 1995; Arber *et al*, 1998; Yang *et al*, 1998; Zebda *et al*, 2000; Niwa *et al*, 2002; Austin Elam *et al*, 2017).

Our structural analysis suggested that luminal filaments were formed of cofilactin (Fig 3G) and the frequency of luminal filaments was increased upon treatment of cells with TG (Fig 2C). We asked if TG activates (i.e., dephosphorylates) cofilin as this could explain the observed increase in luminal cofilactin filaments. To test this, we treated S2 cells with CytD, TG, or MG132 and used antibodies against phosphorylated cofilin (P-cofilin) in western blots. Indeed, TG treatment decreased P-cofilin, indicating cofilin activation (Fig 3H and I). In contrast, MG132 deactivated cofilin by increasing its phosphorylation (Fig 3H and I). These results are consistent with our findings from cryoET where TG but not MG132 treatment increased the frequency of luminal filaments (Figs 2C and EV2A), and provide further support for the role of cofilin in luminal filament formation.

Cofilin dephosphorylation upon specific cell stress has been correlated with the appearance of cofilactin filament bundles (Nishida *et al*, 1987; Ohta *et al*, 1989; Minamide *et al*, 2000). We surveyed

 © 2023 MRC Laboratory of Molecular Biology and The Authors

our tomograms and found putative cytoplasmic cofilactin filaments in three protrusions of control cells (Fig EV3I). In contrast, nine protrusions of TG treated cells contained such filaments in the cytoplasm and they were bundled in five cases (Fig EV3J). Our observation of cofilactin bundles under conditions that activate cofilin are consistent with previous studies.

Luminal filaments have altered morphology in cofilin knock-down cells

To gain further evidence for the presence of cofilin in luminal filaments, we sought to deplete S2 cells of cofilin. We used a dsRNA to reduce cofilin levels to 31.8 ± 18.0% compared to a control dsRNA (Fig 3J and K). We then induced the formation of protrusions and luminal filaments with CytD & TG and prepared samples for cryoET (Movie EV4). To determine changes in luminal filament morphology, we performed subtomogram classification and quantified the results (Fig EV3K and L). In cells treated with a control dsRNA, 82.7 ± 3.9% of luminal filament particles fell into classes with cofilactin morphology (Fig 3L). In contrast, in cofilin knock-down cells this value was reduced to 35.8 ± 20.5% and instead, 63.3 ± 19.9% of particles were present in classes resembling bare f-actin (Fig 3L). We obtained similar results from visual inspection of filament morphologies in the tomograms (Fig EV3M). A small fraction of luminal filaments was neither cofilactin nor bare f-actin and adopted alternative filament morphologies (Fig EV3N). The increase in bare f-actin within the microtubule in the absence of cofilin provides direct evidence that the luminal filaments we observed contain cofilin.

In summary, we combined cryoET, subtomogram averaging, and RNA interference to show that the filaments we observed inside the microtubule lumen of *Drosophila* S2 cells are formed of cofilactin. Furthermore, we provide evidence that these filaments specifically accumulate in response to TG. Although this SERCA inhibitor induces endoplasmic reticulum stress, we suspect that luminal filament accumulation is not a general stress response as we did not observe it in the presence of the proteasome inhibitor MG132. Instead, we suggest luminal filament accumulation is due to TG directly activating cofilin in S2 cells.

Cofilin is an actin-binding protein that severs f-actin at low concentrations and decorates it to form cofilactin at high concentrations (Nishida *et al*, 1984; Maciver *et al*, 1991; Andrianantoandro & Pollard, 2006). It is activated both by cellular stresses, such as energy depletion (Nishida *et al*, 1987; Meberg *et al*, 1998; Minamide *et al*, 2000), and by signaling pathways, particularly those found at the leading edge of the cell (Meberg *et al*, 1998; Chan *et al*, 2000; Zebda *et al*, 2000; Endo *et al*, 2003). Indeed, filaments resembling cofilactin have been observed in the cytoplasm of neuronal growth cones in two recent cryoET studies (Atherton *et al*, 2022; Hylton *et al*, 2022). It remains to be seen whether cofilactin can also accumulate in the microtubule lumen during cell migration.

Although cofilin knock-down in S2 cells led to a major change in the filament morphology, we observed only a minor (and non-significant) decrease in the total frequency of luminal filaments (Fig EV3O). We speculate this may be due to the residual cofilin remaining in the cells (Fig 3J and K) being able to nucleate filaments inside the microtubule. This would be consistent with our observations of luminal filaments that transitioned from a cofilactin to a non-cofilactin morphology (Fig EV3P, Movie EV4).

It is unclear how actin and cofilin get inside the microtubule. Entry of preassembled cofilactin filaments via microtubule ends appears unlikely given the narrow diameter of the microtubule lumen in comparison to cofilactin filaments. Individual components could enter through breaks in the microtubule lattice (Chakraborty *et al*, 2020; Atherton *et al*, 2022; Foster *et al*, 2022). Alternatively, microtubules may polymerize around pre-existing cytoplasmic filaments, resulting in their encapsulation and possibly protection from disassembly through spatial separation from cofilin kinases and other deactivating factors. In this case, actin filaments could "guide" the polymerization of microtubules thereby influencing the organization and dynamics of the microtubule network.

The physiological role of cofilactin within the microtubule lumen, if any, remains unknown. One possibility is that they stabilize the surrounding microtubule. In our best tomograms, we observed connections between luminal filaments and the microtubule wall (Fig EV3Q), which could structurally reinforce the microtubule lattice. A similar stabilizing role has been proposed for the globular luminal particles found in neurons and these are also frequently linked to the surrounding microtubule via short tethers (Garvalov *et al*, 2006; Cuveillier *et al*, 2020; preprint: Chakraborty *et al*, 2022; Foster *et al*, 2022). The observation that microtubule luminal filaments were also found in human cells (Paul *et al*, 2020) suggests that their function may be conserved across species.

Materials and Methods

Cell culture

Schneider S2 cells (UCSF, ThermoFisher Scientific, #R69007, tested for mycoplasma contamination) were grown in Schneider's medium (21720001, Gibco or 04-351Q, Lonza) supplemented with 10% heat-inactivated FBS, 100 units/ml Pen/Strep and 0.25 µg/ml Amphotericin B (15290018, Gibco) at 25°C. Cells were split every 2–4 days and maintained until passage 25.

Generation of a *Drosophila* α-tubulin-acetylase (dTAT) knock-out line using CRISPR

The dTat knock-out cell line was made from S2-DRSC (DGRC Stock 181; https://dgrc.bio.indiana.edu//stock/181; RRID:CVCL_Z992) using protocols created by Bassett *et al* (2014). A pair of 20-mer oligonucleotides targeting the 5′ end of the *dtat* coding sequence (GTTCGCGCAGCCAATCATCA) was ligated into pAc-sgRNA-Cas9 (Addgene) and validated by sequencing. The construct was transfected into low-pass S2 cells using TransIT Insect transfection reagent (Mirus) and stable cells carrying indels for this locus were selected with 5 µg/ml puromycin for 3 weeks. Loss of dTAT was validated by immunoblotting and immunofluorescence for acetylated α-tubulin (Appendix Fig S2A and B). The dTAT knock-out cell line will be distributed through the Drosophila Genomics Resource Center (Bloomington, IN, code DGRC#344).

Double-stranded RNA interference

dsRNAs were synthesized by *in vitro* transcription from PCR products using forward and reverse primers containing the T7 promoter

© 2023 MRC Laboratory of Molecular Biology and The Authors

sequence (TAATACGACTCACTATAGG) with homemade Pfu. Amplicons were selected by querying gene-specific sequences using http://www.genomernai.org/ and amplified from genomic DNA isolated from S2 cells using Trizol purification. Cofilin/*twinstarI* dsRNA was synthesized using primers (5′-TAATACGACTCACTATAGG-CATTTCTGGATATCTTCTAGAAACT-3′ and 5′-TAATACGACTCAC-TATAGGTGGTGTAACTGTGTCTGATGTCTG-3′). PCR product sizes were validated by agarose gel electrophoresis and concentrated by precipitation with sodium acetate and cold ethanol. dsRNA was synthesized overnight at 37°C using T7 RNA polymerase (FisherBiosci) in transcription buffer (40 mM Tris–HCl pH 8.0; 10 mM DTT; 2 mM Spermidine-HCl (Sigma); 20 mM MgCl2; 7.5 mM rNTPs (Promega); inorganic pyrophosphatase (FisherBiosci)). *In vitro* transcription reactions were treated with DNAse (NEB) for 1 h at 37°C, quantitated for yield by comparison against a DNA ladder, precipitated with sodium acetate and cold ethanol, and resuspended in DNAase/RNAse-free water to a concentration of 1 mg/ml. For all experiments, we included a negative control with dsRNA corresponding to the sequence of bacterial chloramphenicol transferase using primers (5′-TAATACGACTCACTATAGGGATCCCAATGGCATCGTAAAGAA-CATTTGAGGC-3′ and 5′- TAATACGACTCACTATAGGGGGGCGAA GAAGTTGTCCATATTGGCCA-3′).

For control and cofilin knock-down experiments, 1 ml of dense S2 cell culture was added to 9 ml of serum-free S2 media (Schneider's medium, 100 units/ml Pen/Strep) and pelleted for 10 min at 600 × *g*. The supernatant was removed and cells were plated at a density of 5 × 10^5 cells/ml in 1 ml serum-free media in a well of a 12-well plate and allowed to adhere to the surface for 20–30 min. The media was then replaced with 500 µl serum-free media containing 7 (replicate 1) or 20 µg (all other replicates) dsRNA for control or cofilin RNA interference and incubated for 1 h at 25°C in a humidified chamber. Then, we added 500 µl of S2 media with 20% FBS. The same procedure was repeated 3 days later. Cells were frozen on day 7 or 8 of the experiment. Knock-down efficiencies of all replicates used for cryoET data acquisition were examined by western blotting for total cofilin. Western blots and quantifications were performed as described in the Section "Western blotting and quantification" with the modification that only approx. 1–10 × 10^5 cells were used. Cells were preincubated with 2 µM TG for 3 h prior to lysis and western blotting because part of each batch was also used for the cryoET sample preparation.

Western blotting and quantification

For western blots of P-cofilin, we seeded cells at 2 × 10^6 cells/ml in S2 media in 2 ml media/well in a 6-well plate with either DMSO (D2650, Merck), 2.5 µM CytD (C2618, Sigma), 2 µM TG (ab147487, Abcam), 2.5 µM CytD & 2 µM TG, 25 µM MG132 (M7449, Merck), or 2.5 µM CytD & 25 µM MG132. All samples were adjusted to 0.275% (v/v) DMSO. Cells were incubated with the drugs for 5 h at 25°C, then washed with 1 ml ice-cold PBS and lysed in 70 µl RIPA buffer/well (89900, Thermo Scientific) supplemented with complete protease inhibitor cocktail (11697498001, Sigma) and Halt™ phosphatase inhibitor cocktail (1862495, Thermo Scientific). All following steps were performed at 4°C unless stated otherwise. Cells were scraped off with the tip of the pipet and transferred to a 1.5 ml tube. Samples were incubated for 15 min and then centrifuged for 12 min at 21,000 × *g*. Supernatants were transferred to a fresh tube and boiled with NuPAGE LDS Sample buffer (4×, NP0007, Invitrogen) for 5 min. Samples were run on a NuPAGE gel at 120–150 V and blotted onto a PVDF membrane using a Trans-blot Turbo Transfer System (Biorad) in the mixed molecular weight program. Membranes were cut above the 25 kDa band to blot for the loading control (α-tubulin) and cofilin or P-cofilin. Membranes were blocked with 5% milk in TBST (20 mM Tris–HCl, 150 mM NaCl, 0.1% (w/v) Tween20, α-tubulin, and cofilin) or in 5% (w/v) BSA (A3311, Sigma) in TBST (P-cofilin) for 30–60 min at room temperature. Membranes were incubated with primary antibodies at 4°C overnight or for 1 h at room temperature at the following dilutions: α-tubulin (sc-32293, Santa Cruz, diluted 1:5,000 in 5% milk, mouse), anti-cofilin (gift from Anna Marie Sokac, published in Figard *et al*, 2019, diluted 1:5,000 in 5% milk, rabbit), anti-P-cofilin (gift from Buzz Baum, published in Jovceva *et al*, 2007, diluted 1:2,000 in 5% (w/v) BSA, rabbit). Membranes were washed four times for 5 min each in TBST and incubated with Polyclonal goat anti-mouse (P044701, Agilent, 1:2,500) or anti-rabbit (P044801, Agilent, 1:2,000) immunoglobulins/HRP for 1 h at room temperature. Membranes were again washed four times for 5 min each with TBST and developed using ECL Select Western Blotting Detection Reagent (RPN2235, Amersham). Membranes were imaged on a Bio-Rad Gel Doc imager.

We quantified the western blots by drawing a square around each band and measuring the integrated density in ImageJ (Schindelin *et al*, 2012). Bands from P-cofilin, cofilin, and α-tubulin were first normalized to the band intensity of the respective DMSO treatment. P-cofilin and cofilin were then normalized to their corresponding loading controls (α-tubulin). To determine the relative phosphorylation, normalized P-cofilin band intensities were divided by normalized cofilin intensities for each sample.

For western blots of acetylated α-tubulin, lysates were prepared from control S2 cells and dTAT knock-out cells by resuspending cells directly into SDS–PAGE sample buffer and boiling. Samples were separated on a 15% SDS–PAGE gel and transferred to nitrocellulose. Blots were probed with mouse monoclonal antibodies against α-tubulin (clone DM1A, T9026, Sigma, diluted 1:10,000, mouse) or acetylated tubulin (clone 6-11B-1, T6793, Sigma, diluted 1:1,000, mouse) overnight at 4°C. After washing, blots were probed with HRP-conjugated goat anti-mouse secondary antibodies (71045, EMD Millipore, diluted 1:5,000) for 2 h at room temperature, washed, and visualized using chemiluminescent detection (SuperSignal West Pico, Thermo Scientific).

Immunofluorescence and light microscopy imaging

For immunofluorescence of α-tubulin in the presence and absence of CytD, autoclaved 13 mm coverslips were placed in wells of a 24-well dish and coated with 0.25 µg/ml Concanavalin A (L7647, Sigma) for at least 1 h and up to overnight at 37°C. Coverslips were washed twice with PBS before plating 1.5 × 10^5 cells/well with either DMSO or 2.5 µM CytD. DMSO concentrations were adjusted to 0.125% (v/v) in both samples. Cells were grown for 5 h before fixing for 10 min in fixation buffer (3% (v/v) paraformaldehyde, 0.1% (v/v) Glutaraldehyde, 4% (w/v) Sucrose, 0.5% (v/v) Triton-X-100, 0.1 M PIPES pH 7, 2 mM EGTA, 2 mM $MgCl_2$). This and all the following steps were performed at room temperature. Samples were washed three times with PBS, permeabilized with 0.1% (v/v) Triton-X-100 in PBS

 © 2023 MRC Laboratory of Molecular Biology and The Authors

for 10 min and washed again with PBS. Samples were blocked with 2% (w/v) BSA in PBS for 30–60 min and then incubated with primary antibody against α-tubulin (sc-32293, Santa Cruz, diluted 1:500, mouse) for 1 h. Samples were washed with PBST and incubated with secondary Alexa Fluor 647 donkey anti-mouse (Invitrogen, diluted 1:1,000) for 1 h. Samples were mounted with mounting media (P36962, Invitrogen) and left to cure overnight before imaging on a Zeiss 880 confocal microscope equipped with a 63×/1.4 NA Oil lens. Z-stacks including the entire volume of the cell (24 slices, 0.63 μm/pixel) were acquired and summed to generate a projection image showing all microtubules within the cell.

Immunofluorescence of acetylated α-tubulin in control and dTAT knock-out cells was performed as described in Rogers and Rogers (2008). Briefly, cells were plated on Concanavalin A coated coverslips and washed with hemolymph-like buffer (70 mM NaCl, 5 mM KCl, 20 mM $MgCl_2$-$6H_2O$, 10 mM $NaHCO_3$, 5 mM trehalose, 115 mM sucrose, 5 mM HEPES, 1 mM EGTA at pH 7.2) before fixation with 10% (v/v) formaldehyde in hemolymph-like buffer for 10 min at room temperature. Samples were permeabilized with 0.1% (v/v) Triton-X-100 in PBS for 15 min and blocked with 5% (w/v) BSA in PBS supplemented with 0.1% Triton-X-100 (PBST). Primary antibodies against α-tubulin (clone DM1A, T9026, Sigma, diluted 1:1,000) or acetylated tubulin (clone 6-11B-1, T6793, Sigma, diluted 1:1,000) in 5% (w/v) BSA in PBST were added for 1 h at room temperature. Samples were washed with PBST and incubated with secondary antibodies (Cy-3-conjugated goat anti-mouse, Jackson Immunoresearch) for 1 h before washing in PBST and mounting. Samples were imaged on a Nikon inverted Ti Eclipse microscope equipped with a 100×/1.4 N.A objective lens.

Sample preparation for cryoET

Quantifoil R3.5/1 Au200 grids (Quantifoil) were glow discharged for 30 s in an Auto306 Edwards Turbo Coater at power 6 (20 mA) and then transferred to a sterile hood. From here, samples were prepared in an upside-down p10 dish, in which the lid was covered to ~70% with a sterile piece of parafilm. 30-μl droplets of 0.25 μg/ml Concanavalin A were placed on the parafilm in the p10 dish and one glow-discharged grid was added to each droplet with the carbon side facing up. The p10 dish was closed and placed in a humidified chamber at 37°C for 1–16 h. Before plating cells, grids were washed twice with PBS. For all experiments, we plated 8,000–10,000 cells in 30 μl droplets per grid and incubated them in a humidified chamber at 25°C until vitrification.

Samples used for quantification and subtomogram averaging of microtubules in protrusions were plated with either 2.5 μM (datasets 1, 2, 3) or 5 μM (dataset 4) CytD for 4 h.

To prepare samples for the quantification of luminal filaments, cells were first plated in a 12-well plate at a density of 1 × 10^6 cells/well with either 0.1% (v/v) DMSO or 2 μM TG (final DMSO conc. 0.1% (v/v)) for 1 h. Cells were then collected, counted, and plated on prepared grids with either 2.5 μM CytD & 0.1% (v/v) DMSO or 2.5 μM CytD & 2 μM TG. Cells were vitrified 4 h after plating on grids. In total, cells were incubated with CytD for 4 h and with DMSO/TG for 5 h. The final DMSO concentration in all samples was 0.125% (v/v).

For preliminary experiments shown in Fig EV2A, cells were plated in 2 μM TG or 25 μM MG132 for 3 h. Cells were then collected and plated on grids with 2.5 μM CytD for another 2 h before vitrification. The final DMSO concentration was 0.125% (v/v) for CytD & TG, and 0.275% (v/v) for CytD & MG132.

Samples from cofilin knock-down cells were prepared on day 7 or 8 of the knock-down. Cells were incubated with 2 μM TG for 3 h, then mixed with 2.5 μM CytD and plated on EM grids following the procedure given above. Samples were vitrified after 5 h of TG and 2 h of CytD treatment.

Vitrification of specimen

Samples were vitrified similar to the procedure published in Foster *et al* (2022). Briefly, Whatman No. 1 filter paper stripes were cut to dimensions of approx. 4 × 0.7 cm and the final 0.5 cm were folded over by 90°, generating an L-shaped stripe. Stripes were incubated in a humidified chamber for at least 20 min before use. A Vitrobot MkII (Thermo Fisher) was set to 25°C with 100% humidity and automated blotting was disabled. One grid was taken up with Vitrobot tweezers, placed on the bench and 4 μl of S2 media with 0.1% (v/v) pluronic-F68 (24040-032, Gibco) and 29% (v/v) 10 nm BSA-coated gold fiducials in PBS (see below) were carefully placed on the grid. The grid was then loaded onto the Vitrobot, blotted from the backside using the L-shaped filter paper stripes and plunge-frozen in liquid ethane.

BSA-coated gold fiducials were generated by incubating 9.5 ml gold colloid (EM.GC10, BBI solutions) with 0.5 ml 5 mM sodium phosphate buffer, pH 5.0, with 2.5 mg BSA. The final mixture contained 250 μg/ml BSA. The sample was incubated at 4°C overnight on a rotating wheel. BSA-coated gold was washed by splitting the volume into 1.5 ml tubes and centrifuging at 25,000 × *g* for 1 h at 4°C. The supernatant was discarded, pellets were resuspended in PBS and the procedure was repeated. Finally, pelleted BSA-coated gold was pooled and resuspended in a final volume of 200–250 μl PBS. Fiducials were stored at 4°C for 6–12 months.

Electron microscopy data acquisition and tomogram reconstruction

Thin regions of protrusions were selected for tilt series acquisition on a 300 kV FEI Titan Krios (Thermo Fisher) equipped with a K2 or K3 detector and energy filter (Gatan, slit width 20 eV) using SerialEM software (Mastronarde, 2005). A dose-symmetric scheme (Hagen *et al*, 2017) was used to acquire tilt series with 2° or 3° increments between ±60°. 10 or 14 dose-fractionated images were acquired at each tilt with a total dose of 112–124 $e^-/Å^2$. Data were acquired with a pixel size of 2.952 or 2.659 Å/pixel and a nominal defocus of −2.5 to −6 μm. Details for each dataset are in Appendix Table S2–S4.

All pre-processing and reconstruction steps were performed in WARP 1.0.9 (Tegunov & Cramer, 2019) unless stated otherwise. Frames were aligned and gain-corrected. The defocus was estimated and bad tilt images were manually removed. A trained model was used to pick fiducials and mask them during tomogram reconstruction. Tilt series were aligned in IMOD (version 4.10.49), AreTomo (version 1.1.1), or in MATLAB using dautoalign (https://github.com/alisterburt/autoalign_dynamo). Tilt series were CTF-corrected and tomograms were reconstructed with a binning of 4 (bin4, 11.81 or 10.64 Å/pixel). For quantifications and images shown in figures,

© 2023 MRC Laboratory of Molecular Biology and The Authors

bin4 tomograms were deconvolved in WARP using a Wiener-like filter (Tegunov & Cramer, 2019). Tomograms of bad quality in which microtubules and luminal filaments could not be reliably identified were excluded from all analyses.

Images of slices through tomograms were generated by opening bin4, deconvolved tomograms in IMOD, setting the slice thickness to 5 and adjusting the angles in the slicer window.

Quantification of protrusion thickness

The thickness of protrusions from vitrified specimen (datasets 1–4) was measured in IMOD using the distance measurement tool. Briefly, bin4, deconvolved tomograms (11.81 Å/pixel) were opened in IMOD and adjusted in the slicer window to show the cross-section of the protrusion with 10 overlaid slices. For each protrusion, we estimated the thickness at three positions along the protrusion and determined the average protrusion thickness. The graph depicts the average thickness of each protrusion. If two protrusions were present in the same tomogram, they were measured separately. 111 protrusions from 107 tomograms were 150.1 nm ± 39.0 (mean ± s.d.) thick. Protrusions from different biological replicates are colored differently.

Subtomogram averaging and classification of microtubules

Microtubules were picked in IMOD from cross-sections of bin4, deconvolved tomograms (11.81 or 10.64 Å/pixel). Approximately 10 points were placed in the middle of each microtubule along the protrusion. Each microtubule was picked in a different contour. Model points were exported as coordinates and imported into Dynamo 1.1.460 (Castaño-Díez *et al*, 2012) with the filament with torsion model workflow using a custom script, as described in Foster *et al* (2022). All following steps were performed in Dynamo unless stated otherwise. Particles were cropped from bin4 tomograms every 8 nm with a box size of 50 pixels. Particles were averaged and jointly aligned to the generated average. After joint alignment, particles from each microtubule were averaged to generate per-microtubule averages. Across our data (> 1,000 microtubules), we found microtubules with 12, 13, 14, and 15 protofilaments. We chose "good-looking" individual filament averages of each protofilament number and rotated each of them by 180° around the axis perpendicular to filament axis to generate a reference with the same protofilament number but opposite polarity. The resulting eight references (12, 13, 14, 15 protofilaments, each plus- and minus-end-facing) were used for an initial multireference alignment of all particles of one dataset. The class averages after this alignment had higher signal-to-noise ratios than the initial references because more particles were present in each average, and they were used as initial references for all the following multireference alignments.

To automatically determine the polarity and protofilament number of each microtubule, the aligned particle table was first cleaned by retaining 80% of particles with the best cross-correlation scores. A custom MATLAB script was used to assess the percentage of particles in each filament that classified into either class and to assign each filament to a protofilament number and orientation. A microtubule was assigned if at least 65% of particles in that filament classified into that class or if at least 50% of particles classified into a single class and the overall cross-correlation score was equal or higher 0.13. Microtubules that did not match either of these criteria remained undetermined (30 of 574) and were excluded from all quantifications. The classification and analysis procedures were validated across multiple datasets by comparing the results of this analysis to visual inspection of individually averaged microtubules. Scripts used for the export of IMOD filament models and for the determination of microtubule polarity and protofilament number are available from GitHub (https://github.com/carterlablmb/Ventura-Rogers-Carter-2023.git).

Using this procedure, we determined the protofilament number of 544 microtubules from 111 protrusions from three biological replicates and five technical replicates. Technical replicates were pooled and the percentage of 12, 13, 14, 15 protofilament microtubules was determined for each biological replicate as shown in Fig 1D. Data from all replicates was pooled to show the number of protrusions containing microtubules with different protofilament numbers in Fig EV1G.

The protofilament numbers of microtubules from DMSO and TG treated cells were determined by classification as described above. We then analyzed which microtubules contained luminal filaments and which did not. We calculated the percentage of microtubules with 12, 13, 14, 15 protofilaments in each biological replicate for microtubules with or without luminal filaments as shown in Fig EV2E. For statistical analysis, means and s.d.s of the normalized protofilament number percentages were calculated for microtubules without and with luminal filaments. Samples treated with DMSO or TG were joined for this analysis as we found no difference in protofilament number distribution (multiple unpaired *t*-tests, non-significant). A two-way ANOVA with comparisons between microtubules with or without luminal filaments of each protofilament number was performed to determine statistical significance. All comparisons showed no significance. The number of microtubules analyzed in each biological replicate under each condition can be found in Appendix Table S3. 35/119, 21/139, and 30/192 microtubules from biological replicates 1–3 (datasets 5–7), respectively, contained luminal filaments. 21 of 450 microtubules had unassigned protofilament number and were excluded from the quantification, leading to the following numbers for microtubules with luminal filaments for the three biological replicates: 35/117, 18/130, and 29/182.

Analysis and quantification of luminal filaments

The selection of regions of the grids on which tomograms were collected was carried out without knowing whether luminal filaments were present or not, because the filaments were not visible until after tomogram reconstruction. For each dataset, luminal filaments were analyzed in tomograms following the order of acquisition (i.e., without randomization). This means that tomograms from each sample condition were analyzed as a batch but the condition was not explicitly known at the time of analysis.

To quantify the occurrence and lengths of luminal filaments, we manually picked them from bin4 deconvolved tomograms in IMOD. Filaments were picked in the slicer window from side views and adjusted in a second slicer window showing a cross-section of the same filament. One filament was picked per contour.

To quantify the percentage of microtubules that contained luminal filaments, we mapped each filament to its surrounding microtubule and determined the percentage of microtubules that contained at least

 © 2023 MRC Laboratory of Molecular Biology and The Authors

one luminal filament (Fig 2C). An unpaired *t*-test was used to determined statistical significance between percentages from 3 biological replicates (technical replicates were pooled). To determine the filament lengths, we extracted the filament contour length using the "imodinfo" command (Fig 2D). The filament lengths were not normally distributed as determined from a D'Agostino and Pearson test applied to each biological replicate and each treatment. Thus, we performed a non-parametric Mann–Whitney test and found no statistically significant difference in luminal filament lengths between CytD & DMSO and CytD & TG treated samples (Fig 2D).

To determine which proportion of microtubule was covered by luminal filaments, we placed multiple points of a single contour along the length of each microtubule and used the "imodinfo" command to extract the contour (i.e., microtubule) length. We then divided the length of the luminal filaments within a single microtubule by the length of the surrounding microtubule (Fig 2E). A coverage of 1 means that the entire microtubule was covered with a luminal filament. A coverage of 0 means that there were no filaments within this microtubule. If there were multiple filaments within the same microtubule, their lengths were summed prior to division by the microtubule length.

To quantify the lengths of luminal filaments with cofilactin or non-cofilactin morphology by visual inspection of tomograms (Fig EV3M), we measured the length of each filament and classified it as cofilactin-like or non-cofilactin like. Examples for cofilactin and non-cofilactin morphologies can be found in the right panel of Fig EV3M. For filaments that transitioned between morphologies, we measured the lengths of each morphology. Percentages of each morphology were determined for three biological replicates (technical replicates were pooled) and mean ± s.d.s are shown in Fig EV3M. An unpaired *t*-test was used to determine the statistical significance between the percentage of luminal cofilactin filaments in control and cofilin dsRNA knock-down cells.

Orientations of luminal filaments relative to the surrounding microtubule were determined as follows. Particle positions and orientations from luminal filaments were obtained from the final particle table obtained by subtomogram averaging (see Section "Subtomogram averaging of luminal filaments"). This included 109 luminal filaments within 76 microtubules in 33 protrusions. A particle table of uniformly oriented microtubules was generated by rotating those that had been classified as "minus-end-directed" in the subtomogram classification approach by −180° around the second Euler angle (theta). As a result, arrows of microtubule particles in Fig EV3E point towards the microtubule plus end. Luminal filament particles also point towards the cofilactin barbed (plus) end. The orientation of each luminal filament was assessed relative to the microtubule, in which it was contained, by plotting particle positions and orientations along the filament axis in Chimera (Pettersen *et al*, 2004) using the "Place object" plugin (Qu *et al*, 2018).

Survey of cytoplasmic cofilactin filaments

To determine the number of cytoplasmic cofilactin filaments, we surveyed tomograms of CytD & DMSO and CytD & TG-treated cells (datasets 5–7) for cytoplasmic filaments with cofilactin morphology, i.e., morphology similar to the filaments found inside the microtubule lumen. We observed such cytoplasmic filaments in three protrusions from CytD & DMSO and in nine protrusions from CytD & TG treated cells. In five of nine cases from CytD & TG cells, the filaments were bundled.

Determination of the cross-over distance of filaments from Fourier transformations

Fourier transforms in Fig EV3A and B were generated from a 10.64-nm thick slice of the luminal filament (A, left panel) or from a masked projection of a cytoplasmic f-actin (B, left panel). Both filaments are from induced S2 cell protrusions.

To quantify the cross-over distance of luminal filaments and cytoplasmic f-actin, three luminal and four cytoplasmic actin filaments from well-aligned bin4 tomograms (11.81 or 10.64 Å/pixel) were oriented horizontally in the xy-plane using the --rot flag in EMAN2 (Tang *et al*, 2007) or newstack in IMOD. The volumes were then trimmed using IMOD's trimvol tool to a box size of 200–285 pixels and masked with a Gaussian cylindrical mask with a diameter of ~28 nm to exclude densities outside the luminal filament and the surrounding microtubule. The masked volumes were projected along the filament axis and Fourier transformed in ImageJ. Fourier transforms with a box size of 512 pixels were binned by 2 to a box size of 256 pixels. Layer lines corresponding to the cross-over distance were identified and the distance between the equator and the line was measured using the line tool in Image J. The corresponding frequency was calculated with the following formula: box size [pixel^2] × pixel size [nm/pixel]/distance between equator and layer line [pixel].

Subtomogram averaging of luminal filaments

Microtubules from dTAT knock-out cells contained luminal filaments with a similar appearance as those from wild-type cells (Appendix Fig S2C, dataset 8) and this data was joined with datasets 5–7 for subtomogram averaging of luminal filaments. All datasets were first processed independently until stated otherwise. IMOD models of luminal filaments picked manually for the quantification of luminal filament lengths (see Section Analysis and quantification of luminal filaments) were exported into Dynamo "filament with torsion" models as described above. Particles were cropped at a four-time binned pixel size (11.81 or 10.64 Å/pixel) with a box size of 50 pixels and with a particle distance of 6 nm. To generate an initial reference, particles were aligned to 5 references generated by averaging 50 randomly chosen particles in a multireference alignment in Dynamo with a cylindrical soft-edged mask. The cone-search along the filament was limited to 30°, the in-plane angular search to 180°, and the shift along the filament axis to 2.4 nm during the alignment with 6 iterations. A good reference was chosen from the final classes and used as an initial reference for a joint alignment of all particles.

To obtain a reference for determining the orientation of filaments, per-filament averages were generated by averaging particles from each filament after joint alignment. A good average was selected and rotated by 180° using EMAN2 to obtain a reference with opposite filament polarity. These two references were used in multireference alignments in Dynamo. Filament polarities were determined with a custom-script that assessed the proportion of particles in each filament that classified into each polarity class. Filaments were assigned to the polarity in which the majority of

© 2023 MRC Laboratory of Molecular Biology and The Authors

particles classified. Particles from filaments with one polarity were rotated by 180° around the second Euler angle using subTOM software (version 1.1.2) (https://github.com/DustinMorado/subTOM/releases/tag/v1.1.4). This rotation was applied to the original particle positions, not the particle positions obtained after the initial alignment. An array of random subsets was generated by placing particles from each filament into the same subset to avoid duplicate particles in separate groups used for resolution estimation.

To transition to Relion, Dynamo particle tables were converted to Relion star files using dynamo2warp from the Dynamo2M package (https://github.com/alisterburt/dynamo2m) and additional columns such as filament number and random subset number were added using the Starparser package (https://github.com/sami-chaaban/starparser). Particles were reconstructed at a pixel size of 5.90 Å/pixel in WARP with a box size of 120 pixels. All following steps were performed in Relion 3.1 unless stated otherwise. Particles from each dataset were imported with different optics groups and joined using the "join star files" option. The starting particle number was 7,549 particles. To obtain an initial reference, a mask and helical parameters, particles from dataset 8 were refined without helical symmetry using a cylindrical mask. The resulting reference was then refined with a mask generated in Chimera by fitting a cofilactin filament model (PDB 5YU8) into the structure and using the "color-zone" function to generate a map from the averaged luminal filament density without the surrounding microtubule density. The resulting refined average from dataset 8 was used to determine initial helical symmetry parameters. The average was lowpass filtered (60 Å) and, together with the mask generated in Chimera, used in a 3D classification of particles from all datasets (5–8) to select good particles. Particles were classified into six classes with $T = 1$ and helical reconstruction and symmetry (initial twist: −162°, initial rise: 29 Å). A selection of 3,801 particles from four classes was further refined. The refined particle positions and orientations were used to re-reconstruct particles at a pixel size of 2.95 Å/pixel with a box size of 256 pixels in WARP. Particles were aligned in a final refinement with $T = 6$.

To determine the correct pixel size of the final map, we used the generated *Drosophila* cofilactin model (see Section Generation of a *Drosophila* cofilactin model). We fit this model into the post-processed and masked map using the option "use map simulated from atoms, resolution 17 Å" in Chimera. The correlation of the fit was noted in a spreadsheet upon adjusting the pixel size of the post-processed map. The correct pixel size of 2.83 Å/pixel was estimated from plotting the correlation values and estimating the peak by eye. We then ran another post-processing job to determine the FSC and to obtain the filtered map.

The final map contains 3,801 particles. The luminal filament average was lowpass filtered to the determined resolution and sharpened with a B-factor of −500 Å^2 in Relion 3.1. The helical symmetry of the luminal filament average (−161.8° twist, 28.5 Å rise) was determined using the relion_helix_toolbox "search" option, with an outer diameter of 120 Å, a rise of 1–40 Å and a twist of −100 to −200°.

Generation of a *Drosophila* cofilactin model

To generate a *Drosophila* cofilactin model, we predicted the structure of *Drosophila* cofilin (Uniprot ID P45594) together with *Drosophila* actin (Act5C, Uniprot ID P10987) using Alphafold 2-Multimer (preprint: Evans *et al*, 2022) through a local installation of Colabfold 1.2.0 (Mirdita *et al*, 2022). The actin moiety in the predicted cofilin-actin unit resembled the actin structure from a previous cofilactin filament model made of chicken cofilin and rabbit actin (PDB 5YU8) (Tanaka *et al*, 2018). The prediction of cofilin also matched the chicken cofilin structure from this model, except for a loop that is absent in *Drosophila* cofilin due to its shorter length (148 residues compared to 166 residues in chicken cofilin).

We aligned the predicted cofilin-actin unit with actin from the cofilactin model (PDB 5YU8) using the "matchmaker" tool in Chimera. We assembled a *Drosophila* cofilactin filament by repeating this procedure for 8 consecutive cofilin-actin units and then removing the two cofilin moieties at the pointed end. The resulting model contains eight actin and six cofilin units. We removed the first 6 N-terminal residues (MCDEEV) and residues 42–50 (QGVMVGMGQ) of the DNAse loop of actin, as these residues were absent from the previous PDB model 5YU8 on which we based our cofilactin model. We truncated side chains in Phenix version 1.10-2155 (Adams *et al*, 2010) using the phenix.pdbtools option remove="protein and sidechain".

To assess the fit of the *Drosophila* cofilactin model in our subtomogram average structure of the luminal filament, we first generated a density map from the model using the Chimera "molmap" function at a resolution of 17 Å. We then fit this into our subtomogram average structure and obtained a score of 0.963 for atoms inside the density.

Classification of luminal filaments from control and cofilin knock-down samples

To determine the percentages of particles with cofilactin, bare f-actin and other morphologies with subtomogram classification, we manually picked particles and exported them to Dynamo filament with torsion models and subsequently to Relion 3 star files following workflows described in the above sections. The initial particle number was 9,679 from four biological replicates (dataset 9: 4,168 particles, dataset 10: 1,778 particles, dataset 11: 2,743 particles, and dataset 12: 990 particles), each containing control (6,271 particles across all datasets) and cofilin knock-down (3,408 particles across all datasets) samples.

We first generated a set of uniformly oriented particles. For this, particles were jointly 3D classified into 20 classes with helical symmetry (−165° twist, 29 Å rise) to select cofilactin, bare f-actin, and other classes. Particles from cofilactin and other classes were pooled while particles from bare f-actin classes were saved separately. We iteratively classified these subsets to obtain classes with different filament polarities and rotated particles from classes with opposite orientation by −180°. To do this, we first converted the star files of opposite-polarity classes into Dynamo tables (dynamo2m package) and then motive lists, rotated particles using the subTOM package, converted the motive lists back into Dynamo tables and then star files (dynamo2m package), and finally replaced the angles columns (AngleRot, AngleTilt, AnglePsi) from the "old" star file with the values determined after rotation using the Starparser package. The uniform orientation of particles was assessed by eye from repeated 3D classification with helical symmetry.

 © 2023 MRC Laboratory of Molecular Biology and The Authors

Once particles were uniformly oriented, we initiated classification to determine the percentage of particles in each class. For this, we performed nine 3D classifications with 5, 10, or 20 classes and each with a helical rise of −162°, −165°, −167°, and 25 iterations. These values for the helical rise correspond to those determined for cofilactin (−162°) or f-actin (−167°). −165° was included to represent a value in between the two. Particles were aligned to a featureless, lowpass filtered (60 Å) rod with a cylindrical soft-edged mask. For each classification, we determined by eye which classes resembled cofilactin, bare f-actin or "other" morphologies. An example of the classification result (helical twist: −165°, 10 classes), the assigned morphology and the quantification from this individual classification run can be found in Fig EV3K and L. We used a custom MATLAB script to determine the number of particles from control or cofilin knock-down cells from each biological replicate in each category of classes (cofilactin-, bare f-actin, other). From this, we calculated the percentages for each biological replicate and control/cofilin knock-down samples for each classification run. The percentages were averaged (s.d.s were in between 0.7% and 14.4%) for each biological replicate and are shown as spheres (control dsRNA) or triangles (cofilin dsRNA) in Fig 3L. Unpaired *t*-tests were used to compare results from classification into cofilactin, bare f-actin and other classes between control and cofilin knock-down samples. Individual class averages representing cofilactin, bare f-actin and other morphologies are shown in the top panel of Fig 3L.

Data availability

The datasets and computer code produced in this study are available in the following databases: (i) CryoET raw data and filtered, bin4 tomograms: EMPIAR (EMPIAR-11450 (https://www.ebi.ac.uk/empiar/EMPIAR-11450/), EMPIAR-11451 (https://www.ebi.ac.uk/empiar/EMPIAR-11451/), EMPIAR-11452 (https://www.ebi.ac.uk/empiar/EMPIAR-11452/), EMPIAR-11453 (https://www.ebi.ac.uk/empiar/EMPIAR-11453/) (Iudin *et al*, 2023), (ii) Representative tomograms and subtomogram average structure: EMDB (wild-type: EMD-16685 (http://www.ebi.ac.uk/pdbe/entry/EMD-16685), EMD-16693 (http://www.ebi.ac.uk/pdbe/entry/EMD-16693), dTAT KO: EMD-16695 (http://www.ebi.ac.uk/pdbe/entry/EMD-16695), luminal filaments in wild-type (EMD-16720) (http://www.ebi.ac.uk/pdbe/entry/EMD-16720) and cofilin knock-down (EMD-16800 (http://www.ebi.ac.uk/pdbe/entry/EMD-16800) and EMD-16811 (http://www.ebi.ac.uk/pdbe/entry/EMD-16811)), luminal filament subtomogram average: EMD-16877 (http://www.ebi.ac.uk/pdbe/entry/EMD-16877)), (iii) *Drosophila* cofilactin model: PDB (PDB 8OH4) (http://www.rcsb.org/pdb/explore/explore.do?structureId=8OH4), (iv) Computer code: GitHub, https://github.com/carterlablmb/Ventura-Rogers-Carter-2023.git, (v) Additional files: Biostudies (S-BSST1048) (https://www.ebi.ac.uk/biostudies/studies/S-BSST1048) (Sarkans *et al*, 2018).

Expanded View for this article is available online.

Acknowledgements

We thank Tom Dendooven and Alister Burt for advice with subtomogram averaging, Emmanuel Derivery and Alice Bittlestone for assistance with S2 cell culture, the MRC LMB Electron Microscopy Facility, Scientific Computing and Light Microscopy Facility for access and support. We acknowledge Anna Marie Sokac and Buzz Baum for gifts of the *Drosophila* cofilin and P-cofilin antibodies. We thank Simon Bullock and members of the Carter laboratory for discussions. This study was supported by the Medical Research Council (MRC_UP_A025_1011) and Wellcome Trust (210711/Z/18/Z) grants to APC, by the National Institute of Neurological Disorders and Stroke grant (R21NS125795) to SLR and by a PhD studentship from the Cambridge Trust to CVS.

Author contributions

Camilla Ventura Santos: Conceptualization; data curation; formal analysis; funding acquisition; validation; investigation; visualization; methodology; writing – original draft; writing – review and editing. **Stephen L Rogers:** Conceptualization; resources; funding acquisition; writing – review and editing. **Andrew P Carter:** Conceptualization; supervision; funding acquisition; writing – original draft; writing – review and editing.

Disclosure and competing interests statement

The authors declare that they have no conflict of interest.

References

Adams PD, Afonine PV, Bunkóczi G, Chen VB, Davis IW, Echols N, Headd JJ, Hung LW, Kapral GJ, Grosse-Kunstleve RW *et al* (2010) PHENIX: a comprehensive Python-based system for macromolecular structure solution. *Acta Crystallogr D Biol Crystallogr* 66: 213 – 221

Agnew BJ, Minamide LS, Bamburg JR (1995) Reactivation of phosphorylated actin depolymerizing factor and identification of the regulatory site. *J Biol Chem* 270: 17582 – 17587

Andrianantoandro E, Pollard TD (2006) Mechanism of actin filament turnover by severing and nucleation at different concentrations of ADF/cofilin. *Mol Cell* 24: 13 – 23

Arber S, Barbayannis FA, Hanser H, Schnelder C, Stanyon CA, Bernards O, Caroni P (1998) Regulation of actin dynamics through phosphorylation of cofilin by LIM-kinase. *Nature* 393: 805 – 809

Atherton J, Stouffer M, Francis F, Moores CA (2022) Visualising the cytoskeletal machinery in neuronal growth cones using cryo-electron tomography. *J Cell Sci* 135: jcs259234

Austin Elam W, Cao W, Kang H, Huehn A, Hocky GM, Prochniewicz E, Schramm AC, Negrón K, Garcia J, Bonello TT *et al* (2017) Phosphomimetic S3D cofilin binds but only weakly severs actin filaments. *J Biol Chem* 292: 19565 – 19579

Bassett AR, Tibbit C, Ponting CP, Liu JL (2014) Mutagenesis and homologous recombination in *Drosophila* cell lines using CRISPR/Cas9. *Biol Open* 3: 42 – 49

Bouchet-Marquis C, Zuber B, Glynn A-M, Eltsov M, Grabenbauer M, Goldie KN, Thomas D, Frangakis AS, Dubochet J, Chrétien D (2007) Visualization of cell microtubules in their native state. *Biol Cell* 99: 45 – 53

Burton PR (1984) Luminal material in microtubules of frog olfactory axons: structure and distribution. *J Cell Biol* 99: 520 – 528

Castaño-Díez D, Kudryashev M, Arheit M, Stahlberg H (2012) Dynamo: a flexible, user-friendly development tool for subtomogram averaging of cryo-EM data in high-performance computing environments. *J Struct Biol* 178: 139 – 151

Chakraborty S, Mahamid J, Baumeister W (2020) Cryoelectron tomography reveals nanoscale organization of the cytoskeleton and its relation to microtubule curvature inside cells. *Structure* 28: 991 – 1003

Chakraborty S, Martinez-sanchez A, Beck F, Toro-nahuelpan M (2022) Cryo-electron tomography reveals enrichment and identifies microtubule

© 2023 MRC Laboratory of Molecular Biology and The Authors

lumenal particles in neuronal differentiation. *bioRxiv* https://doi.org/10.1101/2022.07.28.501854 [PREPRINT]

Chan AY, Bailly M, Zebda N, Segall JE, Condeelis JS (2000) Role of cofilin in epidermal growth factor-stimulated actin polymerization and lamellipod protrusion. *J Cell Biol* 148: 531 – 542

Cherbas L, Gong L (2014) Cell lines. *Methods* 68: 74 – 81

Cuveillier C, Delaroche J, Seggio M, Gory-fauré S, Bosc C, Denarier E, Bacia M, Schoehn G, Mohrbach H, Kulić I *et al* (2020) MAP6 is an intraluminal protein that induces neuronal microtubules to coil. *Sci Adv* 6: eaaz4344

del Castillo U, Winding M, Lu W, Gelfand VI (2015) Interplay between kinesin-1 and cortical dynein during axonal outgrowth and microtubule organization in *Drosophila* neurons. *Elife* 4: 1 – 20

del Castillo U, Müller HAJ, Gelfand VI (2020) Kinetochore protein Spindly controls microtubule polarity in *Drosophila* axons. *Proc Natl Acad Sci USA* 117: 12155 – 12163

Edwards KA, Montague RA, Shepard S, Edgar BA, Erikson RL, Kiehart DP (1994) Identification of *Drosophila* cytoskeletal proteins by induction of abnormal cell shape in fission yeast. *Proc Natl Acad Sci USA* 91: 4589 – 4593

Endo M, Ohashi K, Sasaki Y, Goshima Y, Niwa R, Uemura T, Mizuno K (2003) Control of growth cone motility and morphology by LIM kinase and slingshot via phosphorylation and dephosphorylation of cofilin. *J Neurosci* 23: 2527 – 2537

Evans R, O'Neill M, Pritzel A, Antropova N, Senior A, Green T, Žídek A, Bates R, Blackwell S, Yim J *et al* (2022) Protein complex prediction with AlphaFold-Multimer. *bioRxiv* https://doi.org/10.1007/978-1-61779-361-5_16 [PREPRINT]

Figard L, Zheng L, Biel N, Xue Z, Seede H, Coleman S, Golding I, Sokac AM (2019) Cofilin-mediated actin stress response is maladaptive in heat-stressed embryos. *Cell Rep* 26: 3493 – 3501

Foster HE, Ventura Santos C, Carter AP (2022) A cryo-ET survey of microtubules and intracellular compartments in mammalian axons. *J Cell Biol* 221: e202103154

Garvalov BK, Zuber B, Bouchet-Marquis C, Kudryashev M, Gruska M, Beck M, Leis A, Frischknecht F, Bradke F, Baumeister W *et al* (2006) Luminal particles within cellular microtubules. *J Cell Biol* 174: 759 – 765

Gunsalus KC, Bonaccorsi S, Williams E, Verni F, Gatti M, Goldberg ML (1995) Mutations in twinstar, a *Drosophila* gene encoding a cofilin/ADF homologue, result in defects in centrosome migration and cytokinesis. *J Cell Biol* 131: 1243 – 1259

Hagen WJH, Wan W, Briggs JAG (2017) Implementation of a cryo-electron tomography tilt-scheme optimized for high resolution subtomogram averaging. *J Struct Biol* 197: 191 – 198

Hylton RK, Heebner JE, Grillo MA, Swulius MT (2022) Cofilactin filaments regulate filopodial structure and dynamics in neuronal growth cones. *Nat Commun* 13: 1 – 11

Iudin A, Korir PK, Somasundharam S, Weyand S, Cattavitello C, Fonseca N, Salih O, Kleywegt GJ, Patwardhan A (2023) EMPIAR: the electron microscopy public image archive. *Nucleic Acids Res* 51: D1503 – D1511

Jovceva E, Larsen MR, Waterfield MD, Baum B, Timms JF (2007) Dynamic cofilin phosphorylation in the control of lamellipodial actin homeostasis. *J Cell Sci* 120: 1888 – 1897

Kural C, Kim H, Syed S, Goshima G, Gelfand VI, Selvin PR (2005) Kinesin and dynein move a peroxisome *in vivo*: a tug-of-war or coordinated movement? *Science* 308: 1469 – 1472

Lee DH, Goldberg AL (1998) Proteasome inhibitors: valuable new tools for cell biologists. *Trends Cell Biol* 8: 397 – 403

Lu W, Del Castillo U, Gelfand VI (2013) Organelle transport in cultured *Drosophila* cells: S2 cell line and primary neurons. *J Vis Exp* 81: 50838

Maciver SK, Zot HG, Pollard TD (1991) Characterization of actin filament severing by actophorin from *Acanthamoeba castellanii*. *J Cell Biol* 115: 1611 – 1620

Mastronarde DN (2005) Automated electron microscope tomography using robust prediction of specimen movements. *J Struct Biol* 152: 36 – 51

McGough A, Pope B, Chiu W, Weeds A (1997) Cofilin changes the twist of F-actin: implications for actin filament dynamics and cellular function. *J Cell Biol* 138: 771 – 781

Meberg PJ, Ono S, Minamide LS, Takahashi M, Bamburg JR (1998) Actin depolymerizing factor and cofilin phosphorylation dynamics: response to signals that regulate neurite extension. *Cell Motil Cytoskeleton* 39: 172 – 190

Minamide LS, Striegl AM, Boyle JA, Meberg PJ, Bamburg JR (2000) Neurodegenerative stimuli induce persistent ADF/cofilin-actin rods that disrupt distal neurite function. *Nat Cell Biol* 2: 628 – 636

Mirdita M, Schütze K, Moriwaki Y, Heo L, Ovchinnikov S, Steinegger M (2022) ColabFold: making protein folding accessible to all. *Nat Methods* 19: 679 – 682

Nishida E, Maekawa S, Muneyuki E, Sakai H (1984) Action of a 19K protein from porcine brain on actin polymerization: a new functional class of actin-binding proteins. *J Biochem* 95: 387 – 398

Nishida E, Iida K, Yonezawa N, Koyasu S, Yahara I, Sakai H (1987) Cofilin is a component of intranuclear and cytoplasmic actin rods induced in cultured cells. *Proc Natl Acad Sci USA* 84: 5262 – 5266

Niwa R, Nagata-Ohashi K, Takeichi M, Mizuno K, Uemura T (2002) Control of actin reorganization by slingshot, a family of phosphatases that dephosphorylate ADF/cofilin. *Cell* 108: 233 – 246

Nogales E, Whittaker M, Milligan RA, Downing KH (1999) High-resolution model of the microtubule. *Cell* 96: 79 – 88

Ohta Y, Nishida E, Sakai H, Miyamoto E (1989) Dephosphorylation of cofilin accompanies heat shock-induced nuclear accumulation of cofilin. *J Biol Chem* 264: 16143 – 16148

Paul DM, Mantell J, Borucu U, Coombs J, Surridge KJ, Squire JM, Verkade P, Dodding MP (2020) *In situ* cryo-electron tomography reveals filamentous actin within the microtubule lumen. *J Cell Biol* 219: e201911154

Pettersen EF, Goddard TD, Huang CC, Couch GS, Greenblatt DM, Meng EC, Ferrin TE (2004) UCSF Chimera - a visualization system for exploratory research and analysis. *J Comput Chem* 25: 1605 – 1612

Qu K, Glass B, Dolezal M, Schur FKM, Murciano B, Rein A, Rumlová M, Ruml T, Kräusslich HG, Briggs JAG (2018) Structure and architecture of immature and mature murine leukemia virus capsids. *Proc Natl Acad Sci USA* 115: E11751 – E11760

Rogers SL, Rogers GC (2008) Culture of *Drosophila* S2 cells and their use for RNAi-mediated loss-of-function studies and immunofluorescence microscopy. *Nat Protoc* 3: 606 – 611

Rogers SL, Wiedemann U, Stuurman N, Vale RD (2003) Molecular requirements for actin-based lamella formation in *Drosophila* S2 cells. *J Cell Biol* 162: 1079 – 1088

Sarkans U, Gostev M, Athar A, Behrangi E, Melnichuk O, Ali A, Minguet J, Rada JC, Snow C, Tikhonov A *et al* (2018) The BioStudies database-one stop shop for all data supporting a life sciences study. *Nucleic Acids Res* 46: D1266 – D1270

Scheres SHW (2012) RELION: implementation of a Bayesian approach to cryo-EM structure determination. *J Struct Biol* 180: 519 – 530

Schindelin J, Arganda-Carreras I, Frise E, Kaynig V, Longair M, Pietzsch T, Preibisch S, Rueden C, Saalfeld S, Schmid B *et al* (2012) Fiji: an open-source platform for biological-image analysis. *Nat Methods* 9: 676 – 682

 © 2023 MRC Laboratory of Molecular Biology and The Authors

Schneider I (1972) Cell lines derived from late embryonic stages of *Drosophila melanogaster*. *J Embryol Exp Morphol* 27: 353 – 365

Sosa H, Chrétien D (1998) Relationship between Moire patterns, tubulin shape, and microtubule polarity. *Cell Motil Cytoskeleton* 40: 38 – 43

Tanaka K, Takeda S, Mitsuoka K, Oda T, Kimura-Sakiyama C, Maéda Y, Narita A (2018) Structural basis for cofilin binding and actin filament disassembly. *Nat Commun* 9: 1 – 3

Tang G, Peng L, Baldwin PR, Mann DS, Jiang W, Rees I, Ludtke SJ (2007) EMAN2: an extensible image processing suite for electron microscopy. *J Struct Biol* 157: 38 – 46

Tegunov D, Cramer P (2019) Real-time cryo-electron microscopy data preprocessing with Warp. *Nat Methods* 16: 1146 – 1152

Thastrup O, Cullen PJ, Drobak BK, Hanley MR, Dawson AP (1990) Thapsigargin, a tumor promoter, discharges intracellular Ca^{2+} stores by specific inhibition of the endoplasmic reticulum Ca^{2+}-ATPase. *Proc Natl Acad Sci USA* 87: 2466 – 2470

Yang N, Higuchi O, Ohashi K, Nagata K, Wada A, Kangawa K, Nishida E, Mizuno K (1998) Cofilin phosphorylation by LIM-kinase 1 and its role in Rac-mediated actin reorganization. *Nature* 393: 809 – 812

Zebda N, Bernard O, Bailly M, Welti S, Lawrence DS, Condeelis JS (2000) Phosphorylation of ADF/cofilin abolishes EGF-induced actin nucleation at the leading edge and subsequent lamellipod extension. *J Cell Biol* 151: 1119 – 1127

Zivanov J, Nakane T, Forsberg BO, Kimanius D, Hagen WJH, Lindahl E, Scheres SHW (2018) New tools for automated high-resolution cryo-EM structure determination in RELION-3. *Elife* 7: 1 – 22

License: This is an open access article under the terms of the Creative Commons Attribution License, which permits use, distribution and reproduction in any medium, provided the original work is properly cited.

© 2023 MRC Laboratory of Molecular Biology and The Authors

Expanded View Figures

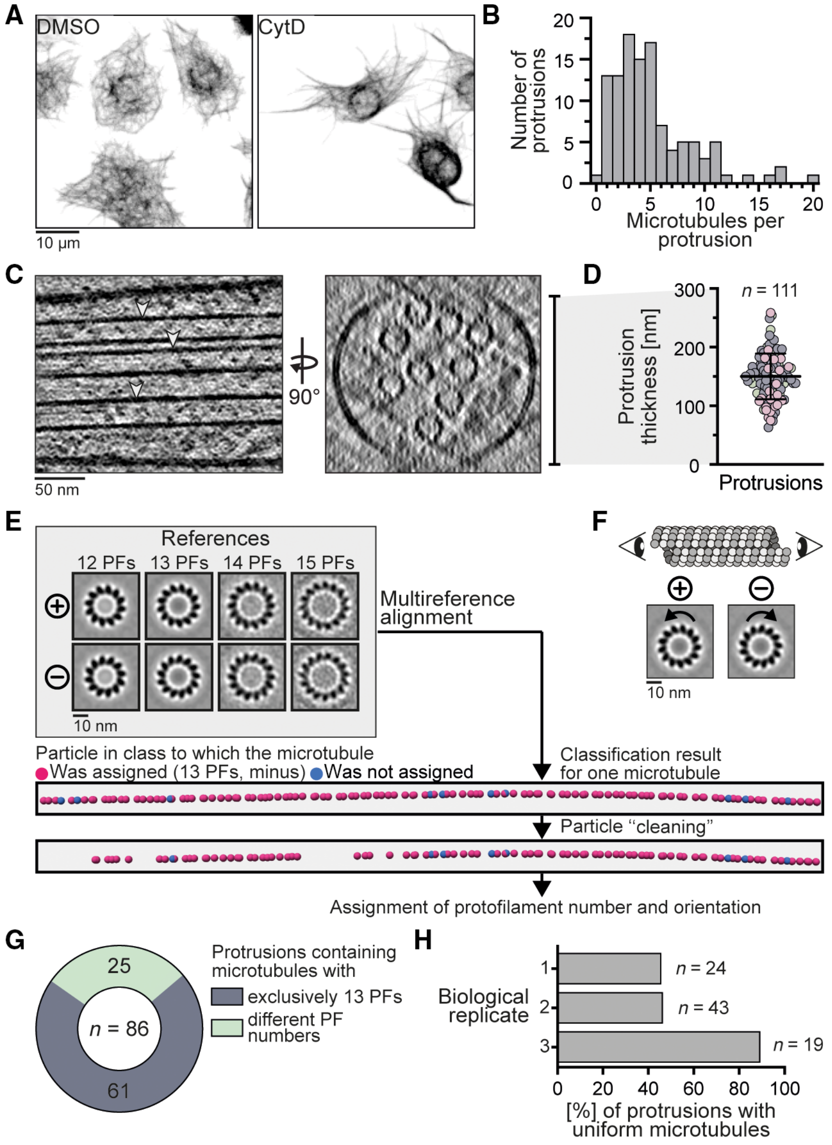

Figure EV1. Analysis of microtubules in induced S2 cell protrusions.

A Immunofluorescence staining of α-tubulin in S2 cells shows that CytD treatment induces the formation of protrusions which contain microtubules. Control cells treated with a vehicle (DMSO) are shown on the left.

B Histogram of the microtubule number per protrusion in targeted regions quantified from tomograms.

C Tomogram slices of a protrusion in side-view (left) and cross-section (right) showing that microtubules (arrowheads) form parallel arrays.

D Protrusion thicknesses (150.1 ± 39.0 nm, mean ± s.d., N = 3, colored accordingly) measured from cross-sections of tomograms exemplified by the bar in (C).

E Classification workflow to determine microtubule protofilament number and orientation. Projections of references used in the multireference alignment (top, projections of plus-end facing microtubules are also used in Fig 1D, those of 13 protofilament microtubules are reused in (F)) and particle positions/classes from a representative microtubule (bottom) before and after removal of particles based on their cross-correlation score ("cleaning") are shown.

F Cartoon and exemplary projections of microtubule subtomogram averages showing that microtubules appear to rotate in anti-clockwise and clockwise directions when viewed from the plus and minus end, respectively (Sosa & Chrétien, 1998). Axial 13 protofilament microtubule projections were reused from Fig 1D and (E).

G Pie chart showing that the majority but not all protrusions contained exclusively 13 protofilament microtubules. 25 protrusions had microtubules with two or three different protofilament numbers.

H Percentages of protrusions containing uniformly oriented microtubules from three biological replicates show that microtubule uniformity can be variable across datasets. N, n: number of biological replicates (N), analyzed protrusions (n).

Source data are available online for this figure.

 © 2023 MRC Laboratory of Molecular Biology and The Authors

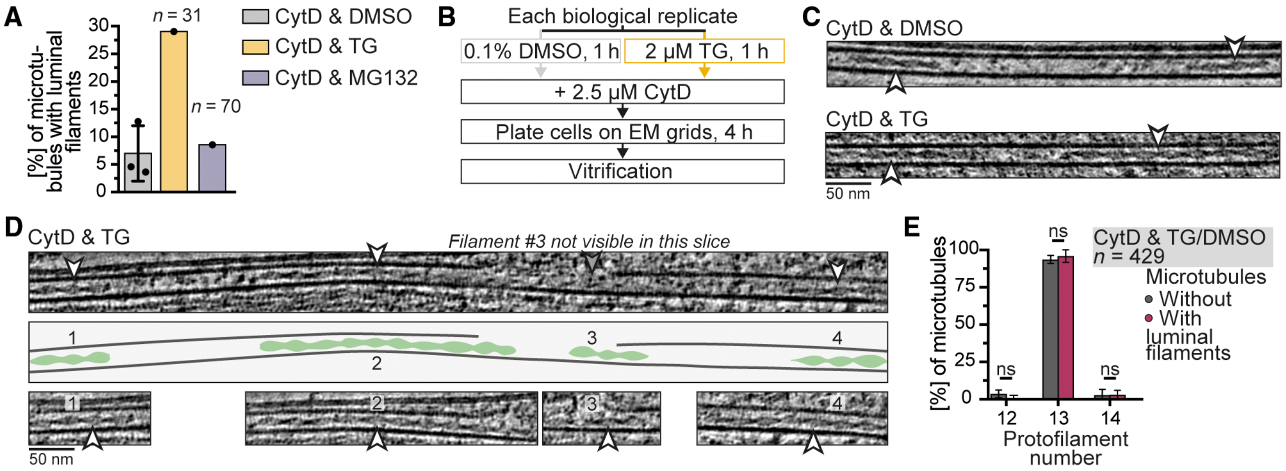

Figure EV2. Analysis of filaments inside the microtubule lumen.

A Percentage of microtubules with at least one luminal filament after treatment with CytD & TG (29.0%) or CytD & MG132 (8.6%) in a preliminary experiment (N = 1). The control (CytD & DMSO, 7.0 ± 5.0%) from Fig 2C is shown for comparison.

B Workflow for the preparation of biological replicates that were treated with CytD & DMSO/TG and used for the analysis of luminal filaments.

C Tomogram slices of microtubules with two luminal filaments (white arrowheads) from samples with indicated treatments.

D Tomogram slices and cartoon of a microtubule that contains four luminal filaments. Top panel shows an overview image of the microtubule where three out of four luminal filaments are visible (arrowheads). The third filament is not clear in this tomographic slice. Middle panel shows a cartoon of the microtubule and luminal filaments. The four luminal filaments are also depicted in the bottom panel with numbers corresponding to those in the cartoon. The example is from cells treated with CytD & TG.

E Percentage of microtubules without (gray) or with (red) luminal filaments that have 12 (3.6 ± 2.6% without, 1.0 ± 1.7% with), 13 (93.6 ± 2.6% without, 96.0 ± 4.3% with) or 14 (2.8 ± 3.8% without, 3.1 ± 2.9% with) protofilaments from CytD & DMSO and CytD & TG treated cells showing no significant difference (mean ± s.d., N = 3, 2-way ANOVA test with comparisons between microtubules of each protofilament number with and without luminal filaments: 12: P = 0.53, 13: P = 0.85, 14: P > 0.99, all ns). N, n: number of biological replicates (N), analyzed microtubules (n).

Source data are available online for this figure.

Figure EV3. Structural analysis of luminal filaments.

A, B Fourier transforms of the luminal filament (A) and cytoplasmic f-actin (B) in Fig 3A and B. Layer lines and corresponding frequencies are indicated. Left panels show a slice (A) or projection (B) of the filament used to generate Fourier transform.

C Cross-over distances of luminal filaments (27.4 ± 0.1 nm, N = 3) and cytoplasmic f-actin (35.7 ± 2.4 nm, N = 4) measured from Fourier transforms showing a significant difference (unpaired t-test, P = 0.0022, **).

D Workflow for subtomogram averaging of luminal filaments.

E Positions and orientations of particles from three microtubules (gray) and luminal filaments (red/orange) in a representative protrusion. Arrows point towards the plus/barbed ends of the filaments. Luminal filament 1 is oriented with its barbed end towards the microtubule minus end while luminal filaments 2 and 3 have their barbed ends oriented towards the microtubule plus end.

F Quantification showing that 46.8 ± 5.5% of luminal filaments had their barbed (plus) end pointing towards the plus end of the surrounding microtubule (N = 3).

G Averages after alignments with a mask (green, left) including the luminal filament (top) or the microtubule (bottom). In the first case, the luminal filament but not the surrounding microtubule was well resolved. In the latter case, the microtubule but not the luminal filament was well resolved.

H Fourier shell correlation of the luminal filament subtomogram average.

I, J Tomogram slices of individual (I) and bundled (J) putative cofilactin filaments in the cytoplasm of S2 protrusions from samples with indicated treatments.

K, L Exemplary subtomogram classification (K) and quantification (L) of luminal filaments from control and cofilin knock-down cells. Classes assigned to cofilactin, bare f-actin and other morphologies are labeled green, blue and gray, respectively. Averages of the percentages of particles with each morphology for each N are shown in Fig 3L.

M Percentage of luminal filament length resembling cofilactin (89.8 ± 7.2% control, 25.2 ± 21.8% cofilin dsRNA) assessed from visual inspection of the tomograms showing a decrease upon cofilin knock-down (N = 4, unpaired t-test, P = 0.0013, **). Panel on the right shows examples of cofilactin and non-cofilactin morphologies. 41.1 and 22.1 μm luminal filament length were sampled in control and cofilin knock-down cells, respectively.

N Tomogram slices of luminal filaments from cofilin knock-down cells with morphologies different from cofilactin or bare f-actin.

O Percentage of microtubules with luminal filaments in control (17.8 ± 7.7%) or cofilin (11.7 ± 3.4%) knock-down cells showing a non-significant reduction (N = 4, unpaired t-test, P = 0.2011, ns).

P Tomogram slice and cartoon of a luminal filament that transitions from a cofilactin (left) to a non-cofilactin (right) morphology.

Q Examples of linking densities (white arrowheads) between luminal filaments and the surrounding microtubule wall. Graphs and numbers show mean ± s.d. N, n: number of biological replicates (N) and analyzed filaments (n).

Source data are available online for this figure.

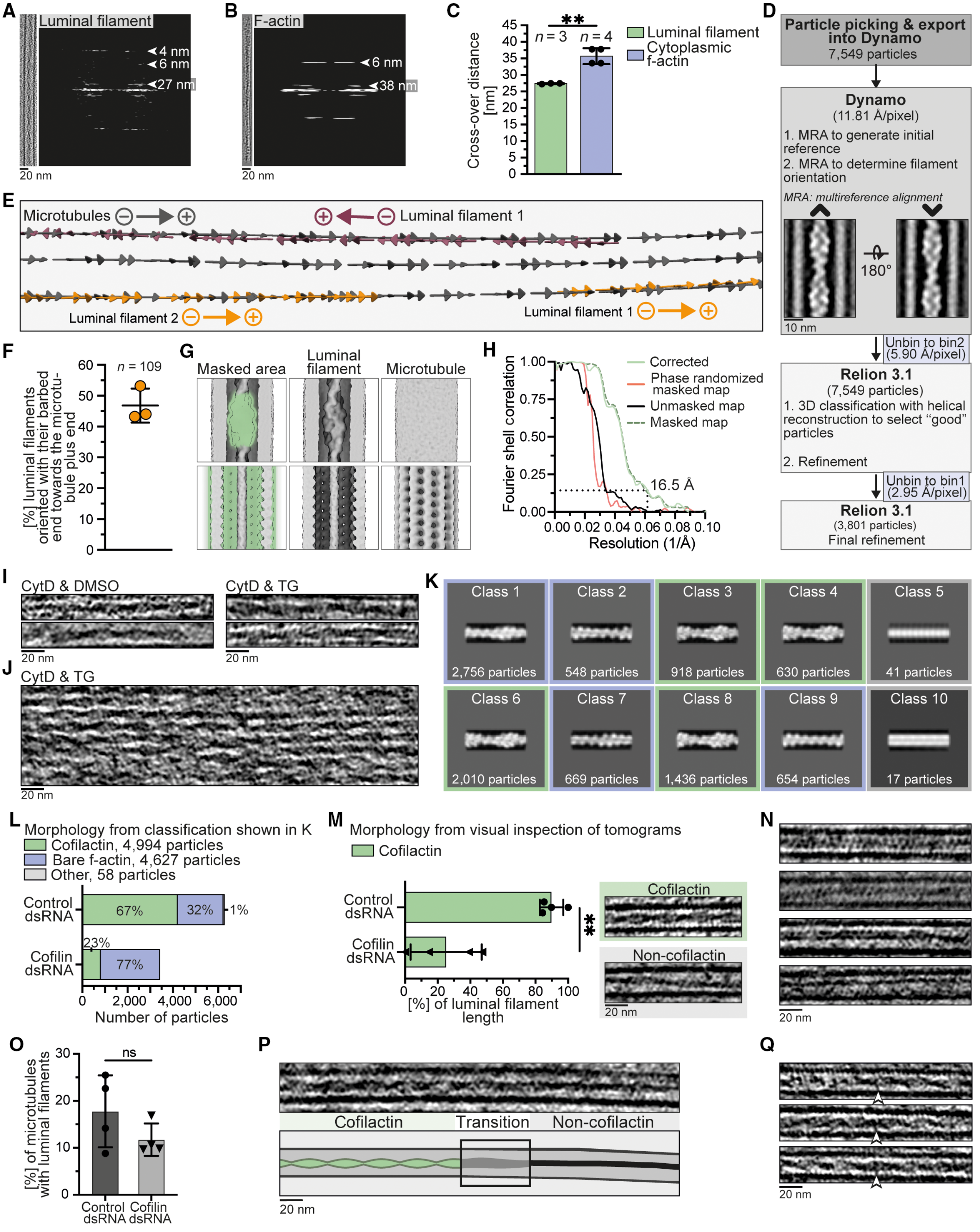

Figure EV3.

© 2023 MRC Laboratory of Molecular Biology and The Authors

Appendix

Table of contents

Appendix Figure S1. Validation of the microtubule orientation assignment. 2

Appendix Figure S2. Validation of the *Drosophila* α-tubulin acetylase (dTAT) CRISPR knock-out. 3

Appendix Table S1. EMDB entries of actin together with actin-binding proteins solved by helical reconstruction. 4

Appendix Table S2. Acquisition and processing parameters for datasets 1 – 4 (EMPIAR-11450). 5

Appendix Table S3. Acquisition and processing parameters for datasets 5 – 7 (EMPIAR-11451) and dataset 8 (EMPIAR-11452). 6

Appendix Table S4. Acquisition and processing parameters for datasets 9 – 12 (EMPIAR-11453). 7

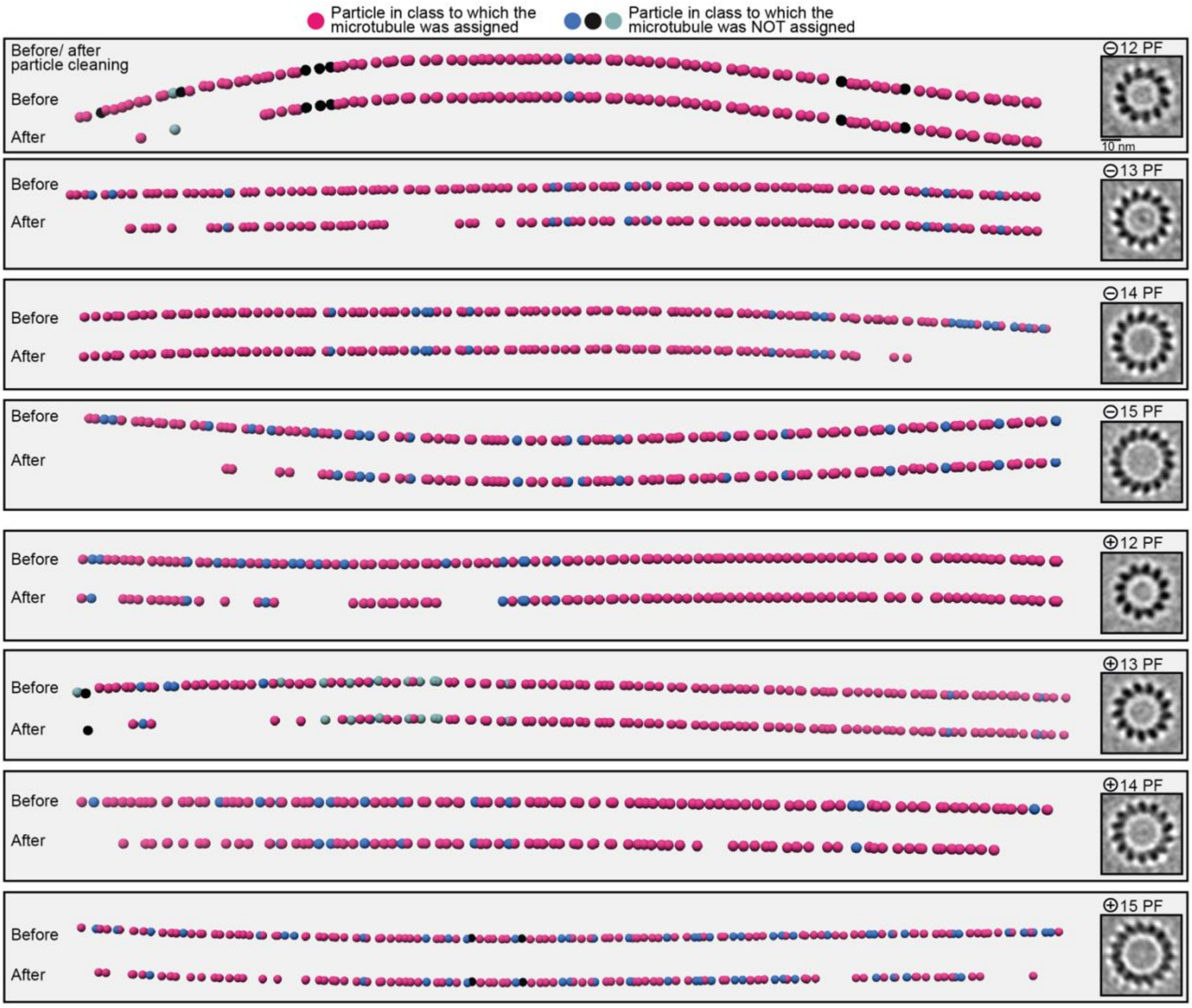

Appendix Figure S1. Validation of the microtubule orientation assignment. Microtubule particle positions and classes are shown for 8 representative microtubules with 12, 13, 14 or 15 protofilaments facing the minus and the plus ends. Pink spheres represent particles in the class, to which the microtubule was assigned. Blue, black and green spheres show particles in classes, to which the microtubule was not assigned. The particle class distribution before (top, 'Before') and after (bottom, 'After') removal of 20% of particles based on their cross-correlation scores ('cleaning') are shown for each microtubule. Projections of subtomogram averages of each of the microtubules before cleaning are shown on the right.

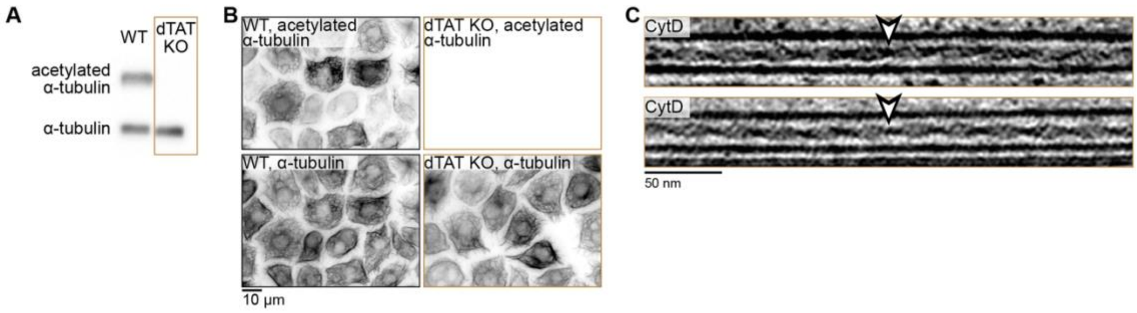

Appendix Figure S2. Validation of the *Drosophila* α-tubulin acetylase (dTAT) CRISPR knock-out. A) Western blot of wild-type (WT, left) or dTAT knock-out (KO, right) cells showing that dTAT KO cells lack acetylated α-tubulin (top). α-tubulin (bottom) was used as loading control. B) Immunofluorescence of WT (left) and dTAT KO (right) cells stained for acetylated α-tubulin (top) or α-tubulin (bottom) showing that dTAT KO cells lack acetylated α-tubulin. C) Tomogram slices of luminal filaments (white arrowheads) in dTAT KO cells showing that they have similar appearance to luminal filaments found in WT cells (Fig. 2A, B).

FAMILY OF ACTIN BINDING PROTEIN	EMD ENTRY NAME	EMD ID	HELICAL RISE [Å]	HELICAL TWIST [°]
**ACTIN (BARE)**	Helical ADP-F-actin	27114	28.1	-166.7
**ACTININ**	alpha-actinin CH1 bound to f-actin	5170	27.7	-166.8
**BETA-SPECTRIN**	F-actin with beta-III-spectrin actin-binding domain	8886	27.2	-166.9
**CATENIN**	alphaE-catenin - F-actin	21925	27.4	-166.9
**CORONIN**	coronin bound f-actin	6101	28.2	-166.3*
**FILAMIN**	FLNaABD-WT bound to phalloidin F-actin	7833	28.1	-166.8
**PLASTIN**	F-actin/Plastin2-ABD2 complex	21155	28.0	-166.5*
**MYOSIN**	cardiac myosin binding protein C C2 domain with f-actin	23497	27.3	-166.5
**TROPOMYOSIN**	F-actin-tropomyosin	6124	27.5	-166.4*
**VINCULIN**	vinculin-actin	6446	27.8	-166.8*
**COFILIN**	cofilactin	6844	27.6	-162.1
**THIS STUDY**	Subtomogram averaging structure of cofilactin filament inside microtubule lumen of Drosophila S2 cell protrusion	16877	28.0	-161.8

Appendix Table S1. EMDB entries of actin together with actin-binding proteins solved by helical reconstruction. Helical twist values marked with a star were deposited as positive values but are displayed with a negative sign because they describe left-handed helix symmetries. Helical parameters were rounded to one decimal.

MICROTUBULE ANALYSIS	**BIOLOGICAL REPLICATE 1**	**BIOLOGICAL REPLICATE 2**		**BIOLOGICAL REPLICATE 3**
**GRID ID**	**DZ4**	**DY2**	**DZ1**	**FB3/FB4**
**DATASET**	**dataset 1**	**dataset 2**	**dataset 3**	**dataset 4**
**GENOTYPE**	WT	WT		WT
**SAMPLE CONDITIONS**	4 h 2.5 µM CytD	4 h 2.5 µM CytD		4 h 5 µM CytD
**INCREMENTS/ NUMBER OF TILTS**	2°/ 61	2°/ 61	3°/ 41	3°/ 41
**FRAMES PER TILT IMAGE**	10	10	14	14
**PIXEL SIZE [Å/PIXEL], DETECTOR**	2.952, K2	2.952, K2		2.952, K2
**TOTAL DOSE [e^-/Å^2]**	123.44	121.68	123.57	122.25
**PROGRAM USED FOR TILT ALIGNMENT**	dautoalign and IMOD	IMOD		IMOD
**ACQUIRED TILT SERIES**	35	22	39	24
**ANALYSED TOMOGRAMS**	33	18	36	22
**NUMBER OF ANALYZED PROTRUSIONS**	34	56		22
**NUMBER OF ANALYZED MICROTUBULES**	109	299		166

Appendix Table S2. Acquisition and processing parameters for datasets 1 – 4 (EMPIAR-11450). These datasets were used for quantification and subtomogram averaging of microtubules in S2 cells.

CYTD & DMSO AND CYTD & TG	BIOLOGICAL REPLICATE 1		BIOLOGICAL REPLICATE 2		BIOLOGICAL REPLICATE 3		DTAT KNOCK-OUT
**ACQUISITION DATE/ GRID ID**	**17.06.2022**		**20.07.2022**		**21.07.2021**		**FE2/FE4**
**DATASET**	**dataset 5**		**dataset 6**		**dataset 7**		**dataset 8**
**GENOTYPE**	WT		WT		WT		dTAT KO
**SAMPLE CONDITIONS**	5 h 0.1% (v/v) DMSO, 4 h 2.5 µM CytD	5 h 2 µM TG, 4 h 2.5 µM CytD	5 h 0.1% (v/v) DMSO, 4 h 2.5 µM CytD	5 h 2 µM TG, 4 h 2.5 µM CytD	5 h 0.1% (v/v) DMSO, 4 h 2.5 µM CytD	5 h 2 µM TG, 4 h 2.5 µM	4 h 5 µM CytD
**INCREMENTS/ NUMBER OF TILTS**	3°/ 41		3°/ 41		3°/ 41		3°/ 41
**FRAMES PER TILT IMAGE**	14		14		14		14
**PIXEL SIZE [Å/PIXEL], DETECTOR**	2.952, K2		2.952, K2		2.659, K3		2.952, K2
**TOTAL DOSE $[e^-/Å^2]$**	118.08		122.19		121.54		122.25
**PROGRAM USED FOR TILT ALIGNMENT**	IMOD		AreTomo		AreTomo		IMOD
**ACQUIRED TILT SERIES**	12	14	23	18	25	18	65
**ANALYSED TOMOGRAMS**	12	12	19	14	20	13	62
**NUMBER OF ANALYSED PROTRUSIONS CONTAINING MICROTUBULES**	10	14	18	15	17	13	62
**NUMBER OF ANALYZED MICROTUBULES**	119		139		192		447

Appendix Table S3. Acquisition and processing parameters for datasets 5 – 7 (EMPIAR-11451) and dataset 8 (EMPIAR-11452). Datasets 5 – 7 were used for quantification of luminal filaments and had a final DMSO concentration of 0.125% (v/v). Datasets 5 – 8 were used for subtomogram averaging of luminal filaments. Dataset 8 was collected on the dTAT knock-out mutant.

CONTROL AND COFILIN KNOCK-DOWN	**BIOLOGICAL REPLICATE 1**		**BIOLOGICAL REPLICATE 2**		**BIOLOGICAL REPLICATE 3**		**BIOLOGICAL REPLICATE 4**	
**ACQUISITION DATE/ GRID ID**	**08.12.2022 (part 1) & 17.12.2022 (part 2)**		**16.12.2022**		**02.02.2023 (part 1) & 12.12.2022 (part 2)**		**03.02.2023**	
**DATASET**	**dataset 9**		**dataset 10**		**dataset 11**		**dataset 12**	
**DAY 1 OF KNOCK-DOWN**	01.12.2022		09.12.2022		24.11.2022		17.01.2023	
**GENOTYPE**	Control knock-down	Cofilin knock-down	Control knock-down	Cofilin knock-down	Control knock-down	Cofilin knock-down	Control knock-down	Cofilin knock-down
**SAMPLE CONDITIONS**	5 h 2 µM TG, 2 h 2.5 µM CytD		5 h 2 µM TG, 2 h 2.5 µM CytD		5 h 2 µM TG, 2 h 2.5 µM CytD		5 h 2 µM TG, 2 h 2.5 µM CytD	
**INCREMENTS/ NUMBER OF TILTS**	3°/ 41		3°/ 41		3°/ 41		3°/ 41	
**FRAMES PER TILT IMAGE**	14		14		14		14	
**PIXEL SIZE [Å/PIXEL], DETECTOR**	2.659, K3 (08.12.2022) & 2.952, K2 (17.12.2022)		2.952, K2		2.952, K2 (02.02.2023) & 2.659, K3 (12.12.2022)		2.952, K2	
**TOTAL DOSE $[e^-/Å^2]$**	113.74 (08.12.2022) & 111.61 (17.12.2022)		111.61		119.66 (part 1) & 111.61 (part 2)		120.91	
**PROGRAM USED FOR TILT ALIGNMENT**	IMOD & AreTomo		AreTomo		IMOD		IMOD	
**ACQUIRED TILT SERIES**	21	20	19	19	22	15	14	13
**ANALYSED TOMOGRAMS**	19	17	16	18	22	13	14	13
**NUMBER OF ANALYSED PROTRUSIONS**	20	17	18	18	22	14	14	13

Appendix Table S4. Acquisition and processing parameters for datasets 9 – 12 (EMPIAR-11453). These datasets were used for quantification of luminal filaments upon control or cofilin knock-down and treatment with CytD & TG (Fig. 3J – L). 8 tilt series from biological replicate 1 (dataset 11) and 3 tilt series from replicate 3 (dataset 13) were collected in different acquisition settings. All samples had a final DMSO concentration of 0.125% (v/v) DMSO.
