# Supplementary material for: CryoET shows cofilactin filaments inside the microtubule lumen
Source: EMBO Rep. 2023 Sep 13;24(11):e57264. doi: 10.15252/embr.202357264 (PMC10626427; doi:10.15252/embr.202357264)
Supplement: Supplementary file 9 — Source Data for Figure 1 [file EMBR-24-e57264-s012.zip › EMBOR-2023-57264V1_SourceDataForFigure1B-E/C/Fig1C_Readme.rtf]

Image was generated in IMOD from tomogram TS_08 (dataset 1, uploaded to EMD-16685 and EMPIAR-11450) as PNG image. Scale bar is 100 µm. 
