# Supplementary material for: CryoET shows cofilactin filaments inside the microtubule lumen
Source: EMBO Rep. 2023 Sep 13;24(11):e57264. doi: 10.15252/embr.202357264 (PMC10626427; doi:10.15252/embr.202357264)
Supplement: Supplementary file 9 — Source Data for Figure 1 [file EMBR-24-e57264-s012.zip › EMBOR-2023-57264V1_SourceDataForFigure1B-E/D/Fig1D_Readme.rtf]

Fig1D and Fig1D_E_EV1F_G_H_MicrotubuleAnalysis are PRISM and Excel files with numerical data.Files called ‘ref_12PF_plus_11.808Apx.png’ - ‘ref_15PF_plus_11.808Apx.png’ are images generated in IMOD as PNGs from the raw files ‘ref_PF_MRA_12PF_PLUS.em’ - ‘ref_PF_MRA_15PF_PLUS.em’. Raw files have a pixel size of 11.808 Å/pixel. The 5 pixel scale bar in PNG files corresponds to 5.9 nm.
