# Supplementary material for: CryoET shows cofilactin filaments inside the microtubule lumen
Source: EMBO Rep. 2023 Sep 13;24(11):e57264. doi: 10.15252/embr.202357264 (PMC10626427; doi:10.15252/embr.202357264)
Supplement: Supplementary file 9 — Source Data for Figure 1 [file EMBR-24-e57264-s012.zip › EMBOR-2023-57264V1_SourceDataForFigure1B-E/B/Fig1B_Readme.rtf]

PNG Image was generated in IMOD as PNG image from low-magnification images acquired during tilt series acquisition. A high-resolution TIF file of the low-magnification square map is provided as ‘DZ4_2A_TS_08.tif’. 
