# Supplementary material for: CryoET shows cofilactin filaments inside the microtubule lumen
Source: EMBO Rep. 2023 Sep 13;24(11):e57264. doi: 10.15252/embr.202357264 (PMC10626427; doi:10.15252/embr.202357264)
Supplement: Supplementary file 10 — Source Data for Figure 2 [file EMBR-24-e57264-s007.zip › EMBOR-2023-57264V1_SourceDataForFigure2A-E/B/Fig2B_Readme.rtf]

Image was generated in IMOD from tomogram TS_297 (dataset 7, uploaded to EMD-16720 and EMPIAR-11451) as PNG image. 
