# Supplementary material for: CryoET shows cofilactin filaments inside the microtubule lumen
Source: EMBO Rep. 2023 Sep 13;24(11):e57264. doi: 10.15252/embr.202357264 (PMC10626427; doi:10.15252/embr.202357264)
Supplement: Supplementary file 11 — Source Data for Figure 3 [file EMBR-24-e57264-s001.zip › EMBOR-2023-57264V1_SourceDataForFigure3A-B_H-L/H/Fig3H_Readme.rtf]

- Blot2_Annotated_Western_221017.png show the annotated western blot with images of the full blots and indications of cropped areas and molecular weight- other PNG files were generated from the raw imaging blots and used to generate Fig. 3H and the Blot2_Annotated_Western_221017.png file.
