# Supplementary material for: CryoET shows cofilactin filaments inside the microtubule lumen
Source: EMBO Rep. 2023 Sep 13;24(11):e57264. doi: 10.15252/embr.202357264 (PMC10626427; doi:10.15252/embr.202357264)
Supplement: Supplementary file 11 — Source Data for Figure 3 [file EMBR-24-e57264-s001.zip › EMBOR-2023-57264V1_SourceDataForFigure3A-B_H-L/J/Fig3J_Readme.rtf]

Files show the annotated blot including images of the full imaged membrane and indications of where the image was cropped as well as PNGs of the imaged blot (Fig3J_alpha-tubulin_Dataset9.png, Fig3J_cofilin_Dataset9.png) which were used to display tubulin and cofilin levels in Fig. 3J, respectively.
