# Supplementary material for: CryoET shows cofilactin filaments inside the microtubule lumen
Source: EMBO Rep. 2023 Sep 13;24(11):e57264. doi: 10.15252/embr.202357264 (PMC10626427; doi:10.15252/embr.202357264)
Supplement: Supplementary file 11 — Source Data for Figure 3 [file EMBR-24-e57264-s001.zip › EMBOR-2023-57264V1_SourceDataForFigure3A-B_H-L/B/Fig3B_Readme.rtf]

Image was generated in IMOD from tomogram TS_215 (dataset 5, uploaded to EMPIAR-11451) as PNG image. 
