# Supplementary material for: CryoET shows cofilactin filaments inside the microtubule lumen
Source: EMBO Rep. 2023 Sep 13;24(11):e57264. doi: 10.15252/embr.202357264 (PMC10626427; doi:10.15252/embr.202357264)
Supplement: Supplementary file 12 [file EMBR-24-e57264-s008.pdf]

EMBO reports

CryoET shows cofilactin filaments inside the microtubule lumen

Camilla Ventura Santos, Stephen Rogers, and Andrew Carter
DOI: 10.15252/embr.202357264

Corresponding author(s): Andrew Carter (cartera@mrc-lmb.cam.ac.uk) , Andrew Carter (cartera@mrc-lmb.cam.ac.uk)

Review Timeline:

Submission Date:	29th Mar 23
Editorial Decision:	3rd May 23
Revision Received:	31st Jul 23
Editorial Decision:	24th Aug 23
Revision Received:	25th Aug 23
Accepted:	30th Aug 23

Editor: Ioannis Papaioannou

Transaction Report:

(Note: With the exception of the correction of typographical or spelling errors that could be a source of ambiguity, letters and reports are not edited. Depending on transfer agreements, referee reports obtained elsewhere may or may not be included in this compilation. Referee reports are anonymous unless the Referee chooses to sign their reports.)

1st Editorial Decision **3rd May 2023**

Dear Dr. Carter,

Thank you for submitting your research manuscript for consideration by EMBO reports. It has now been seen by three experts in the field, and we have received the full set of their reports, which are included below.

As you will see, the referees find the results intriguing and the work technically robust, and they are positive about its publication in EMBO reports once a few concerns and suggestions they provide for the improvement of the manuscript are addressed.

Given their constructive comments, we would like to invite you to revise your manuscript with the understanding that the referee concerns (as detailed in their reports) must be fully addressed and their suggestions taken on board. Please address all referee concerns in a complete point-by-point response. Acceptance of the manuscript will depend on a positive outcome of a second round of review. It is EMBO reports policy to allow a single round of revision only and acceptance or rejection of the manuscript will therefore depend on the completeness of your responses included in the next, final version of the manuscript. If you have any questions or comments, we can also discuss the revisions in a video chat, if you like.

We realize that it is difficult to revise to a specific deadline. In the interest of protecting the conceptual advance provided by the work, we usually recommend a revision within 3 months (August 2nd). Please discuss with me the revision progress ahead of this time if you require more time to complete the revisions.

Please note that your manuscript is formatted as a Report. For Reports, the manuscript should not exceed 27,000 characters (including spaces but excluding Materials & Methods and References) and 5 main plus 5 Expanded View figures. The Results and Discussion sections must be combined, which helps to shorten the manuscript text by eliminating some redundancy that is inevitable when discussing the same experiments twice. The entire Materials and Methods must be included in the main manuscript file.

*****
IMPORTANT NOTE:
We perform an initial quality control of all revised manuscripts before re-review. Your manuscript will FAIL this control and the handling will be DELAYED if your manuscript contains statistics and error bars based on n=2. Please use scatter plots in these cases. No statistics should be calculated if n=2.

When submitting your revised manuscript, please carefully review the instructions that follow below. Failure to include requested items will delay the evaluation of your revision.
*****

When submitting your revised manuscript, we will require:

1) A .docx formatted version of the manuscript text (including legends for main figures, EV figures and tables). Please make sure that the changes are highlighted to be clearly visible.

2) Individual production quality figure files as .eps, .tif, .jpg (one file per figure).
Please download our Figure Preparation Guidelines (figure preparation pdf) from our Author Guidelines pages https://www.embopress.org/page/journal/14693178/authorguide for more info on how to prepare your figures.

3) A .docx formatted letter INCLUDING the reviewers' reports and your detailed point-by-point responses to their comments. As part of the EMBO Press transparent editorial process, the point-by-point response is part of the Review Process File (RPF), which will be published alongside your paper unless you opt out of this (please see below for further information).

4) A complete author checklist, which you can download from our author guidelines (). Please insert information in the checklist that is also reflected in the manuscript. The completed author checklist will also be part of the RPF (please see below for more information).

5) Please note that all corresponding authors are required to supply an ORCID ID for their name upon submission of a revised manuscript (). Please find instructions on how to link your ORCID ID to your account in our manuscript tracking system in our Author guidelines
()

6) We replaced Supplementary Information with Expanded View (EV) Figures and Tables that are collapsible/expandable online. A maximum of 5 EV Figures can be typeset. EV Figures should be cited as 'Figure EV1, Figure EV2" etc... in the text and their respective legends should be included in the main text after the legends of regular figures.

- For the figures that you do NOT wish to display as Expanded View figures, they should be bundled together with their legends in a single PDF file called *Appendix*, which should start with a short Table of Content. Appendix figures should be referred to in the main text as: "Appendix Figure S1, Appendix Figure S2" etc. See detailed instructions regarding expanded view here:

- Additional Tables/Datasets should be labeled and referred to as Table EV1, Dataset EV1, etc. Legends have to be provided in a separate tab in case of .xls files. Alternatively, the legend can be supplied as a separate text file (README) and zipped together with the Table/Dataset file.

7) Before submitting your revision, primary datasets (and computer code, where appropriate) produced in this study need to be deposited in appropriate public databases (see < https://www.embopress.org/page/journal/14693178/authorguide#dataavailability>).

Please remember to provide a reviewer password if the datasets are not yet public.

The accession numbers and database should be listed in a formal "Data availability " section (placed after Materials & Methods) that follows the model below (see also < https://www.embopress.org/page/journal/14693178/authorguide#dataavailability>):

Data availability

The datasets (and computer code) produced in this study are available in the following databases:

- RNA-seq data: Gene Expression Omnibus GSE46843 (https://www.ncbi.nlm.nih.gov/geo/query/acc.cgi?acc=GSE46843)
- [data type]: [name of the resource] [accession number/identifier/doi] ([URL or identifiers.org/DATABASE:ACCESSION])

*** Note: all links should resolve to a page where the data can be accessed. ***

*** Note: the Data Availability Section is restricted to new primary data that are part of this study. ***

8) We request authors to consider both actual and perceived competing interests. Please review the new policy () and update your competing interests statement if necessary. Please name this section 'Disclosure and competing interests statement' and place it after the Acknowledgements section.

9) Figure legends and data quantification:
The following points must be specified in each figure legend:

- the name of the statistical test used to generate error bars and P values,
- the number (n) of independent experiments (please specify technical or biological replicates) underlying each data point,
- the nature of the bars and error bars (s.d., s.e.m.)
- If the data are obtained from n {less than or equal to} 2, use scatter plots showing the individual data points.

Discussion of statistical methodology can be reported in the Materials and Methods section, but figure legends should contain a basic description of n, P and the test applied.

See also the guidelines for figure legend preparation:
https://www.embopress.org/page/journal/14693178/authorguide#figureformat

- Please also include scale bars in all microscopy images.

10) We now request publication of original source data with the aim of making primary data more accessible and transparent to the reader. Our source data coordinator will contact you to discuss which figure panels we would need source data for and will also provide you with helpful tips on how to upload and organize the files.

11) Our journal encourages inclusion of *data citations in the reference list* to directly cite datasets that were re-used and obtained from public databases. Data citations in the article text are distinct from normal bibliographical citations and should directly link to the database records from which the data can be accessed. In the main text, data citations are formatted as follows: "Data ref: Smith et al, 2001" or "Data ref: NCBI Sequence Read Archive PRJNA342805, 2017". In the Reference list, data citations must be labeled with "[DATASET]". A data reference must provide the database name, accession number/identifiers and a resolvable link to the landing page from which the data can be accessed at the end of the reference. Further instructions are available at .

12) Please also note our reference format:
.

13) We now use CRediT to specify the contributions of each author in the journal submission system. CRediT replaces the author contribution section, which should be removed from the manuscript. Please use the free text box to provide more detailed descriptions. See also guide to authors:
.

14) As part of the EMBO publications' Transparent Editorial Process, EMBO reports publishes online a Review Process File to accompany accepted manuscripts. This File will be published in conjunction with your paper and will include the referee reports, your point-by-point response and all pertinent correspondence relating to the manuscript.

You can opt out of this by letting the editorial office know (emboreports@embo.org). If you do opt out, the Review Process File link will point to the following statement: "No Review Process File is available with this article, as the authors have chosen not to make the review process public in this case."

We would also welcome the submission of cover suggestions, or motifs to be used by our Graphics Illustrator in designing a cover.

I look forward to seeing a revised version of your manuscript when it is ready. Please let me know if you have any questions or comments regarding the revision.

Yours sincerely,

Ioannis Papaioannou, PhD
Editor
EMBO reports

Referee #1:

In their paper "CryoET shows cofilactin filaments inside the microtubule lumen", Santos, Rogers, and Carter perform cellular cryo-electron tomogoraphy studies (cryo-ET) that demonstrate the presence of cofilin-satured actin filaments ("cofilactin") within the lumen of microtubules in Drosophila S2 cells treated with the F-actin inhibitor cytochalaisin D. This work significantly expands on a previous study which found distorted actin filaments (F-actin) in the microtubules of human cells (Paul et al., JCB, 2020) treated with a kinesin inhibitor, demonstrating that this phenomenon isn't restricted to a single organism or chemical treatment, but may exist broadly in nature. Furthermore, the authors confidently identify cofilin saturation binding as the cause of the distortion using both subtomogram averaging and docking analysis, as well as RNAi knock-down of cofilin, which results in bare F-actin inside microtubules. They also utilize thapsigargin to increase the prevalence of luminal cofilactin, which they confirm reduces cofilin phosphorylation, consistent with cofilin phosphorylation inhibiting its F-actin engagement inside the microtubule, as expected from its well-established inhibitory role.

Overall, we found this to be clear, concise, and thorough study. It generalizes the existence of intra-luminal actin filaments from one observation in chemically manipulated human cells to a different species, and furthermore characterizes it much more extensively and convincingly. We think this paper will no doubt spur the wider community to look for microtubules featuring luminal F-actin / cofilactin in other species and cell types using cryo-ET and other methods, potentially in more physiological settings where they are likely to be rarer and thus overlooked. Moreover, we appreciate that the authors did not make mechanistic or functional claims beyond the scope of their observations. Consistent with the scope of an EMBO Reports paper, they describe a single striking finding, which is well-supported using a combination of cryo-ET and cell biological manipulations that will serve as a very good example of the rigorous combination of these methods for the field.

We have a few modest suggestions for improving the paper, after which we enthusiastically recommend acceptance by EMBO Reports.

Minor point / suggestion:

The authors report that the registers of tubulin and cofilactin subunits are not correlated, as each becomes smeared out when the other drives alignment during subtomogram averaging. Although this doesn't support a robust cross-talk between the two filaments, they also note hints of possible connecting density, suggesting there may be some binding interactions. We suggest

the authors analyze whether there is a correlation between the polarity of a microtubule and its internal F-actin / cofilactin filament (information they likely already have from their subtomogram averaging analysis). Either a correlation or a lack thereof would be interesting when considering how F-actin internalization might occur.

Small details:
1. Figure 3 A,B show a tomogram slice and crossover lengths obtained from cytoplasmic F-actin. Was this actin filament from these datasets?
2. Figure EV2 D. The legend misstates that the fourth filament is "not clear in this this tomographic slice", rather than the third.
3. It would be nice to include some supplementary movies of the tomograms if allowed by the journal, so that non-specialist authors could appreciate the nature of the data.
4. Kudos to the authors for depositing representative tomograms.

Referee #2:

It was found that cytoplasmic microtubules can contain small proteins or filaments within their structures. Nevertheless, the identity of these proteins and the factors contributing to their accumulation remain uncertain. Santos et al.'s manuscript offers highly intriguing cryo-ET structures of cofilactin in the microtubule lumen, elucidating this previously elusive area of research. The authors convincingly demonstrate that inhibiting the SERCA with thapsigargin increases the frequency of these filaments. Their findings significantly advance our understanding of microtubule-associated proteins within the microtubule lumen. The work is technically robust, supported by a variety of complementary experimental approaches and thorough quantitative analyses. Furthermore, the research topic is highly relevant to the scope of this journal. I have no concerns regarding the main conclusions or the potential impacts of this study in the cytoskeletal field, and thus, I highly recommend its publication after addressing a few minor technical issues.
1. The authors discovered at least 38 protrusions containing microtubules with mixed orientations. This is particularly interesting because a cellular-level mechanism must be involved to regulate the distribution of MTOC in the induced protrusions. I suggest that the authors elaborate on the differences among the various protrusions, explain why some protrusions may contain mixed microtubules while others do not, and propose potential factors that might control the orientation distributions.

2. In relation to the above point, I have several additional questions about the cofilactin structures they determined: What about the orientation distribution of the luminal cofilactin from the reconstructed tomograms? Are the orientations of luminal cofilactin strictly correlated with the MT orientation? What are their orientation distributions in the 3D tomograms? Did the authors observe any preferred locations (outer regions, central regions, or no preference)? What about the orientation distribution changes after SERCA inhibition? Are there any interesting patterns that could potentially be linked to local protrusion geometry and structures? Could these differences subsequently control or affect protrusion growth, cellular cargo transport, and local structural differentiation? The authors do not need to address all these questions in the revised manuscript, but I am curious if there is a possibility to perform slightly more in-depth analyses at the level of whole tomograms to identify any interesting patterns.

3. Given that microtubule protofilaments are nearly straight and parallel to the longitudinal axis, there is a high cross-correlation between a correctly aligned microtubule and misaligned ones in the opposite direction. At the reported resolution, it is possible that certain sub-volumes of the microtubule may not be correctly oriented. The authors clearly demonstrated that protofilaments of microtubules viewed from the plus end appear to rotate in an anti-clockwise direction and conversely in a clockwise direction when viewed from the minus end. However, this is the final result of sub-volume averaging, not the features directly observed from individual sub-volumes of the originally reconstructed tomograms. Because it is an average, even a few misaligned microtubule sub-volumes would not affect the final appearance of this feature at such resolution. Therefore, I am asking whether the authors could provide a validation test on the orientation assignment of a representative microtubule (of average quality) and demonstrate how robust the current alignment is. For example, the authors might consider rotating a set of aligned microtubule segments by 180 degrees, re-refining them, and estimating the cross-correlation coefficients at the opposite orientations. If the differences are not statistically significant, I may have some concern about the final distribution in Figure 1E.

4. A minor formatting issue: In the Materials and Methods section, I noticed that all the units of DNA/RNA concentrations, as well as many other types of units, contain a space after the slash, e.g., µg/ ml, Å/ pixel. It seems more common to use "µg/mL" (without a space before "mL" and many other units), Å/pixel, v/v, etc.

Referee #3:

Please find below the review of the article EMBOR-2023-57264V1 entitled "CryoET shows cofilactin filaments inside the

microtubule lumen" by Santos and co-authors.

In this manuscript, the authors report that in Drosophilia S2 cells, they found in some cases actin filaments (decorated with ADF/cofilin) inside the microtubule lumen. The observation of actin filaments inside the lumen of microtubules has been reported previously Paul et al, JCB, 2020. The originality here is that the actin filaments inside the lumen appear to be decorated by ADF/cofilin.

Decreasing the cellular concentration of cofilin using SiRNA reduces the number of actin filaments inside the microtubule lumen. Overall, the data are very interesting, but the role of this interaction is unclear.
As such, the manuscript describes a curiosity.

Perhaps the authors could clarify whether cofilin-decorated actin filaments are observed outside the microtubule lumen? Do the microtubules protect these filaments from depolymerization?

An interesting possibility raised by the authors is that this is a mechanism of cofilin sequestration, but this mechanism is not challenged by the experiments.

An interesting experiment, if possible, is to polymerize actin in the presence or absence of excess of cofilin in vitro, then add tubulin and assemble microtubules. Will it be possible to observe actin filaments within the lumen of microtubules only if they are decorated by cofilin? This would demonstrate that actin filaments could somehow serve as a template for microtubule assembly.

Point-by-point response

Referee #1:

In their paper "CryoET shows cofilactin filaments inside the microtubule lumen", Santos, Rogers, and Carter perform cellular cryo-electron tomography studies (cryo-ET) that demonstrate the presence of cofilin-saturated actin filaments ("cofilactin") within the lumen of microtubules in Drosophila S2 cells treated with the F-actin inhibitor cytochalasin D. This work significantly expands on a previous study which found distorted actin filaments (F-actin) in the microtubules of human cells (Paul et al., JCB, 2020) treated with a kinesin inhibitor, demonstrating that this phenomenon isn't restricted to a single organism or chemical treatment, but may exist broadly in nature. Furthermore, the authors confidently identify cofilin saturation binding as the cause of the distortion using both subtomogram averaging and docking analysis, as well as RNAi knock-down of cofilin, which results in bare F-actin inside microtubules. They also utilize thapsigargin to increase the prevalence of luminal cofilactin, which they confirm reduces cofilin phosphorylation, consistent with cofilin phosphorylation inhibiting its F-actin engagement inside the microtubule, as expected from its well-established inhibitory role.

Overall, we found this to be clear, concise, and thorough study. It generalizes the existence of intra-luminal actin filaments from one observation in chemically manipulated human cells to a different species, and furthermore characterizes it much more extensively and convincingly. We think this paper will no doubt spur the wider community to look for microtubules featuring luminal F-actin / cofilactin in other species and cell types using cryo-ET and other methods, potentially in more physiological settings where they are likely to be rarer and thus overlooked. Moreover, we appreciate that the authors did not make mechanistic or functional claims beyond the scope of their observations. Consistent with the scope of an EMBO Reports paper, they describe a single striking finding, which is well-supported using a combination of cryo-ET and cell biological manipulations that will serve as a very good example of the rigorous combination of these methods for the field.

We have a few modest suggestions for improving the paper, after which we enthusiastically recommend acceptance by EMBO Reports.

Minor point / suggestion:

The authors report that the registers of tubulin and cofilactin subunits are not correlated, as each becomes smeared out when the other drives alignment during subtomogram averaging. Although this doesn't support a robust cross-talk between the two filaments, they also note hints of possible connecting density, suggesting there may be some binding interactions. We suggest the authors analyze whether there is a correlation between the polarity of a microtubule and its internal F-actin / cofilactin filament (information they likely already have from their subtomogram averaging analysis). Either a correlation or a lack thereof would be interesting when considering how F-actin internalization might occur.

We have analyzed the orientations of microtubules and luminal filaments relative to each other and incorporated the results in the text (lines 122 – 123) and in Fig. EV3E, F. Briefly, our analysis revealed no correlation between the orientation of the luminal filaments and the surrounding microtubules as 46.8 ± 5.5% of luminal filaments had their plus (barbed) ends pointing in the same direction as the surrounding microtubule's plus end (Fig. EV3F).

Small details:
1. Figure 3 A,B show a tomogram slice and crossover lengths obtained from cytoplasmic F-actin. Was this actin filament from these datasets?

The f-actin filament is also from one of the datasets (dataset 5). We have clarified this in the figure legend (Fig. 3A, B).

2. Figure EV2 D. The legend misstates that the fourth filament is "not clear in this this tomographic slice", rather than the third.

We have corrected the figure legend.

3. It would be nice to include some supplementary movies of the tomograms if allowed by the journal, so that non-specialist authors could appreciate the nature of the data.

We have compiled movies of representative tomograms and uploaded them as expanded view files.

4. Kudos to the authors for depositing representative tomograms.

Referee #2:

It was found that cytoplasmic microtubules can contain small proteins or filaments within their structures. Nevertheless, the identity of these proteins and the factors contributing to their accumulation remain uncertain. Santos et al.'s manuscript offers highly intriguing cryo-ET structures of cofilactin in the microtubule lumen, elucidating this previously elusive area of research. The authors convincingly demonstrate that inhibiting the SERCA with thapsigargin increases the frequency of these filaments. Their findings significantly advance our understanding of microtubule-associated proteins within the microtubule lumen. The work is technically robust, supported by a variety of complementary experimental approaches and thorough quantitative analyses. Furthermore, the research topic is highly relevant to the scope of this journal. I have no concerns regarding the main conclusions or the potential impacts of this study in the cytoskeletal field, and thus, I highly recommend its publication after addressing a few minor technical issues.

1. The authors discovered at least 38 protrusions containing microtubules with mixed orientations. This is particularly interesting because a cellular-level mechanism must be involved to regulate the distribution of MTOC in the induced protrusions. I suggest that the authors elaborate on the differences among the various protrusions, explain why some protrusions may contain mixed microtubules while others do not, and propose potential factors that might control the orientation distributions.

To test if there were differences between protrusions with uniform or mixed microtubule arrays, we performed three analyses. First, we looked at whether protrusions that contained many or few microtubules tended to have uniform or mixed microtubule arrays. As shown in Referee Figure 1, we observed protrusions with up to 16 or 17 microtubules with uniform or mixed microtubule orientation, respectively, indicating no correlation between the number of microtubules per protrusion and the overall orientations of microtubules therein.

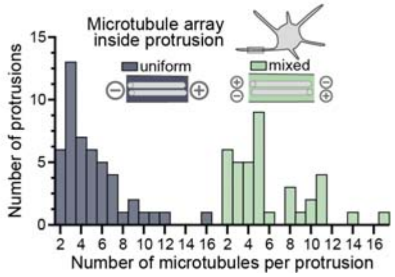

***Referee Figure 1*. Microtubule number inside protrusions with uniform or mixed microtubule arrays.** Histogram of the number of microtubules per protrusion in protrusions with uniform (blue) or mixed (green) microtubule arrays. The cartoon at the top exemplifies uniform/mixed microtubule arrays.

Second, we analyzed whether protrusions with uniform or mixed orientation contained γ-tubulin ring complexes (γ-TuRCs), which could be expected to nucleate microtubules in a particular orientation. Across our data, we observed 89 microtubule ends. Of these, 46 were minus ends and 4 appeared to be capped by structures resembling full or partial γ-TuRCs (Referee Figure 2). These events were too rare to quantify and were present in protrusions with both uniform (n = 2) or mixed (n = 1) microtubules. The presence of γ-TuRCs, therefore, does not seem to determine the overall distribution of microtubule orientations in a protrusion.

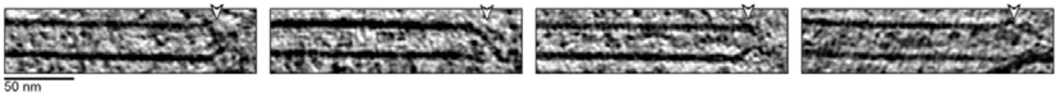

Referee Figure 2. Putative γ-TuRCs (arrowheads) observed inside protrusions.

Finally, we looked at whether the distance of the protrusion position from the cell body determined the orientations of microtubules. We estimated the distance of the tomogram acquisition position from the cell body membrane in 47 cases, for which we could confidently trace back the protrusion to the corresponding cell body. Distances were in the range of ~2 – 32 µm. This analysis showed that protrusions with uniform and mixed microtubule arrays were found at similar distances from the cell body.

Overall, our analyses revealed no obvious correlation that would give insights into the mechanism that governs microtubule orientation within protrusions. This is consistent with our prior work showing that known microtubule nucleating factors are not required for steady-state assembly of the acentrosomal microtubule array in S2 cells (Rogers et al. MBOC 19:3163 (2008)). However, research from the Gelfand lab suggested that microtubule-based motors are responsible for generating uniformly oriented microtubule arrays inside protrusions. Based on this, we speculate that microtubules with opposite orientation to the majority within protrusion were captured prior to motor-mediated sorting. As the establishment of microtubule polarity in the cell processes was not the focus of this work, we have included our speculation regarding this mechanism in the manuscript (lines 78 – 80).

2. In relation to the above point, I have several additional questions about the cofilactin structures they determined:

What about the orientation distribution of the luminal cofilactin from the reconstructed tomograms? Are the orientations of luminal cofilactin strictly correlated with the MT orientation? What are their orientation distributions in the 3D tomograms? Did the authors observe any preferred locations (outer regions, central regions, or no preference)? What about the orientation distribution changes after SERCA inhibition?

As explained in response to Referee #1, we analyzed the orientations of luminal filaments relative to the microtubules within the protrusions and found that 46.8 ± 5.5% of luminal filaments had their plus (barbed) ends pointing in the same direction as the plus end of the surrounding microtubule. We have included two figure panels explaining the quantification and results (Fig. EV3E, F) and incorporated these findings into the text (lines 122 – 123).

Regarding the orientation of luminal filaments within the 3D tomogram, most microtubules, and consequently, luminal filaments, ran along the length of the protrusion.

To analyze changes in luminal filament orientations relative to the surrounding microtubule upon SERCA inhibition, we looked at the number of luminal filaments that were oriented with their plus (barbed) ends towards the microtubule plus and minus end in CytD & DMSO and thapsigargin treated (CytD & TG) cells (Referee Figure 3). Under both treatments, we observed many luminal filaments that were oriented with their barbed (plus) ends towards the microtubule plus and minus ends, indicating no change upon SERCA inhibition.

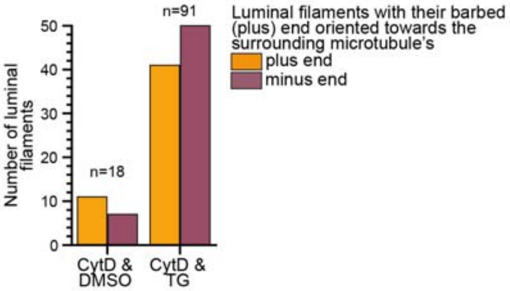

Referee Figure 3. Luminal filament orientation relative to the surrounding microtubule in control and TG-treated cells. Number of luminal filaments in control (CytD & DMSO) or thapsigargin (CytD & TG) treated cells with their barbed (plus) ends pointing towards the plus (n=11, CytD & DMSO, n=41, CytD & TG) or minus (n=7, CytD & DMSO, n=50, CytD & TG) end of the surrounding microtubule.

To look at whether there were preferred locations for the occurrence of luminal filaments (proximal vs. distal regions) we estimated the distance of tomogram positions from the cell body for multiple tomograms with and without luminal filaments. We found no obvious pattern but had too low numbers to approach this in a statistically meaningful way.

Are there any interesting patterns that could potentially be linked to local protrusion geometry and structures? Could these differences subsequently control or affect protrusion growth, cellular cargo transport, and local structural differentiation?

When looking through our tomograms of control and TG-treated cells, we observed no obvious differences in protrusion geometry, which we defined as protrusion width and shape. To gain further insight, we analyzed how many microtubules were contained within each protrusion in control and TG treated cells. This analysis revealed that the targeted protrusions contained very similar numbers of microtubules under both conditions (4.8±4.6 microtubules per protrusion for CytD & DMSO and 4.9±4.1 for CytD & TG, Referee Figure 4). Therefore, we observed no obvious change in protrusion geometry upon SERCA inhibition.

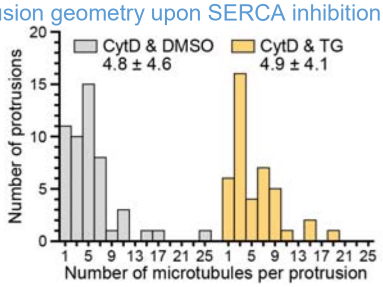

Referee Figure 4. Comparison of microtubule number per protrusion in control and TG-treated cells. Histogram of CytD & DMSO and CytD & TG are shown. Mean ± s.d.s are indicated at the top.

3. Given that microtubule protofilaments are nearly straight and parallel to the longitudinal axis, there is a high

cross-correlation between a correctly aligned microtubule and misaligned ones in the opposite direction. At the reported resolution, it is possible that certain sub-volumes of the microtubule may not be correctly oriented. The authors clearly demonstrated that protofilaments of microtubules viewed from the plus end appear to rotate in an anti-clockwise direction and conversely in a clockwise direction when viewed from the minus end. However, this is the final result of sub-volume averaging, not the features directly observed from individual sub-volumes of the originally reconstructed tomograms. Because it is an average, even a few misaligned microtubule sub-volumes would not affect the final appearance of this feature at such resolution. Therefore, I am asking whether the authors could provide a validation test on the orientation assignment of a representative microtubule (of average quality) and demonstrate how robust the current alignment is. For example, the authors might consider rotating a set of aligned microtubule segments by 180 degrees, re-refining them, and estimating the cross-correlation coefficients at the opposite orientations. If the differences are not statistically significant, I may have some concern about the final distribution in Figure 1E.

The method we used to determine microtubule protofilament numbers and orientations was very similar to that described in our previous publication (Foster et al. 2022). We are now specifically referring to this publication in the manuscript and making clear that the procedures are related (lines 62 – 63 and line 70). In addition, we have expanded Fig. EV1E, F to show the classification result of particles within a representative microtubule. Further representative results and corresponding per-microtubule subtomogram averages of 12 – 15-protofilament microtubules in plus- and minus-end-facing orientations have been included in the Appendix (Appendix Figure S1).

As requested, we have also validated our analysis in two ways. First, we generated per-microtubule averages after the multireference alignment. Visual inspection of these averages gave the same results for the polarity and protofilament number as the assignment by classification. The only exception were microtubules, which were not assigned by either method and whose results could therefore not be compared across methods.
Second, we analyzed the cross-correlation scores. In the multireference alignment used for the polarity determination, each particle is aligned to 8 references (12, 13, 14, 15 protofilament microtubules each with plus- and minus-end-facing orientation). Thus, each particle has a cross-correlation score for comparison to each of the 8 references. We have plotted all cross-correlation scores for particles from 8 representative microtubules (Referee Figure 5). Each particle is assigned to the class (i.e. reference), for which it has the highest cross-correlation score and this value is highlighted in pink for each particle. We performed one-way ANOVA tests with matched values for each particle and multiple comparisons to the assigned class. All ANOVAs and multiple comparisons had p-values <0.0001 and only the significance/p-values between comparisons of classes with opposing orientations (with the same protofilament number) are indicated with bars and p-values in Referee Figure 5.

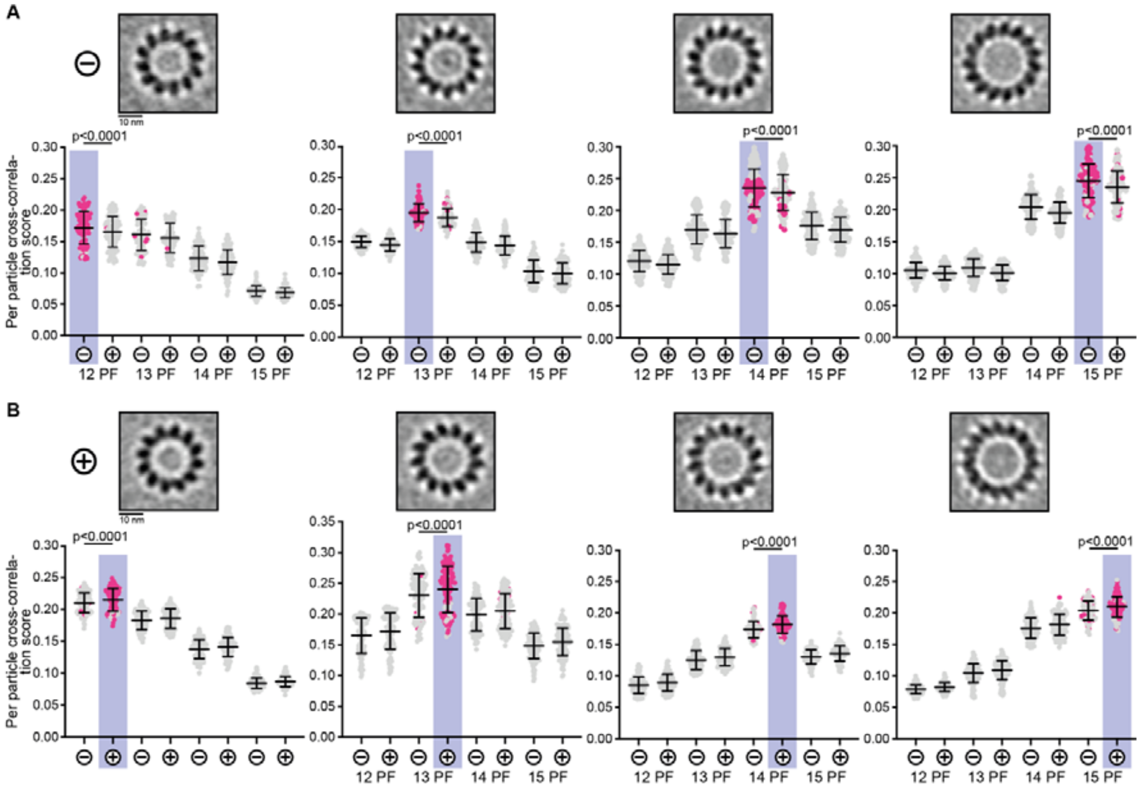

Referee Figure 5: Validation of the assignment of microtubule orientation and protofilament number by per-particle classification. A, B) Cross-correlation scores for each particle for the alignment to each of the 8 references in the multireference alignment are shown for representative 12, 13, 14, 15 protofilament minus-end- (A) and plus-end-facing (B) microtubules. For each particle, the highest cross-correlation score is shown in pink and this determines the class into which that particle fell. Each microtubule was assigned based on the percentage of particles that fell into each class. The assigned class for each microtubule is highlighted in purple. Per-microtubule subtomogram averages are shown at the top for each of the shown microtubules.

4. A minor formatting issue: In the Materials and Methods section, I noticed that all the units of DNA/RNA concentrations, as well as many other types of units, contain a space after the slash, e.g., µg/ ml, Å/ pixel. It seems more common to use “µg/mL” (without a space before “mL” and many other units), Å/pixel, v/v, etc.

We thank the referee for the comment and adjusted this accordingly.

Referee #3:

Please find below the review of the article EMBOR-2023-57264V1 entitled “CryoET shows cofilactin filaments inside the microtubule lumen” by Santos and co-authors.

In this manuscript, the authors report that in Drosophilia S2 cells, they found in some cases actin filaments (decorated with ADF/cofilin) inside the microtubule lumen. The observation of actin filaments inside the lumen of microtubules has been reported previously Paul et al, JCB, 2020. The originality here is that the actin filaments inside the lumen appear to be decorated by ADF/cofilin.

Decreasing the cellular concentration of cofilin using SiRNA reduces the number of actin filaments inside the microtubule lumen.
Overall, the data are very interesting, but the role of this interaction is unclear.
As such, the manuscript describes a curiosity.

Perhaps the authors could clarify whether cofilin-decorated actin filaments are observed outside the microtubule lumen? Do the microtubules protect these filaments from depolymerization?

We did observe putative cofilactin filaments in the cytoplasm of some protrusions and have added this observation to the manuscript text (lines 153 – 157) with images of these filaments in Fig. EV3I, J.

We have added the speculation that filaments inside the microtubule lumen may be protected from disassembly to the discussion (lines 193 – 194).

An interesting possibility raised by the authors is that this is a mechanism of cofilin sequestration, but this mechanism is not challenged by the experiments.

We have removed the discussion of cofilin sequestration.

An interesting experiment, if possible, is to polymerize actin in the presence or absence of excess of cofilin in vitro, then add tubulin and assemble microtubules. Will it be possible to observe actin filaments within the lumen of microtubules only if they are decorated by cofilin? This would demonstrate that actin filaments could somehow serve as a template for microtubule assembly.

We acknowledge that this experiment would give insights into the prerequisites of luminal filament formation. However, it is not within the scope of the current study. We are aware that another group is actively pursuing these experiments.

Dear Dr. Carter,

Thank you for submitting your revised manuscript to EMBO reports. We have now received the full set of reports from the three referees that were asked to re-evaluate your study. Their comments are included below.

As you will see, all three referees are satisfied with the revision, they mention that the paper has been strengthened and their previous concerns satisfactorily addressed, and they all support publication of your manuscript in EMBO reports.

From the editorial side, there are also a few things that we need from you to address in a revised version of your manuscript before we can proceed with its acceptance:

- Please provide up to 5 keywords in your revised manuscript (after the Abstract).

- Appendix Table S1 is either missing or the Appendix Table labels need to be corrected. In the latter case, please update all relevant callouts accordingly.

- In checking your figures, we detected possible re-use of images between Fig. 1D cells and Fig. EV1E reference cells. This is not directly mentioned in the figure legends. If images are re-used between figures, this must be clearly mentioned in the respective figure legends.

- Please note that EMBO press papers are accompanied online by:
A) a short (1-2 sentences) summary of the findings and their significance,
B) 2-4 short bullet points highlighting the key results, and
C) a synopsis image that is exactly 550 pixels wide and 300-600 pixels high (the height is variable). You can either show a model or key data in the synopsis image. Please note that the text needs to be readable at the final size.
Please upload this information along with your revised manuscript (the text for A and B should be provided in a separate Word file).

We would also welcome the submission of cover suggestions or motifs to be used by our Graphics Illustrator in designing a cover.

Please also note that as part of the EMBO publications' Transparent Editorial Process, EMBO reports publishes online a Review Process File to accompany accepted manuscripts. This File will be published in conjunction with your paper and will include the referee reports, your point-by-point response and all pertinent correspondence relating to the manuscript.

You can opt out of this by letting the editorial office know (emboreports@embo.org). If you do opt out, the Review Process File link will point to the following statement: "No Review Process File is available with this article, as the authors have chosen not to make the review process public in this case."

We look forward to seeing a final version of your manuscript as soon as possible.

Yours sincerely,

Ioannis Papaioannou, PhD
Editor
EMBO reports

Referee #1:

The authors have thoroughly responded to the reviewer comments, strengthening the already excellent paper substantially. It should be accepted for publication without further delay.

Referee #2:

The authors have carefully addressed my concerns regarding cryo-ET data analysis, and also provided excellent explanations to my additional questions originally just out of curiosity. Thus, I strongly recommend this paper be published.

Referee #3:

The authors have satisfactorily addressed most of my concerns.

The authors have addressed all minor editorial requests.

Dr. Andrew Carter
MRC Laboratory of Molecular Biology
Structural Studies
Francis Crick Avenue
Cambridge Biomedical Campus
Cambridge CB2 0QH
United Kingdom

Dear Dr. Carter,

I am very pleased to accept your manuscript for publication in the next available issue of EMBO reports. Thank you for your contribution to our journal.

At the end of this email I include important information about how to proceed. Please ensure that you take the time to read the information and complete and return the necessary forms to allow us to publish your manuscript as quickly as possible.

As part of the EMBO publication's Transparent Editorial Process, EMBO reports publishes online a Review Process File to accompany accepted manuscripts. As you are aware, this File will be published in conjunction with your paper and will include the referee reports, your point-by-point response and all pertinent correspondence relating to the manuscript.

If you do NOT want this File to be published, please inform the editorial office within 2 days, if you have not done so already, otherwise the File will be published by default [contact: emboreports@embo.org]. If you do opt out, the Review Process File link will point to the following statement: "No Review Process File is available with this article, as the authors have chosen not to make the review process public in this case."

Should you be planning a Press Release on your article, please get in contact with emboreports@wiley.com as early as possible, in order to coordinate publication and release dates.

Thank you again for your contribution to EMBO reports and congratulations on a successful publication. Please consider us again in the future for your most exciting work.

Yours sincerely,

Ioannis Papaioannou, PhD
Editor
EMBO reports

****

THINGS TO DO NOW:

Please note that you will be contacted by Wiley Author Services to complete licensing and payment information. The required 'Page Charges Authorization Form' is available here: https://www.embopress.org/pb-assets/embo-site/er_apc.pdf - please download and complete the form and return to embopressproduction@wiley.com

EMBO Press participates in many Publish and Read agreements that allow authors to publish Open Access with reduced/no publication charges. Check your eligibility: https://authorservices.wiley.com/author-resources/Journal-Authors/open-access/affiliation-policies-payments/index.html

You will receive proofs by e-mail approximately 2-3 weeks after all relevant files have been sent to our Production Office; you should return your corrections within 2 days of receiving the proofs.

Please inform us if there is likely to be any difficulty in reaching you at the above address at that time. Failure to meet our deadlines may result in a delay of publication, or publication without your corrections.

All further communications concerning your paper should quote reference number EMBOR-2023-57264V3 and be addressed to emboreports@wiley.com.

Should you be planning a Press Release on your article, please get in contact with emboreports@wiley.com as early as

possible, in order to coordinate publication and release dates.

EMBO Press Author Checklist

Corresponding Author Name: Andrew Carter	
Journal Submitted to: EMBO Reports	
Manuscript Number: 2023-57264	

USEFUL LINKS FOR COMPLETING THIS FORM
The EMBO Journal - Author Guidelines
EMBO Reports - Author Guidelines
Molecular Systems Biology - Author Guidelines
EMBO Molecular Medicine - Author Guidelines

Reporting Checklist for Life Science Articles (updated January
This checklist is adapted from Materials Design Analysis Reporting (MDAR) Checklist for Authors. MDAR establishes a minimum set of requirements in transparent reporting in the life sciences (see Statement of Task: 10.31222/osf.io/9sm4x). Please follow the journal's guidelines in preparing your
Please note that a copy of this checklist will be published alongside your article.

Abridged guidelines for figures
1. Data
The data shown in figures should satisfy the following conditions:
- the data were obtained and processed according to the field's best practice and are presented to reflect the results of the experiments in an accurate and unbiased manner.
- ideally, figure panels should include only measurements that are directly comparable to each other and obtained with the same assay.
- plots include clearly labeled error bars for independent experiments and sample sizes. Unless justified, error bars should not be shown for technical
- if n<5, the individual data points from each experiment should be plotted. Any statistical test employed should be justified.
- Source Data should be included to report the data underlying figures according to the guidelines set out in the authorship guidelines on Data

2. Captions
Each figure caption should contain the following information, for each panel where they are relevant:
- a specification of the experimental system investigated (eg cell line, species name).
- the assay(s) and method(s) used to carry out the reported observations and measurements.
- an explicit mention of the biological and chemical entity(ies) that are being measured.
- an explicit mention of the biological and chemical entity(ies) that are altered/varied/perturbed in a controlled manner.
- the exact sample size (n) for each experimental group/condition, given as a number, not a range;
- a description of the sample collection allowing the reader to understand whether the samples represent technical or biological replicates (including how many animals, litters, cultures, etc.).
- a statement of how many times the experiment shown was independently replicated in the laboratory.
- definitions of statistical methods and measures:
  - common tests, such as t-test (please specify whether paired vs. unpaired), simple χ2 tests, Wilcoxon and Mann-Whitney tests, can be unambiguously identified by name only, but more complex techniques should be described in the methods section;
  - are tests one-sided or two-sided?
  - are there adjustments for multiple comparisons?
  - exact statistical test results, e.g., P values = x but not P values < x;
  - definition of 'center values' as median or average;
  - definition of error bars as s.d. or s.e.m.

Please complete ALL of the questions below.
Select "Not Applicable" only when the requested information is not relevant for your study.

Materials

Newly Created Materials	Information included in the manuscript?	In which section is the information available? (Reagents and Tools Table, Materials and Methods, Figures, Data Availability Section)
New materials and reagents need to be available; do any restrictions apply?	Yes	The dTAT knock-out S2 cell line is available from the Drosophila Genomics Resource Center (code DGRC#344). This is also specified in the Materials and methods section.

Antibodies	Information included in the manuscript?	In which section is the information available? (Reagents and Tools Table, Materials and Methods, Figures, Data Availability Section)
For **antibodies** provide the following information:<br>- Commercial antibodies: RRID (if possible) or supplier name, catalogue number and or/clone number<br>- Non-commercial: RRID or citation	Yes	Clone number, catalogue number or citations (for non-commercial antibodies) are specified in the Materials and methods section.

DNA and RNA sequences	Information included in the manuscript?	In which section is the information available? (Reagents and Tools Table, Materials and Methods, Figures, Data Availability Section)
**Short novel DNA or RNA including primers, probes**: provide the sequences.	Yes	Sequences are given in the Materials and methods section.

Cell materials	Information included in the manuscript?	In which section is the information available? (Reagents and Tools Table, Materials and Methods, Figures, Data Availability Section)
**Cell lines:** Provide species information, strain. Provide accession number in repository **OR** supplier name, catalog number, clone number, and/**OR** RRID.	Yes	Used cell lines are specified in the Materials and methods section.
**Primary cultures:** Provide species, strain, sex of origin, genetic modification status.	Not Applicable	
Report if the cell lines were recently **authenticated** (e.g., by STR profiling) and tested for mycoplasma contamination.	Yes	Specified in Materials and methods (mycoplasma testing).

Experimental animals	Information included in the manuscript?	In which section is the information available? (Reagents and Tools Table, Materials and Methods, Figures, Data Availability Section)
**Laboratory animals or Model organisms:** Provide species, strain, sex, age, genetic modification status. Provide accession number in repository **OR** supplier name, catalog number, clone number, **OR** RRID.	Not Applicable	
**Animal observed in or captured from the field:** Provide species, sex, and age where possible.	Not Applicable	
Please detail **housing and husbandry conditions**.	Not Applicable	

Plants and microbes	Information included in the manuscript?	In which section is the information available? (Reagents and Tools Table, Materials and Methods, Figures, Data Availability Section)
**Plants:** provide species and strain, ecotype and cultivar where relevant, unique accession number if available, and source (including location for collected wild specimens).	Not Applicable	
**Microbes:** provide species and strain, unique accession number if available, and source.	Not Applicable	

Human research participants	Information included in the manuscript?	In which section is the information available? (Reagents and Tools Table, Materials and Methods, Figures, Data Availability Section)
If collected and within the bounds of privacy constraints report on age, sex and gender or ethnicity for all study participants.	Not Applicable	

Core facilities	Information included in the manuscript?	In which section is the information available? (Reagents and Tools Table, Materials and Methods, Figures, Data Availability Section)
If your work benefited from core facilities, was their service mentioned in the acknowledgments section?	Yes	Core facilities are acknowledged in the Acknowledgements section.

Design

Study protocol	Information included in the manuscript?	In which section is the information available? (Reagents and Tools Table, Materials and Methods, Figures, Data Availability Section)

If study protocol has been **pre-registered, provide DOI in the manuscript**. For clinical trials, provide the trial registration number **OR** cite DOI.	Not Applicable	
Report the **clinical trial registration number** (at ClinicalTrials.gov or equivalent), where applicable.	Not Applicable	

Laboratory protocol	Information included in the manuscript?	In which section is the information available? (Reagents and Tools Table, Materials and Methods, Figures, Data Availability Section)
Provide DOI OR other citation details if **external detailed step-by-step protocols** are available.	Yes	Specified in Materials and Methods.

Experimental study design and statistics	Information included in the manuscript?	In which section is the information available? (Reagents and Tools Table, Materials and Methods, Figures, Data Availability Section)
Include a statement about **sample size** estimate even if no statistical methods were used.	Not Applicable	
Were any steps taken to minimize the effects of subjective bias when allocating animals/samples to treatment (e.g. **randomization procedure**)? If yes, have they been described?	Not Applicable	
Include a statement about **blinding** even if no blinding was done.	Yes	Specified in Materials and methods.
Describe **inclusion/exclusion criteria** if samples or animals were excluded from the analysis. Were the criteria pre-established?<br><br>If sample or data points were omitted from analysis, report if this was due to attrition or intentional exclusion and provide justification.	Yes	Inclusion/exclusion criteria are described in the manuscript text, Materials and Methods, Figure legends.
For every figure, are **statistical tests** justified as appropriate? Do the data meet the assumptions of the tests (e.g., normal distribution)? Describe any methods used to assess it. Is there an estimate of variation within each group of data? Is the variance similar between the groups that are being statistically compared?	Yes	Materials and Methods, Figure legends.

Sample definition and in-laboratory replication	Information included in the manuscript?	In which section is the information available? (Reagents and Tools Table, Materials and Methods, Figures, Data Availability Section)
In the figure legends: state number of times the experiment was **replicated** in laboratory.	Yes	This information can be found in the Figure legends.
In the figure legends: define whether data describe **technical or biological replicates**.	Yes	This information can be found in the Figure legends.

Ethics

Ethics	Information included in the manuscript?	In which section is the information available? (Reagents and Tools Table, Materials and Methods, Figures, Data Availability Section)
Studies involving **human participants**: State details of **authority granting ethics approval** (IRB or equivalent committee(s), provide reference number for approval.	Not Applicable	
Studies involving **human participants**: Include a statement confirming that **informed consent** was obtained from all subjects and that the experiments conformed to the principles set out in the WMA Declaration of Helsinki and the Department of Health and Human Services Belmont Report.	Not Applicable	
Studies involving **human participants:** For publication of **patient photos**, include a statement confirming that consent to publish was obtained.	Not Applicable	
Studies involving experimental **animals**: State details of **authority granting ethics approval** (IRB or equivalent committee(s), provide reference number for approval. Include a statement of compliance with ethical regulations.	Not Applicable	
Studies involving **specimen and field samples:** State if relevant **permits** obtained, provide details of authority approving study; if none were required, explain why.	Not Applicable	

Dual Use Research of Concern (DURC)	Information included in the manuscript?	In which section is the information available? (Reagents and Tools Table, Materials and Methods, Figures, Data Availability Section)
Could your study fall under dual use research restrictions? Please check biosecurity documents and list of **select agents and toxins** (CDC): https://www.selectagents.gov/sat/list.htm	Not Applicable	
If you used a select agent, is the security level of the lab appropriate and reported in the manuscript?	Not Applicable	
If a study is subject to dual use research of concern regulations, is the name of the **authority granting approval and reference number** for the regulatory approval provided in the manuscript?	Not Applicable	

Reporting

The MDAR framework recommends adoption of discipline-specific guidelines, established and endorsed through community initiatives. Journals have their own policy about requiring specific guidelines and recommendations to complement MDAR.

Adherence to community standards	Information included in the manuscript?	In which section is the information available? (Reagents and Tools Table, Materials and Methods, Figures, Data Availability Section)
State if relevant guidelines or checklists (e.g., **ICMJE, MIBBI, ARRIVE, PRISMA**) have been followed or provided.	Not Applicable	
For **tumor marker prognostic studies,** we recommend that you follow the **REMARK** reporting guidelines (see link list at top right). See author guidelines, under 'Reporting Guidelines'. Please confirm you have followed these guidelines.	Not Applicable	
For **phase II and III randomized controlled trials**, please refer to the **CONSORT** flow diagram (see link list at top right) and submit the CONSORT checklist (see link list at top right) with your submission. See author guidelines, under 'Reporting Guidelines'. Please confirm you have submitted this list.	Not Applicable	

Data Availability

Data availability	Information included in the manuscript?	In which section is the information available? (Reagents and Tools Table, Materials and Methods, Figures, Data Availability Section)
Have **primary datasets** been deposited according to the journal's guidelines (see 'Data Deposition' section) and the respective accession numbers provided in the Data Availability Section?	Yes	Specified in the Data Availability section.
Were **human clinical and genomic datasets** deposited in a public access-controlled repository in accordance to ethical obligations to the patients and to the applicable consent agreement?	Not Applicable	
Are **computational models** that are central and integral to a study available without restrictions in a machine-readable form? Were the relevant accession numbers or links provided?	Yes	Specified in the Data Availability section.
If publicly available data were reused, provide the respective **data citations in the reference list**.	Not Applicable	
